# Indole-2-carboxamides Optimization for Antiplasmodial Activity

**DOI:** 10.1021/acsbiomedchemau.5c00058

**Published:** 2025-07-31

**Authors:** Malkeet Kumar, Anees Ahmad, Anna Caroline Campos Aguiar, Sarah El Chamy Maluf, Anwar Shamim, Mariana Ferrer, Guilherme E. Souza, Marcos L. Gazarini, Dhelio B. Pereira, Thomas W. von Geldern, Delphine Baud, Barry Jones, Susanta Kumar Mondal, Paul A. Willis, Rafael Victorio Carvalho Guido, Luiz Carlos Dias

**Affiliations:** † Institute of Chemistry, University of Campinas, Barão Geraldo, PO Box 6154, Campinas, SP 13083-970, Brazil; ‡ Sao Carlos Institute of Physics, University of Sao Paulo, IFSC − USP, 13566-590 Sao Carlos, SP, Brazil; § Department of Microbiology, Immunology and Parasitology, University Federal of Sao Paulo, Ed. Ciências Biomédicas - R. Botucatu, 862 - Vila Clementino, São Paulo, SP 04023-062, Brazil; ∥ Department of Biosciences, University Federal of Sao Paulo, Ed. Ciências Biomédicas - R. Silva Jardim, 136 - Vila Mathias, Santos, SP 11015-531, Brazil; ⊥ Centro de Pesquisa em Medicina Tropical, CEP 76812-329 Porto Velho, RO, Brazil; # Medicines for Malaria Venture, ICC, Route de Pré-Bois 20, P.O. Box 1826, 1215 Geneva, Switzerland; ∇ 576687 Pharmaron U.K., West Hill Innovation Park, Hoddesdon EN11 9FH, U.K.; ○ TCG LifeSciences PVT. LTD, Block-BN, Plot-7, Sector-V, Salt Lake Electronic Complex, Kolkata 700091, India

**Keywords:** malaria, antimalarials, indoles, resistance, drug development

## Abstract

Malaria still stands out as one of the most devastating and prevalent diseases globally, where the rise of resistance to different antimalarial drugs in different regions has posed significant obstacles to global treatment and elimination. Consequently, there is a pressing need for the development of new antimalarial agents with novel modes of action. In this study, we report the identification and optimization of new indole-2-carboxamide derivatives where structural modifications have yielded new compounds **6x** with enhanced potency (*Pf*3D7-IC50 ∼ 0.3 μM) and improved metabolic stability (hMics = 3 μL/min/mg), while also minimizing the human ether-a-go-go-related gene (hERG, IC_50_ > 20 μM) channel activity and cytotoxic effect on hepatic cells (CC_50_ > 30 μM). Mode-of-action investigations revealed that a representative compound from this series interfered with homeostasis of the parasite’s digestive vacuole. However, cross-resistance was observed with resistant strains, which was linked to efflux pumps such as *Plasmodium falciparum* chloroquine resistance transporter (*Pf*CRT). Despite this challenge, these indole-2-carboxamides provide versatile molecular templates for innovative medicinal chemistry to overcome cross-resistance while maintaining other attractive properties of this novel series.

## Graphical abstract

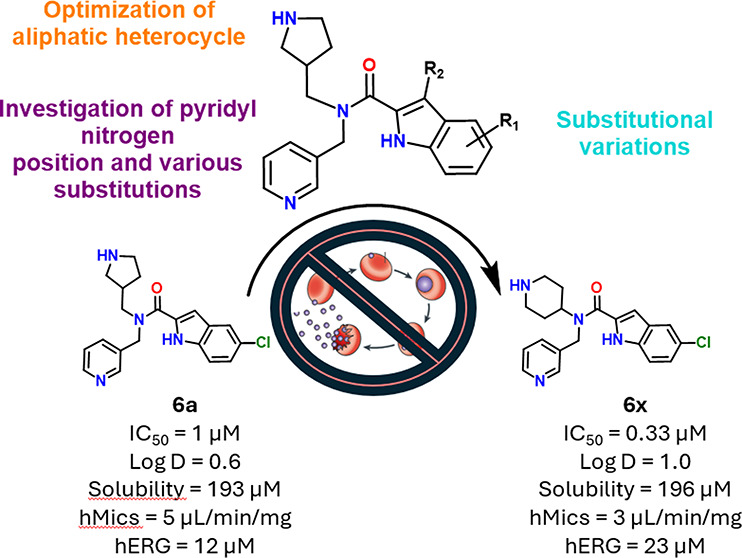

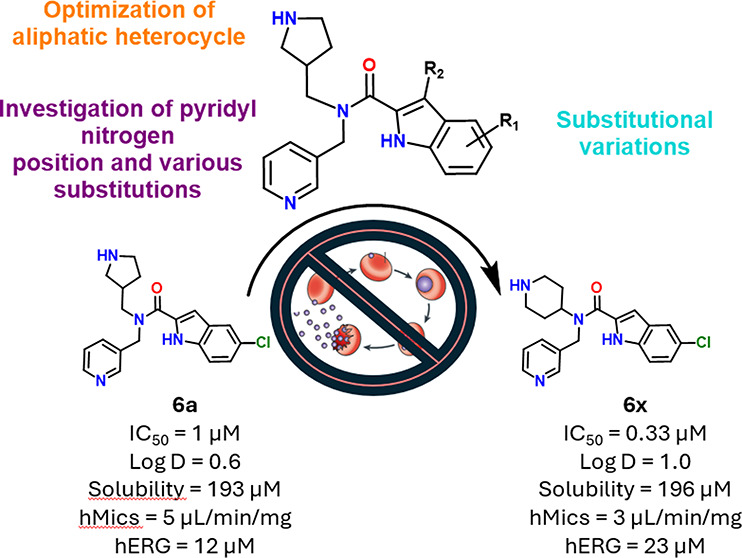

## Introduction

Malaria is caused by *Plasmodium* spp. parasites and is one of the most devastating and prevalent diseases worldwide. Approximately half of the world population is at risk of malaria, where in 2023, there were 263 million cases leading to an estimated 597,000 deaths.^1^ Countries with low economic development face a higher risk, with 94% of global cases originating from the World Health Organization (WHO) African region. The WHO Malaria Report of 2024 emphasizes the heightened vulnerability of infants and pregnant women in the WHO African Region, with children under the age of 5 accounting for 76% of malaria-related deaths. Although the percentage of total malaria deaths in children aged under 5 years decreased by 86.7% in 2000 and 73.7% in 2023, there has been no further improvement since then. Brazil along with Bolivarian, Republic of Venezuela, and Colombia accounts for 76.8% of overall cases in the WHO Region of the Americas.^1^

Malaria can be caused by six *Plasmodium* parasite species among which, *Plasmodium vivax* and *Plasmodium falciparum* are the most epidemiologically relevant and infectious, leading to severe illness and death, if not treated in a timely manner.^2^ However, malaria is curable with various drug regimens among which chloroquine-based combination therapies for chloroquine-sensitive malaria, and artemisinin-based combination therapies (ACTs) for chloroquine-resistant malaria are primarily employed for *P. falciparum* malaria uncomplicated infections. In combination therapies, the fast-acting inhibitors chloroquine (CQ, Figure 1) and artemisinin (ART, Figure 1) are partnered with a second antimalarial agent to minimize the risk of resistance development. However, like CQ, the continued emergence of *P. falciparum* resistance to ART could jeopardize further progress toward global malaria treatment and elimination goals.^3–5^ Therefore, the development of new antimalarial agents with novel modes of action and enhanced efficacy remain strategies to address drug resistance.^6^

**Figure 1:**
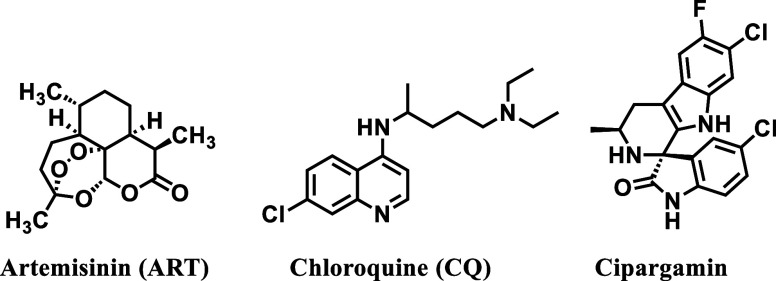
Structure of Cipargamin and major antimalarial agents.

There are number of strategies employed for the malaria drug discovery, which includes exploiting various pharmacophores from natural resources, drug repurposing, and optimization of hits identified from high-throughput phenotypic or target-based screens.^7–9^ In this study, we report the discovery and structure activity relationship (SAR) studies of novel indole-based, tertiary amide scaffolds with the potential for subsequent exploration into clinical candidates.

In addition to exhibiting pharmacological activities across different disease areas, indole derivatives are well-recognized for their proven antimalarial activity.^10^ For example, promising antimalarial agents based on indole scaffolds have recently been reported, including prenylated indole alkaloids, spiro-indolones, bis-indoles, conjugated indoles, amino indoles, piperidine indoles, and hybrid indole compounds.^2,11–23^ Notably, spiro-indolones have demonstrated multistage activity, targeting both asexual and gametocyte stages with Cipargamin (being developed by Novartis), currently in clinical development (Figure 1).^24,25^ Mechanistic investigations identified the target as selective inhibition of *Plasmodium falciparum* P-type cation-transporter ATPase 4 (*Pf*ATP4), a P-type Na^+^-ATPase located in the plasma membrane of the parasite, leading to a fatal disruption of sodium homeostasis. This underscores the potential of indole-containing pharmacophores with antimalarial profiles for development as novel antimalarial agents, potentially featuring a distinctive mode of action.^20^

## Results and Discussion

### Discovery of Indole Carboxamide Derivatives with Antiplasmodial Activity

High-throughput screening (HTS) of a Medicines for Malaria Venture (MMV) library of 39,995 commercially available compounds against *P. falciparum* 3D7 strain (*Pf*3D7) led to identification of the initial hit **6a** (Table 1). This library was designed to maximize chemical diversity and novelty of compounds with physicochemical properties consistent with potential for further development. For example, the indole carboxamide derivative **6a** showed encouraging antiplasmodial activity (*Pf*3D7-IC_50_ = 1.39 μM; *Pf*NF54-IC_50_ = 0.84 μM). Further profiling (Table 1) highlighted acceptable solubility (193 μM at pH-7.4), low hERG channel activity (IC_50_ = 11.65 μM) with selectivity (SI ∼ 14) and good metabolic stability (rat hepatocyte intrinsic clearance: 14.6 μL/min/10^6^ cells, and human microsomal intrinsic clearance: 4.6 μL/min/mg). Additionally, the rate of kill was measured *in vitro* using the parasite reduction ratio (PRR) assay, which showed that it was as fast as that of CQ, representing an additional attractive feature for the development of novel clinical agents.^26^

**Table 1:**
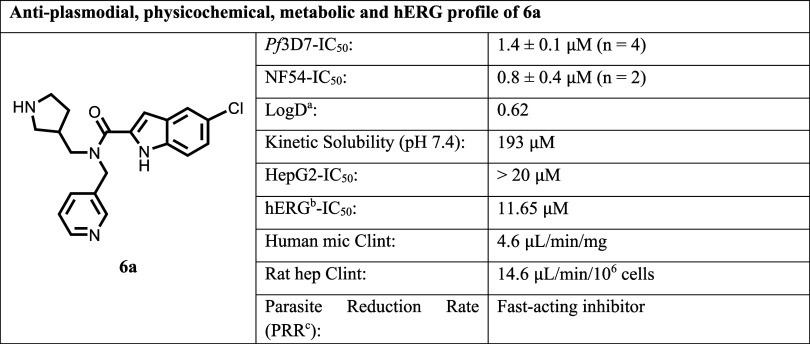
Profile of the Novel Indole-2-carboxamide (**6a**)

a LogD was determined at pH7.4 using the miniaturized shake flask method.

b Human Ether-a-go-go-Related Gene, IC_50_ was determined using automated patch-clamping method;

c Parasite reduction rate.^26–28^

### Structure–Activity Relationship Studies (SAR) Approach

SAR investigations were directed toward three key structural components (e.g., indole, pyridyl, and pyrrolidine) exemplified by **6a** (Figure 2) to explore the significance of these motifs in relation to pharmacology and physicochemical properties. First, the *R*- and *S*-enantiomers of **6a** were isolated and assessed against *P. falciparum* where both exhibited comparable potencies (Table 2). It is worth noting that enantiomers with similar potency may exhibit differences in nonspecific binding, which could influence their pharmacokinetic and pharmacodynamic profiles. Consequently, all subsequent chiral analogues were initially evaluated as racemates. Subsequently, indole variants were designed to discern the most suitable substitutions and their positions on the indole moiety. Additionally, in a small set of analogues, the indole scaffold was replaced with imidazole, benzimidazole, benzyl, and indazole to explore whether scaffold hopping could lead to an enhancement of potency. Next, the significance of the pyridyl motif was explored via variation of the ring nitrogen position and assessing replacements with benzyl and aliphatic amines. SARs around the pyrrolidine motif aimed to determine the significance of the basic nitrogen center and the methylene linker in relation to potency. Additionally, replacement with piperidine was explored to eliminate the original chiral center to determine any effect on antiplasmodial activity.

**Figure 2:**
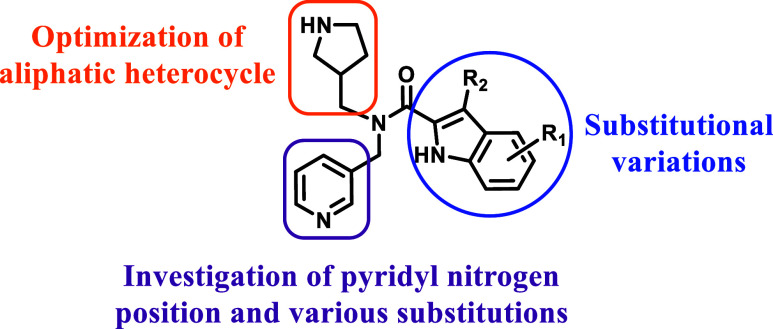
Initial SAR investigation for **6a**.

### Chemistry

The synthesis of indole-2-carboxamide analogues commenced by the reductive amination of commercially available amines **1** and aldehydes **2** to obtain intermediate **3** (Scheme 1). The nucleophilic displacement of the tosylated pyrrolidin-2-one **9** (prepared from **8**) with pyridine amine **10** afforded intermediate **11.** These amines (**3** and **11**) were further reacted with various acids to obtain amides **5**. Deprotection where necessary, provided indole-2-carboxamides **6**. The *N*-methylated analogue **7a** was obtained via reductive amination of **6a** with aqueous formaldehyde and NaCNBH_3_.

**Scheme 1:**
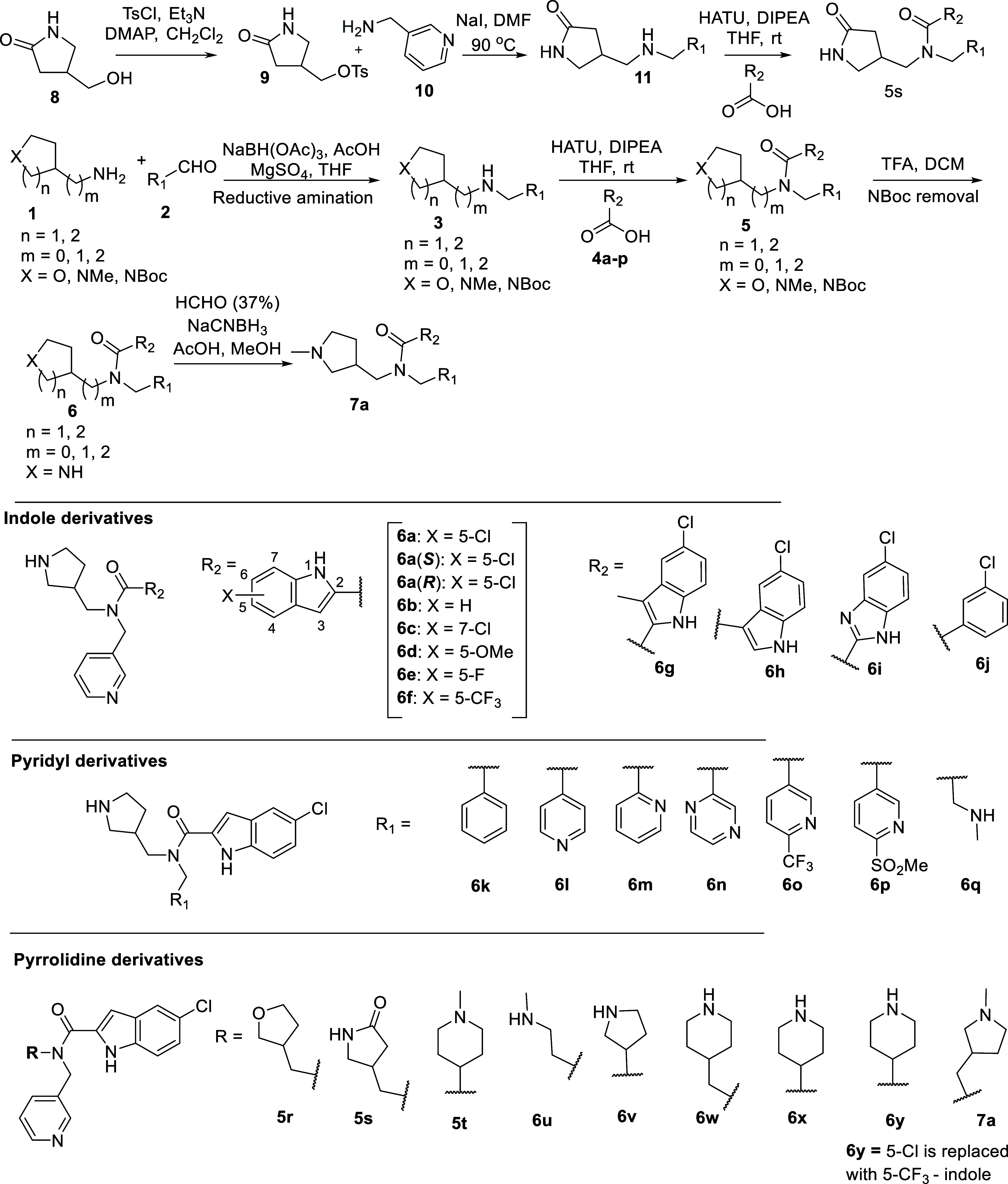
Synthetic Route for Indole-2-carboxamide Analogues

Analogues **6a**–**j** were synthesized to investigate the effects of substituted indole moiety, such as Cl, F, CF_3_, and OMe, as well as the replacement of indole ring with other heterocycles on *P. falciparum* inhibition. Similarly, variation of the pyridyl ring and incorporation of the respective amines were achieved during the first step of reductive amination, leading to compounds **6k**–**p**, **5r**–**5t**, **6u**–**6y**, and **7a**.

### Structure Activity Relationship (SAR) Studies

All novel compounds were screened against *Pf*3D7 with most were also tested for solubility, lipophilicity (LogD), and hERG channel activity. Representative compounds were also evaluated for metabolic stability and cytotoxicity based on structural diversity and pharmacology profile (*Pf*3D7-IC_50_ < 10 μM) (Tables 2–4). The initial set of analogues developed by the replacement of the 5-Cl indole scaffold with benzimidazole (**6i**, *Pf*3D7-IC_50_ > 10 μM) and 3-Cl-phenyl (**6j**, *Pf*3D7-IC_50_ > 25 μM) moieties resulted in complete loss of potency (*Pf*3D7-IC_50_ > 10 μM), demonstrating the bicyclic indole is imperative for antiplasmodial activity (Table 2). The unsubstituted indole analogue **6b** (*Pf*3D7-IC_50_ = 8.3 μM) showed a decrease in potency, emphasizing the importance of ring substitution. For example, the trifluoromethyl substitution for chlorine was tolerated with **6f** (*Pf*3D7-IC_50_ = 0.4 μM) exhibiting 2-fold higher potency than initial hit **6a** and low cytotoxic effect on human hepatocellular carcinoma cells (HepG2 cell line, CC_50_ > 30 μM). However, the 5-fluoro (**6e**, *Pf*3D7-IC_50_ = 8.8 μM) and 5-methoxy (**6d**, *Pf*3D7-IC_50_ > 10 μM) substituents were not tolerated and showed significant loss in potency. Additionally, the carboxamide analogues (**6g**, *Pf*3D7-IC_50_ = 5.8 μM and **6h**, *Pf*3D7-IC_50_ > 10 μM**)**, 7-chloro regio-isomer (**6c**, *Pf*3D7-IC_50_ = 2.2 μM), underscored that position 5 on indole for substitution and position 2-for carboxamide linkage are the most suitable for this indole series of compounds. The assessment of hERG inhibition for these indole derivatives revealed that 5-chloro substitution is most suitable as the initial hit **6a** showed the best combination of properties with a hERG/*Pf*3D7 selectivity index of 12. Replacement of the 5-chloro with OCH_3_ **(6d**, hERG-IC_50_ = 0.8 μM) and CF_3_ (**6f**, hERG-IC_50_ **=** 1.2 μM) increased hERG channel activity by 14- and 9-fold, respectively. Additionally, 7-Cl substitution also led to increased hERG channel activity (**6c**, hERG-IC_50_ = 1.6 μM) but with minor change in antiplasmodial potency. Surprisingly, a disconnect between lipophilicity and hERG channel activity was also observed as analogues **6c** (hERG-IC_50_ = 1.6 μM) and **6d** (hERG-IC_50_ = 0.8 μM) with lower log D values (>3-fold) than **6a** (Log D = 0.62) showed higher hERG channel activity (>7-fold) while **6f** (hERG-IC_50_ = 1.2 μM) with a log D value of 1.1 demonstrated a 10-fold greater hERG inhibition. All indole analogues exhibited good solubility (>190 μM). In conclusion, the 5-chloro indole scaffold was retained due to its potency, lower hERG channel activity and metabolic stability.

**Table 2:**
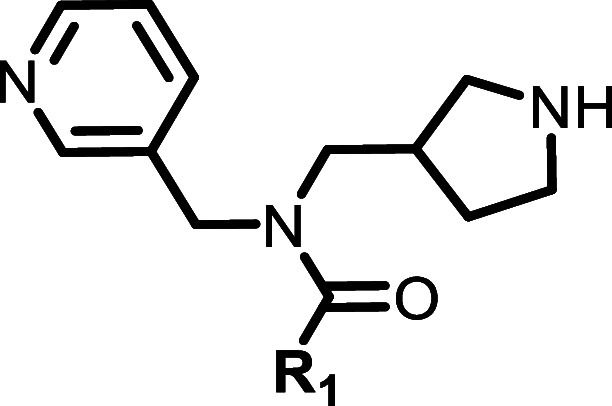

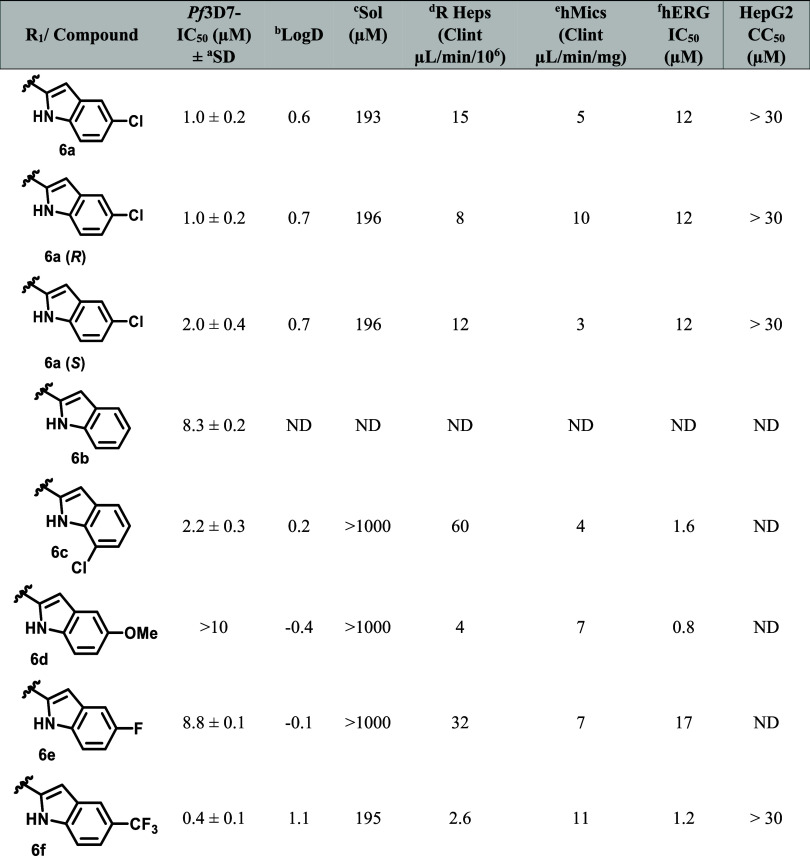
*In Vitro* Antiplasmodial Activity, Solubility, Lipophilicity, Metabolic Stability, and hERG Activity of Indole Carboxamides with Modification in Indole Motif

a SD: Standard deviation.

b LogD was determined at pH7.4 using the miniaturized shake flask method.

c Sol: kinetic solubility at pH 7.4.

d R Heps: Rat hepatocyte stability.

e hMics: human microsome stability.

f Human Ether-a-go-go-Related Gene, IC_50_ was determined using automated patch-clamping method; ND: Not determined.

SAR around the pyridyl motif demonstrated that the 3-nitrogen atom was required for antiplasmodial activity as the benzyl analogue **6k** (*Pf*3D7-IC_50_ = 6.7 μM) demonstrated a loss in activity (Table 3). Additionally, the inactivity of regio-isomers **6l** and **6m** (*Pf*3D7-IC_50_ > 10-fold) compared to **6a** confirmed that 3-pyridyl is the most suitable substituent for antiplasmodial potential. The pyrazine **6n** (*Pf*3D7-IC_50_ > 10 μM) also showed a loss in potency and confirmed that an additional nitrogen atom was not tolerated. Similarly, analogues **6o** (*Pf*3D7-IC_50_ = 4.3 μM) and **6p** (*Pf*3D7-IC_50_ > 10 μM) with substituents (CF_3_ and SO_2_Me) in the 3-pyridine ring also did not improve potency. The replacement of pyridyl with *N*-methyl propyl amine **6q** (*Pf*3D7-IC_50_ > 10 μM) resulted in a complete loss of potency, underscoring the essential role of the heteroaromatic pyridyl moiety. Consequently, the 3-pyridinebenzyl substituent was retained for modification of the pyrrolidine motif in subsequent analogues.

**Table 3:**
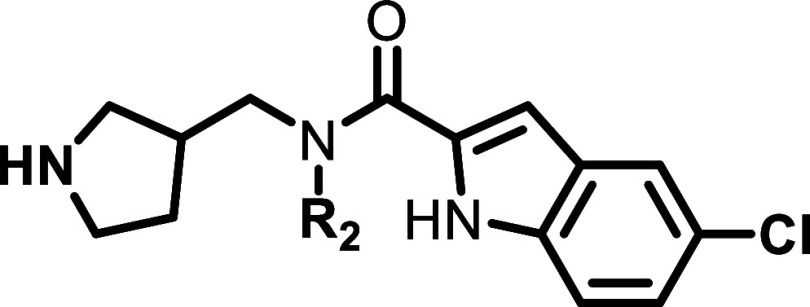

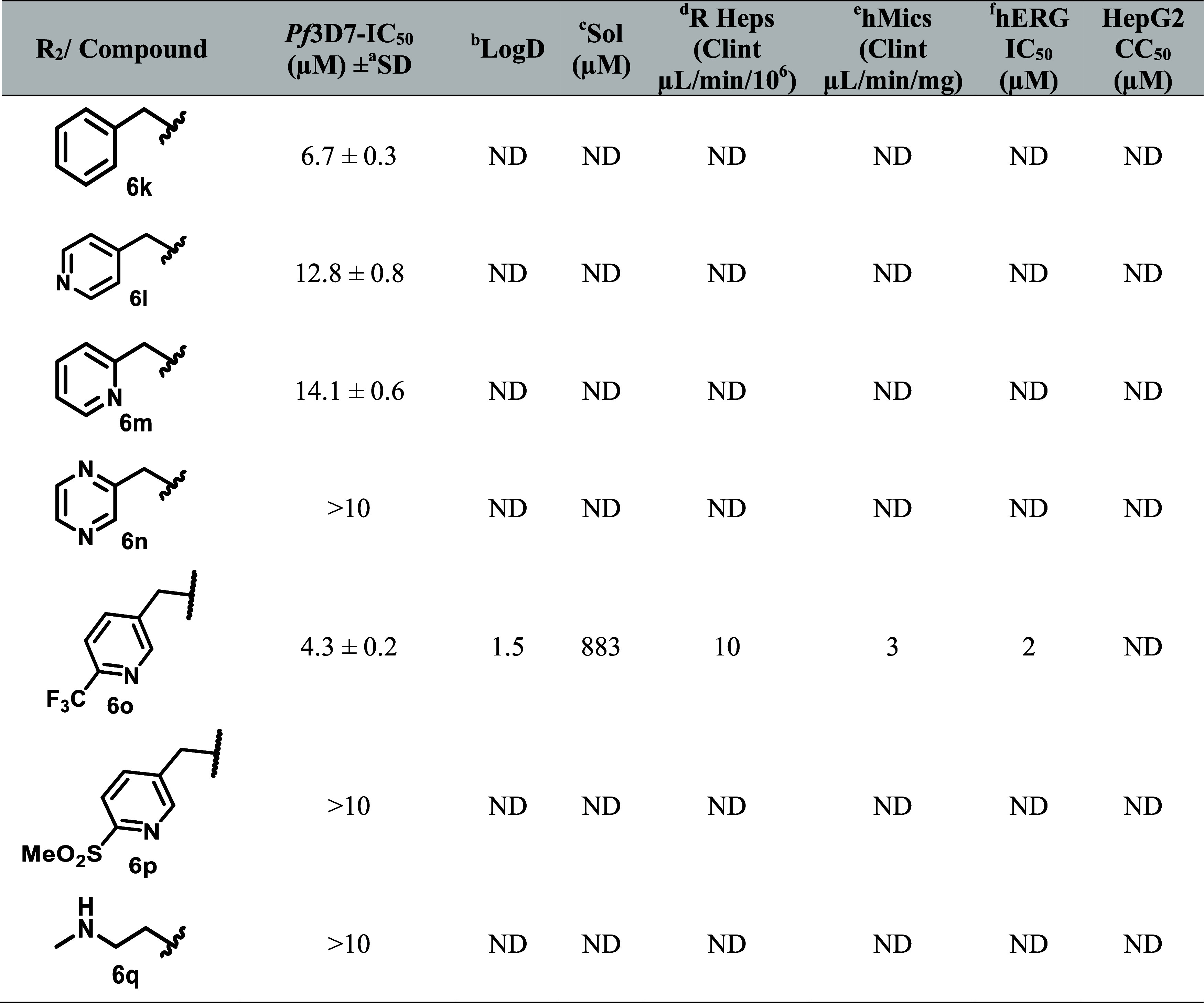
*In* *Vitro* Antiplasmodial Activity, Solubility, Lipophilicity, Metabolic Stability, and hERG Activity of Indole Carboxamide Analogues with Modification in Pyridyl Motifs

a SD: Standard deviation.

b LogD was determined at pH7.4 using the miniaturized shake flask method.

c Sol: kinetic solubility at pH 7.4.

d R Heps: Rat hepatocyte stability.

e hMics: human microsome stability.

f Human Ether-a-go-go-Related Gene, IC_50_ was determined using automated patch-clamping method; ND: Not determined.

Cyclic pyrrolidine and basic nitrogen are imperative features for inhibitory activity, as a complete loss in activity was observed with the replacement of pyrrolidine with tetrahydrofuran **5r** (*Pf*3D7-IC_50_ > 10 μM) or pyrrolidinone **5s** (*Pf*3D7-IC_50_ > 10 μM) while *N*-substitution (**7a**, *Pf*3D7-IC_50_ = 0.46 μM) led to a slight increase in activity (Table 4). The acyclic *N*-methyl ethyl replacement was tolerated with 2-fold decreased potency (**6u**, *Pf*3D7-IC_50_ = 1.8 μM) relative to **6a**. However, this compound showed decreased metabolic stability and high hERG (hERG-IC_50_ = 1 μM) channel activity. Removing the methylene linker showed retention of activity (**6v**, *Pf*3D7-IC_50_ = 0.57 μM). Replacement of the 3-pyrrolidinyl methyl moiety with a piperidinyl methyl, devoid of a chiral center, afforded compounds with retention of potency (**6w**, *Pf*3D7-IC_50_ = 0.9 μM), which is significant for novel antimalarials where a low cost of goods is critical for any drug candidates.^29^ In the piperidine subseries, removal of the methylene linker (**6x**, *Pf*3D7-IC_50_ = 0.33 μM), *N*-methylation (**5t**, *Pf*3D7-IC_50_ = 0.37 μM; CC_50_ > 30 μM) and replacement of the 5-Cl-indole with a 5-CF_3_-substituent (**6y**, *Pf*3D7-IC_50_ = 0.22 μM; CC_50_ > 30 μM) improved potency and demonstrated a similar SAR to the 3-pyrrolidinyl subseries. Except compound **6x**, piperidine (**5t**) and *N*-methylpiperidine (**6y**) containing compounds showed lower metabolic stability highlighting that methyl substituent on piperidine and replacement of 5-Cl on indole motif with CF_3_ increased the susceptibility toward metabolism. In addition to good solubility (≥100 μM) demonstrated by all the piperidine-containing compounds, **6x** showed low susceptibility toward metabolism in both rat hepatocytes (2 μL/min/10^6^) and human microsomes (3 μL/min/mg), and improved metabolic stability compared to the initial pyrrolidine **6a**. Consequently, the 4-piperidine analogue **6x** was the most promising *P. falciparum* inhibitor with potent inhibitory activity against the parasite, low hERG channel activity, good solubility, and improved metabolic stability.

**Table 4:**
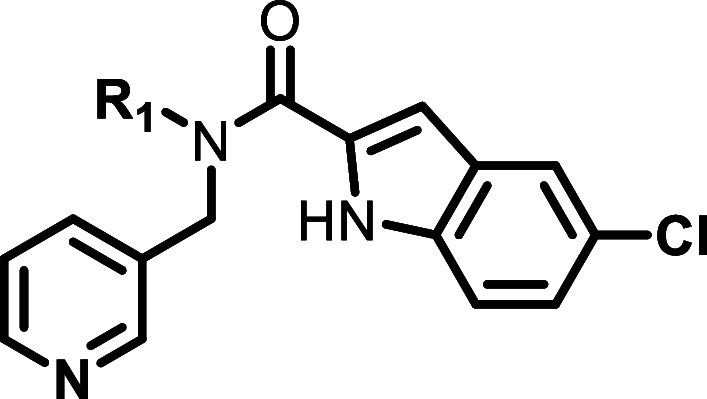

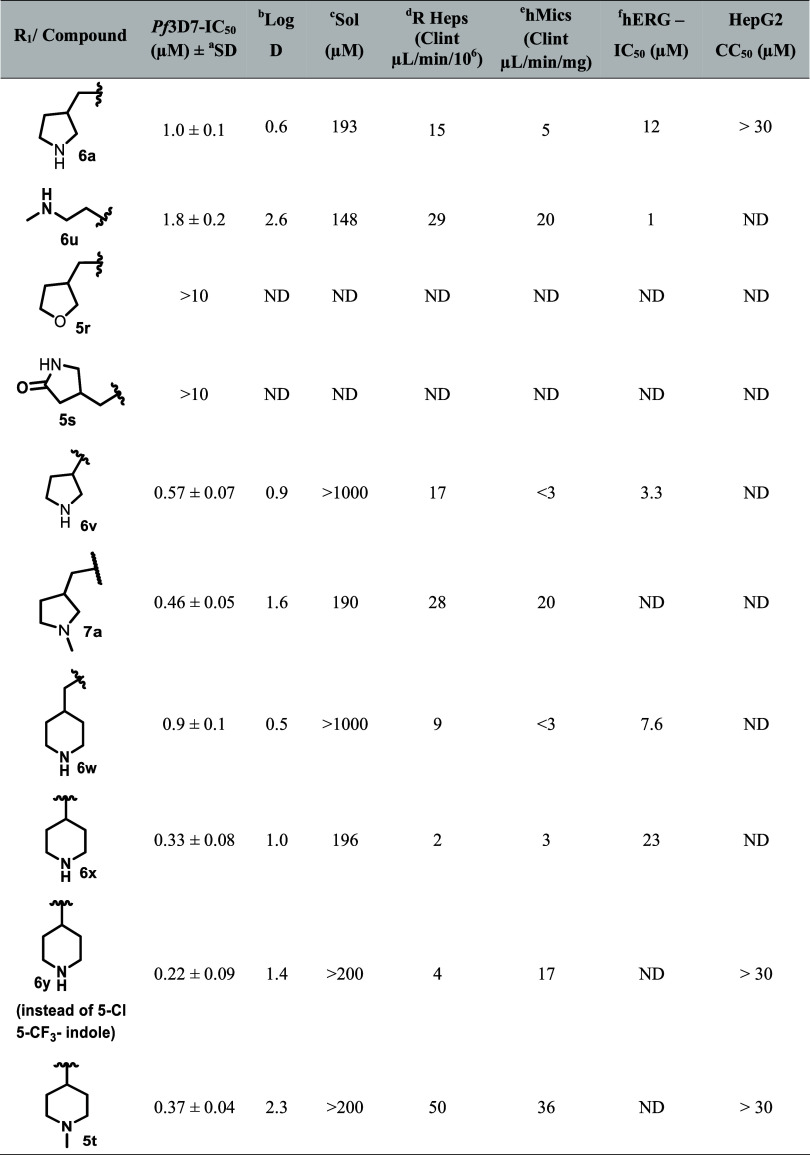
*In Vitro* Antiplasmodial Activity, Solubility, Lipophilicity, Metabolic Stability, and hERG Activity of Indole Carboxamides with Modification in Pyrrolidinone Motif

a SD: Standard deviation.

b LogD was determined at pH7.4 using the miniaturized shake flask method.

c Sol: kinetic solubility at pH 7.4.

d R Heps: Rat hepatocyte stability.

e hMics: human microsome stability.

f Human Ether-a-go-go-Related Gene, IC_50_ was determined using automated patch-clamping method; ND: Not determined.

### Assessment of Indole Carboxamide 6f throughout the Asexual Parasite’s Life Cycle and Gametocytes Inhibition

To gain deeper insights into the parasitological profile of the indole carboxamide series, we compared the asexual blood stage-specificity profile of **6f** (Figure 3A–B) to CQ (Figure 3D–E). In this assay, synchronized parasites (3D7 strain) were exposed to a range of compounds concentrations for 8 h (hours) during the early ring, late ring, early trophozoite, late trophozoite, and schizont blood stages. Cultures were followed over 60 h to allow parasite development in the absence of compound, extending through to invasion of new red blood cells (RBCs) and development until the trophozoite stage. Both **6f** and CQ were classified based on their timing of peak activity, defined as the asexual blood stage at which the compounds showed the lowest IC_50_^8h^ values. **6f** showed increased potency on the early ring forms, with an IC_50_^8h^ value comparable to the IC_50_^72h^ value (Figure 3A,B). Light microscopy confirmed that the various times of inhibitor exposure corresponded to the different malaria developmental stages, indicating that all asexual blood stages were profiled (Figure 3C). As expected, CQ showed minor variation in IC_50_^8h^ values throughout the ring and trophozoite stages and were consequently classified in the group with peak activity at ring and trophozoite stages (Figure 3D,E). These findings indicated that **6f** acted in the early stages of parasite development, thereby providing valuable insights into the asexual blood stage dynamics of the compounds.

**Figure 3:**
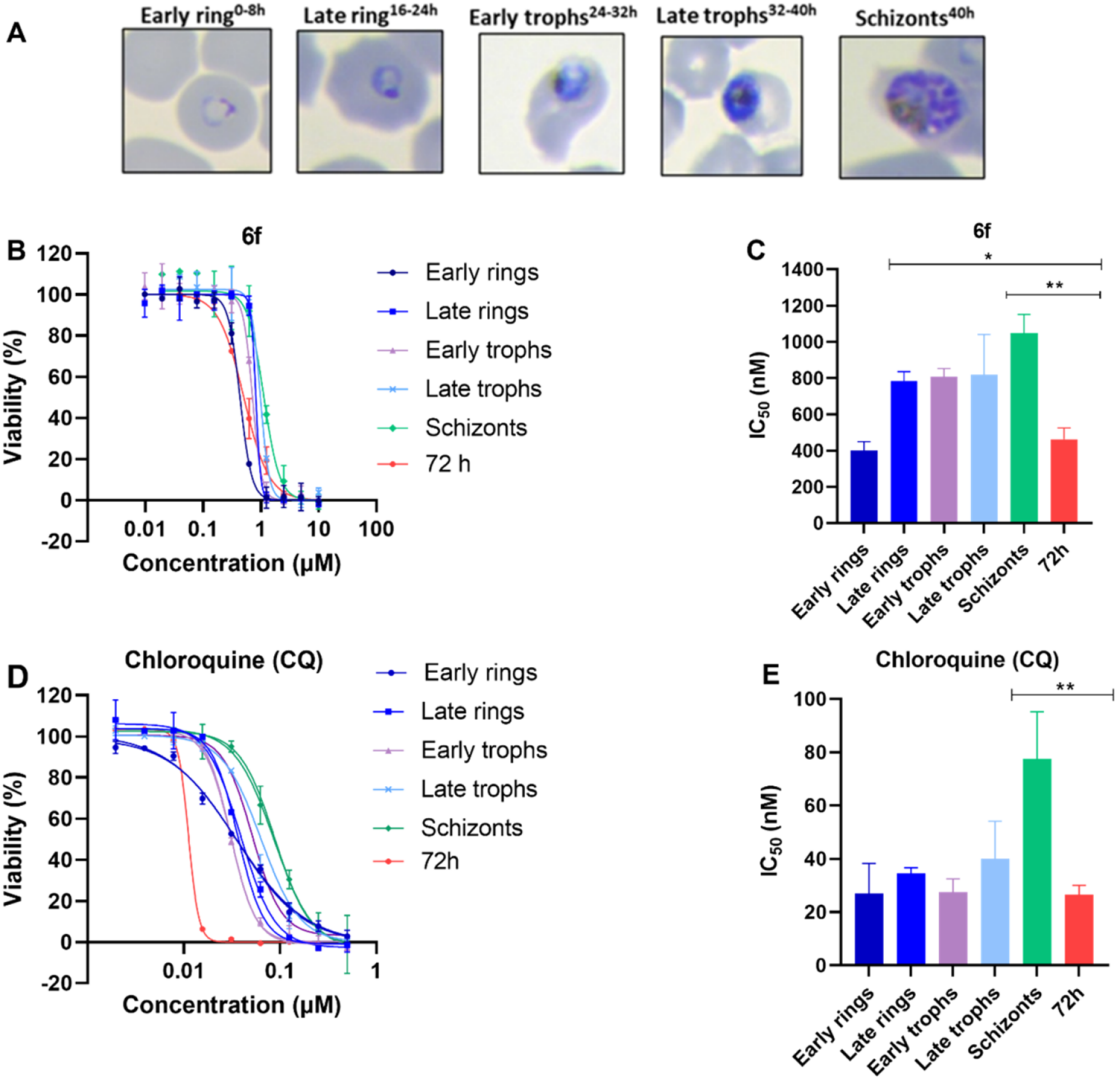
(A) Light microscopy of Giemsa-stained blood smears of each 8 h of exposure corresponded to the different *P. falciparum* developmental stages. Asexual blood stage susceptibility profiles for **6f** (B and C) and CQ (D and E). Bar graphs indicate mean IC_50_^8h^ values, whereas viability graphs show the most representative curves from independent repeats. Error bars indicate the standard error of the mean based on two independent experiments. Statistical analysis was performed using two-way ANOVA followed by Dunnett’s multiple comparisons test. Statistical significance was defined as *p* < 0.1 (*), *p* < 0.01 (**).

Next, **6f** was evaluated in an established *P. falciparum* dual gamete formation assay (*Pf* DGFA). The *Pf* DGFA evaluates the ability of the molecules to prevent male and female gametocytes from differentiating into gametes *in vitro*, which is the first step of parasite development in the mosquito.^30^ **6f** was tested against male and female *P. falciparum* gametocytes at 1 μM in the *Pf* DGFA. At this concentration, no significant inhibition of male and female gametocytes was observed. The compound showed only 28% inhibition against male gametocytes and 7% against female gametocytes.

### Antiplasmodial Activity of Indole Carboxamide 6f against a Panel of Resistant Strains of *P. falciparum*

Compound **6f** was screened against a representative panel of sensitive and multidrug-resistant (MDR) strains of the parasite. The panel included MDR strains RF12, Dd2, K1, 7G8, TM90–2CB,^31^ and drug-sensitive NF54 strains. Compounds with a resistance index (RI) value of less than 3 are not classified as cross-resistant. An RI value between 3 and 5 indicates moderate resistance, while an RI value greater than 5 signifies a high resistance index. **6f** showed cross-resistance against all the resistant parasites evaluated (Table 5). In this sense, the RI values of the tested compounds varied from 5 to 20. These findings led us to suspect that these indole carboxamide compounds could be acting via the parasite’s digestive vacuole in a similar mode of action to CQ, considering that all evaluated strains possess mutations in the *pact* gene, which is associated with CQ resistance.

**Table 5:** *In Vitro* Inhibitory Activity of Compound **6f** against Resistant *P. falciparum* Strains (RF12, K1, Dd2, 7G8, TM90-2CB) and the Drug-Sensitive Strains NF54 and 3D7, Including the Corresponding Resistance Index (RI) for Each Strain

**strain**	**6f IC**_**50**_ (μM) ± SD	**resistance index** ^a^
RF12	2.0 ± 0.3	5
K1	7.3 ± 0.2	20
Dd2	2.6 ± 0.4	7
7G8	1.8 ± 0.3	5
TM90-2CB	2.8 ± 0.5	8
3D7	0.4 ± 0.1	1
NF54	0.37 ± 0.06	

a Resistance index (RI) values were calculated as the ratio of the IC_50_ between the resistant strain and the susceptible strain NF54. Dd2 has the N86F mutation in the *pfmdr1* gene; mutations M74I, N75E, and K76T in the *pfcrt* gene; N51I, C59R, and S108N in the *pfdhfr* gene; and S436F, A437G, and A613S in the *pfdhps* gene. K1 has the N86F mutation in the *pfmdr1* gene; mutations M74I, N75E, and K76T in the *pfcrt* gene; N51I, C59R, and S108N in the *pfdhfr* gene; and A437G and A613S in the *pfdhps* gene. 7G8 has Y184F, S1034R, N1042D, and D1246Y mutations in the *pfmdr1* gene; mutations C72S, M74I, N75E, and K76T in the *pfcrt* gene; N51I and S108N in the *pfdhfr* gene; and S436F and A437G in the *pfdhps* gene. TM90–2CB has the Y268S mutation in the *pfcytb* gene; Y184F in the *pfmdr1* gene; N51I, C59R, and S108N in the *pfdhfr* gene; and S436F, A437G, and A581G in the *pfdhps* gene. The *pfmdr1* copy number was 1 for the wild-type strain 3D7 and 3 for Dd2.

To assess the susceptibility of other compounds in the series to the resistant Dd2 and K1 strains (resistant to chloroquine, mefloquine, and sulfadoxine), eight representative analogues were evaluated in parallel with the 3D7 strain (chloroquine-sensitive). A color-coded Table 6 indicates the cross-resistance ratios assessed for the indole derivatives. Among the compounds tested, **6x**, **6a**, **6f**, and **6y** derivatives exhibited fold-shifts in the IC_50_ values against the resistant strains greater than 5, suggesting a cross-resistance profile against Dd2 and K1 strains. The other analogues, namely **6c**, **5t**, **6g**, and **7a**, also showed cross-resistance; however, lower levels of IC_50_ fold-shifts were observed in Dd2 and K1, all below 5-fold related to 3D7 (Table 6). These findings underline that cross-resistance is a prominent characteristic of the series. Despite efforts to introduce structural variations, the observed cross-resistance remained a limitation within the series, emphasizing the need for further exploration and optimization in any future antimalarial drug development efforts.

**Table 6:**
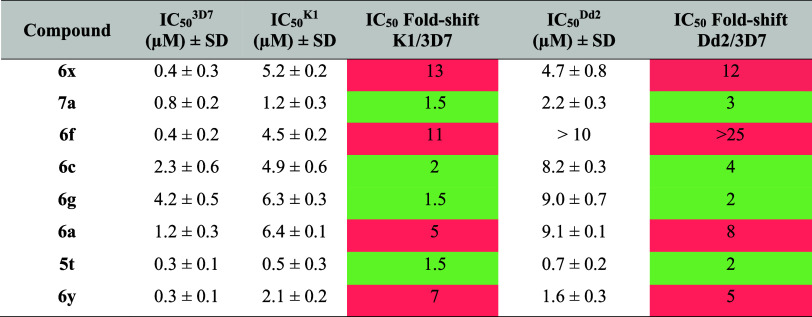
IC_50_ Values of Indole Compounds against a Sensitive (3D7) and Resistant (K1 and Dd2) *P. falciparum* Strains

### Verapamil Reversed the Resistance of *P. falciparum* (Dd2) to Indole Carboxamide **6f** and **6x**

Based on the observed decreased potency profile of the indole carboxamide derivatives (**6x**, **7a**, **6f**, **6c**, **6g**, **6a**, **5t** and **6y**) against the resistant strains of the parasite and their relationship with the mutated *pfcrt* gene, we further investigated the susceptibility of selected derivatives (e.g., **6f** and **6x)** to efflux pumps (Figure 4). Aiming to determine whether these compounds exhibit a resistance mechanism comparable to that of CQ, which is known to be susceptible to efflux pumps, we assessed the inhibitory activity of the indole carboxamide derivatives in the presence and absence of verapamil (VP), a known efflux pump inhibitor.^32^ In this assay, the addition of verapamil is expected to reverse the resistance of *P. falciparum* toward the CQ and restore the inhibitory activity. Thus, CQ was included as a positive control and displayed potent antiplasmodial activity, with IC_50_ values against 3D7 parasites of 40–50 nM. The inhibitory activity of CQ decreased by 17-fold when tested against the CQ-resistant Dd2 strain (Figure 4A,B). CQ-resistant parasites could be partially affected *in vitro* by VP, due to the ability of VP to inhibit the efflux of CQ via mutant *Pf*CRT. As expected, the presence of 10 μM of VP decreased the CQ-IC_50_ by 10-fold in the Dd2 strain and showed no effect in the CQ-sensitive 3D7 strain (Figure 4A,B).

**Figure 4:**
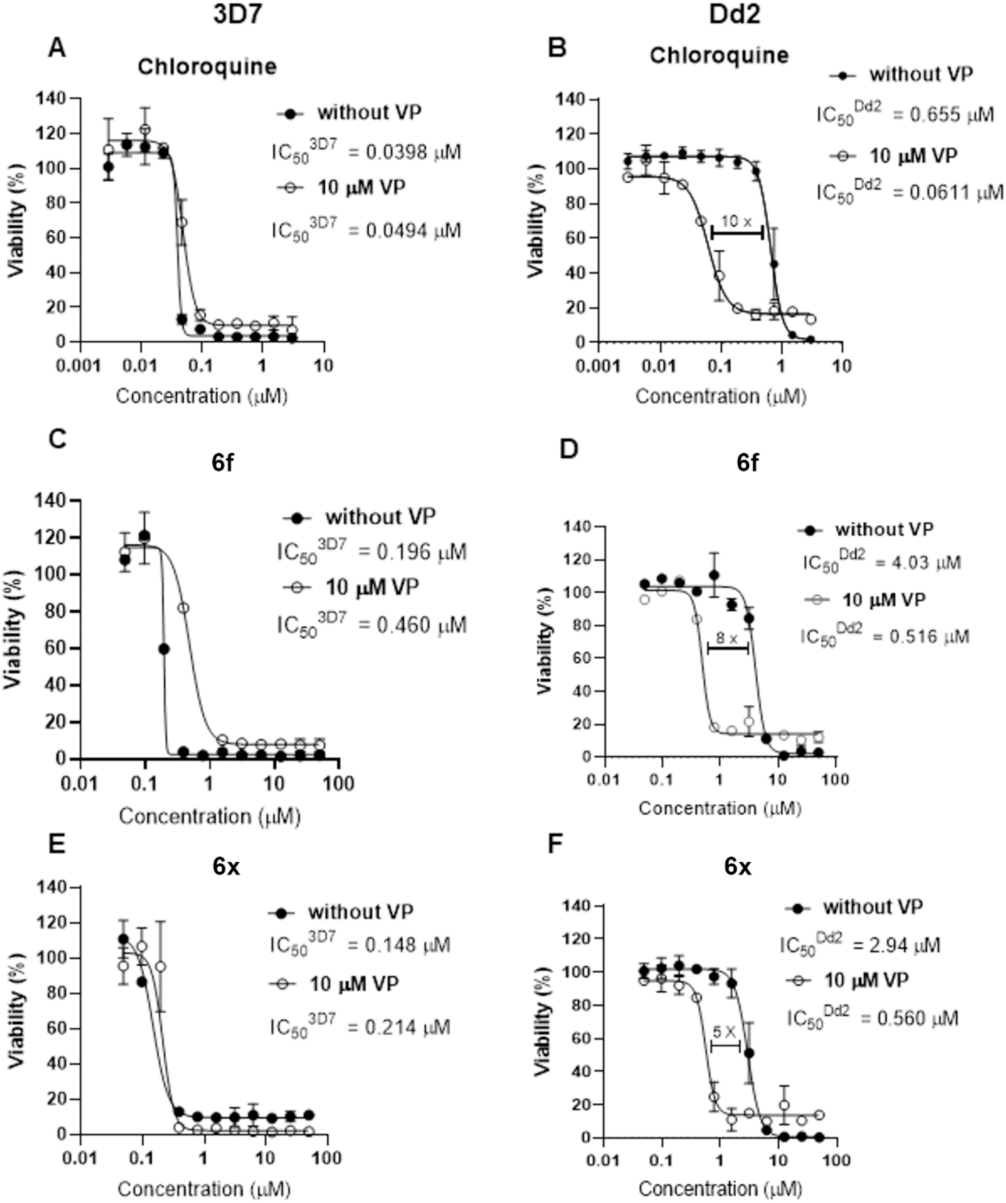
Inhibitory activity of CQ, **6f**, and **6x** in *P. falciparum* 3D7 (sensitive, left-handed plots) and Dd2 (resistant, right-handed plots) strains in the presence and absence of verapamil (VP). IC_50_ values of CQ (A), **6f** (C), and **6x** (E) in 3D7 strain in the absence (black dots) or presence (white dots) of 10 μM of VP. IC_50_ values of chloroquine (B), **6f** (D), and **6x** (F) in Dd2 strain in the absence (black dots) or presence (white dots) of 10 μM of VP.

The indole derivatives **6f** and **6x** presented IC_50_ values of, approximately, 200 nM and 150 nM, in 3D7 strain, respectively (Figure 4,E). These values increased by 20-fold in the CQ-resistant Dd2 strain (Figure 4D,F). The presence of 10 μM of VP decreased the **6f** and **6x** IC_50_ values by, approximately, 8- and 5-fold in the Dd2 strain, respectively (Figure 4D,F). As expected, no significant changes were observed in the 3D7 strain (Figure 4C,E). These findings suggested that the decreased susceptibility of CQ-resistant parasite Dd2 to the indole derivatives was due to the interaction of these inhibitors with the mutant isoforms of *Pf*CRT.

### Indole Carboxamide **6d** and **6f** Interfere with the Parasite’s Digestive Vacuole Homeostasis

Since PfCRT localizes the parasite’s digestive vacuole, we assessed the interaction of representative inhibitors (e.g., **6f** and **6d**) with this organelle in the parasite using the lysosomotropic probe acridine orange (AO) and CQ as control.^33^ Acridine orange accumulates within acidic organelles, such as lysosomes and the parasite’s food vacuole, emitting red fluorescence (>560 nm). Fluorometric analyses revealed that **6f** interferes with proton (H^+^) homeostasis, as indicated by an increase in cytoplasm fluorescence following treatment, resulting in reduced acidity in digestive vacuole after efflux of H^+^ (Figure 5). The same observation was seen for **6d**. An intriguing finding was that **6d** showed poor inhibitory activity against the parasite (IC_50_ > 10 μM, Table 2); however, it exhibited a strong interaction with the parasite’s digestive vacuole. The data supports the SAR findings about the role of basic nitrogen in antiplasmodial activity, possibly through a bioaccumulation process during intraerythrocytic stages, and suggest that compound **6d** may have different binding properties on the molecular target within the organelle. The digestive vacuole of *P. falciparum* is the site for hemoglobin digestion and heme detoxification. The hydrolysis of hemoglobin within the malaria parasite’s digestive vacuole occurs through the integrated action of aspartic, cysteine, and metalloproteases, resulting in the formation of hemozoin (malaria pigment), a biocrystal from the toxic precursor ferriprotoporphyrin IX (FPIX). Our findings indicated that the indole carboxamide derivatives act in the digestive vacuole of the parasite, impacting multiple pathways related to the parasite’s metabolism.

**Figure 5:**
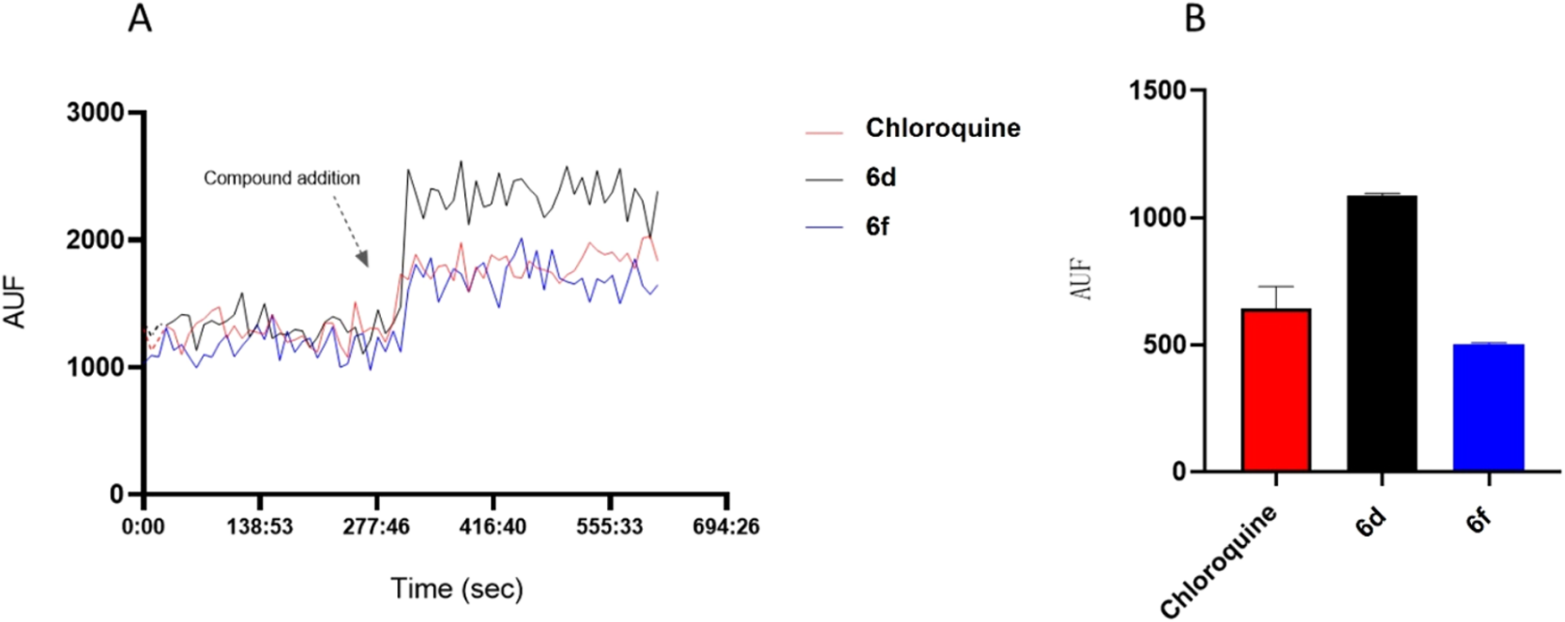
Effect of **6d, 6f**, and chloroquine on intracellular acridine orange (AO) mobilization from acidic compartments of isolated *P. falciparum* parasites. (A) The lysosomotropic fluorochrome AO (5 μM) was added in the isolated parasites (10^7^ cells mL^–1^) solution. 10 μM of **6d, 6f**, and chloroquine were added during AO fluorescence acquisition in a spectrofluorometer cuvette. Fluorescence intensities (arbitrary units fluorescence AUF) represent at least three different cell preparations. (B) Histogram of AO fluorescence (red channel) with mean ± SD (*n* = 3), representing the mean fluorescence difference from the initial fluorescence (f0) and final fluorescence (f1).

### Indole Carboxamide 6f is Active against *P. vivax* Isolates

We performed *ex vivo* activity assays against *P. falciparum* and *P. vivax* field isolates from Brazil. Compound **6f** was tested against 9 *P. vivax* and 7 *P. falciparum* Brazilian clinical isolates. It showed activity against *P. vivax* field isolates, with a median EC_50_ value of 698 nM and a range of 636 to 910 nM across individual isolates. Against the *P. falciparum* 3D7 clone, the EC_50_ was 363 nM, which was comparable to the values observed for *P. vivax* isolates (Table 7). However, compound **6f** was inactive against *P. falciparum* field isolates at a concentration of 10 μM. The *P. vivax* isolates were sensitive to artesunate and chloroquine, with EC_50_ values of 1 nM and 295 nM, respectively. The *P. falciparum* isolates were also sensitive to artesunate, with a low EC_50_ of 0.6 nM, but showed reduced sensitivity to chloroquine, with a median EC_50_ of 1202 nM. The results for *P. falciparum* are consistent with those observed in experiments using laboratory strains. It is important to note that in the study region (Porto Velho), the *P. falciparum* isolates are resistant to chloroquine, presenting mutations in codons 72 and 76 of the pfcrt gene.

**Table 7:** *P. vivax* and *P. falciparum* *Ex Vivo* Drug Susceptibility for **6f** Compound and Antimalarial Controls

		**median IC**_**50**_ **nM (range)**	
**compound**	*P. falciparum* **3D7 IC**_**50**_ **nM**	*P. vivax* **(9)**	*P. falciparum* **(7)**
**6f**	363	698 (363–910)	>10,000
**artesunate**	8	1 (0.6–1.6)	0.6 (0.1–9)
**chloroquine**	12	295 (41–356)	1202 (626–2595)

## Summary and Conclusions

An indole carboxamide hit (**6a**) identified through an HTS program displayed moderate potency and a good physicochemical profile. The hit was profiled and optimized to deliver new indole carboxamide derivatives with 2- to 3-fold greater potencies and no hERG channel activity. The structural modification led to the replacement of methylene pyrrolidine with an *N*-methylpiperidine, removing the chiral center while enhancing potency. The most promising compound **6x** showed improvements in potency and metabolic stability, and weak hERG channel activity. Resistant strain screening suggested a mode of action like CQ and the susceptibility of indole carboxamide for the *Pf*CRT, like CQ. The *Pf*CRT efflux-mediated resistance was further confirmed by demonstrating a reversal effect on antiplasmodial IC_50_s in CQ-resistant strains, by addition of the known efflux pump inhibitor VP. Fluorometric evidence also suggested analogues interact with parasite digestive vacuole H^+^ homeostasis. Nevertheless, the pronounced cross-resistance observed with CQ-resistant strains, attributed to efflux pumps like *Pf*CRT, led to the deprioritization of the project. Despite this setback, this study highlights the antimalarial potential of indole carboxamides. Therefore, these indole carboxamides could serve as promising candidates for future medicinal chemistry if strategies can be identified with potential to overcome cross-resistance challenges.

## Experimental Protocols

### Chemistry

All the starting materials and solvents were purchased from commercial sources or synthesized according to the literature procedure. Organic solutions were dried over anhydrous sodium sulfate.

Unless noted, all reactions were performed under an atmosphere of argon with dry solvents and magnetic stirring. Dichloromethane (DCM) and triethylamine (Et_3_N) were distilled from CaH_2_. Tetrahydrofuran (THF) was distilled from sodium/benzophenone. Yields refer to homogeneous materials obtained after purification of reaction products by flash column chromatography using silica gel (200–400 mesh), liquid–liquid extraction, or recrystallization. Analytical thin-layer chromatography was performed on silica gel 60 and GF (5–40 μm thickness) plates, and visualization was accomplished using UV light, basic potassium permanganate staining, or ninhydrin solution followed by heating. ^1^H and proton-decoupled ^13^C NMR spectra were acquired in CD_3_OD or DMSO-*d*_6_ at 400 MHz (^1^H) and 75 or 126 MHz (^13^C) (Bruker Avance 400). Chemical shifts (δ) are reported in ppm using residual nondeuterated solvent as an internal standard CD_3_OD at 3.31 ppm, DMSO-*d*_6_ at 2.50 ppm, and TMS at 0.00 ppm for ^1^H NMR spectra and CD_3_OD at 49.0 ppm, DMSO-*d*_6_ at 39.52 ppm for ^13^C NMR spectra. Multiplicity data are reported as follows: s = singlet, d = doublet, t = triplet, q = quartet, br s = broad singlet, dd = doublet of doublets, dt = doublet of triplets, ddd = doublet of doublet of doublets, tt = triplet of triplets, m = multiplet, and br m = broad multiplet. The multiplicity is followed by the coupling constant(s) in Hz and integration. High resolution mass spectrometry (HRMS) was measured using electrospray ionization (ESI) (Waters 3 xevo Q-tof, Thermo LTQ-FT ultra, or Thermo Q exactive) or using electron ionization (EI) (GCT premier waters).

Purity of the final compound were determined with HPLC using C18 column (GEMINI (100 × 4.6 mm) C18,3u or Sunfire-C-18, (100 × 4.6), 5u or XSELECT (75 × 2.1 mm), 2.5 um), mobile phase of A (Acetonitrile) and B (0.05% HCCOH in water) with 0–100% gradient. The analysis is reported as HPLC Purity (percentage by area) and retention time (*t*_R_).

### General Procedures

#### General Procedure A1: Reductive Amination

To a stirred solution of Amine (**1**) and Aldehyde (**2**) (1 equiv) in THF was added anhydrous MgSO_4_ (4 equiv). The reaction mixture was cooled to 0 °C and acetic acid (2 equiv) was added. The mixture was stirred at room temperature for 2 h. The reaction mixture was cooled to 0 °C and NaBH­(OAc)_3_ (2 equiv) was added portion-wise. The reaction mixture was stirred at room temperature for 16 h. The reaction mixture was poured into a mixture of ice water and saturated aqueous NaHCO_3_. The reaction mixture was extracted with dichloromethane and the combined organic part was washed with water, brine, dried over anhydrous sodium sulfate and concentrated under reduced pressure to afford a crude product, which was used for the next step without further purification.

#### General Procedure B1: Amidation

To a solution of Acid (**4**) (1 equiv) in DMF were added HATU (1 equiv), and DIPEA (2 equiv) and was stirred at room temperature for 5 min. Then the amine (**3 or 11**) (1 equiv) was added, and the mixture was stirred at room temperature for 8–16 h. The reaction mixture was diluted with saturated NaHCO_3_ solution, and the aqueous layer was extracted with ethyl acetate. The combined organic layer was washed with water, brine and dried over anhydrous sodium sulfate, and concentrated. The crude product was purified by Combiflash chromatography to afford the amide **5**.

#### General Procedure C1: Boc Removal

TFA (2 equiv) was added to the carbamate (**5**) (1 equiv) solution in DCM in a dropwise manner at 0 °C and stirred at room temperature and stirred for 2 h. After completion, the reaction mixture was concentrated under reduced pressure to afford the crude product. The product was purified by column chromatography or preparative HPLC.

#### General Procedure D1: Tosylation

To a solution of 4-(hydroxymethyl)­pyrrolidin-2-one (1 equiv) **8** in dichloromethane were added trimethylamine (2 equiv) and DMAP (0.1 equiv). Then, p-toluenesulfonyl chloride (1 equiv) was added in small portions. The resulting solution was stirred at room temperature for 18 h. The reaction mixture was diluted with dichloromethane and washed with saturated aqueous NaHCO_3_ solution. The organic layer was dried over anhydrous sodium sulfate and concentrated to afford **(5-oxopyrrolidin-3-yl) methyl 4-methylbenzenesulfonate (9)**, which was used for the next step without further purification.

#### General Procedure E1: Nucleophilic Substitution

To a stirred solution of (5-oxopyrrolidin-3-yl)­methyl 4-methylbenzenesulfonate (**9**) (500 mg, 1.85 mmol) in DMF were added 3-pyridylmethanamine (**10**) (201 mg, 1.85 mmol) and NaI (cat). Then, the reaction mixture was heated at 90 °C for 16 h. The reaction mixture was diluted with dichloromethane and washed with saturated aqueous NaHCO_3_ solution. The organic layer was dried over an anhydrous sodium sulfate and concentrated to afford **4-[(3-pyridylmethylamino)­methyl]­pyrrolidin-2-one (11)** (480 mg, crude), which was used for the next step without further purification.

##### 5-Chloro-*N*-(pyridin-3-ylmethyl)-*N*-((tetrahydrofuran-3-yl)­methyl)-1*H*-indole-2-carboxamide (**5r**)

The title compound was prepared according to general procedure A1 and B1, starting with 5-chloro-1*H*-indole-2-carboxylic acid and 1-(pyridin-3-yl)-*N*-((tetrahydrofuran-3-yl)­methyl)­methanamine. The crude product was purified by Combiflash chromatography using 5% methanol in dichloromethane as eluent to get **5-chloro-** ***N*** **-(pyridin-3-ylmethyl)-** ***N*** **-((tetrahydrofuran-3-yl)­methyl)-1** ***H*** **-indole-2-carboxamide** (**5r**) (100 mg, 21%) as a white solid. ^1^H NMR (100 °C) (400 MHz, DMSO-*d*_6_) δ 11.68–11.35 (m, 1H), 8.55 (d, *J* = 2.3 Hz, 1H), 8.50 (d, *J* = 4.0 Hz, 1H), 7.69 (d, *J* = 8.0 Hz, 1H), 7.62 (d, *J* = 2.0 Hz, 1H), 7.46 (d, *J* = 8.0 Hz, 1H), 7.36 (dd, *J* = 7.9, 4.8 Hz, 1H), 7.21–7.14 (m, 1H), 6.76 (s, 1H), 4.97–4.81 (m, 2H), 3.75–3.55 (m, 5H), 3.40 (dd, *J* = 8.6, 5.8 Hz, 1H), 2.71–2.55 (m, 1H), 2.01–1.88 (m, 1H), 1.63–1.49 (m, 1H). ^13^C NMR (75 MHz, DMSO-*d*_6_) δ 163.87, 149.25, 149.01, 135.41, 134.77, 133.53, 131.82, 128.43, 124.71, 124.18, 123.97, 121.0, 114.11, 103.71, 70.74, 67.2, 38.16, 29.83; HRMS: [M + H]^+^ calcd for C_20_H_21_ClN_3_O_2_ = 370.1322; found = 370.1313. HPLC Purity: 99%, *t*_R_ = 6.43 min.

##### 5-Chloro-*N*-[(5-oxopyrrolidin-3-yl)­methyl]-*N*-(3-pyridylmethyl)-1*H*-indole-2-carboxamide (**5s**)

The title compound was prepared according to general procedures A1 and B1, starting with 4-[(3-pyridylmethylamino)­methyl]­pyrrolidin-2-one and 5-chloro-1*H*-indole-2-carboxylic acid. The crude product was purified by prep HPLC to afford **5-chloro-** ***N*** **-[(5-oxopyrrolidin-3-yl)­methyl]-** ***N*** **-(3-pyridylmethyl)-1** ***H*** **-indole-2-carboxamide (5s)** (3%) as an off-white solid. ^1^H NMR (400 MHz, DMSO-*d*_6_): δ 11.77 (br s, 1H), 8.67 (t, *J =* 5.8 Hz, 1H), 8.48–8.47 (m, 2H) 7.69 (d, *J* = 2.0 Hz, 1H), 7.63–7.61 (m, 1H), 7.42 (d, *J =* 8.6 Hz, 1H), 7.34 (dd, *J =* 7.8, 4.8 Hz, 1H), 7.18 (dd, *J* = 8.7, 2.0 Hz, 1H), 7.07 (br s, 1H), 4.41 (d, *J =* 15.2 Hz, 1H), 4.39 (d, *J =* 15.2 Hz, 1H), 3.40–3.32 (m, 3H), 3.11–3.07 (m, 1H), 2.67–2.22 (m, 3H) ppm; ^13^C NMR (75 MHz, DMSO-*d*_6_) δ 173.74, 161.51, 149.45, 149.01, 135.85, 135.30, 133.53, 133.07, 128.54, 124.65, 124.14, 123.84, 120.98, 114.35, 102.66, 50.28, 43.58, 42.63, 34.90, 31.65. HRMS: [M + H]^+^ calcd for C_20_H_20_ClN_4_O_2_ = 383.1275; found = 383.1267. HPLC Purity: 95%, *t*_R_ = 5.77 min.

##### 5-Chloro-*N*-(1-methylpiperidin-4-yl)-*N*-(pyridin-3-ylmethyl)-1*H*-indole-2-carboxamide (**5t**)

The title compound was prepared according to general procedure A1 and B1, starting from *tert*-butyl 4-aminopiperidine-1-carboxylate and nicotinaldehyde. The crude product was purified by flash chromatography (2% MeOH in dichloromethane) to afford **5-chloro-** ***N*** **-(1-methylpiperidin-4-yl)-** ***N*** **-(pyridin-3-ylmethyl)-1** ***H*** **-indole-2-carboxamide (5t)** (57%) as off-white solid. ^1^H NMR (500 MHz, CDCl_3_) δ 9.71 (s, 1H), 8.61 (s, 1H), 8.54 (s, 1H), 7.65 (d, *J* = 7.9 Hz, 1H), 7.56 (s, 1H), 7.31 (d, *J* = 8.7 Hz, 1H), 7.21 (dd, *J* = 8.7, 1.5 Hz, 1H), 6.59 (s, 1H), 4.89 (s, 2H), 4.64 (ddd, *J* = 15.7, 11.8, 3.9 Hz, 1H), 2.93 (d, *J* = 10.6 Hz, 2H), 2.29 (s, 3H), 2.09 (t, *J* = 11.4 Hz, 2H), 1.78 (m, 2H). ^13^C NMR (126 MHz, CDCl_3_) δ 163.68, 148.86, 148.60, 134.68, 134.08, 130.42, 128.72, 126.36, 125.39, 123.63, 121.42, 112.97, 104.29, 55.16, 46.06 (2C), 29.84 (2C). HRMS: [M + H]^+^ calcd for C_21_H_23_ClN_4_OH = 383.1633; found = 383.1628. NMR Purity ∼ 95%.

##### 5-Chloro-*N*-(pyridin-3-ylmethyl)-*N*-(pyrrolidin-3-ylmethyl)-1*H*-indole-2-carboxamide (**6a**)

The title compound was prepared according to General procedure A1, B1, and C1, starting with *tert*-butyl 3-{[1-(5-chloro-1*H*-indol-2-yl)-*N*-(pyridin-3-ylmethyl)­formamido]­methyl}­pyrrolidine-1-carboxylate. The crude product was purified by preparative HPLC to afford **5-chloro-** ***N*** **-(pyridin-3-ylmethyl)-** ***N*** **-(pyrrolidin-3-ylmethyl)-1** ***H*** **-indole-2-carboxamide (6a)** (46%) as a white solid. ^1^H NMR (400 MHz, DMSO-*d*_6_): δ 12.21 (br s, 1H), 8.55 (s, 1H), 8.50 (d, *J =* 3.9 Hz, 1H), 7.75–7.70 (m, 1H), 7.64 (s, 1H), 7.44–7.37 (m, 2H), 7.18 (dd, *J =* 8.7, 1.8 Hz, 1H), 6.81 (br s, 1H), 4.87 (s, 2H), 3.65–3.55 (m, 2H), 3.19–2.98 (m, 2H), 2.79–2.67 (m, 2H), 2.46–2.42 (m, 1H), 1.90–1.73 (m, 1H), 1.30–1.20 (m, 1H) ppm; ^13^C NMR (75 MHz, DMSO-*d*_6_) δ 163.83, 149.19, 148.95, 135.53, 134.89, 133.63, 132.22, 131.79, 128.43, 124.64, 124.15, 123.88, 120.95, 114.13, 104.16, 50.05, 45.75, 45.36, 38.07, 29.79, 29.26. HRMS: [M + H]^+^ calcd for C_21_H_24_ClN_4_O = 369.1482; found = 369.1475. Purity: 99%, *t*_R_ = 4.77 min.

##### 5-Chloro-*N*-(3-pyridylmethyl)-*N*-[[(3*R*)-pyrrolidin-3-yl]­methyl]-1*H*-indole-2-carboxamide­(6a­(*R*))

The title compound was prepared according to General procedure A1, B1, and C1, starting with *tert*-butyl (3*S*)-3-[[(5-chloro-1*H*-indole-2-carbonyl)-(3-pyridylmethyl)­amino]­methyl]­pyrrolidine-1-carboxylate. The crude product was purified by preparative HPLC to afford **5-chloro-** ***N*** **-(3-pyridylmethyl)-** ***N*** **-[[(3** ***R*** **)-pyrrolidin-3-yl]­methyl]-1** ***H*** **-indole-2-carboxamide­(6a­(*R*))** (64%) as a gummy liquid. ^1^H NMR (400 MHz, DMSO-*d*_6_): δ 8.55 (s, 1H), 8.48 (d, *J =* 4.4 Hz, 1H), 7.69 (s, 1H), 7.64 (s, 1H), 7.44–7.38 (m, 2H), 7.18 (dd, *J =* 8.8, 1.6 Hz, 1H), 6.78 (s, 1H), 4.87 (s, 2H), 3.62–3.52 (m, 2H), 2.88–2.76 (m, 3H), 2.53–2.50 (m, 1H), 2.44–2.42 (m, 1H), 1.83–1.75 (m, 1H), 1.39–1.31 (m, 1H) ppm; ^13^C NMR (125 MHz, DMSO-*d*_6_) δ 163.88, 149.15, 148.98, 134.90, 133.57, 132.05, 128.45, 124.67, 124.17, 123.91, 120.97, 114.16, 103.85, 49.65, 45.56, 37.96, 29.54; HRMS: [M + H]^+^ calcd for C_20_H_22_ClN_4_O = 369.1482; found = 369.1480. HPLC Purity: 97%, *t*_R_ = 4.74 min.

##### 5-Chloro-*N*-(3-pyridylmethyl)-*N*-[[(3*S*)-pyrrolidin-3-yl]­methyl]-1*H*-indole-2-carboxamide­(6a­(S))

The title compound was prepared according to General procedure A1, B1, and C1, starting with *tert*-butyl (3*R*)-3-[[(5-chloro-1*H*-indole-2-carbonyl)-(3-pyridylmethyl)­amino]­methyl]­pyrrolidine-1-carboxylate. The crude product was purified by preparative HPLC to afford **5-chloro-** ***N*** **-(3-pyridylmethyl)-** ***N*** **-[[(3** ***S*** **)-pyrrolidin-3-yl]­methyl]-1** ***H*** **-indole-2-carboxamide­(6a­(S))** (51%) as a gummy liquid. ^1^H NMR (400 MHz, DMSO-*d*_6_): δ 11.88 (s, 1H), 8.54 (s, 1H), 8.49 (d, *J =* 4.4 Hz, 1H), 7.70 (s, 1H), 7.64 (s, 1H), 7.44–7.38 (m, 2H), 7.18 (dd, *J =* 8.8, 1.6 Hz, 1H), 6.79 (s, 1H), 4.88 (s, 2H), 3.62–3.52 (m, 2H), 2.88–2.76 (m, 3H), 2.53–2.50 (m, 1H), 2.44–2.42 (m, 1H), 1.83–1.75 (m, 1H), 1.39–1.31 (m, 1H) ppm; ^13^C NMR (125 MHz, DMSO-*d*_6_) δ 163.87, 149.25, 148.98, 135.42, 134.90, 133.69, 132.14, 128.45, 124.67, 124.17, 123.91, 120.97, 114.15, 104.13, 49.80, 45.60, 37.98, 29.61; HRMS: [M + H]^+^ calcd for C_21_H_24_ClN_4_O = 369.1482; found = 369.1480. HPLC Purity: 98%, *t*_R_ = 4.74 min.

##### *N*-(3-Pyridylmethyl)-*N*-(pyrrolidin-3-ylmethyl)-1*H*-indole-2-carboxamide (**6b**)

The title compound was prepared according to General procedure A1, B1, and C1, starting with 3-[[1*H*-indole-2-carbonyl­(3-pyridylmethyl)­amino]­methyl]­pyrrolidine-1-carboxylate. The crude product was purified by Combiflash chromatography using 10% methanol in dichloromethane as eluent to get ***N*** **-(3-pyridylmethyl)-** ***N*** **-(pyrrolidin-3-ylmethyl)-1** ***H*** **-indole-2-carboxamide** (**6b**) (33 mg, 22%) as an off-white solid. ^1^H NMR (100 °C) (400 MHz, DMSO-*d*_6_) δ 12.23–10.70 (m, 1H), 8.56 (d, *J* = 1.8 Hz, 1H), 8.49 (dd, *J* = 4.7, 1.7 Hz, 1H), 7.75–7.67 (m, 1H), 7.58 (d, *J* = 8.0 Hz, 1H), 7.46 (dd, *J* = 8.3, 1.1 Hz, 1H), 7.36 (dd, *J* = 7.9, 4.7 Hz, 1H), 7.23–7.14 (m, 1H), 7.08–6.99 (m, 1H), 6.80 (s, 1H), 4.89 (s, 2H), 3.68–3.52 (m, 2H), 2.89–2.69 (m, 3H), 2.59–2.52 (m, 1H), 2.49–2.38 (m, 1H), 1.85–1.71 (m, 1H), 1.41–1.25 (m, 1H). ^13^C NMR (125 MHz, DMSO-*d*_6_) δ 164.17, 149.26, 148.92, 136.44, 135.43, 133.81, 130.53, 127.39, 124.14, 123.82, 121.92, 120.20, 112.52, 104.58, 50.26, 45.94, 45.34, 38.14, 29.90; HRMS: [M + H]^+^ calcd for C_20_H_23_ClN_4_O = 335.1872; found = 335.1834. HPLC Purity: 98%, *t*_R_ = 4.30 min.

##### 7-Chloro-*N*-(3-pyridylmethyl)-*N*-(pyrrolidin-3-ylmethyl)-1*H*-indole-2-carboxamide (**6c**)

The title compound was prepared according to General procedure A1, B1, and C1, starting with *tert*-butyl 3-[[(7-chloro-1*H*-indole-2-carbonyl)-(3-pyridylmethyl)­amino]­methyl]­pyrrolidine-1-carboxylate (**57**). The crude product was purified by preparative HPLC to afford **7-chloro-** ***N*** **-(3-pyridylmethyl)-** ***N*** **-(pyrrolidin-3-ylmethyl)-1** ***H*** **-indole-2-carboxamide (6c)** (38%) as an off-white Solid. ^1^H NMR (400 MHz, DMSO-*d*_6_): δ 8.60 (br s, 1H), 8.50–8.49 (m, 1H), 7.76 (br s, 1H), 7.55 (d, *J =* 7.9 Hz, 1H) 7.39 (dd, *J =* 4.8, 2.9 Hz, 1H), 7.24 (d, *J =* 7.5 Hz, 1H), 7.02 (t, *J =* 7.7 Hz, 1H), 6.95–6.90 (br s, 1H), 4.92–4.63 (m, 2H), 3.57–2.58 (m, 7H), 1.83–1.75 (m, 1H), 1.38–1.30 (m, 1H) ppm; ^13^C NMR (75 MHz, DMSO-*d*_6_) δ 164.46, 149.59, 148.95, 135.91, 133.60, 129.23, 124.09, 122.79, 120.88, 120.55, 117.00, 106.62, 50.46, 48.54, 46.08, 44.60, 37.01, 28.87; HRMS: [M + H]^+^ calcd for C_21_H_24_ClN_4_O = 369.1482; found = 369.1476. HPLC Purity: 99%, *t*_R_ = 4.63 min.

##### 5-Methoxy-*N*-(3-pyridylmethyl)-*N*-(pyrrolidin-3-ylmethyl)-1*H*-indole-2-carboxamide (**6d**)

The title compound was prepared according to General procedure A1, B1, and C1, starting with *tert*-butyl 3-[[(5-methoxy-1*H*-indole-2-carbonyl)-(3-pyridylmethyl)­amino]­methyl]­pyrrolidine-1-carboxylate. The crude product was purified by preparative HPLC to afford **5-methoxy-** ***N*** **-(3-pyridylmethyl)-** ***N*** **-(pyrrolidin-3-ylmethyl)-1** ***H*** **-indole-2-carboxamide (6d)** (16%) as a sticky solid. ^1^H NMR (400 MHz, DMSO-*d*_6_): δ 11.80–11.70 (br s, 1H), 8.55 (s, 1H), 8.50 (d, *J =* 3.7 Hz, 1H), 7.70 (d, *J =* 7.5 Hz, 1H), 7.44–7.38 (m, 1H), 7.31 (d, *J =* 8.9 Hz, 1H), 7.04 (s, 1H), 6.83 (dd, *J =* 8.9, 2.4 Hz, 1H), 6.65–6.75 (br s, 1H), 4.88 (br s, 2H), 3.73 (s, 3H), 3.60–3.50 (m, 2H), 3.21–3.10 (m, 1H), 2.83–2.62 (m, 3H), 2.48–2.37 (m, 2H), 1.80–1.69 (m, 1H), 1.36–1.24 (m, 1H) ppm; ^13^C NMR (75 MHz, DMSO-*d*_6_) δ 164.06, 154.19, 149.21, 148.89, 135.40, 133.83, 131.69, 130.77, 127.69, 124.13, 115.07, 113.36, 104.39, 102.35, 55.66, 50.37, 46.00, 41.06, 38.20, 29.95. HRMS: [M + H]^+^ calcd for C_21_H_24_ClN_4_O = 365.1978; found = 365.1968. HPLC Purity: 99%, *t*_R_ = 4.29 min.

##### 5-Fluoro-*N*-(3-pyridylmethyl)-*N*-(pyrrolidin-3-ylmethyl)-1*H*-indole-2-carboxamide (**6e**)

The title compound was prepared according to General procedure A1, B1, and C1, starting with *tert*-butyl 3-[[(5-fluoro-1*H*-indole-2-carbonyl)-(3-pyridylmethyl)­amino]­methyl]­pyrrolidine-1-carboxylate. The crude product was purified by preparative HPLC to afford **5-fluoro-** ***N*** **-(3-pyridylmethyl)-** ***N*** **-(pyrrolidin-3-ylmethyl)-1** ***H*** **-indole-2-carboxamide (6e)** (46%) as a gummy Liquid. ^1^H NMR (400 MHz, DMSO-*d*_6_): δ 12.30–12.00 (br s, 1H), 8.55 (s, 1H), 8.50 (d, *J =* 4.2 Hz, 1H), 7.71 (d, *J =* 6.1 Hz, 1H), 7.43–7.38 (m, 2H), 7.35 (d, *J =* 9.2 Hz, 1H), 7.03 (td, *J =* 9.2, 2.4 Hz, 1H), 6.90–6.70 (br s, 1H), 4.87 (br s, 2H), 3.61–3.53 (m, 2H), 2.78–2.42 (m, 5H), 1.77–1.72 (m, 2H), 1.29 (m, 1H) ppm; ^13^C NMR (75 MHz, DMSO-*d*_6_) δ 163.88, 156.02, 149.29, 148.93, 135.52, 133.71, 133.21, 132.39, 127.52, 127.38, 124.14, 113.79, 113.66, 112.65, 112.30, 106.19, 105.88, 104.54, 50.35, 45.92, 38.13, 29.90. HRMS: [M + H]^+^ calcd for C_21_H_24_ClN_4_O = 353.1778; found = 353.1768. HPLC Purity: 97%, *t*_R_ = 4.44 min.

##### *N*-(3-Pyridylmethyl)-*N*-(pyrrolidin-3-ylmethyl)-5-(trifluoromethyl)-1*H*-indole-2-carboxamide (**6f**)

The title compound was prepared according to General procedure A1, B1, and C1, starting with *tert*-butyl 3-[[3-pyridylmethyl-[5-(trifluoromethyl)-1*H*-indole-2-carbonyl]­amino]­methyl]­pyrrolidine-1-carboxylate. The crude product was purified by preparative HPLC to afford ***N*** **-(3-pyridylmethyl)-** ***N*** **-(pyrrolidin-3-ylmethyl)-5-(trifluoromethyl)-1** ***H*** **-indole-2-carboxamide (6f)** (27%) as an off-white Solid. ^1^H NMR (400 MHz, DMSO-*d*_6_): δ 8.57 (s, 1H), 8.50 (d, *J =* 4.4 Hz, 1H), 8.02 (s, 1H), 7.72 (s, br s, 1H), 7.60 (d, *J =* 8.6 Hz, 1H), 7.47 (d, *J =* 8.6 Hz, 1H), 7.41–7.38 (dd, *J =* 4.8, 2.9 Hz, 1H), 7.05–6.95 (br s, 1H), 4.88 (br s, 2H), 3.54–2.55 (m, 7H), 2.57 (s, 3H), 1.82–1.71 (m, 1H), 1.28–1.22 (m, 1H) ppm; ^13^C NMR (125 MHz, DMSO-*d*_6_) δ 163.76, 148.99, 137.95, 133.62, 126.92, 126.69, 124.76, 124.16, 120.88, 120.04, 119.92, 113.41, 72.75, 60.73, 45.78, 42.58, 40.92, 38.11, 30.29; HRMS: [M + H]^+^ calcd for C_21_H_22_F_3_N_4_O = 403.1746; found = 403.1742. HPLC Purity: 99%, *t*_R_ = 5.04 min.

##### 5-Chloro-3-methyl-*N*-(3-pyridylmethyl)-*N*-(pyrrolidin-3-ylmethyl)-1*H*-indole-2-carboxamide (**6g**)

The title compound was prepared according to General procedure A1, B1, and C1, starting with *tert*-butyl 3-[[(5-chloro-3-methyl-1*H*-indole-2-carbonyl)-(3-pyridylmethyl)­amino]­methyl]­pyrrolidine-1-carboxylate. The crude product was purified by preparative HPLC to afford **5-chloro-3-methyl-** ***N*** **-(3-pyridylmethyl)-** ***N*** **-(pyrrolidin-3-ylmethyl)-1** ***H*** **-indole-2-carboxamide (6g)** (36%) as off-white solid. ^1^H NMR (400 MHz, DMSO-*d*_6_): δ 11.62 (m, 1H), 8.72–8.48 (m, 2H), 7.60–7.55 (m, 1H), 7.59 (s, 1H), 7.37–7.34 (m, 2H), 7.16 (d, *J =* 7.4 Hz, 1H), 4.70–4.66 (m, 2H), 3.58–3.45 (m, 2H), 3.70–2.24 (m, 5H), 2.23 (s, 3H), 1.85–1.70 (m, 1H), 1.23–1.11 (m, 1H) ppm; ^13^C NMR (75 MHz, DMSO-*d*_6_) δ 165.30, 149.41, 149.06, 135.81, 134.58, 133.50, 128.97, 124.08 (2C), 104.03, 123.18, 119.01, 113.71, 61.71, 49.58, 45.70, 37.40, 9.45; HRMS: [M + H]^+^ calcd for C_21_H_24_ClN_4_O = 383.1639; found = 383.1630. HPLC Purity: 99%, *t*_R_ = 3.58 min.

##### 5-Chloro-*N*-(3-pyridylmethyl)-*N*-(pyrrolidin-3-ylmethyl)-1*H*-indole-3-carboxamide (**6h**)

The title compound was prepared according to General procedure A1, B1, and C1, starting with *tert*-butyl 3-[[(5-chloro-1*H*-indole-3-carbonyl)-(3-pyridylmethyl)­amino]­methyl]­pyrrolidine-1-carboxylate. The crude product was purified by preparative HPLC to afford **5-chloro-** ***N*** **-(3-pyridylmethyl)-** ***N*** **-(pyrrolidin-3-ylmethyl)-1** ***H*** **-indole-3-carboxamide (6h)** (76%) as an off-white solid. ^1^H NMR (400 MHz, DMSO-*d*_6_): δ 11.74 (br s, 1H), 8.51 (s, 1H), 8.48 (d, *J =* 3.8 Hz, 1H), 7.82 (s, 1H), 7.75 (d, *J =* 1.8 Hz, 1H), 7.70 (d, *J =* 7.5 Hz, 1H), 7.47 (d, *J =* 8.6 Hz, 1H), 7.39 (dd, *J =* 7.8, 4.8 Hz, 1H), 7.18 (dd, *J =* 8.6, 2.0 Hz, 1H), 4.84–4.81 (m, 2H), 3.53–3.47 (m, 3H), 3.17–3.15 (m, 1H), 2.75–2.64 (m, 2H), 2.41 (d, *J =* 7.4 Hz, 1H), 1.70–1.68 (m, 1H), 1.24 (m, 1H) ppm; ^13^C NMR (125 MHz, DMSO-*d*_6_) δ 166.93, 149.16, 148.81, 135.41, 134.62, 134.33, 129.20, 128.46, 125.39, 124.12, 122.52, 120.05, 113.99, 109.81, 50.41, 46.12, 45.29, 38.17, 37.68, 30.01. HRMS: [M + H]^+^ calcd for C_20_H_22_ClN_4_O = 369.1482; found = 369.1479. HPLC Purity: 96%, *t*_R_ = 2.54 min.

##### 5-Chloro-*N*-(3-pyridylmethyl)-*N*-(pyrrolidin-3-ylmethyl)-1*H*-benzimidazole-2-carboxamide (**6i**)

The title compound was prepared according to General procedure A1, B1, and C1, starting with *tert*-butyl 3-[[(5-chloro-1*H*-benzimidazole-2-carbonyl)-(3-pyridylmethyl)­amino]­methyl]­pyrrolidine-1-carboxylate. The crude product was purified by preparative HPLC to afford **5-chloro-** ***N*** **-(3-pyridylmethyl)-** ***N*** **-(pyrrolidin-3-ylmethyl)-1** ***H*** **-benzimidazole-2-carboxamide­(6i)** (40%) as an off-white solid. ^1^H NMR (400 MHz, DMSO-*d*_6_): δ 8.61 (d, *J =* 1.2 Hz, 1H), 8.55–8.48 (m, 1H), 8.04 (m, 1H), 7.77–7.73 (m, 1H), 7.66 (d, *J =* 1.6 Hz, 1H), 7.62 (dd, *J =* 8.8, 4.0 Hz, 1H), 7.40–7.34 (m, 1H), 7.30–7.25 (m, 1H), 5.55–5.50 (q, *J* = 16 Hz, 1H) 4.86–4.71 (q, *J* = 15.0 Hz, 1H), 4.22–4.05 (m1H), 3.98–3.82 (m, 1H), 2.88–2.81 (m, 3H), 2.77–2.73 (m, 1H), 2.54 (m, 1H), 1.86 (m, 1H), 1.38, (m, 1H) ppm; ^13^C NMR (75 MHz, DMSO-*d*_6_) δ 161.58, 160.54, 149.64, 149.42, 149.15, 148.94, 147.67, 139.47, 137.43, 135.91, 135.74, 133.77, 133.54, 128.08, 127.51, 124.14, 124.08, 123.58, 118.21, 118.10, 116.34, 116.27, 50.62, 50.39, 49.55, 49.24, 47.12, 45.95, 45.36, 38.07, 37.69, 29.94, 29.22. HRMS: [M + H]^+^ calcd for C_21_H_24_ClN_4_O = 370.1435; found = 370.1426. HPLC Purity: 99%, *t*_R_ = 4.49 min.

##### 3-Chloro-*N*-(pyridin-3-ylmethyl)-*N*-(pyrrolidin-3-ylmethyl)­benzamide (**6j**)

HRMS: [M + H]^+^ calcd for C_18_H_21_ClN_3_O = 330.1373; found = 330.136, LCMS Purity: 95%, *t*_R_ = 1.38 min.

#### The Following Compound Was Purchased (Recorded only LC-MS and HRMS for Registration)

##### *N*-Benzyl-5-chloro-*N*-(pyrrolidin-3-ylmethyl)-1*H*-indole-2-carboxamide (**6k**)

The title compound was prepared according to General procedure A1, B1, and C1, starting with *tert*-butyl 3-[[benzyl-(5-chloro-1*H*-indole-2-carbonyl)­amino]­methyl]­pyrrolidine-1-carboxylate and TFA. The crude which was purified by column chromatography (silica 100–200) using 10% MeOH in DCM as eluent to afford ***N*** **-benzyl-5-chloro-** ***N*** **-(pyrrolidin-3-ylmethyl)-1** ***H*** **-indole-2-carboxamide** (**6k**) (68 mg, 34%) as a brown sticky sold. ^1^H NMR (100 °C) (400 MHz, DMSO-*d*_6_) δ 7.60 (d, *J* = 1.9 Hz, 1H), 7.46 (d, *J* = 8.7 Hz, 1H), 7.41–7.33 (m, 2H), 7.34–7.24 (m, 3H), 7.17 (dd, *J* = 8.7, 2.1 Hz, 1H), 6.74 (s, 1H), 4.88 (s, 2H), 3.54 (dd, *J* = 7.4, 2.4 Hz, 2H), 2.94–2.76 (m, 3H), 2.63–2.51 (m, 1H), 2.49–2.41 (m, 1H), 1.86–1.74 (m, 1H), 1.43–1.33 (m, 1H). ^13^C NMR (75 MHz, DMSO-*d*_6_) δ 163.78, 137.81, 134.82, 134.75, 132.05, 129.16, 128.40, 127.72, 127.38, 124.63, 123.86, 120.92, 114.10, 103.58, 49.94, 45.68, 37.87, 37.79, 29.65; HRMS: [M + H]^+^ calcd for C_21_H_24_ClN_4_O = 368.1530; found = 368.1523. HPLC Purity: 90%, *t*_R_ = 5.89 min.

##### 5-Chloro-*N*-(4-pyridylmethyl)-*N*-(pyrrolidin-3-ylmethyl)-1*H*-indole-2-carboxamide (**6l**)

The title compound was prepared according to General procedure A1, B1, and C1, starting with 3-[[(5-chloro-1*H*-indole-2-carbonyl)-(4-pyridylmethyl)­amino]­methyl]­pyrrolidine-1-carboxylate and TFA. The volatiles were removed, and the crude was purified by prep-HPLC to get the desired compound as TFA salt (**6l**) (28 mg, 17%) as a light-yellow sticky gum. ^1^H NMR (100 °C) (400 MHz, DMSO-*d*_6_) δ 11.64–11.47 (m, 1H), 8.58 (d, *J* = 5.8 Hz, 3H), 7.61 (d, *J* = 2.0 Hz, 1H), 7.47 (d, *J* = 8.8 Hz, 1H), 7.31 (d, *J* = 5.2 Hz, 2H), 7.19 (dd, *J* = 8.7, 2.1 Hz, 1H), 6.71 (s, 1H), 4.94 (d, *J* = 2.6 Hz, 2H), 3.75–3.62 (m, 2H), 3.36–3.07 (m, 3H), 2.99–2.84 (m, 1H), 2.78–2.62 (m, 1H), 2.15–1.99 (m, 1H), 1.77–1.60 (m, 1H). ^13^C NMR (125 MHz, DMSO-*d*_6_) δ 163.97, 158.87, 158.60, 147.70, 134.86, 131.18, 128.42, 124.81, 124.27, 123.50, 123.38, 121.09, 114.23, 104.21, 48.35, 44.90, 28.34; HRMS: [M + H]^+^ calcd for C_20_H_22_ClN_4_O = 369.1482; found = 369.1477. HPLC Purity: 99%, *t*_R_ = 4.93 min.

##### 5-Chloro-*N*-(2-pyridylmethyl)-*N*-(pyrrolidin-3-ylmethyl)-1*H*-indole-2-carboxamide (**6m**)

The title compound was prepared according to General procedure A1, B1, and C1, starting with *tert*-butyl 3-[[(5-chloro-1*H*-indole-2-carbonyl)-(2-pyridylmethyl)­amino]­methyl]­pyrrolidine-1-carboxylate and TFA. The crude product was purified by column chromatography using 10% methanol in dichloromethane as eluent to afford **5-chloro-** ***N*** **-(2-pyridylmethyl)-** ***N*** **-(pyrrolidin-3-ylmethyl)-1** ***H*** **-indole-2-carboxamide** (**6m)** (20 mg, 13%) as a white solid. ^1^H NMR (400 MHz, DMSO-*d*_6_) δ 12.84–11.31 (m, 1H), 8.57 (s, 1H), 7.80 (t, *J* = 7.7 Hz, 1H), 7.71–7.53 (m, 1H), 7.42 (d, *J* = 8.7 Hz, 1H), 7.38–7.27 (m, 2H), 7.17 (dd, *J* = 8.7, 2.1 Hz, 1H), 7.08–6.48 (m, 1H), 5.31–4.61 (m, 2H), 3.97–3.40 (m, 2H), 2.94–2.49 (m, 5H), 1.82–1.68 (m, 1H), 1.41–1.25 (m, 1H); ^13^C NMR (75 MHz, DMSO-*d*_6_) δ 163.82, 157.45, 149.84, 137.52, 134.80, 132.38, 128.39, 124.58, 123.77, 122.97, 122.11, 120.90, 114.09, 103.86, 54.19, 50.50, 46.05, 45.36, 38.10, 30.05; HRMS: [M + H]^+^ calcd for C_21_H_24_ClN_4_O = 369.1482; found = 369.1474. HPLC Purity: 98%, *t*_R_ = 5.64 min.

##### 5-Chloro-*N*-(pyrazin-2-ylmethyl)-*N*-(pyrrolidin-3-ylmethyl)-1*H*-indole-2-carboxamide­(**6n**)

The title compound was prepared according to General procedure A1, B1, and C1, starting with *tert*-butyl 3-[[(5-chloro-1*H*-indole-2-carbonyl)-(pyrazin-2-ylmethyl)­amino]­methyl]­pyrrolidine-1-carboxylate. The crude product was purified by preparative HPLC to afford **5-chloro-** ***N*** **-(pyrazin-2-ylmethyl)-** ***N*** **-(pyrrolidin-3-ylmethyl)-1** ***H*** **-indole-2-carboxamide (6n**) (12%) as an off-white solid. ^1^H NMR (400 MHz, DMSO-*d*_6_): δ 11.9–12.02 (br s, 1H), 8.66 (s, 1H), 8.62 (s, 1H), 8.56 (s, 1H), 7.63 (br s, 1H), 7.42 (d, *J* = 8.5 Hz, 1H), 7.18 (dd, *J* = 8.6, 1.6 Hz, 1H), 6.83 (br s, 1H), 4.90–4.51 (br s, 2H), 3.81–2.45 (m, 7H), 1.75–1.77 (m, 1H), 1.31–1.33 (m, 1H) ppm; ^13^C NMR (75 MHz, DMSO-*d*_6_) δ 163.88, 153.51, 144.68, 144.30, 144.01, 134.88, 132.22, 128.40, 124.59, 123.83, 120.94, 114.11, 104.76, 50.27, 45.96, 38.26, 29.99. HRMS: [M + H]^+^ calcd for C_21_H_24_ClN_4_O = 370.1435; found = 370.1426. HPLC Purity: 97%, *t*_R_ = 5.18 min.

##### 5-Chloro-*N*-(pyrrolidin-3-ylmethyl)-*N*-[[6-(trifluoromethyl)-3-pyridyl]­methyl]-1*H*-indole-2-carboxamide (**6o**)

The title compound was prepared according to General procedure A1, B1, and C1, starting with *tert*-butyl 3-[[(5-chloro-1*H*-indole-2-carbonyl)-[[6-(trifluoromethyl)-3-pyridyl]­methyl]­amino]­methyl] pyrrolidine-1-carboxylate. The crude product was purified by preparative HPLC to afford **5-chloro-** ***N*** **-(pyrrolidin-3-ylmethyl)-** ***N*** **-[[6-(trifluoromethyl)-3-pyridyl]­methyl]-1** ***H*** **-indole-2-carboxamide (6o)** (39%) as an off-white solid. ^1^H NMR (400 MHz, DMSO-*d*_6_): δ 12.1 (br s, 1H), 8.74 (s, 1H), 8.00–7.98 (m, 1H), 7.90 (d, *J =* 8.0 Hz, 1H), 7.65 (s, 1H), 7.43 (d, *J =* 8.7 Hz, 1H), 7.18 (dd, *J =* 9.0, 1.9 Hz, 1H), 6.85 (br s, 1H), 4.94 (br s, 2H), 3.59–3.50 (m, 2H), 2.79–2.62 (m, 3H), 2.50–2.42 (m, 2H), 1.82–1.70 (m, 1H), 1.29–1.23 (m, 1H) ppm; ^13^C NMR (75 MHz, DMSO-*d*_6_) δ 163.88, 149.77, 145.97, 138.08, 137.39, 134.94, 131.98, 128.43, 124.66, 123.96, 121.13, 120.99, 120.37, 114.15, 104.39, 79.63, 50.16, 45.88, 41.17, 38.19, 29.82. HRMS: [M + H]^+^ calcd for C_21_H_24_ClN_4_O = 437.1356; found = 437.1345. HPLC Purity: 99%, *t*_R_ = 5.77 min.

##### 5-Chloro-*N*-[(6-methylsulfonyl-3-pyridyl)­methyl]-*N*-(pyrrolidin-3-ylmethyl)-1*H*-indole-2-carboxamide (**6p**)

The title compound was prepared according to General procedure A1, B1, and C1, starting with *tert*-butyl 3-[[(5-chloro-1*H*-indole-2-carbonyl)-[(6-methylsulfonyl-3-pyridyl)­methyl]­amino]­methyl] pyrrolidine-1-carboxylate. The crude product was purified by preparative HPLC to afford **5-chloro-** ***N*** **-[(6-methylsulfonyl-3-pyridyl)­methyl]-** ***N*** **-(pyrrolidin-3-ylmethyl)-1** ***H*** **-indole-2-carboxamide (6p)** (30%) as an off-white solid. ^1^H NMR (400 MHz, DMSO-*d*_6_): δ 12.3 (m, 1H), 8.75 (s, 1H), 8.01 (s, 2H), 7.65 (s, 1H), 7.44 (d, *J =* 8.0 Hz, 1H), 7.19 (dd, *J =* 8.0, 1.96 Hz, 1H), 6.85 (br s, 1H), 4.95 (br s, 2H), 3.65–3.55 (br s, 2H), 3.27 (s, 3H), 2.72–2.43 (m, 5H), 1.79–1.71 (m, 1H), 1.29–1.27 (m, 1H) ppm; ^13^C NMR (75 MHz, DMSO-*d*_6_) δ 163.88, 156.95, 149.68, 138.68, 137.84, 131.95, 128.43, 124.67, 123.97, 121.17, 121.00, 114.17, 104.53, 50.23, 45.93, 40.36, 38.24, 29.84. HRMS: [M + H]^+^ calcd for C_21_H_24_ClN_4_O_3_S = 447.1258; found = 447.1250. HPLC Purity: 98%, *t*_R_ = 5.27 min.

##### 5-Chloro-*N*-[2-(methylamino)­ethyl]-*N*-(pyrrolidin-3-ylmethyl)-1*H*-indole-2-carboxamide (**6q**)

The title compound was prepared according to General procedure A1, B1, and C1, starting with *tert*-butyl 3-[[2-[tert-butoxycarbonyl­(methyl)­amino]­ethyl-(5-chloro-1*H*-indole-2-carbonyl)­amino]­methyl] pyrrolidine-1-carboxylate. The crude product was purified by preparative HPLC to afford **5-chloro-** ***N*** **-[2-(methylamino)­ethyl]-** ***N*** **-(pyrrolidin-3-ylmethyl)-1** ***H*** **-indole-2-carboxamide (6q)** (5%) as an off-white solid. ^1^H NMR (400 MHz, DMSO-*d*_6_): δ 11.80 (br s, 1H), 7.66 (s, 1H), 7.44 (d, *J =* 8.8 Hz, 1H), 7.17 (dd, *J =* 8.4, 1.6 Hz, 1H), 6.86 (s, 1H), 3.63–3.56 (m, 2H), 3.18 (s, 3H), 2.85–2.90 (m, 1H), 2.78–2.83 (m, 3H), 2.76–2.66 (m, 2H), 2.55–2.52 (m, 1H), 2.50–2.44 (m, 1H), 2.13–2.06 (m, 1H), 1.80–1.71 (m, 1H), 1.34–1.27 (m, 1H) ppm; HRMS: [M + H]^+^ calcd for C_17_H_24_ClN_4_O = 335.1639; found = 335.1629. HPLC Purity: 99%, *t*_R_ = 4.23 min.

##### 5-Chloro-*N*-[2-(methylamino)­ethyl]-*N*-(3-pyridylmethyl)-1*H*-indole-2-carboxamide (**6u**)

The title compound was prepared according to General procedure A1, B1, and C1, starting with *tert*-butyl *N*-[2-[(5-chloro-1*H*-indole-2-carbonyl)-(3-pyridylmethyl)­amino]­ethyl]-*N*-methyl-carbamate. The crude product was purified by preparative HPLC to afford **5-chloro-** ***N*** **-[2-(methylamino)­ethyl]-** ***N*** **-(3-pyridylmethyl)-1** ***H*** **-indole-2-carboxamide (6u)** (13%) as an off-white solid. Acetate salt ^1^H NMR (400 MHz, DMSO-*d*_6_): δ 11.80 (s, 1H), 8.52 (d, *J =* 1.6 Hz, 1H), 8.43 (dd, *J =* 4.6, 1.4 Hz, 1H), 7.73 (d, *J =* 7.6 Hz, 1H), 7.65 (d, *J =* 1.8 Hz, 1H), 7.41 (d, *J =* 8.8 Hz, 1H), 7.34–7.30 (m, 1H), 7.18 (dd, *J =* 8.7, 2.0 Hz, 1H), 6.85 (s, 1H), 3.74 (s, 3H), 3.70–3.55 (m, 4H), 2.80–2.70 (m, 2H), 1.89 (s, 3H) ppm; ^13^C NMR (75 MHz, DMSO-*d*_6_) δ 172.68, 149.83, 148.37, 136.54, 136.11, 134.59, 132.45, 128.55, 124.52, 123.80 (2C), 120.88, 114.11, 104.66, 50.52, 46.64, 33.08, 21.70; HRMS: [M + H]^+^ calcd for C_18_H_20_ClN_4_O = 343.1326; found = 343.1318. HPLC Purity: 97%, *t*_R_ = 4.99 min.

##### 5-Chloro-*N*-(3-pyridylmethyl)-*N*-pyrrolidin-3-yl-1*H*-indole-2-carboxamide (**6v**)

The title compound was prepared according to General procedure A1, B1, and C1, starting with *tert*-butyl 3-[(5-chloro-1*H*-indole-2-carbonyl)-(3-pyridylmethyl)­amino]­pyrrolidine-1-carboxylate. The crude product was purified by preparative HPLC to afford **5-chloro-** ***N*** **-(3-pyridylmethyl)-** ***N*** **-pyrrolidin-3-yl-1** ***H*** **-indole-2-carboxamide (6v)** (45%) as an off-white solid. ^1^H NMR (400 MHz, DMSO-*d*_6_): δ 11.85 (br s, 1H), 8.53 (s, 1H), 8.46 (d, *J =* 4.3 Hz, 1H), 7.68–7.65 (m, 2H), 7.42 (d, *J =* 8.7 Hz, 1H), 7.36 (dd, *J =* 7.68, 4.76 Hz, 1H), 7.19–7.17 (m, 1H), 6.90 (br s, 1H), 4.92–4.80 (m,3H), 3.0–2.66 (m, 4H), 2.07–2.02 (m, 1H) 1.80–1.72 (m, 1H) ppm; ^13^C NMR (75 MHz, DMSO-*d*_6_) δ 163.89, 148.45 (2C), 135.06, 134.84, 134.53, 132.25, 128.39, 124.68, 123.88 (2C), 120.93, 114.09, 103.41, 59.08, 50.50, 46.34, 30.77. HRMS: [M + H]^+^ calcd for C_19_H_20_ClN_4_O = 355.1326; found = 355.1317. HPLC Purity: 98%, *t*_R_ = 4.83 min.

##### 5-Chloro-*N*-(4-piperidylmethyl)-*N*-(3-pyridylmethyl)-1*H*-indole-2-carboxamide (**6w**)

The title compound was prepared according to General procedure A1, B1, and C1, starting with *tert*-butyl 4-[[(5-chloro-1*H*-indole-2-carbonyl)-(3-pyridylmethyl)­amino]­methyl]­piperidine-1-carboxylate. The crude product was purified by preparative HPLC to afford **5-chloro-** ***N*** **-(4-piperidylmethyl)-** ***N*** **-(3-pyridylmethyl)-1** ***H*** **-indole-2-carboxamide (6w)** (50%) as an off-white solid. ^1^H NMR (400 MHz, DMSO-*d*_6_): δ 11.84 (s, 1H), 8.54 (s, 1H), 8.50 (d, *J =* 4.8 Hz, 1H), 7.70–7.64 (m, 2H), 7.43–7.38 (m, 2H), 7.19 (dd, *J =* 4.7, 1.9 Hz, 1H), 6.76 (br s, 1H), 4.99–4.76 (m, 2H), 3.53–3.40 (m, 2H), 2.86 (m, 2H), 2.35–2.32 (m, 2H), 1.84–1.81 (m, 1H), 1.49 (s, 2H), 1.09–0.82 (m, 2H) ppm; ^13^C NMR (75 MHz, DMSO-*d*_6_) δ 163.89, 149.23, 148.96, 135.58, 134.71, 133.68, 132.11, 128.43, 124.66, 124.14, 123.83, 120.95, 114.07, 103.62, 46.06, 35.17, 30.92; HRMS: [M + H]^+^ calcd for C_21_H_24_ClN_4_O = 383.1639; found = 383.1630. HPLC Purity: 99%, *t*_R_ = 4.77 min.

##### 5-Chloro-*N*-(piperidin-4-yl)-*N*-(pyridin-3-ylmethyl)-1*H*-indole-2-carboxamide hydrochloride (**6x**)

The title compound was prepared according to General procedure A1, B1, and C1, starting from *tert*-butyl 4-aminopiperidine-1-carboxylate and nicotinaldehyde. The crude product was purified by flash chromatography (2% MeOH in dichloromethane) to afford **5-chloro-** ***N*** **-(piperidin-4-yl)-** ***N*** **-(pyridin-3-ylmethyl)-1** ***H*** **-indole-2-carboxamide hydrochloride (6x)** (46%) as a white solid. ^1^H NMR (500 MHz, DMSO-*d*_6_) δ 11.95 (s, 1H), 9.23 (s, 1H), 9.16 (s, 1H), 8.91 (s, 1H), 8.81 (d, *J* = 5.3 Hz, 1H), 8.46 (d, *J* = 7.9 Hz, 1H), 7.98 (dd, *J* = 7.9, 5.6 Hz, 1H), 7.67 (s, 1H), 7.47 (d, *J* = 8.7 Hz, 1H), 7.21 (dd, *J* = 8.7, 2.0 Hz, 1H), 6.89 (s, 1H), 4.91 (s, 2H), 4.71 (s, 1H), 3.29 (d, *J* = 11.9 Hz, 2H), 2.99 (dd, *J* = 23.1, 11.8 Hz, 2H), 2.25 (d, *J* = 10.7 Hz, 2H), 1.95 (d, *J* = 10.0 Hz, 2H). ^13^C NMR (126 MHz, CD_3_OH) δ 164.88, 147.61, 147.48, 135.61, 135.13, 134.78, 130.79, 128.28, 125.49, 124.04, 123.89, 120.50, 112.84, 103.24, 65.55, 54.89, 44.17 (2C) and 29.07 (2C). HRMS: [M + H]^+^ calcd for C_20_H_22_ClN_4_O = 369.1477; found = 369.1475. NMR Purity ∼95%.

##### *N*-(Piperidin-4-yl)-*N*-(pyridin-3-ylmethyl)-5-(trifluoromethyl)-1*H*-indole-2-carboxamide (**6y**)

The title compound was prepared according to General procedure A1, B1, and C1, starting from *tert*-butyl 4-aminopiperidine-1-carboxylate and nicotinaldehyde. The crude product was purified by flash chromatography (2% MeOH in dichloromethane) to afford ***N*** **-(piperidin-4-yl)-** ***N*** **-(pyridin-3-ylmethyl)-5-(trifluoromethyl)-1** ***H*** **-indole-2-carboxamide** (**6y**) (46%) as a white solid. ^1^H NMR (500 MHz, DMSO-*d*_6_) δ 12.22 (s, 1H), 9.27 (s, 2H), 8.90 (s, 1H), 8.79 (d, *J* = 5.2 Hz, 1H), 8.42 (d, *J* = 7.3 Hz, 1H), 8.05 (s, 1H), 7.98 – 7.89 (m, 1H), 7.65 (d, *J* = 8.6 Hz, 1H), 7.50 (d, *J* = 8.6 Hz, 1H), 7.09 (s, 1H), 4.91 (s, 2H), 4.71 (s, 1H), 3.29 (d, *J* = 11.8 Hz, 2H), 2.99 (dd, *J* = 22.2, 11.3 Hz, 2H), 2.28 (d, *J* = 10.8 Hz, 2H), 1.96 (d, *J* = 9.4 Hz, 2H). ^13^C NMR (126 MHz, DMSO-*d*_6_) δ 164.00, 142.28, 138.58, 137.89, 131.95, 126.74, 126.72, 125.82 (q, *J* = 271.4 Hz), 121.07 (q, *J* = 31.2 Hz), 120.14 (d, *J* = 2.6 Hz), 120.02 (d, *J* = 4.1 Hz), 113.50, 105.35, 82.19 – 78.10 (m), 53.65, 42.96, 38.72, 26.77. HRMS: [M + H]^+^ calcd for C_21_H_21_F_3_N_4_OH = 403.1740; found = 403.1733. NMR Purity: ∼ 95%.

##### 5-Chloro-*N*-[(1-methylpyrrolidin-3-yl)­methyl]-*N*-(pyridin-3-ylmethyl)-1*H*-indole-2-carboxamide (**7a**)

The title compound was prepared according to General procedure A1, D1, E1, and B1, starting with 5-chloro-*N*-(pyridin-3-ylmethyl)-*N*-(pyrrolidin-3-ylmethyl)-1H-indole-2-carboxamide (TFA salt) (100 mg, 0.21 mmol) in dichloromethane (5 mL) was added DIPEA (3.0 equiv) and stirred for 10 min. The mixture was concentrated, and the residue was dissolved in MeOH (50 mL). Acetic acid (0.1 mL) was added to the reaction mixture followed by aqueous formaldehyde (0.5 mL, 4.14 mmol 37% in water). The mixture was stirred for 1 h then NaCNBH_3_ (39 mg, 0.62 mmol) was added, and the reaction continued for 6 h. The reaction mixture was diluted with dichloromethane and washed with saturated aqueous NaHCO_3_ solution. The organic layer was dried over anhydrous sodium sulfate and concentrated. The crude product was purified by preparative HPLC to afford **5-chloro-** ***N*** **-[(1-methylpyrrolidin-3-yl)­methyl]-** ***N*** **-(pyridin-3-ylmethyl)-1** ***H*** **-indole-2-carboxamide** (**7a)** (30 mg, 38%) as a gummy liquid.

^1^H NMR (400 MHz, DMSO-*d*_6_): δ 12.06 (br s, 1H), 8.55 (s, 1H), 8.49 (d, *J =* 4.2 Hz, 1H), 7.75–7.65 (m, 1H), 7.64 (s, 1H), 7.44–7.38 (m, 2H), 7.19 (dd, *J =* 8.7, 1.8 Hz, 1H), 6.94 (br s, 1H), 4.83 (s, 2H), 3.70–3.50 (m, 2H), 2.57–2.33 (m, 5H), 2.21 (s, 3H), 1.85–1.75 (m, 1H), 1.35–1.25 (m, 1H) ppm; LC-MS *m*/*z*: 383.25. HPLC Purity: 98%, *t*_R_ = 4.80 min.

### Biology

#### *P. falciparum* Asexual Blood Stages Parasite Culture

The *P. falciparum* strains were cultured in human erythrocytes maintained in RPMI 1640 medium (Sigma-Aldrich), supplemented with 0.2% NaHCO_3_, 25 mM HEPES, 11 mM d-glucose, 10 mg/L hypoxanthine, 25 mg/L gentamicin, and 0.5% (m/v) AlbuMAX II, essentially as previously described by Trager and Jensen, 1976. The culture medium was routinely changed daily, and culture flasks maintained under a 90% N_2_, 5% CO_2_, 5% O_2_ gas mixture at 37 °C.^34^

#### *P. falciparum* Sexual Blood Stages Parasite Culture

*P. falciparum* NF54 strain was cultured as previously described,^35^ initiating gametocyte cultures at 1% asexual parasitemia and 4% hematocrit in 40 mL final volume. Complete culture medium (RPMI 1640 with 25 mM HEPES, 50 μg/mL hypoxanthine, 2 g/L NaHCO_3_, and 10% human A+ serum) was replaced daily for 14 days to induce gametocyte production without adding new erythrocytes. On day 14, Gametocyte production was assessed by thin smear, Giemsa staining, and exflagellation counting. Cultures with >0.2% exflagellation were used for assays.

#### *P. falciparum* Dual Gamete Formation Assay (Pf DGFA)

In screening mode, mature gametocyte cultures (>0.2% exflagellation) were diluted to 14 million cells/ml and dispensed into 384-well plates. Parasites were incubated with compounds for 48 h in a humidified chamber, then gamete formation was triggered by adding ookinete medium containing anti-Pfs25 and cooling the plate. Exflagellation was immediately recorded by brightfield microscopy, followed by a 24-h incubation at 28 °C to allow female gametes to express Pfs25. Female gamete formation was recorded by fluorescence microscopy.^35^ Exflagellation and gamete formation were quantified using ICY Image Analysis software.^36^

#### Biological Activity of Compounds against *P. falciparum* Blood-Stage Parasites *In Vitro*

The antiplasmodial activity of compounds was evaluated against different strains of *P. falciparum* blood parasites (RF12, Dd2, K1, 7G8, TM90-2CB, 3D7, Dd2, and NF54). The parasites were obtained through MR4 as part of the BEI Resources Repository. The parasites were synchronized at ring stage using sorbitol treatment and the parasitemia were microscopically evaluated by Giemsa-stained blood smears.^37^ The culture with 0.5% parasitemia, 2.5% hematocrit was added to 96-well plates and incubated with the compounds in concentrations from 10 to 0.01 μM; 1 to 0.001 μM, obtained through 7 serial dilutions factor 2. The culture of uninfected erythrocytes and infected erythrocytes without any treatment was maintained in parallel as control negative and positive, respectively. DMSO concentration was maintained below 0.05% (v/v). The plates were incubated for 72 h at 37 °C in a humidified incubator with a gas mixture of 90% N_2_, 5% O_2_, and 5% CO_2_. Once completed the period of incubation, the culture medium was removed, and the cells were resuspended in 100 μL PBS (116 mM NaCl, 10 mM NaH_2_PO_4_, 3 mM KH_2_PO_4_) and lysed with 100 μL lysis buffer (20 mM Tris base, 5 mM EDTA, 0.0008% (v/v) Triton X-100, 0.008% (m/v) saponin, pH 8.0) containing 0.002% (v/v) SYBR Green I.^38^ The plates were incubated at room temperature for 30 min and the fluorescence corresponding to the density of parasites was determined using a SpectraMAX Gemini EM plate reader (Molecular Devices Corp., Sunnyvale, CA) (λ_Ex_:485 nm and λ_Em_: 535 nm). The half-maximal inhibitory concentration (IC_50_) was determined by nonlinear regression analysis of the concentration–response curve using the GraphPad Prism 8 program (GraphPad Software, San Diego, California).

#### Resistance Assessment

The antiplasmodial activity of compounds was assessed against a panel of *P. falciparum* strains: 3D7 (chloroquine-sensitive), K1 (resistant to chloroquine, mefloquine, and sulfadoxine), and Dd2 (resistant to chloroquine, mefloquine, and pyrimethamine). The assay to determine the IC_50_ value of compounds against the panel of resistant strains was conducted as described above. After the determination of the IC_50_ value for each resistant strain, a resistance index (RI) was calculated using the following equation: RI *=* IC_50_ Resistant strain/IC_50_^3D7^ (RI values greater than 5 were considered indicative of cross-resistance).^31^

#### Effect of Verapamil on Resistance of *P. falciparum* to Indole Compounds

The 3D7 (chloroquine-sensitive) and Dd2 (chloroquine resistance) cultures with 0.5% parasitemia, 2.5% hematocrit were added to 96-well plates and incubated with **6f** and **6x** in concentrations from 50 to 0.05 μM, and chloroquine in concentrations from 3 to 0.03 μM, obtained through 7 serial dilutions factor 2, in the presence or absence of 10 μM of Verapamil (VP). After 72 h of incubation at 37 °C in a humidified incubator with a gas mixture of 90% N_2_, 5% O_2_, and 5% CO_2_, the parasite proliferation was evaluated using the SYBR green method.

#### *P. falciparum* Stage-Specificity Assay

To determine the specific asexual blood stage at which the compounds were more active we applied the protocol described previously.^39^ *P. falciparum* parasites were synchronized at the ring stage using the sorbitol lysis method as described by Lambros and Vanderberg (1979).^37^ Briefly, cultures containing mixed-stage parasites were centrifuged at 500*g* for 3 min, and the resulting pellet was resuspended in 5% D-sorbitol solution prewarmed to 37 °C. The suspension was incubated at 37 °C for 10 min to selectively lyse late parasites, while ring-stage-infected erythrocytes remained intact. Following incubation, the cells were washed twice with RPMI 1640 medium and resuspended in complete culture medium. When the majority of parasites had developed to the schizont stage, the cultures were centrifuged at 500*g* for 5 min, and the pellet was resuspended in incomplete RPMI medium. The suspension was applied to pre-equilibrated LS columns mounted on a magnetic MACS separator (Miltenyi Biotec). Schizonts were then purified magnetically using MACS LS columns, which selectively retain schizont-infected erythrocytes due to their hemozoin content. Nonmagnetic cells were removed through sequential washes with RPMI, and enriched schizonts were eluted by removing the column from the magnetic field and flushing it with prewarmed medium. The purified schizonts were returned to culture at 2% hematocrit to allow for merozoite egress and reinvasion. After invasion was complete, cultures were again synchronized with 5% sorbitol to eliminate remaining late stages and obtain a highly synchronized ring-stage population (defined as time = 0 h). These parasites were then plated in five 96-well plates and exposed to compounds as early rings (0–8 h), late rings (8–16 h), early trophozoites (16–24 h), late trophozoites (24–32 h) or schizonts (32–40 h). The growth inhibition was assessed at the 60 h time point at which parasites had expanded, reinvaded new RBCs, and developed into the trophozoite stage that allows straightforward quantification. Parasite survival for both the 72 h and stage-specific 8 h exposures was assessed by SYBR Green assay. IC_50_ values were derived from growth inhibition data using nonlinear regression (GraphPad Prism 8.0.1). All asexual blood stage assays were repeated on at least three independent occasions with two technical replicates.

#### *Ex Vivo* Field Isolates Activity

This study received ethical approval from the Centro de Pesquisa em Medicina Tropical (CEPEM) in Rondônia (CAAE 58738416.1.0000.0011). Written informed consent was obtained from all participants prior to blood collection, which was carried out by a trained nurse.

Clinical isolates of *P. falciparum* were obtained in March and April 2021 from patients attending CEPEM in Porto Velho, Rondônia, located in the western Brazilian Amazon. Only individuals with confirmed *P. falciparum* monoinfection, parasitemia between 2000 and 80,000 parasites/μL, and ≥70% ring-stage parasites were enrolled. Exclusion criteria included prior antimalarial treatment within the past month or presentation with severe malaria symptoms. The final study cohort comprised 24 individuals residing in this high-transmission area. Peripheral blood samples (5 mL) were collected via venipuncture into heparinized tubes and immediately used for ex vivo drug susceptibility assays on predosed plates containing diluted antimalarial compounds. The test compound **6f**, along with control drugs (artesunate and chloroquine), were prepared as 2 mM stock solutions in DMSO (from dilution of solution A), then further diluted in assay medium to initial concentrations of 0.1 μM for artesunate and 50 μM for chloroquine and 100 μM for **6f**. 2-fold serial dilutions were performed in assay medium, and 20 μL of each dilution was added to the assay plates, followed by 10-fold dilution into the final parasite-containing medium.^40,41^ For sample processing, 5 mL of whole blood was centrifuged at 800*g* for 10 min. Plasma and buffy coat were removed, and the red blood cell (RBC) pellet was washed with RPMI 1640 medium, adjusted to 50% hematocrit, and filtered through a CF11 cellulose column. The resulting packed infected RBCs (iRBCs) were then diluted to 2% hematocrit using complete RPMI 1640 medium supplemented with 20% human serum. Control assays using the 3D7 laboratory strain were also performed under the same conditions. iRBCs (180 μL per well) were distributed into the drug-preloaded assay plates. The plates were incubated in a hypoxia chamber (5% O_2_, 5% CO_2_, 90% N_2_) at 37 °C for 40 to 47 h to allow parasite maturation from ring to schizont stages. Drug-free control wells were cultured in parallel. The assay was concluded once 40% of the parasites in control wells had matured to schizonts, defined by the presence of at least three nuclei per parasite. Thick blood smears were prepared from each well, air-dried, stained with 5% Giemsa for 30 min, and microscopically examined. Schizonts were counted among 200 asexual-stage parasites per well, and results were normalized to the drug-free control (set as 100%). The half-maximal effective concentration (EC_50_) was determined by fitting dose–response curves using OriginLab software (OriginLab Corporation, Northampton, MA), based on parasite growth relative to controls.

#### Resazurin Assay for Cytotoxicity Evaluation

HepG2 cells were trypsinized, counted, and distributed in a 96-well plate at a density of 30,000 cells per well (180 μL). The plate was incubated at 37 °C and 5% CO_2_ for 24 h. Following incubation, 20 μL of serial dilutions of the tested compounds were added to the wells, with concentrations ranging from 30 to 0.47 μM. The plate was incubated for an additional 72 h at 37 °C and 5% CO_2_. Untreated cells served as positive controls, while wells containing only medium were used as negative controls. Postincubation, microscopy was employed to determine the highest compound concentration for treatment results. Cytotoxicity was assessed by adding 40 μL of resazurin (0.15 mg/mL) to each well, followed by 4 h of incubation at 37 °C and 5% CO_2_. Fluorescence intensity was measured using a SpectraMAX Gemini EM plate reader (excitation wavelength at 560 nm, emission wavelength at 590 nm) and analyzed against controls with GraphPad Prism version 8.0.1 software. Concentration–response curves were generated, and half-maximal inhibitory concentration (CC_50_) values were determined using nonlinear regression analysis. The selectivity index (SI) was calculated by the ratio of CC_50_ to IC_50_, with compounds having an SI over 10 generally considered selective.

#### *P. falciparum’s* Digestive Vacuole Homeostasis

Synchronous trophozoites of *P. falciparum* (3D7 strain) were marked with the lysosomotropic probe acridine orange (AO) (Sigma-Aldrich) with modifications.^33^ Briefly, the culture with 10% parasitemia was centrifuged for 5 min at 9000*g* and resuspended in RPMI without phenol red. The RBC number was determined using a Neubauer chamber and it was adjusted to 1 × 107 RBC.mL^–1^ of MOPS (116 mM NaCl, 5.4 mM KCl, 0.8 mM MgSO_4_, 5.5 mM d-glucose, 50 mM MOPS, and 2 mM CaCl_2_, pH 7.2) supplemented with 5 μM AO. The sample was incubated for 40 min at 37 °C. After that, cells were washed three times and 700 μL were and the fluorescence measured before and after a 3 min-treatment with compounds **6d** and **6f** and CQ at 10 μM. Fluorescence was measured in a Hitachi F-7000 fluorimeter (Tokyo, Japan) by continuous measurement of the fluorescence (λ_ex_ = 488 nm; λ_em_ = 560 nm) at 37 °C. Experiments were performed in triplicate.

#### Automated Patch-Clamping of hERG Potassium Channels Expressed in CHO Cells

hERG currents were recorded from stably transfected CHO cells (hERG DUO, B’SYS GmbH) using automated patch-clamping (Q-Patch, Sophion). Cells were cultivated under standard conditions and passed at a confluence of 50 to 80%. Extracellular solution for electrophysiological experiments contained (in mM) 137 NaCl, 4 KCl, 1.8 CaCl_2_, 1 MgCl_2_, 10 HEPES, 10 d-Glucose, pH (NaOH) 7.4, intracellular solution contained (in mM) 130 KCl, 2 CaCl_2_, 4 MgCl_2_, 4 Na_2_ATP, 10 HEPES, 5 EGTA, pH (KOH) 7.2. After forming GΩ seal and whole cell configuration, cells were clamped to −80 mV and depolarized to +20 mV for 2 s, followed by a voltage step to −40 mV for 3 s, frequency: 0.1 Hz, sampling frequency: 1 kHz. The tail current amplitudes were analyzed. Increasing concentrations of the test item were perfused for at least 250 s per concentration. The steady state current amplitude in the presence of test item was analyzed and normalized to the initial current amplitude of the same cell in duplicates. Normalized and average current amplitudes were fitted with a logistic equation to determine IC_50_ and Hill coefficient.

#### Liver Microsome Stability

A solution of the test compounds in phosphate buffer solution (1 μM) was incubated in pooled human or mouse liver microsomes (0.5 mg/mL) for 0, 5, 20, 30, 45, and 60 min at 37 °C in the presence and absence of an NADPH regeneration system (NRS). The tests were conducted by TCGLS, Kolkata, India. The reaction was terminated with the addition of ice-cold acetonitrile, containing a system suitable standard, at designated time points. The sample was centrifuged (4200*g*) for 20 min at 20 °C and the supernatant was diluted by half in water and then analyzed by LC-MS/MS. The % parent compound remaining, half-life (*t*_1/2_) and clearance (CLint) were calculated using standard methodology. The experiment was carried out in duplicate. Verapamil, diltiazem, phenacetin, and imipramine were used as reference standards.

#### Hepatocyte Stability

The solution of the test compound in Krebs-Henseleit buffer solution (1 μM) was incubated in pooled rat hepatocytes (1 × 10^6^ cells/mL) for 0, 15, 30, 45, 60, 75, and 90 min at 37 °C (5% CO_2_, 95% relative humidity). The reaction was terminated with the addition of ice-cold MeCN at designated time points. The samples were then centrifuged (4200 rpm) for 20 min at 20 °C, and the supernatant was half diluted in water and then analyzed using LCMS/MS. The % parent compound remaining, half-life (*t*_1/2_), and clearance (CLint) were calculated using standard methodology. The experiment was carried out in duplicate. Diltiazem, 7-Ethoxy Coumarin, Propranolol, and Midazolam were used as reference standards.

#### Solubility

Kinetic solubility assay was performed using UV–vis detection. Compound (200 μM in DMSO) was incubated in a solution of phosphate-buffered saline (PBS, pH 7.4) with constant shaking (600 rpm) at 25 °C for 2 h. The samples were filtered using a multiscreen solubility filter plate. The filtrate was diluted by 50% with MeCN. A five-point linearity curve was prepared in PBS/MeCN (1:1, v/v) at 200, 150, 75, 25, and 2.5 μM. Blank, linearity, and test samples (*n* = 2) were transferred to a UV-readable plate, and the plate was scanned for absorbance. Best-fit calibration curves were constructed using the calibration standards and used to determine the test sample solubility. The experiment was carried out in duplicate. Diethylstilbestrol, Haloperidol, and Diclofenac Sodium were used as reference standards.

#### eLogD at pH 7.4

eLogD at pH 7.4 was determined using a miniaturized shake flask method. A solution of a presaturated mixture of 1-octanol and phosphate-buffered saline (PBS) (1:1, v/v) and the test compound (75 μM) was incubated at 25 °C with constant shaking (850 rpm) for 2h. After incubation, the organic and aqueous phases were separated, and samples of each phase were transferred to a plate for dilution. The organic phase was diluted 1000-fold, and the aqueous phase was diluted 20-fold. The samples were quantitated using LC-MS/MS. The experiment was carried out in duplicate. Propranolol, Amitriptyline, and Midazolam were used as reference standards.

## Supplementary Material



## Notes

General experimental procedures and details of spectroscopic data (^1^HNMR, ^13^C-NMR, LCMS and HRMS) of final compounds (PDF)
