# Supplementary material for: Indole-2-carboxamides Optimization for Antiplasmodial Activity
Source: ACS Bio Med Chem Au. 2025 Jul 31;5(5):821–39. doi: 10.1021/acsbiomedchemau.5c00058 (PMC12531871; doi:10.1021/acsbiomedchemau.5c00058)
Supplement: Supplementary file 1 [file bg5c00058_si_001.pdf]

Indole-2-carboxamides optimization for anti-plasmodial activity

Malkeet Kumar,[a&] Anees Ahmad,[a&] Anna Caroline Campos Aguiar,[b,c&] Sarah El Chamy Maluf,[b&] Anwar Shamim,[a] Mariana Ferrer,[a] Guilherme E. Souza,[b] Marcos L. Gazarini,[d] Dhelio B. Pereira,[e] Thomas W. von Geldern,[f] Delphine Baud,[f] Barry Jones,[g] Susanta Kumar Mondal,[h] Paul A. Willis,[f] Rafael Victorio Carvalho Guido,[b*] Luiz Carlos Dias,[a*]

AUTHOR ADDRESS

[a] Institute of Chemistry, University of Campinas, Barão Geraldo, PO Box 6154, Campinas, SP, 13083-970, Brazil

[b] Sao Carlos Institute of Physics, University of Sao Paulo, IFSC – USP, 13566-590, Sao Carlos, SP, Brazil

[c] Department of Microbiology, Immunology and Parasitology, University Federal of Sao Paulo, Ed. Ciências Biomédicas - R. Botucatu, 862 - Vila Clementino, São Paulo - SP, 04023-062, Brazil

[d] Department of Biosciences, University Federal of Sao Paulo, Ed. Ciências Biomédicas - R. Silva Jardim, 136 - Vila Mathias, Santos - SP, 11015-531, Brazil

[e] Centro de Pesquisa em Medicina Tropical, CEP 76812-329, Porto Velho, RO, Brazil

[f] Medicines for Malaria Venture, ICC, Route de Pré-Bois 20, P.O. Box 1826, 1215 Geneva, Switzerland

[g] Pharmaron UK, West Hill Innovation Park, Hoddesdon, EN11 9FH, UK

[h] TCG LifeSciences PVT. LTD, Block-BN, Plot-7, Sector-V, Salt Lake Electronic Complex, Kolkata – 700091, India

*Corresponding authors: rvcguido@usp.br; ldias@unicamp.br

[&]These authors contributed equally to this work

KEYWORDS. Malaria, Antimalarials, Indoles, Resistance, Drug Development

Experimental Procedures

Experimental protocol:

All the starting materials and solvents were purchased from commercial sources or synthesized according to the literature procedure. Organic solutions were dried over anhydrous sodium sulfate.

Unless noted, all reactions were performed under an atmosphere of argon with dry solvents and magnetic stirring. Dichloromethane (DCM) and triethylamine (Et_3N) were distilled from CaH_2. Tetrahydrofuran (THF) was distilled from sodium/benzophenone. Yields refer to homogeneous materials obtained after purification of reaction products by flash column chromatography using silica gel (200-400 mesh), liquid-liquid extraction or recrystallization. Analytical thin-layer chromatography was performed on silica gel 60 and GF (5-40 μm thickness) plates, and visualization was accomplished using UV light, basic potassium permanganate staining, or ninhydrin solution followed by heating. 1H and proton-decoupled ^{13}C NMR spectra were acquired in CD_3OD or DMSO-d_6 at 400 MHz (^1H) and 75 or 126 MHz (^{13}C) (Bruker Avance 400). Chemical shifts (δ) are reported in ppm using residual non-deuterated solvent as an internal standard CD_3OD at 3.31 ppm, DMSO-d_6 at 2.50 ppm, and TMS at 0.00 ppm for ^1H NMR spectra and CD_3OD at 49.0 ppm, DMSO-d_6 at 39.52 ppm for ^{13}C NMR spectra). High resolution mass spectrometry (HRMS) was measured using electrospray ionization (ESI) (waters 3 xevo Q-tof, thermo LTQ-FT ultra, or thermos Q exactive) or using electron ionization (EI) (GCT premier waters).

General Procedures:

General Procedure A1: Reductive amination

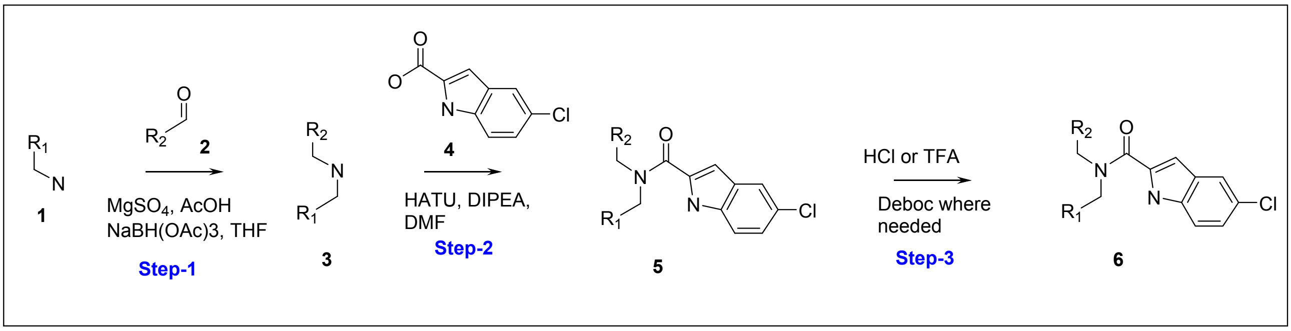

To a stirred solution of amine (**1**) and aldehyde (**2**) (1 equiv.) in THF was added anhydrous $MgSO_4$ (4 equiv.) The reaction mixture was cooled to 0 °C and acetic acid (2 equiv.) was added. The mixture was stirred at room temperature for 2 h. The reaction mixture was cooled to 0 °C and $NaBH(OAc)_3$ (2 equiv.) was added portion-wise. The reaction mixture was stirred at room temperature for 16 h. The

reaction mixture was poured into a mixture of ice water and saturated aqueous $NaHCO_3$. The reaction mixture was extracted with dichloromethane and the combined organic part was washed with water, brine, dried over anhydrous sodium sulfate and concentrated under reduced pressure to afford a crude product which was used for the next step without further purification.

General Procedure B1: Amidation

To a solution of acid (**4**) (1 equiv.) in DMF were added HATU (1 equiv.), and DIPEA (2 equiv.) and was stirred at room temperature for 5 minutes. Then the amine (**3**) (1 equiv.) was added, and the mixture was stirred at room temperature for 8-16 h. The reaction mixture was diluted with saturated $NaHCO_3$ solution, and the aqueous layer was extracted with ethyl acetate. The combined organic layer was washed with water, brine and dried over anhydrous sodium sulfate, and concentrated. The crude product was purified by Combiflash chromatography to afford the amide-**5**.

General Procedure C1: Boc removal

TFA (2 equiv.) was added to the carbamate (**5**) (1 equiv.) dropwise at 0 °C and stirred at room temperature for 2 h. After completion, the reaction mixture was concentrated under reduced pressure to afford crude products (**6**). The product was purified by column chromatography or preparative HPLC.

General Procedure D1: Tosylation

To a solution of 4-(hydroxymethyl)pyrrolidin-2-one (1 equiv.) (**8**) in dichloromethane were added trimethylamine (2 equiv.) and DMAP (0.1 equiv.) p-toluenesulfonyl chloride (1 equiv.) was added in small portions. The resulting solution was stirred at room temperature for 18 h. The reaction mixture was diluted with dichloromethane and washed with saturated aqueous $NaHCO_3$ solution. The organic layer was dried over anhydrous sodium sulphate and concentrated to afford (5-oxopyrrolidin-3-yl)methyl 4-methylbenzenesulfonate (**9**) which was used for the next step.

General Procedure E1: Nucleophilic Substitution

To a stirred solution of (5-oxopyrrolidin-3-yl)methyl 4-methylbenzenesulfonate (500 mg, 1.85 mmol) (9) in DMF were added 3-pyridylmethanamine (201 mg, 1.85 mmol) (**10**) and NaI (cat). Then, the reaction mixture was heated at 90 °C for 16 h. The reaction mixture was diluted with dichloromethane and washed with saturated aqueous $NaHCO_3$ solution. The organic layer was dried over anhydrous sodium sulphate and concentrated to afford 4-[(3-pyridylmethylamino)methyl]pyrrolidin-2-one (**11**) (480 mg, crude) which was used for the next step without purification.

5-chloro-N-(pyridin-3-ylmethyl)-N-((tetrahydrofuran-3-yl)methyl)-1H-indole-2-carboxamide (5r):

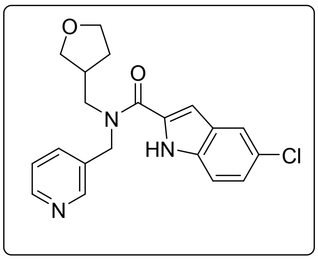

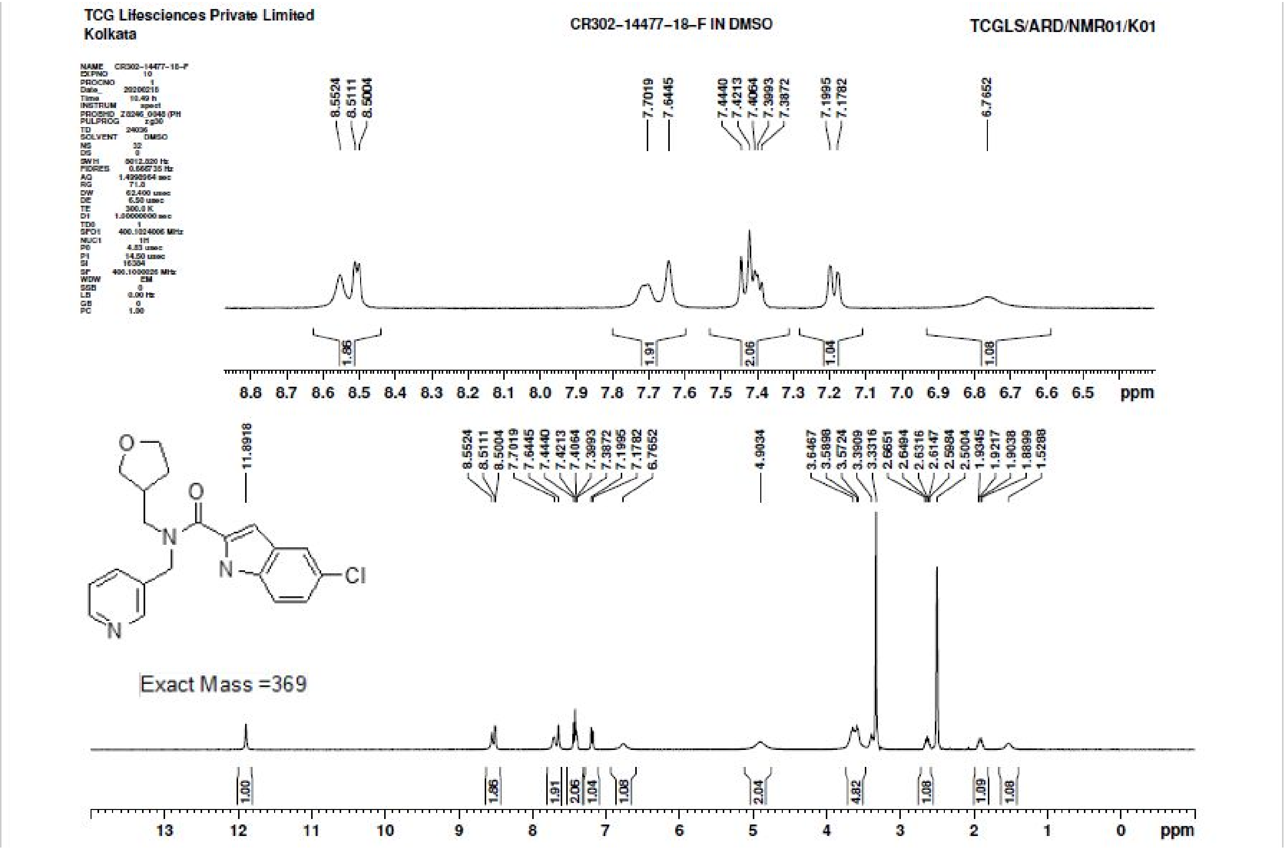

^{1}H NMR (400 MHz, DMSO-d_6)-5r

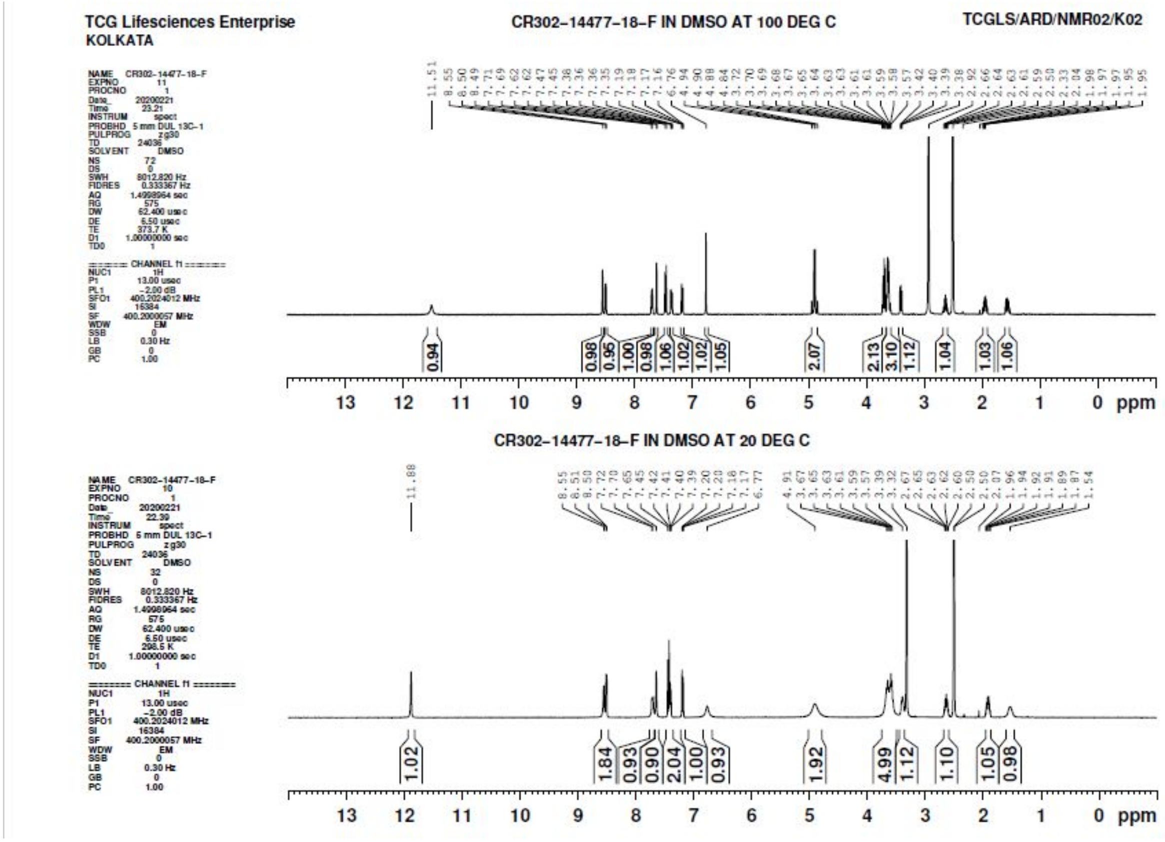

^{1}H NMR (20 °C and 100 °C) (400 MHz, DMSO-d_6)-5r

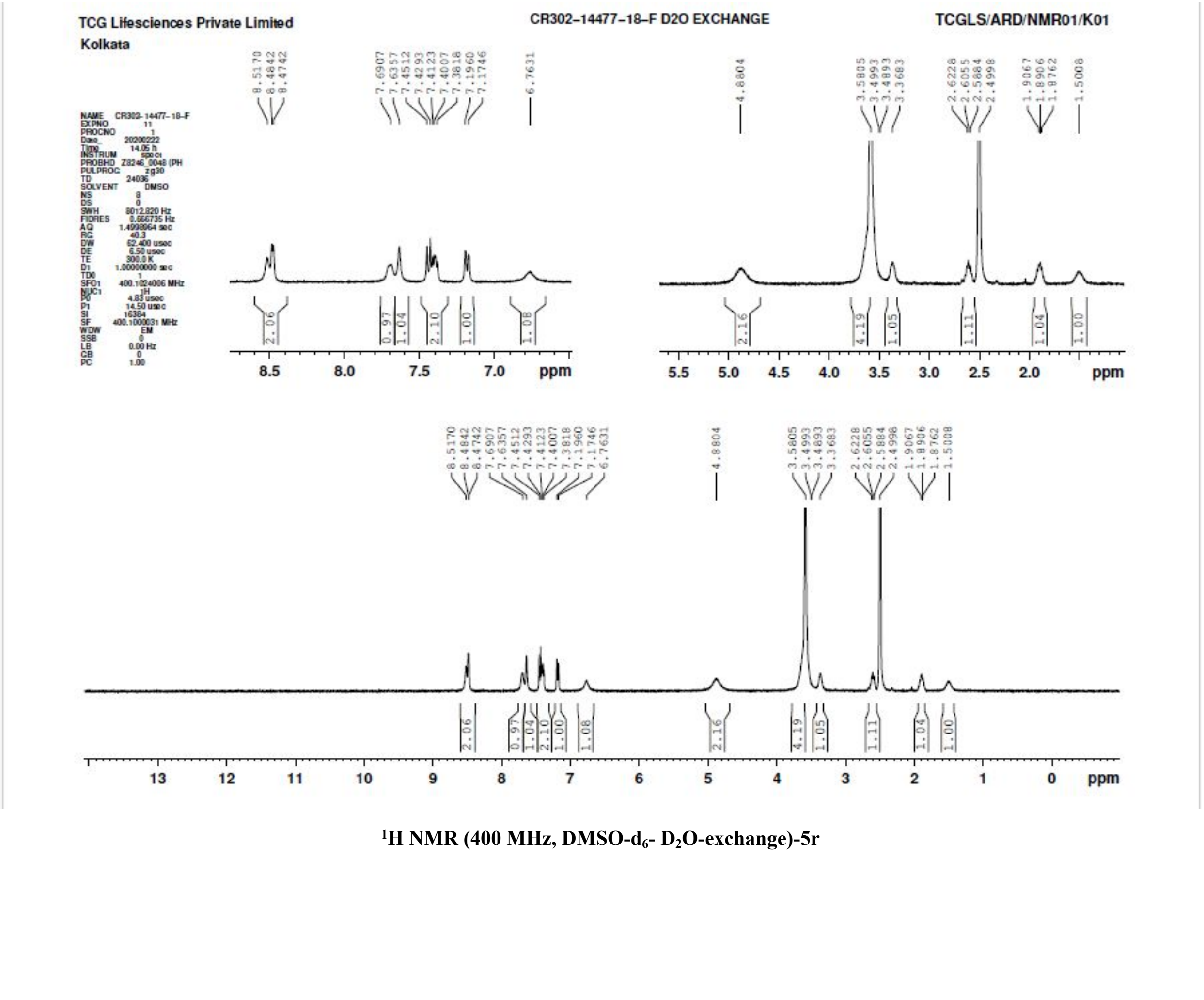

^{1}H NMR (400 MHz, DMSO-d_6- D_2O-exchange)-5r

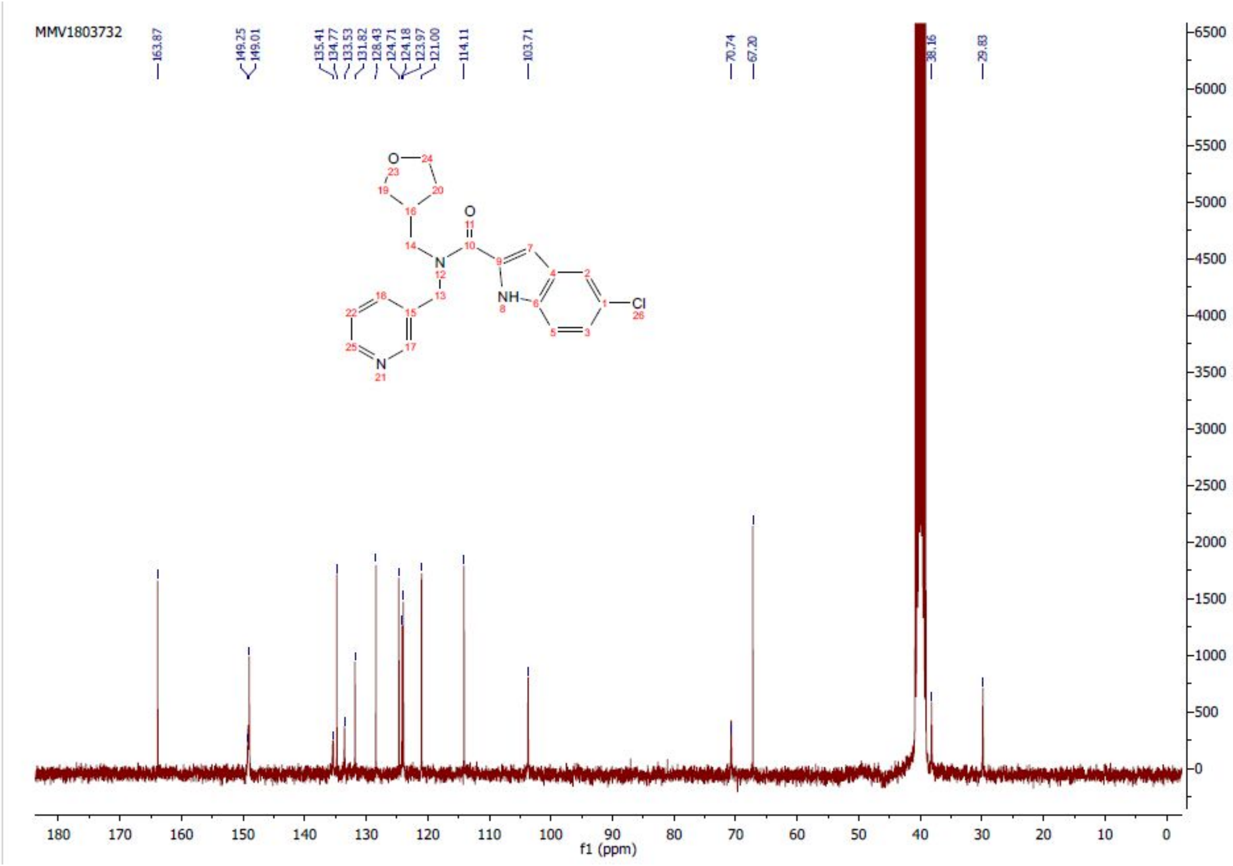

^{13}C NMR (75 MHz, DMSO-d_6)-5r

TCG Lifesciences Pvt Ltd , Kolkata , India

Data file:	D:\LCMS 2019\DATA\FEBRUARY-2020\18022020 2020-02-18 09-41-19\CR302-14477-18-F R.D
Sample name:	**CR302-14477-18-F**

Instrument:	TCGLS_ARD_LCMS20_K79	Location:	D1F-C5
Injection date:	2/18/2020 1:45:20 PM	Injection volume:	0.200
Acq. method:	Moni +ve-ve_FA_5MIN.M		
Description:	Column:YMC TRIART C18(33x2.1mm,3μ)-FAF		

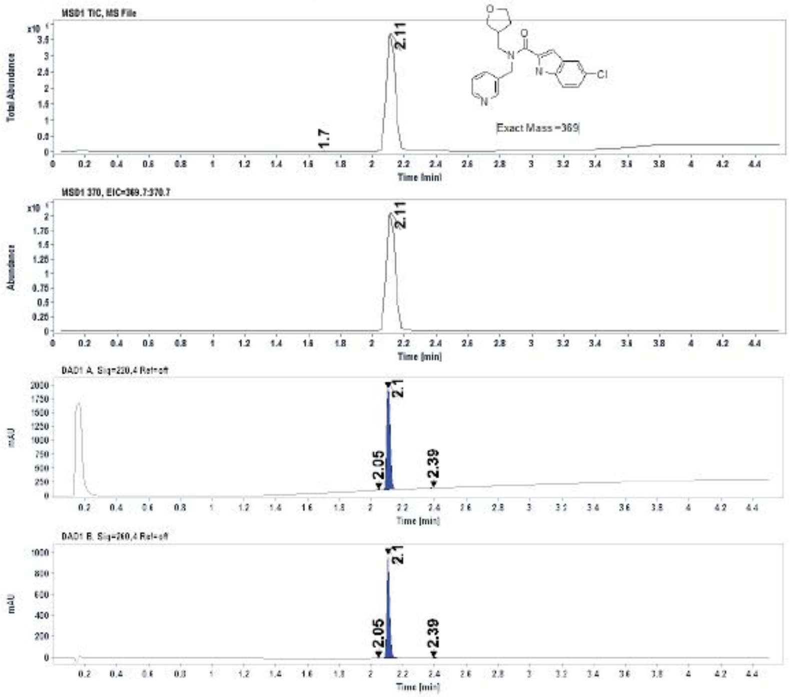

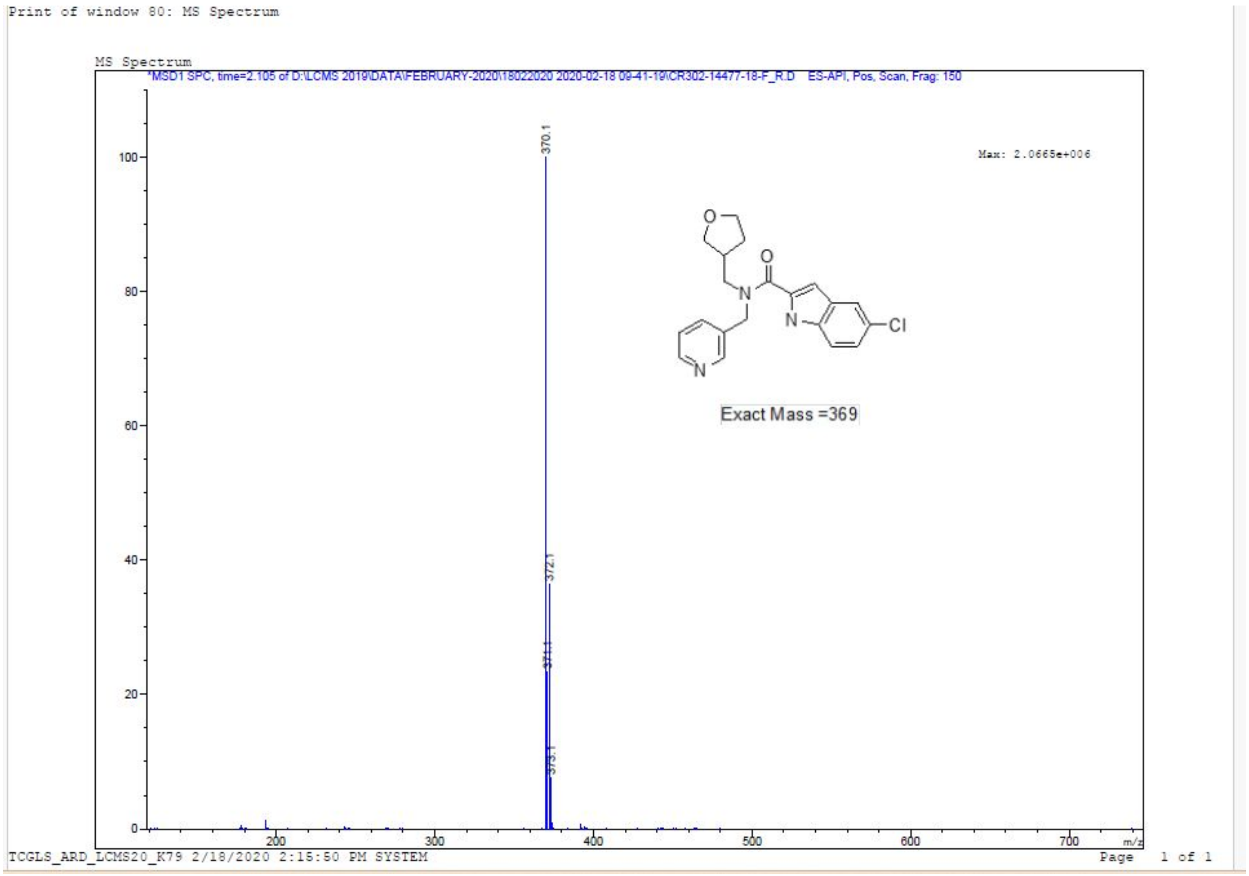

LCMS-5r

Instituto de Química	Resultados HRMS		LIE Laboratório Institucional de Espectrometria de Massas
	Responsável:	Diego C Andrade	
	Data:	06/10/22	
	**Laboratório Institucional de Espectrometria de Massas**		

Prof. Dr. Luiz Carlos Dias
Aluno Anwar Shamim
Amostra MMV732

Espectro completo de 50 a 750 m/z em modo positivo

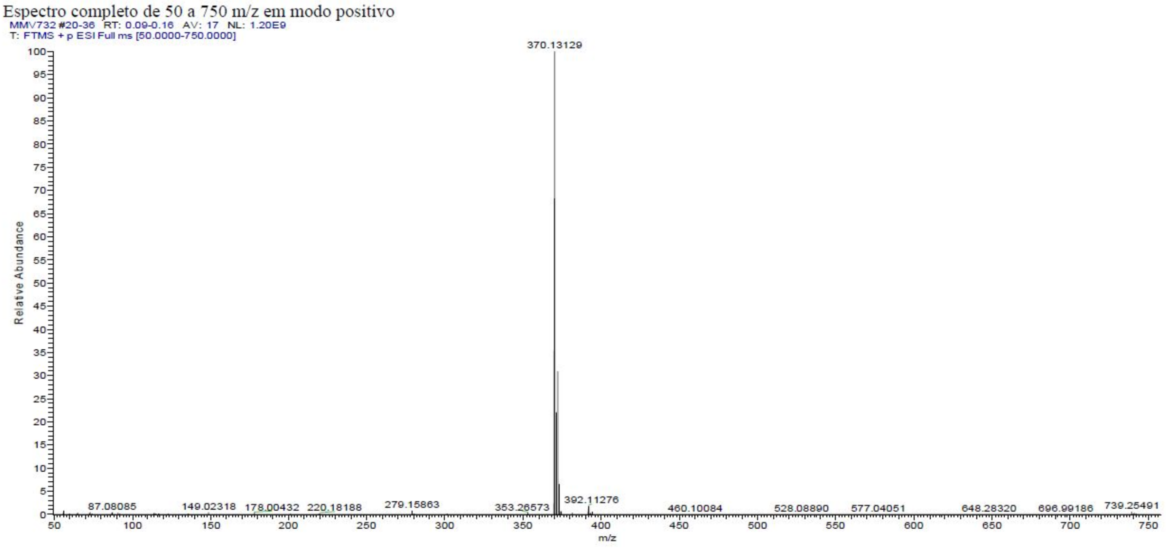

HRMS-5r

Instituto de Química	Resultados HRMS		LIE Laboratório Institucional de Espectrometria de Massas
	Responsável:	Diego C Andrade	
	Data:	06/10/22	
	**Laboratório Institucional de Espectrometria de Massas**		

Comparação entre espectro real (superior) e simulado (inferior) para C20H20ClN3O2H+

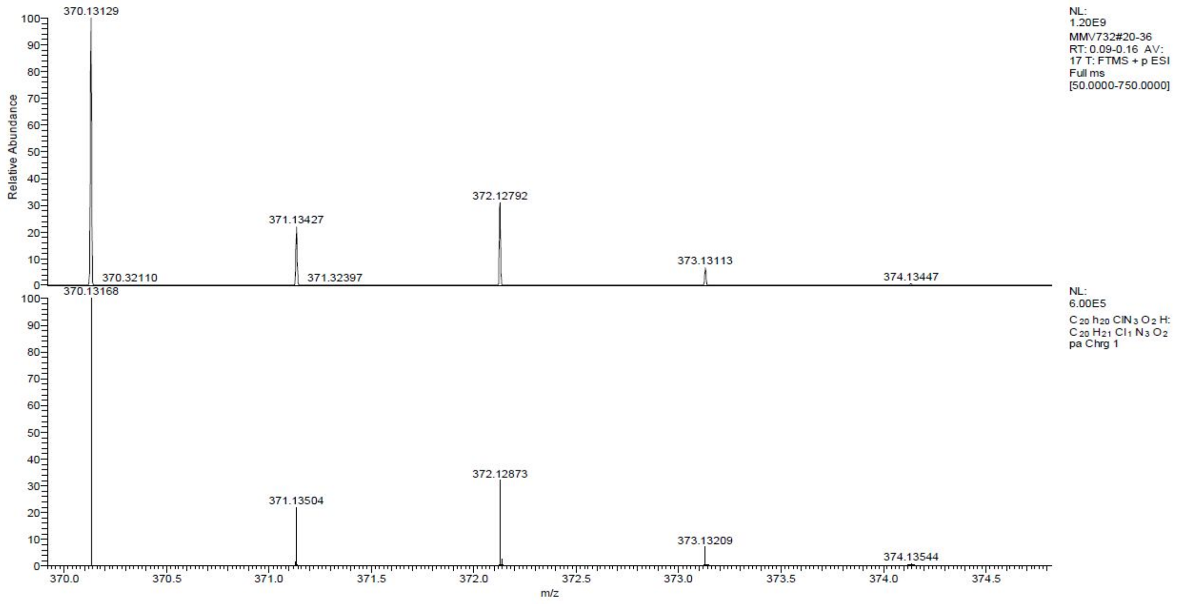

HRMS-5r

5-chloro-N-[(5-oxopyrrolidin-3-yl)methyl]-N-(3-pyridylmethyl)-1H-indole-2-carboxamide (5s)

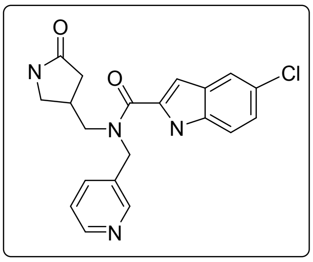

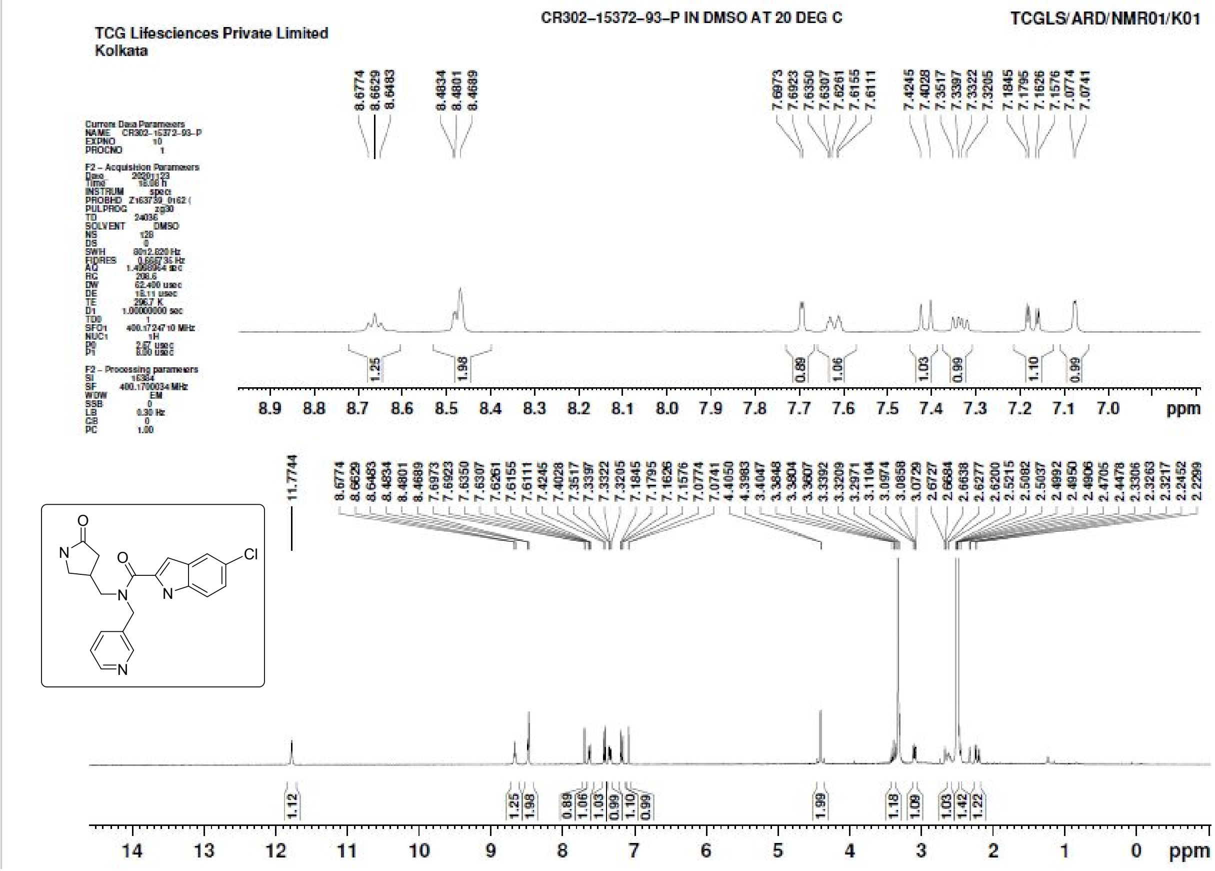

^{1}H NMR (400 MHz, DMSO-d_6)-5s

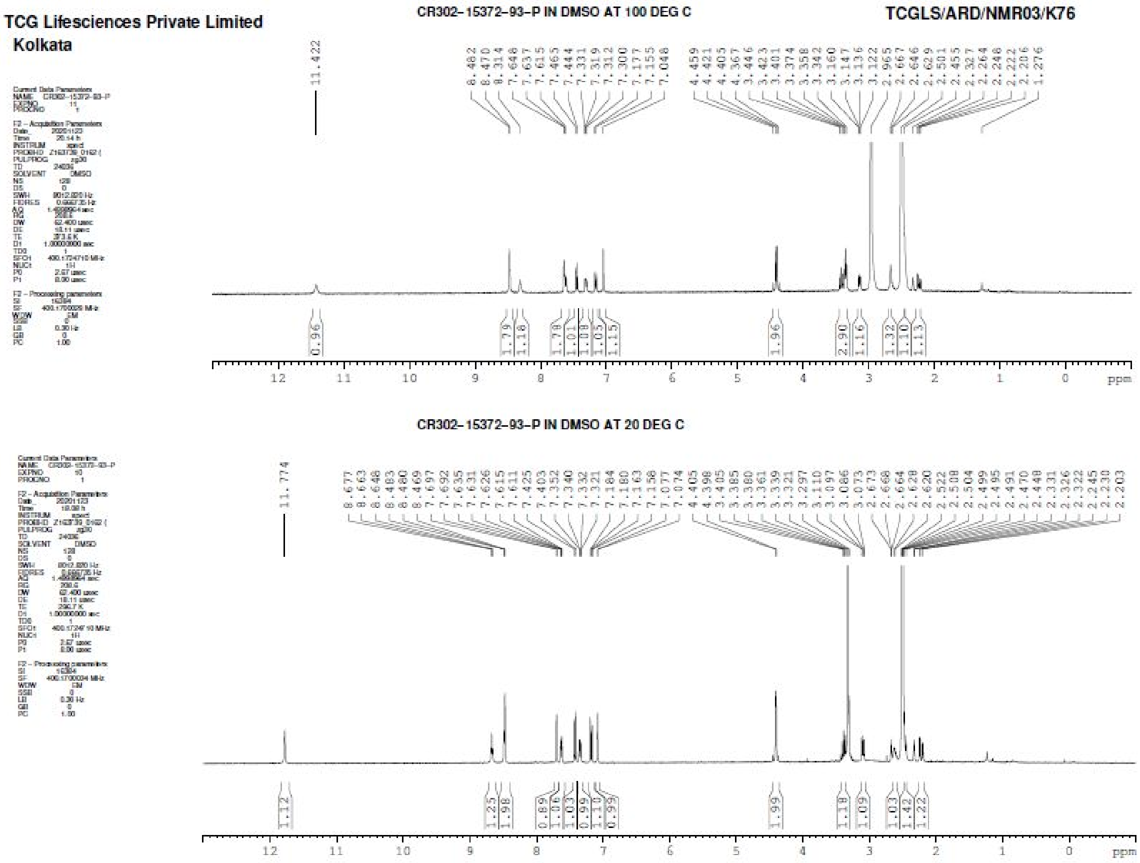

^{1}H NMR (20 °C and 100 °C) (400 MHz, DMSO-d_6)-5s

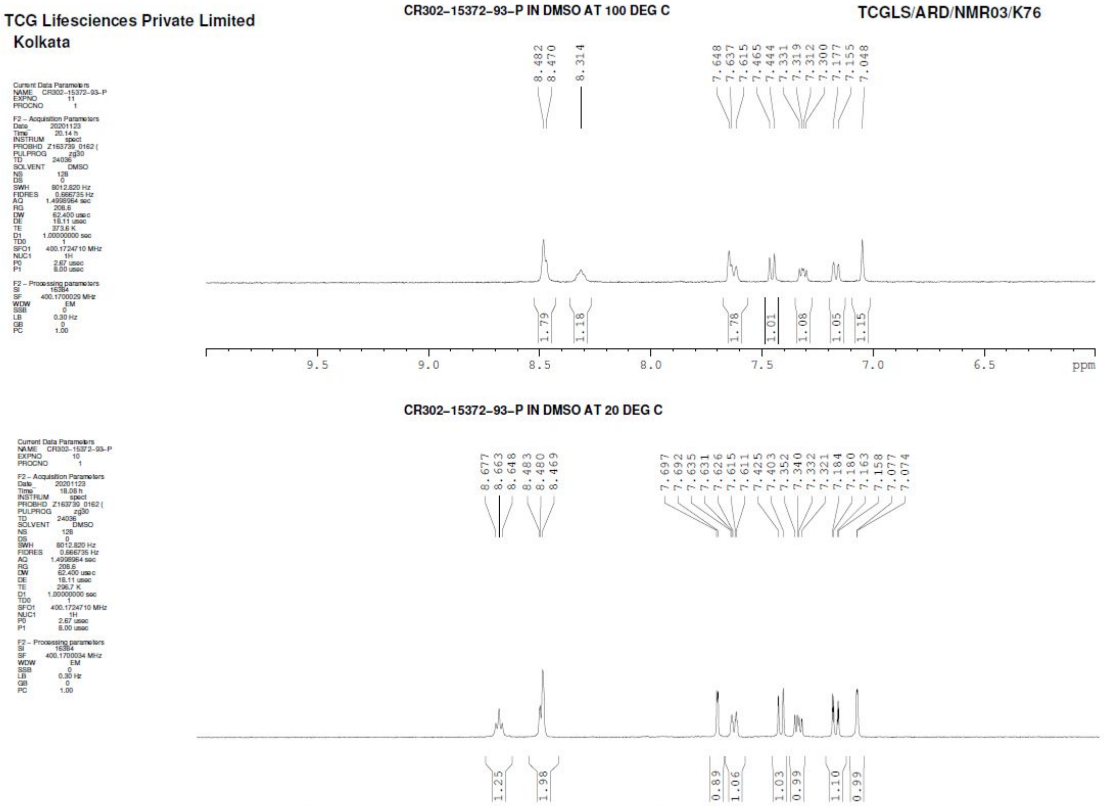

^{1}H NMR (20 °C and 100 °C) (400 MHz, DMSO-d_6)-5s

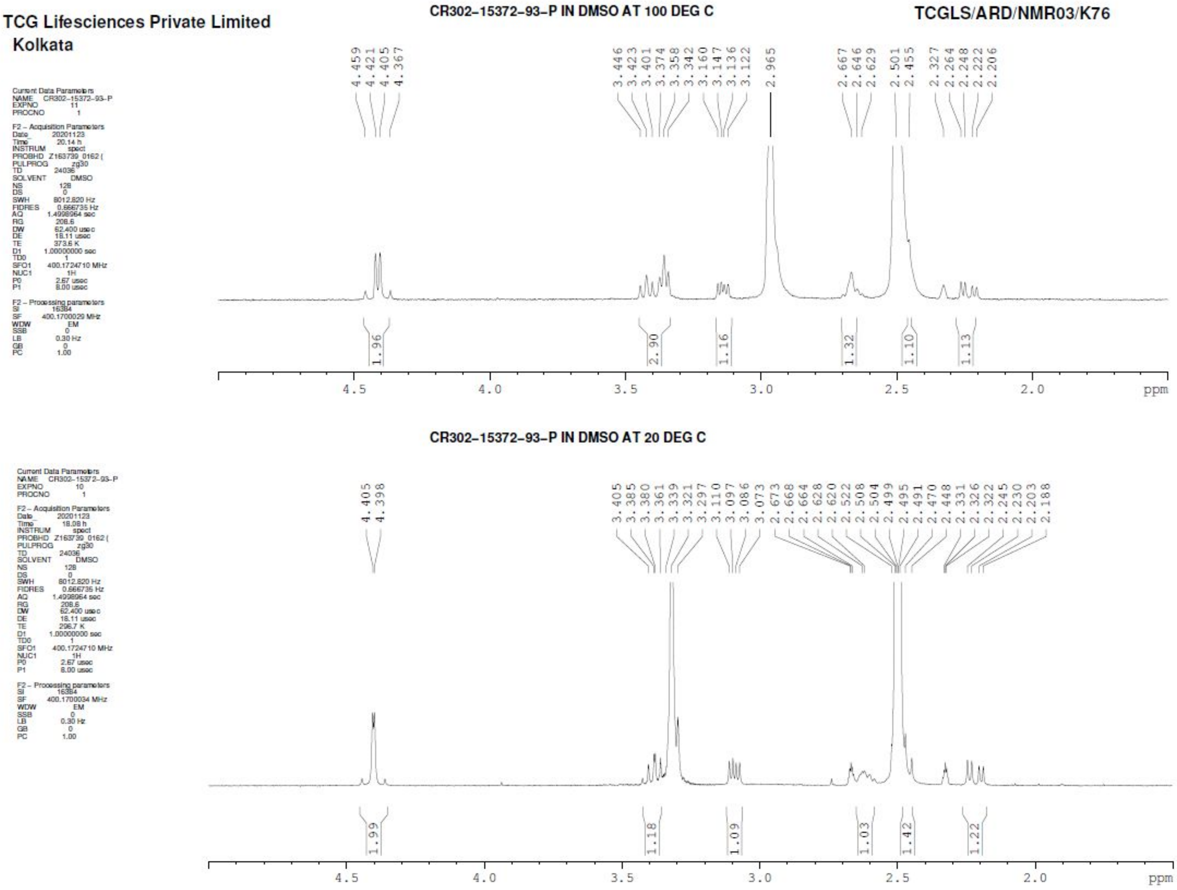

^{1}H NMR (20 °C and 100 °C) (400 MHz, DMSO-d_6)-5s

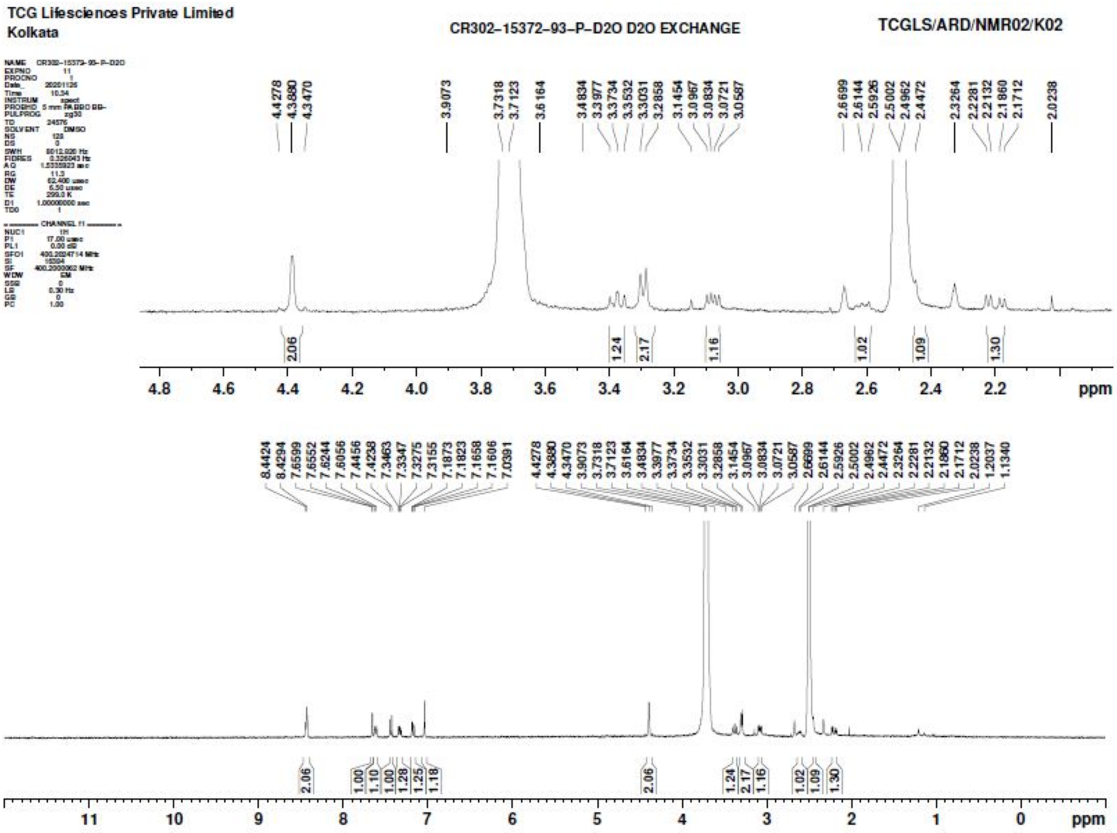

^{1}H NMR (400 MHz, DMSO-d_6- D_2O-exchange)-5r

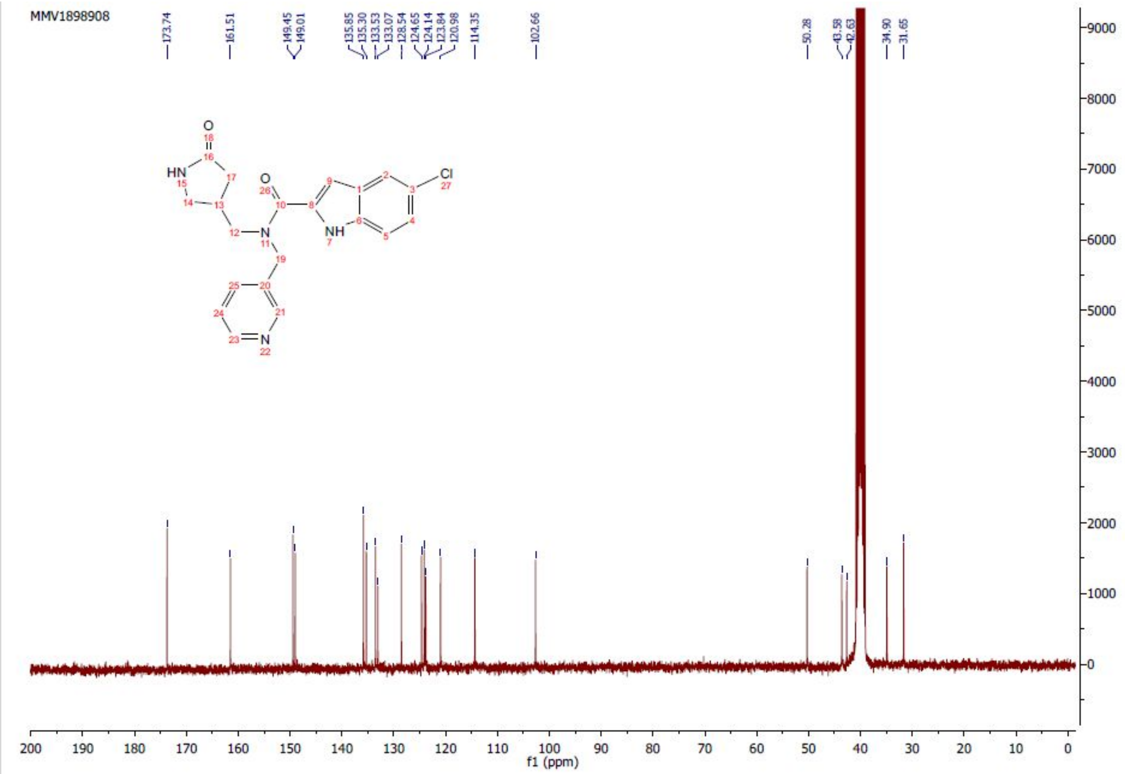

^{13}C NMR (75 MHz, DMSO-d$_6$)-5s

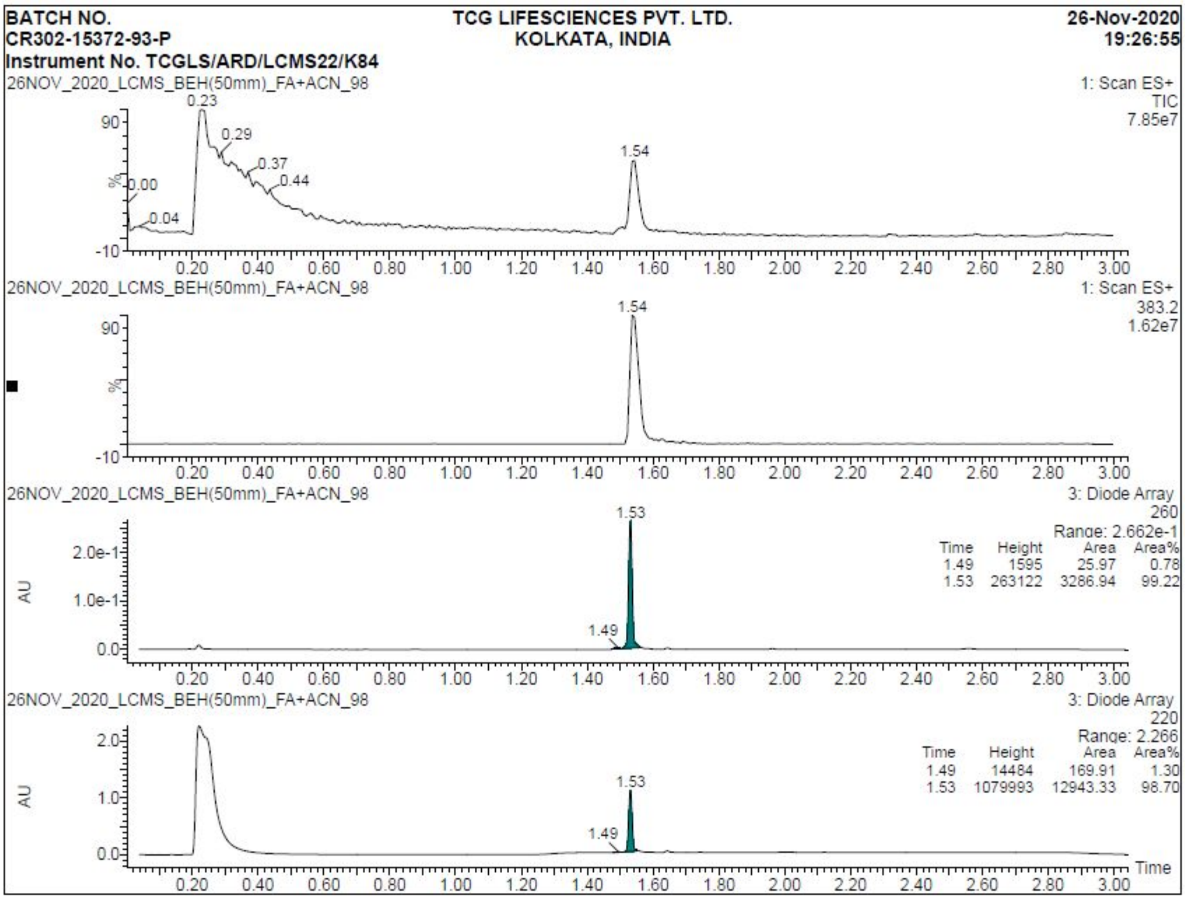

LCMS-5s

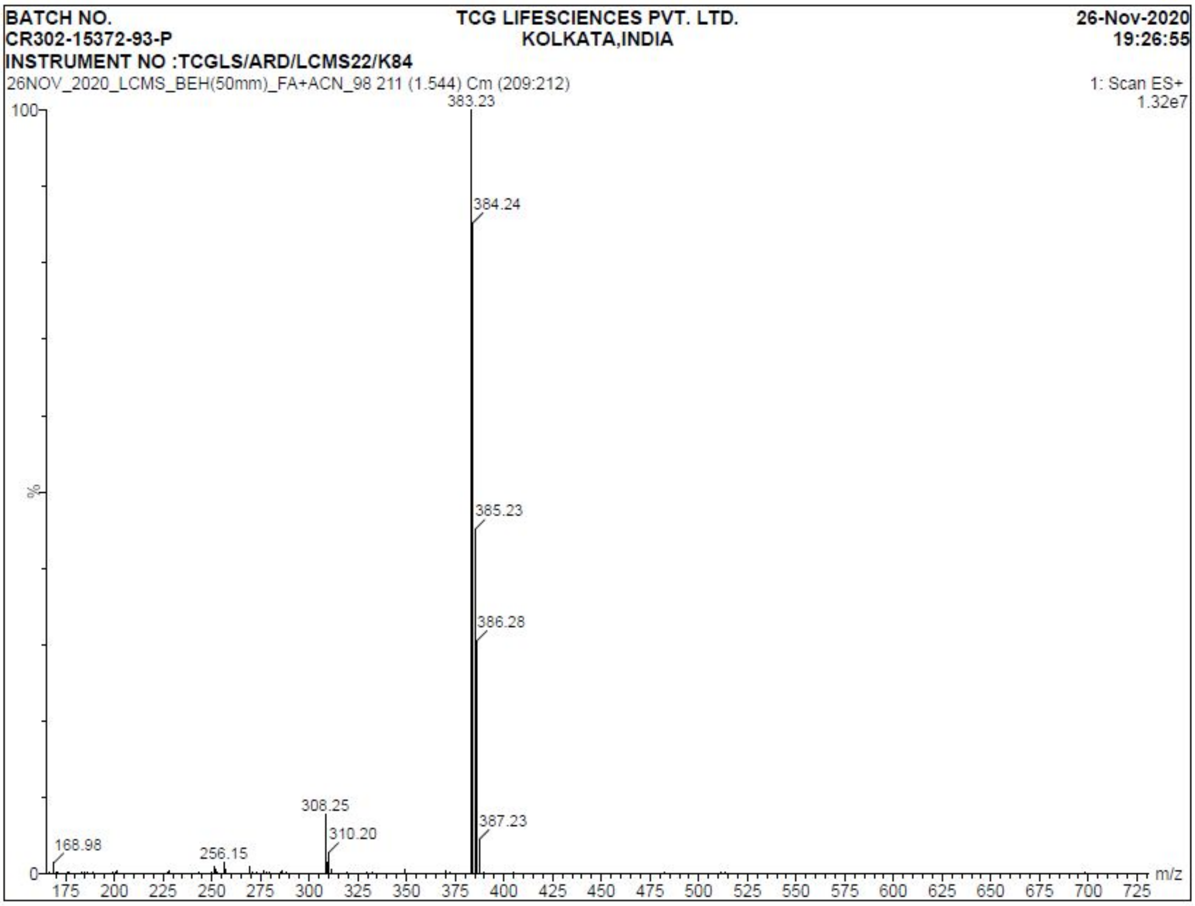

LCMS-5s

	Resultados HRMS	
	Responsável:	Diego C Andrade
	Data:	06/10/22

Laboratório Institucional de Espectrometria de Massas

Prof. Dr. Luiz Carlos Dias
Aluno Anwar Shamim
Amostra MMV908

Espectro completo de 50 a 750 m/z em modo positivo

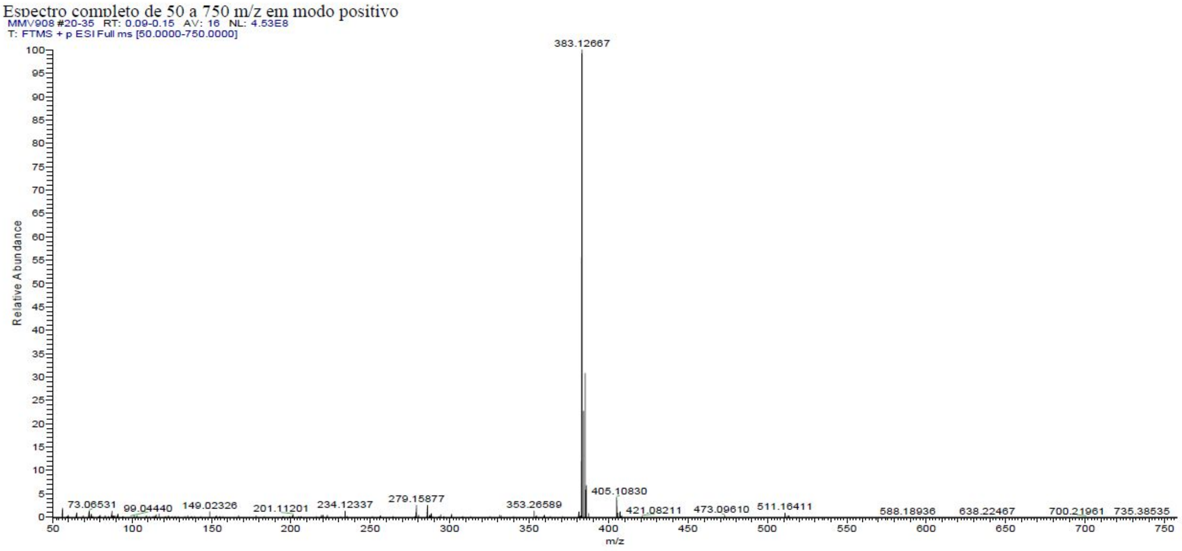

HRMS-5s

Resultados HRMS	
Responsável:	Diego C Andrade
Data:	06/10/22
**Laboratório Institucional de Espectrometria de Massas**	

Comparação entre espectro real (superior) e simulado (inferior) para C20H19ClN4O2H+

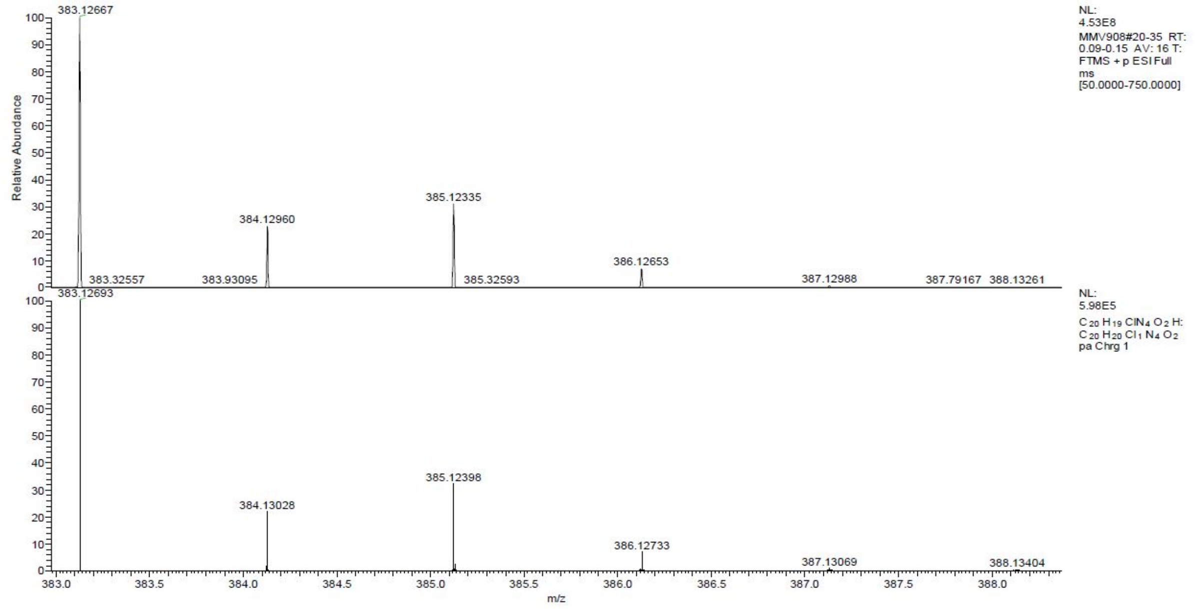

HRMS-5s

5-chloro-N-(1-methylpiperidin-4-yl)-N-(pyridin-3-ylmethyl)-1H-indole-2-carboxamide (5t).

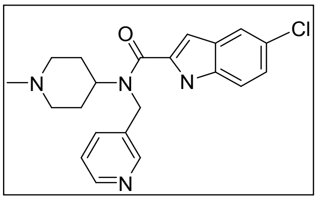

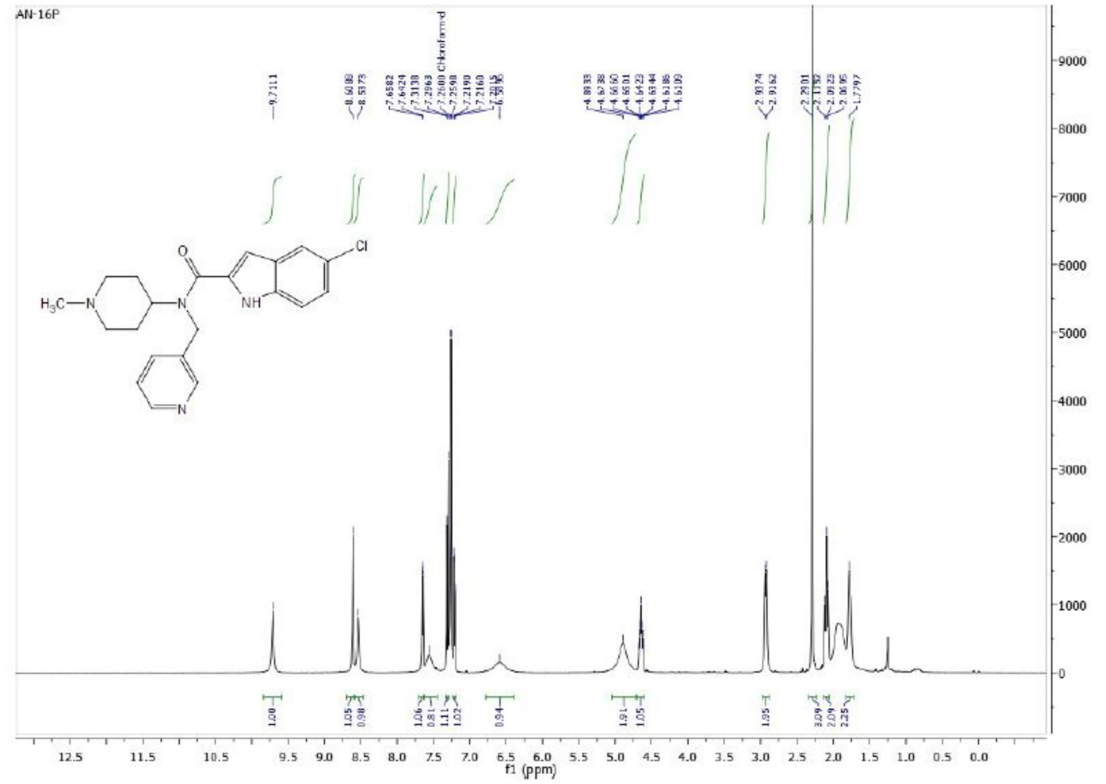

^{1}H NMR (500 MHz, $CDCl_3$)-5t.

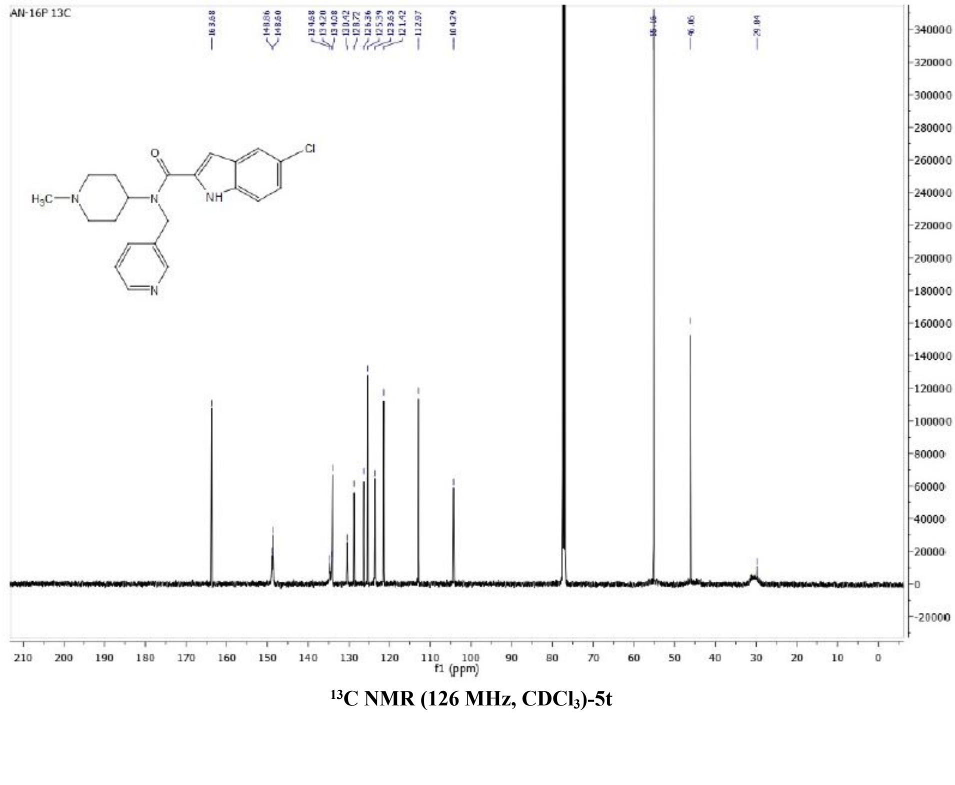

^{13}C NMR (126 MHz, $CDCl_3$)-5t

Relatório de atividade:	Amostras Alta Resolução
Responsável:	Diego C Andrade
Data:10/09/2021	

Laboratório Institucional de Espectrometria de Massas

Amostra: AN-16A
Prof. Dr. Luiz Carlos Dias
Aluno Anees Ahmad

Espectro completo de 50 a 750 m/z em polaridade positiva

AN-16A #20-96 RT: 0.07-0.30 AV: 77 NL: 9.50E8
T: FTMS + p ESI Full ms [50.0000-750.0000]

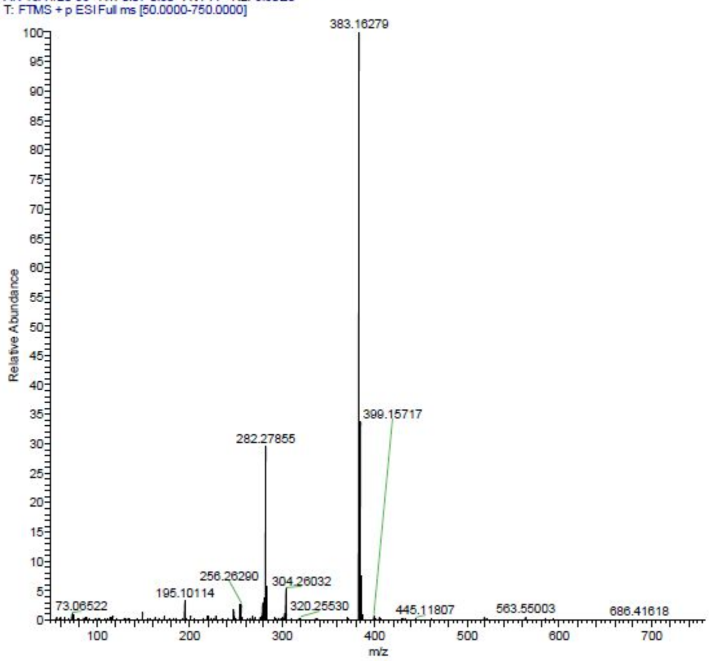

HRMS-5t

Relatório de atividade:	Amostras Alta Resolução
Responsável:	Diego C Andrade
Data:10/09/2021	

Laboratório Institucional de Espectrometria de Massas

Comparação entre espectro real (superior) e simulado (inferior) para $C_{21}H_{23}ClN_4OH^+$

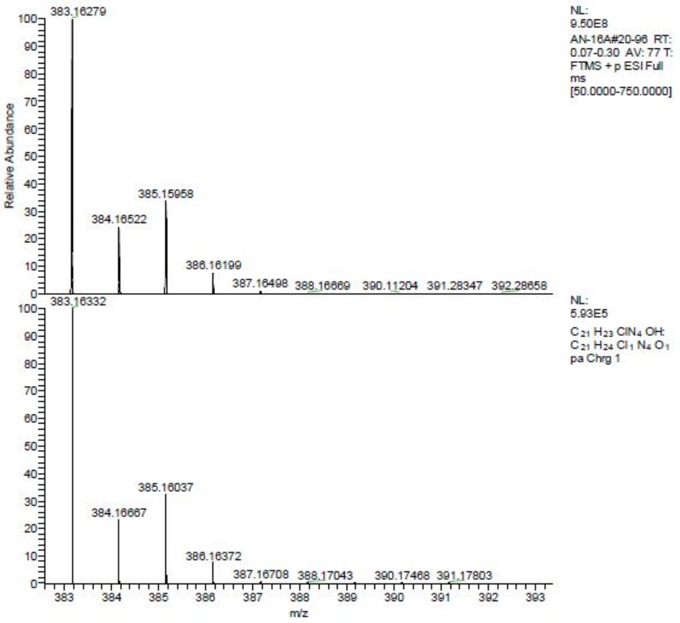

HRMS-5t

5-chloro-N-(pyridin-3-ylmethyl)-N-(pyrrolidin-3-ylmethyl)-1H-indole-2-carboxamide (6a).

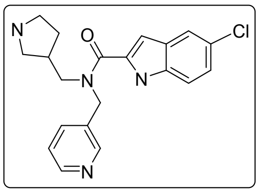

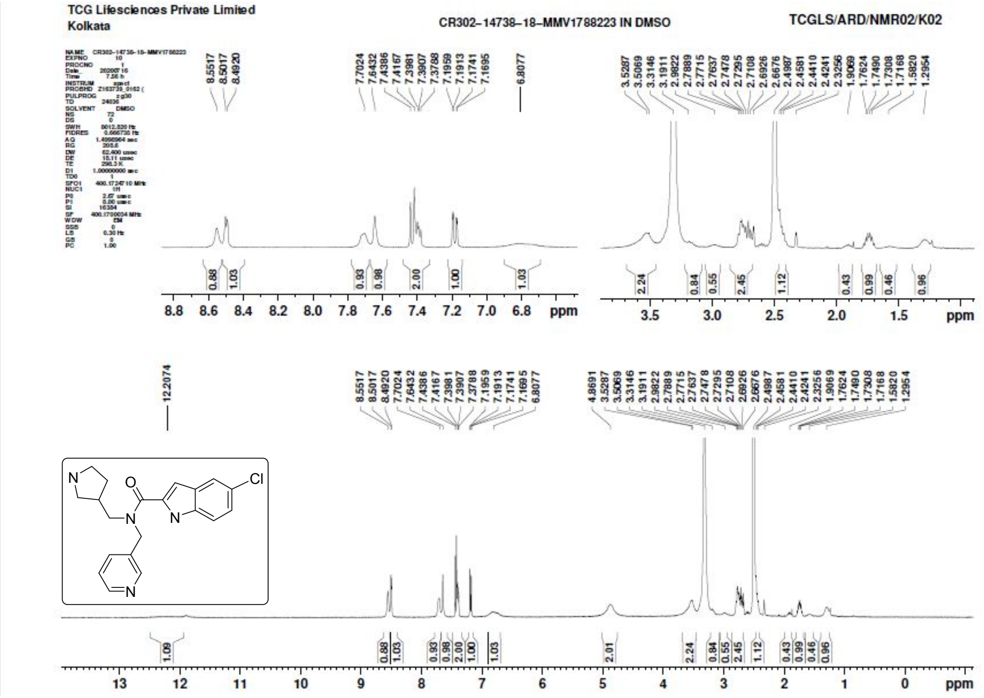

^{1}H NMR (400 MHz, DMSO-d_6)-6a

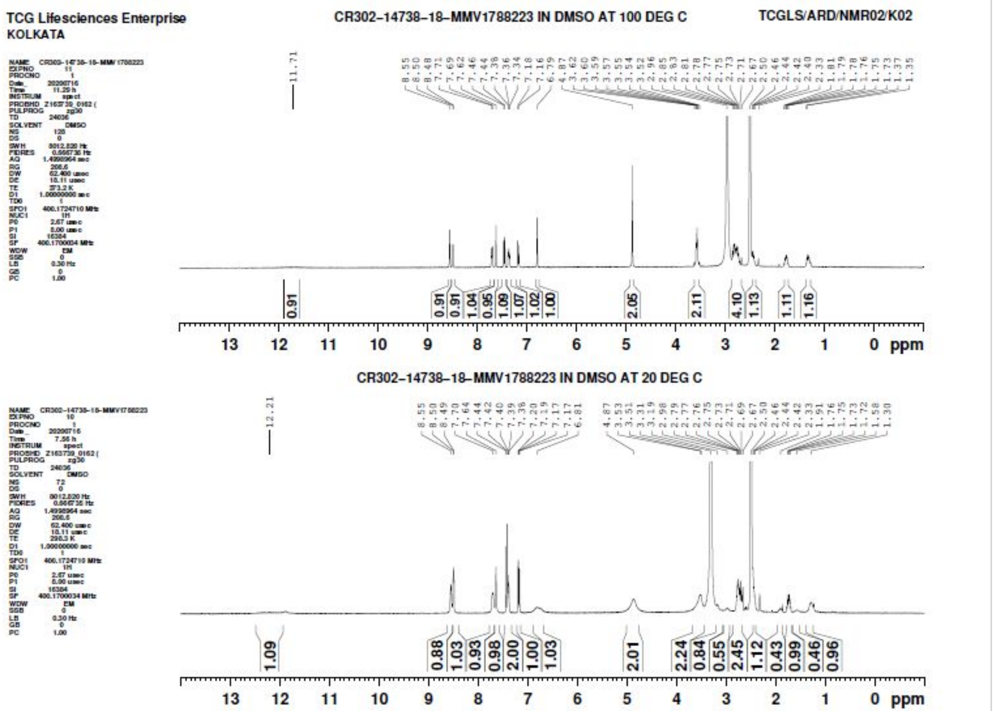

^{1}H NMR (20 °C and 100 °C) (400 MHz, DMSO-d_6)-6a

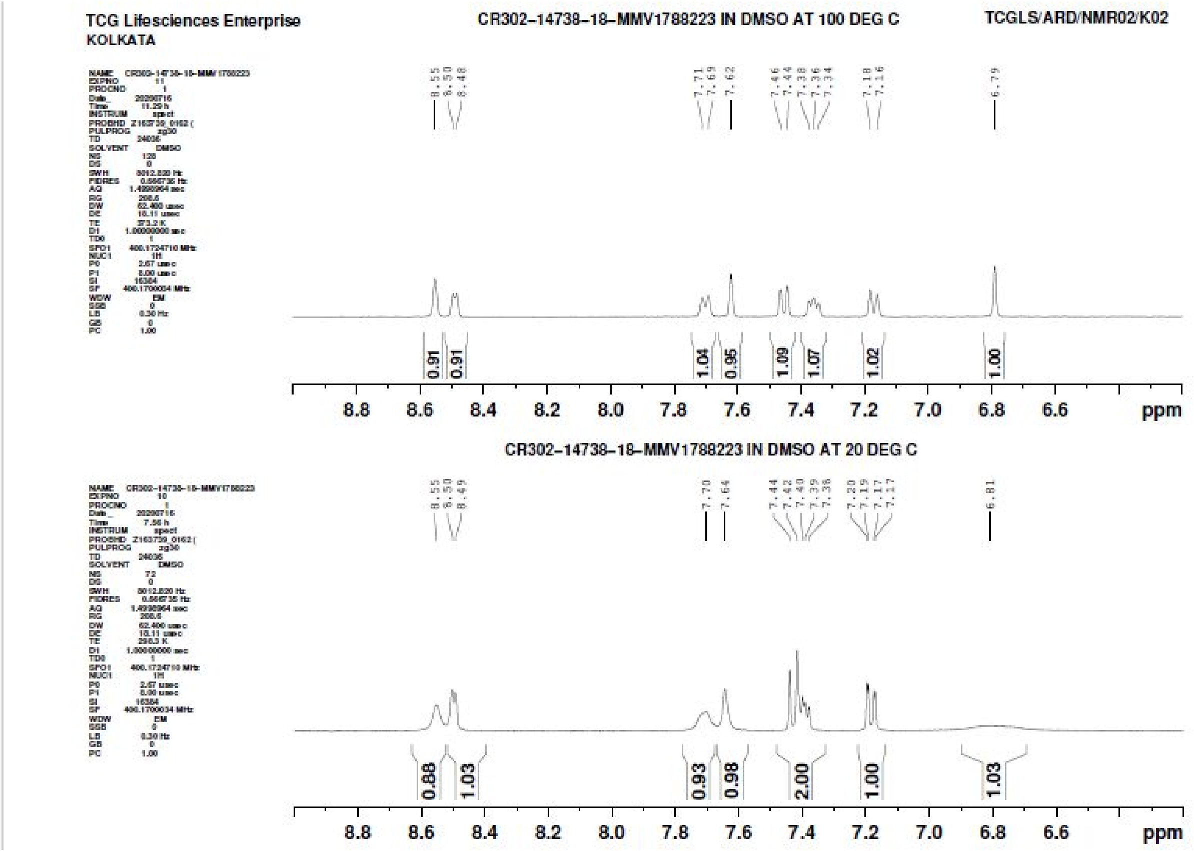

^{1}H NMR (20 °C and 100 °C) (400 MHz, DMSO-d_6)-6a

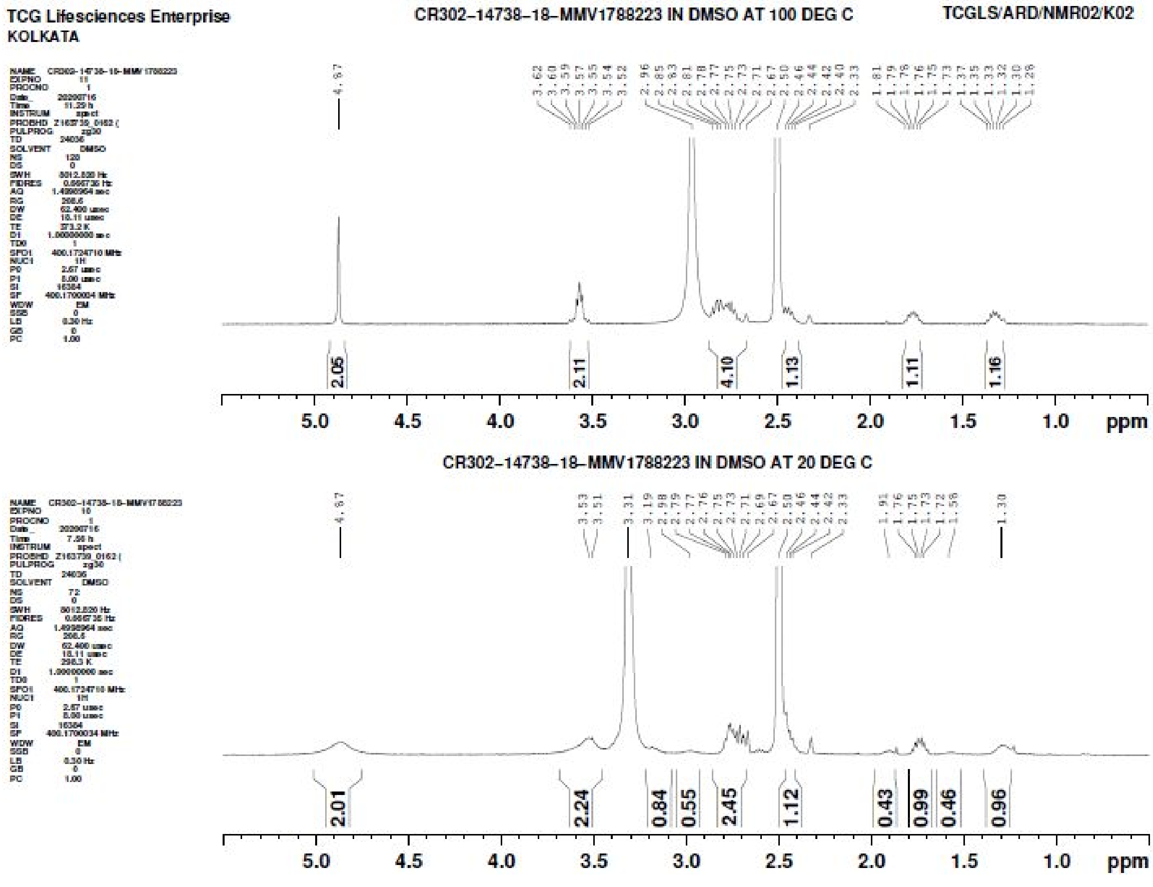

^{1}H NMR (20 °C and 100 °C) (400 MHz, DMSO-d_6)-6a

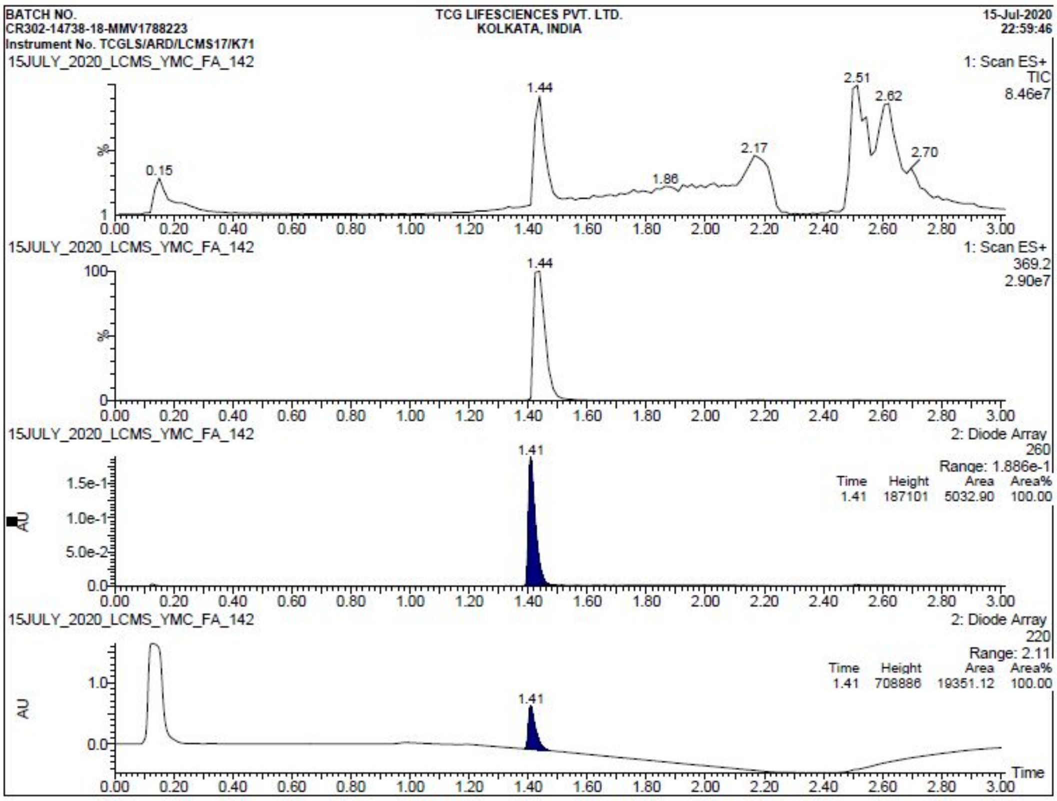
BATCH NO.
CR302-14738-18-MMV1788223
Instrument No. TCGLS/ARD/LCMS17/K71
TCG LIFESCIENCES PVT. LTD.
KOLKATA, INDIA
15-Jul-2020
22:59:46
15JULY_2020_LCMS_YMC_FA_142
1: Scan ES+
TIC
8.46e7
369.2
2.90e7
2: Diode Array
260
Range: 1.886e-1
Time Height Area Area%
1.41 187101 5032.90 100.00
220
Range: 2.11
1.41 708886 19351.12 100.00
Time

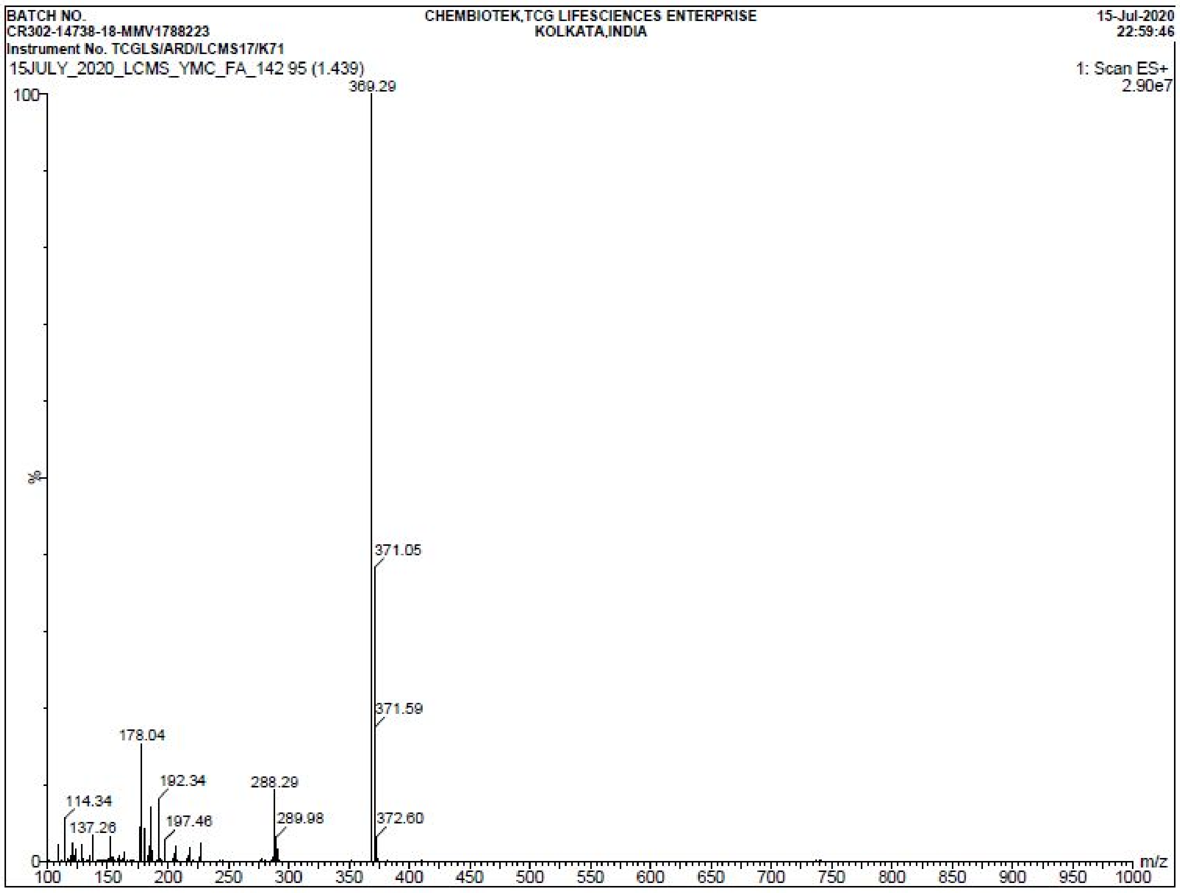
BATCH NO.
CR302-14738-18-MMV1788223
Instrument No. TCGLS/ARD/LCMS17/K71
CHEMBIOTEK,TCG LIFESCIENCES ENTERPRISE
KOLKATA,INDIA
15-Jul-2020
22:59:46
15JULY_2020_LCMS_YMC_FA_142 95 (1.439)
1: Scan ES+
2.90e7
369.29
371.05
371.59
372.60
178.04
192.34
288.29
289.98
114.34
137.26
197.46
m/z

LCMS-6a

Resultados HRMS	
Responsável:	Diego C Andrade
Data:	06/10/22
**Laboratório Institucional de Espectrometria de Massas**	

Prof. Dr. Luiz Carlos Dias
Aluno Anwar Shamim
Amostra MMV223

Espectro completo de 50 a 750 m/z em modo positivo

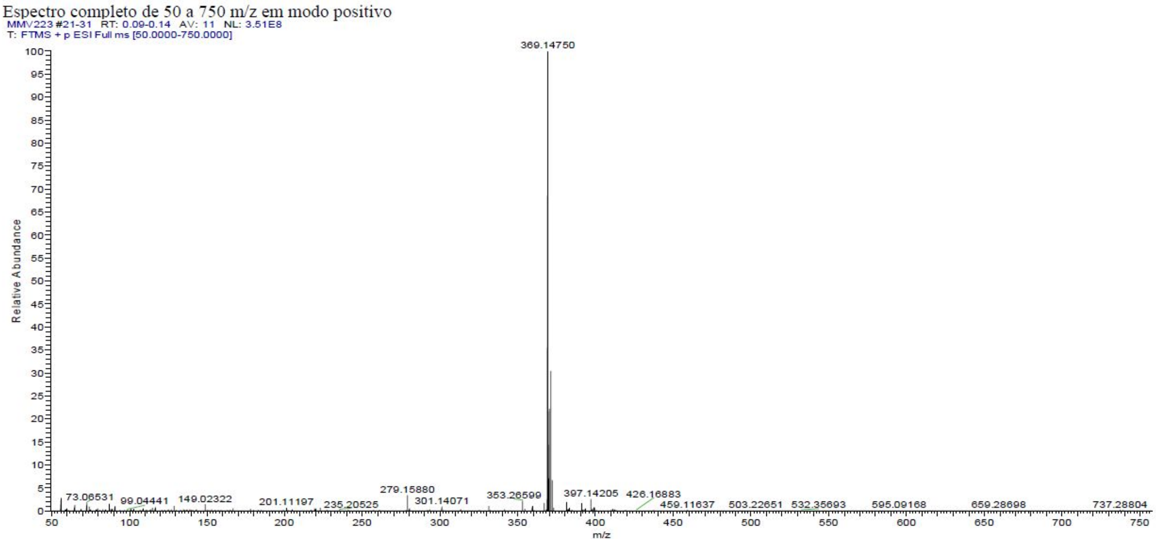

HRMS-6a

Resultados HRMS	
Responsável:	Diego C Andrade
Data:	06/10/22
**Laboratório Institucional de Espectrometria de Massas**	

Comparação entre espectro real (superior) e simulado (inferior) para C20H21ClN4OH+

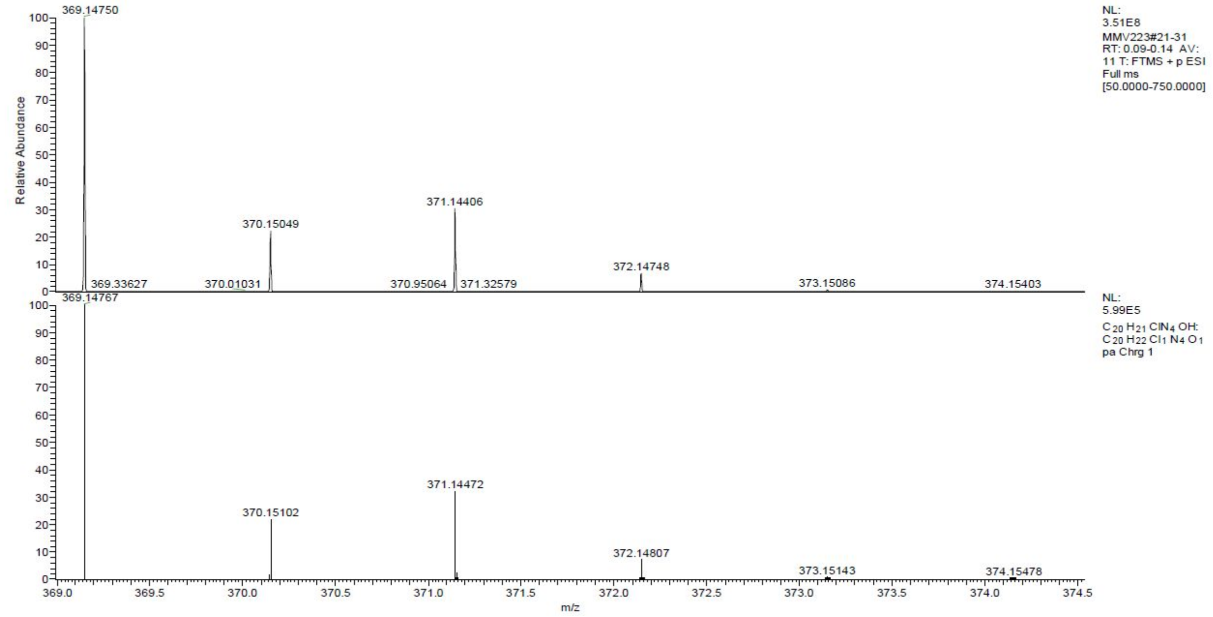

HRMS-6a

5-chloro-N-(3-pyridylmethyl)-N-[[(3R)-pyrrolidin-3-yl]methyl]-1H-indole-2-carboxamide(6a(R)):

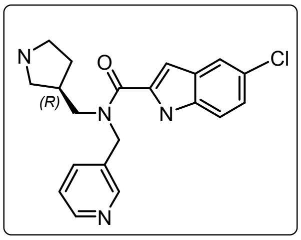

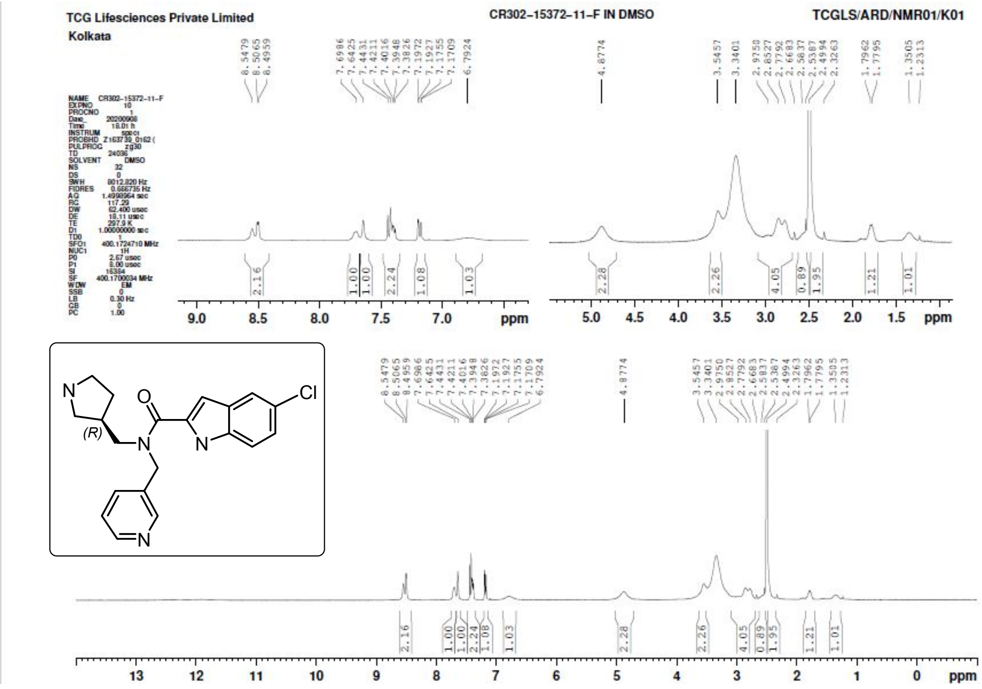

^{1}H NMR (400 MHz, DMSO-d$_6$)-6a(R)

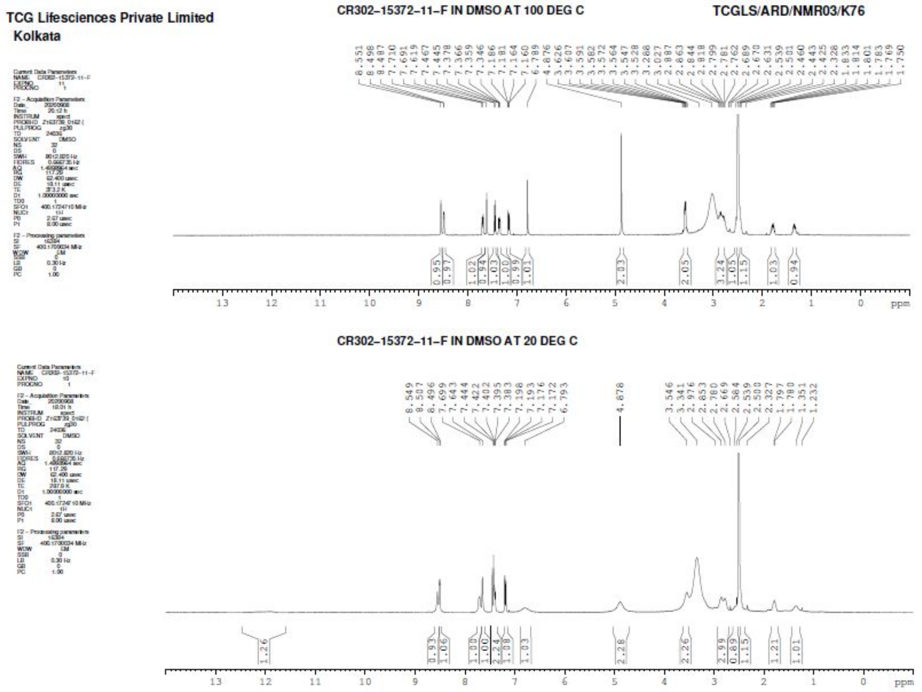

^{1}H NMR (20 °C and 100 °C) (400 MHz, DMSO-d$_6$)-6a(R)

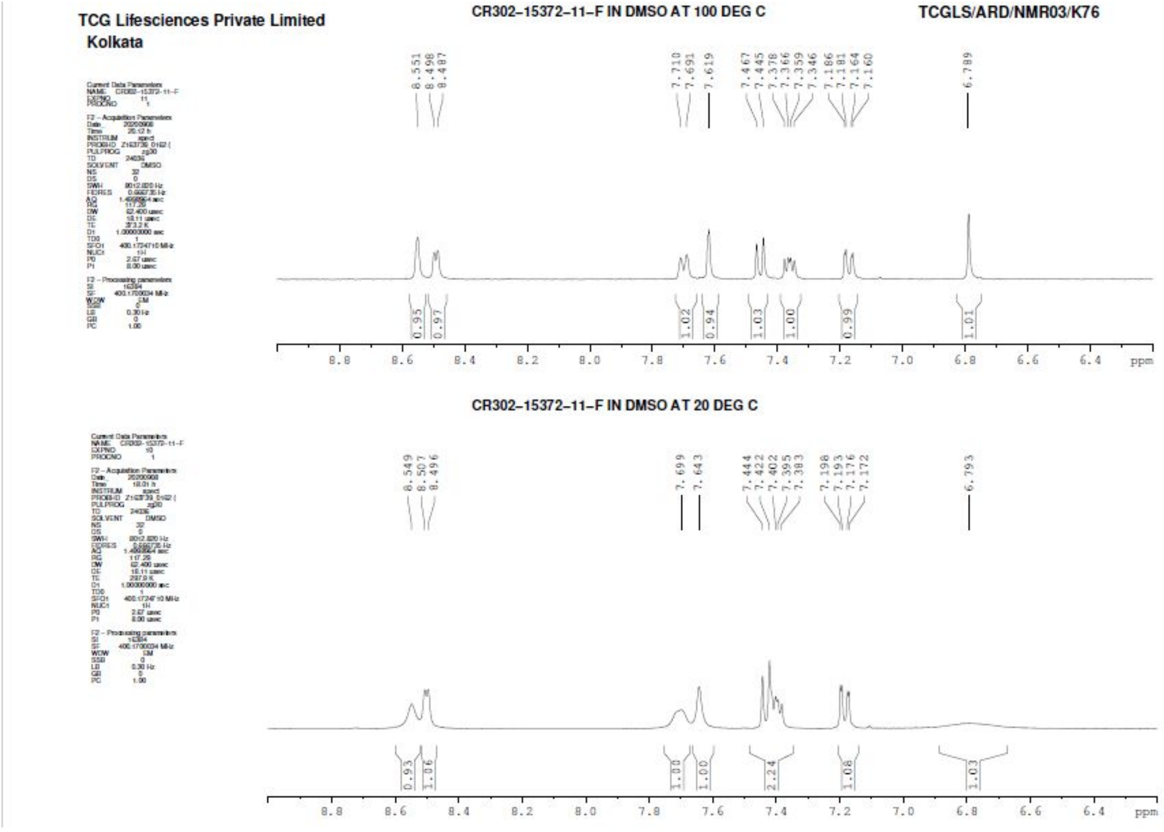

^{1}H NMR (20 °C and 100 °C) (400 MHz, DMSO-d_6)-6a(R)

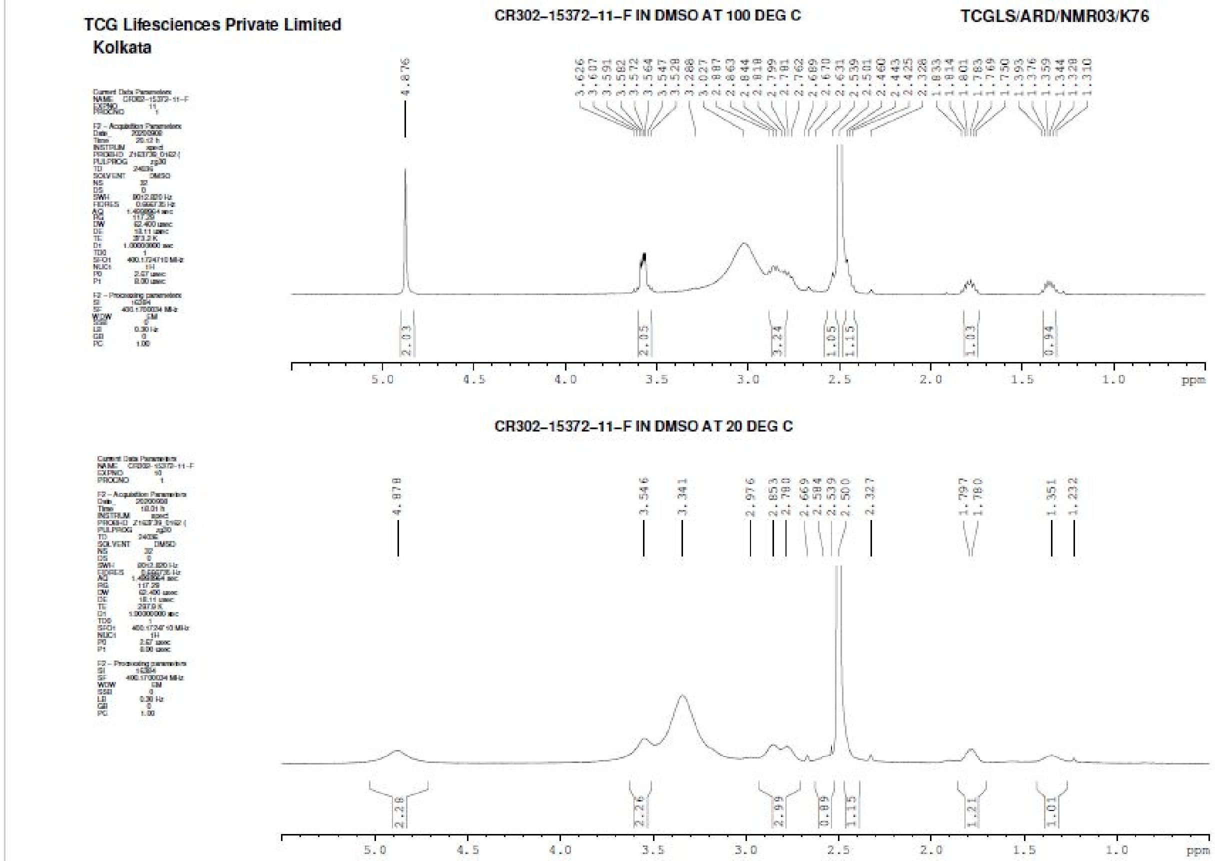

^{1}H NMR (20 °C and 100 °C) (400 MHz, DMSO-d_6)-6a(R)

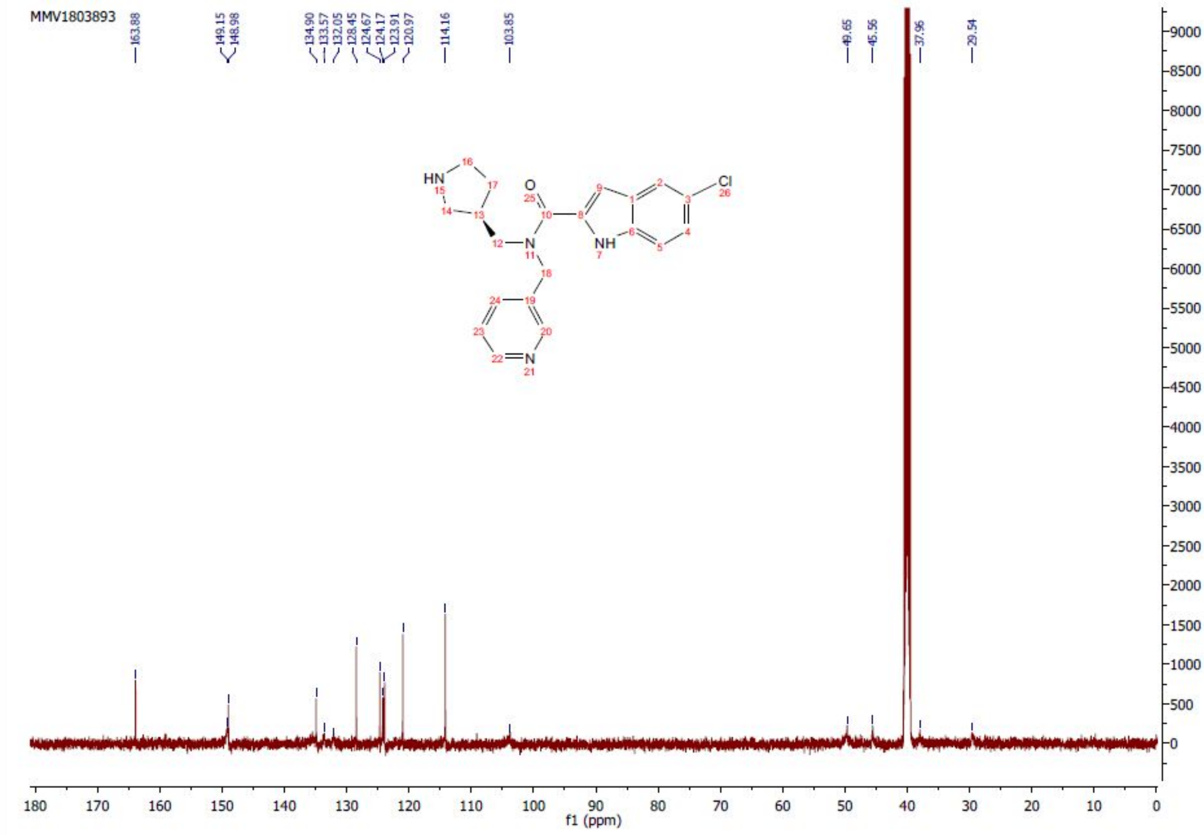

^{13}C NMR (125 MHz, DMSO-d_6)-6a(R)

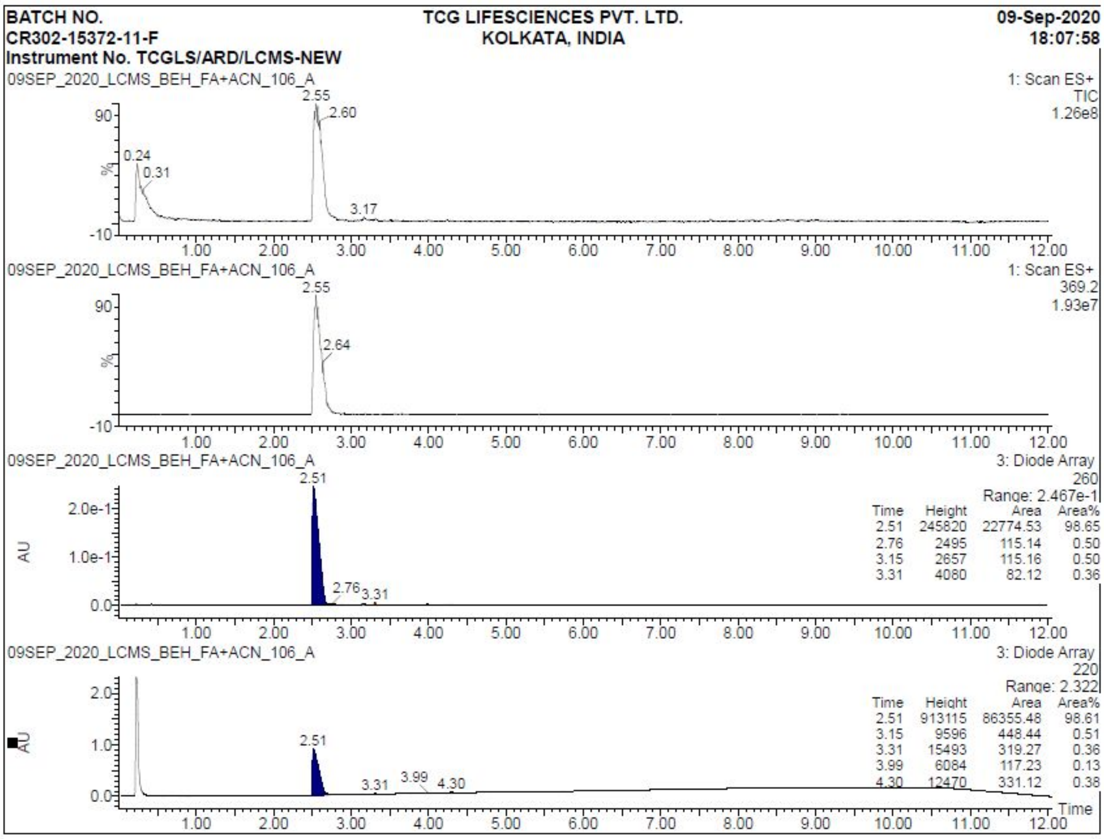

LCMS-6a(R)

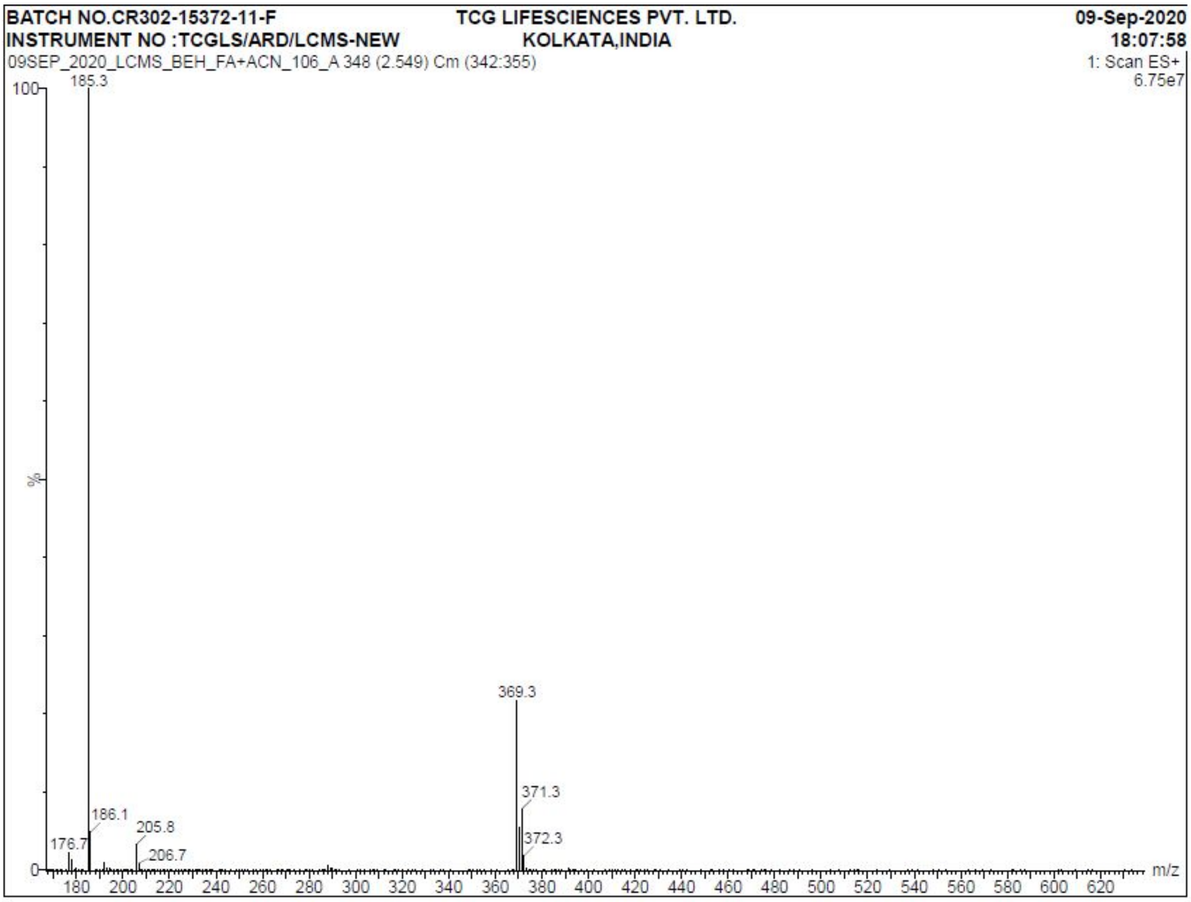

LCMS-6a(R)

Resultados HRMS	
Responsável:	Diego C Andrade
Data:	03/11/22

Laboratório Institucional de Espectrometria de Massas

Prof. Dr. Luiz Carlos Dias
Aluna Mariana Ferrer
Amostra MMV893

Espectro completo de 80 a 750 m/z em modo positivo

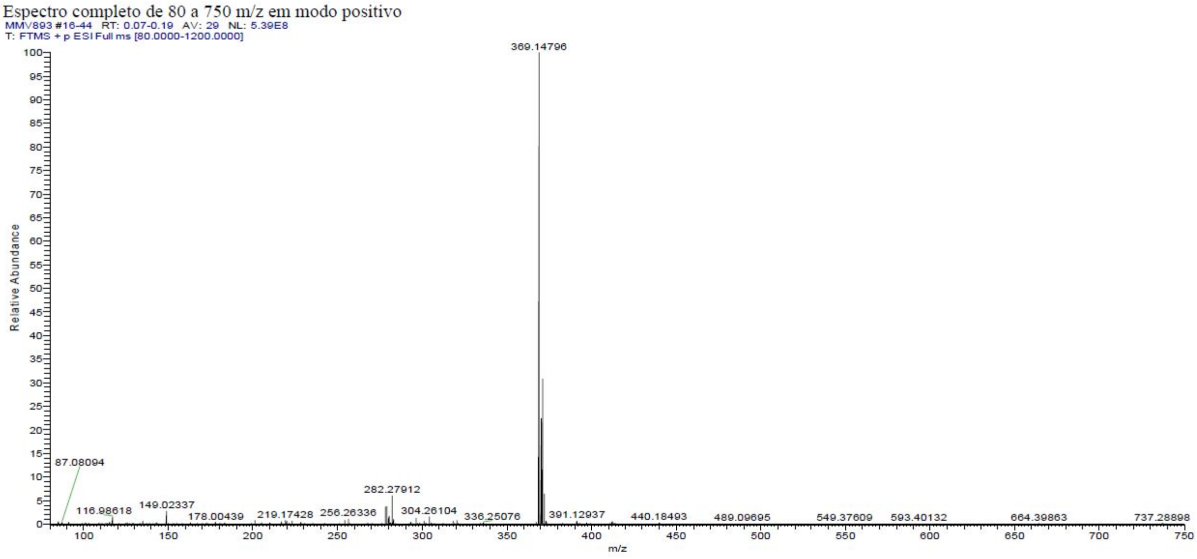

HRMS-6a(R)

Resultados HRMS	
Responsável:	Diego C Andrade
Data:	03/11/22
**Laboratório Institucional de Espectrometria de Massas**	

Comparação entre espectro real (superior) e simulado (inferior) para C20H21ClN4OH+

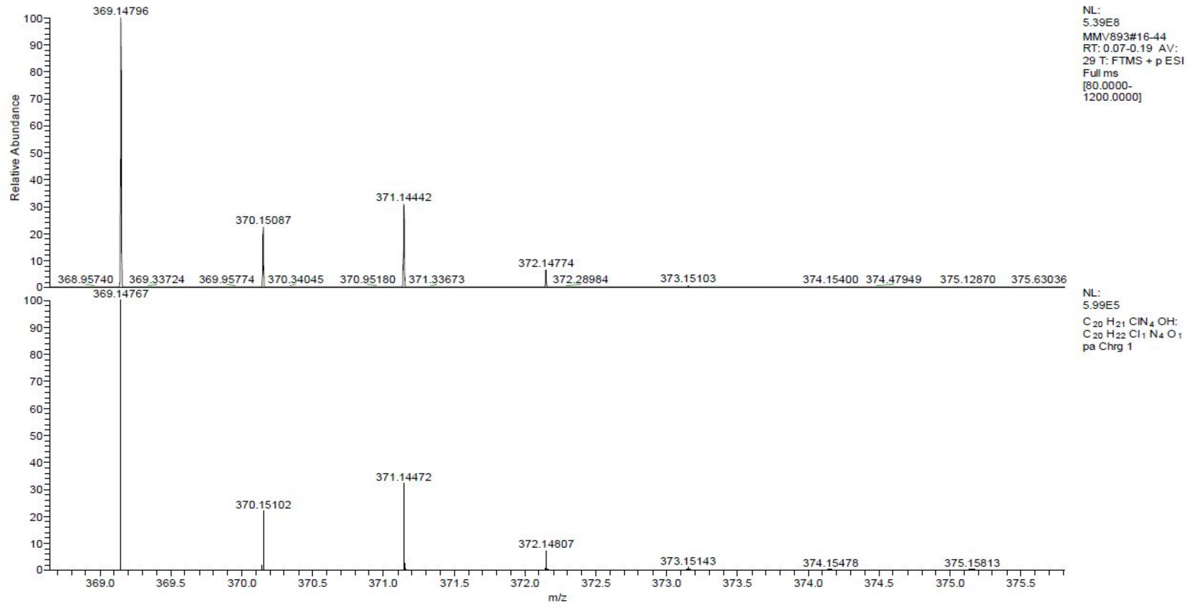

HRMS-6a(R)

5-chloro-N-(3-pyridylmethyl)-N-[[(3S)-pyrrolidin-3-yl]methyl]-1H-indole-2-carboxamide(6a(S)):

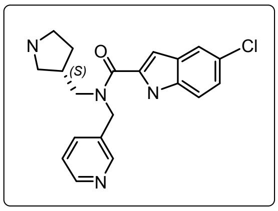

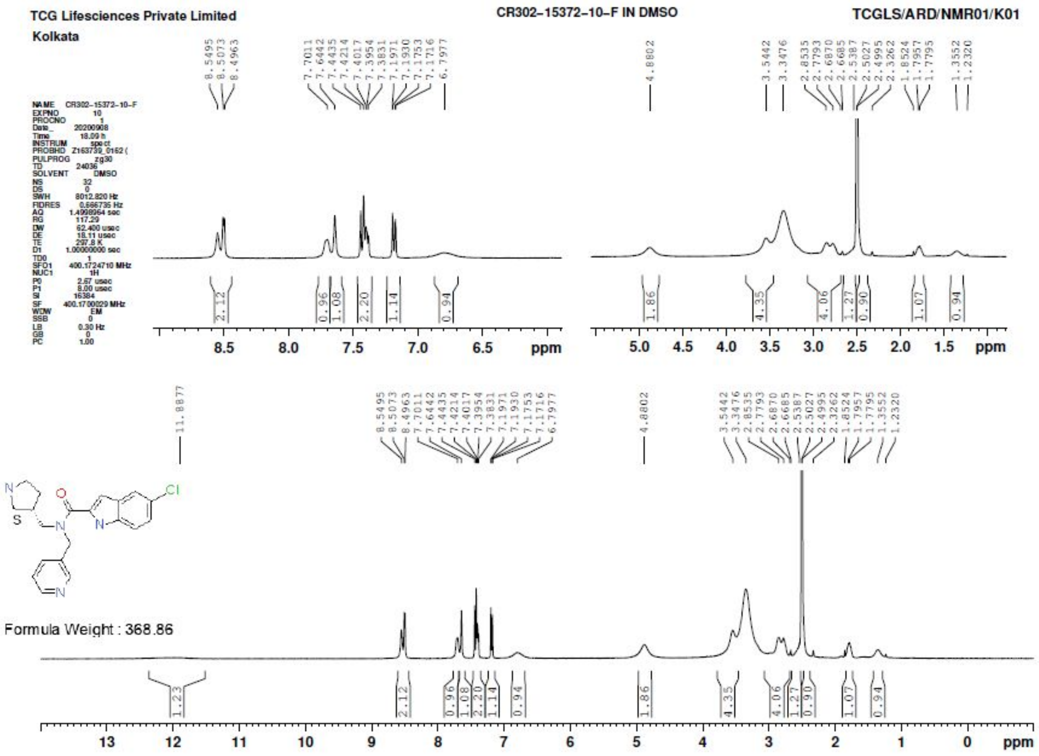

^{1}H NMR (400 MHz, DMSO-d$_6$)-6a(S)

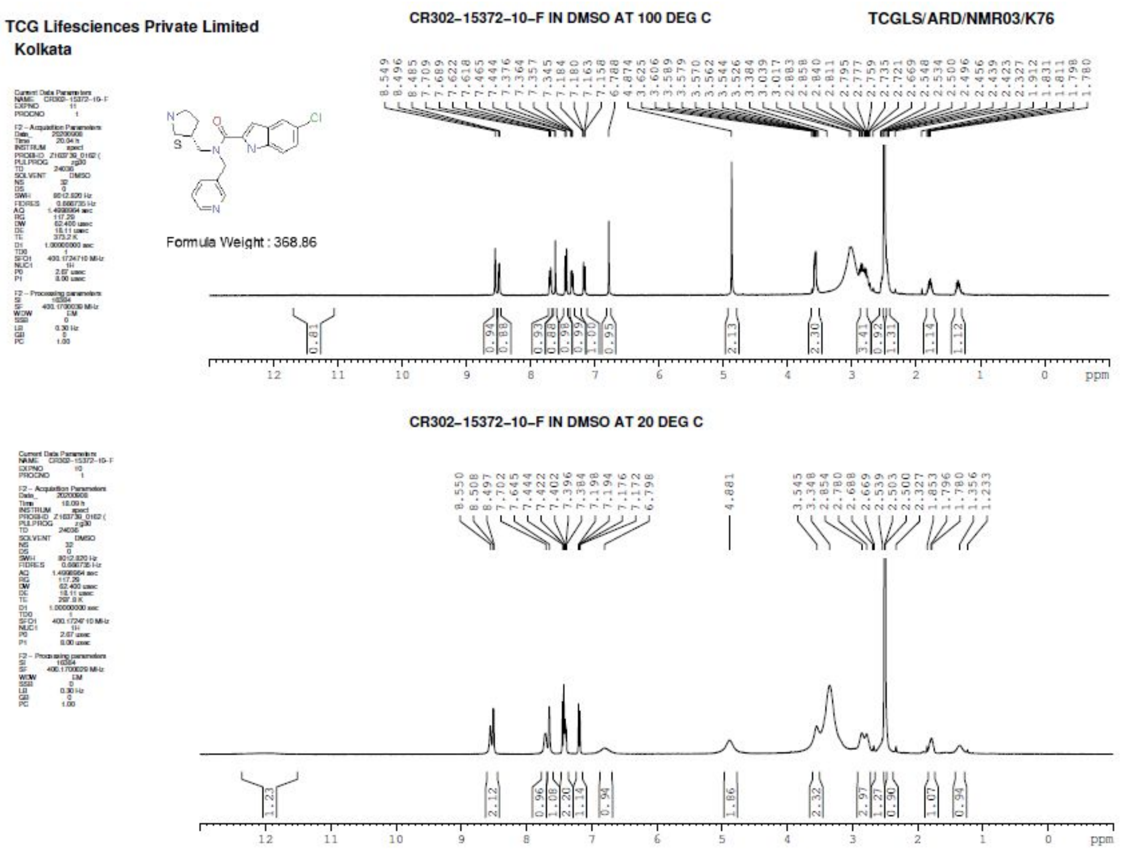

¹H NMR (20 °C and 100 °C) (400 MHz, DMSO-d_6)-6a(s)

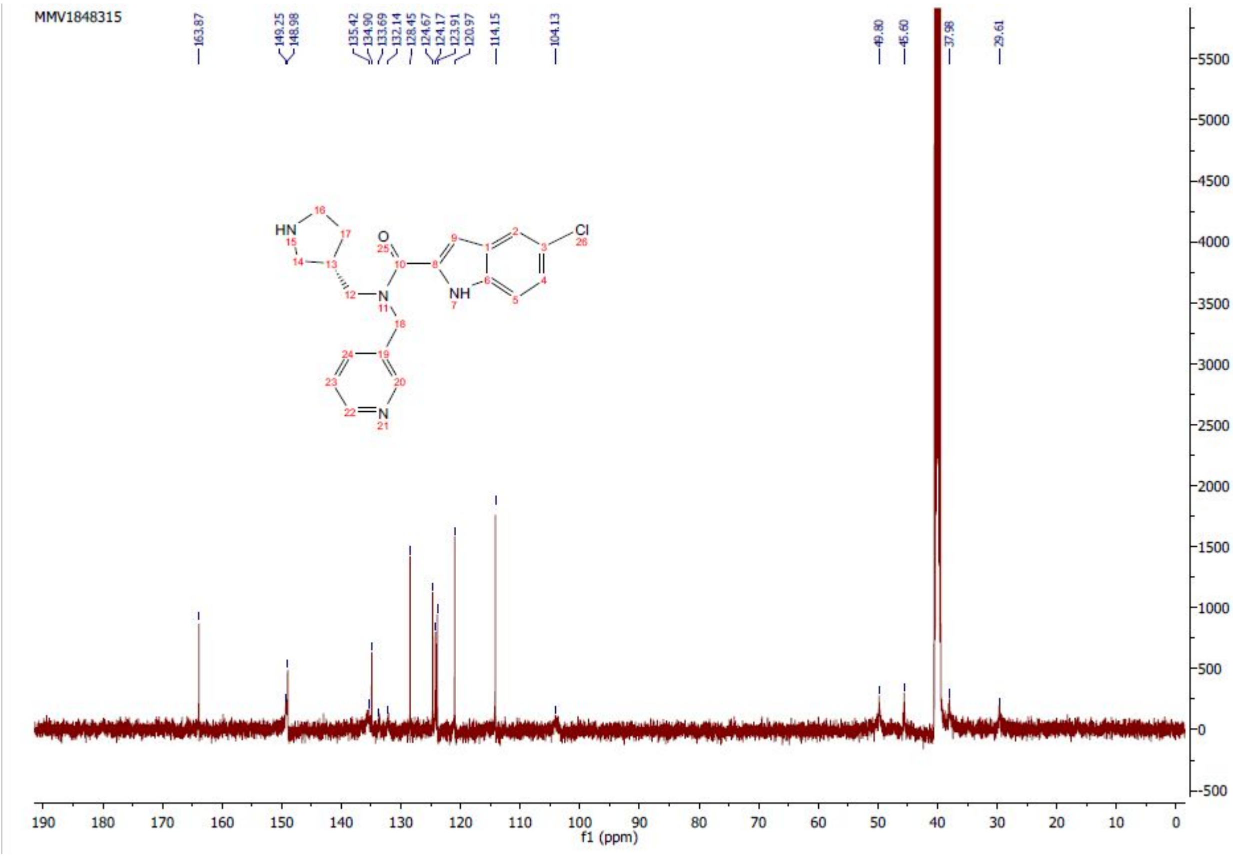

¹³C NMR (125 MHz, DMSO-d_6)-6a(S)

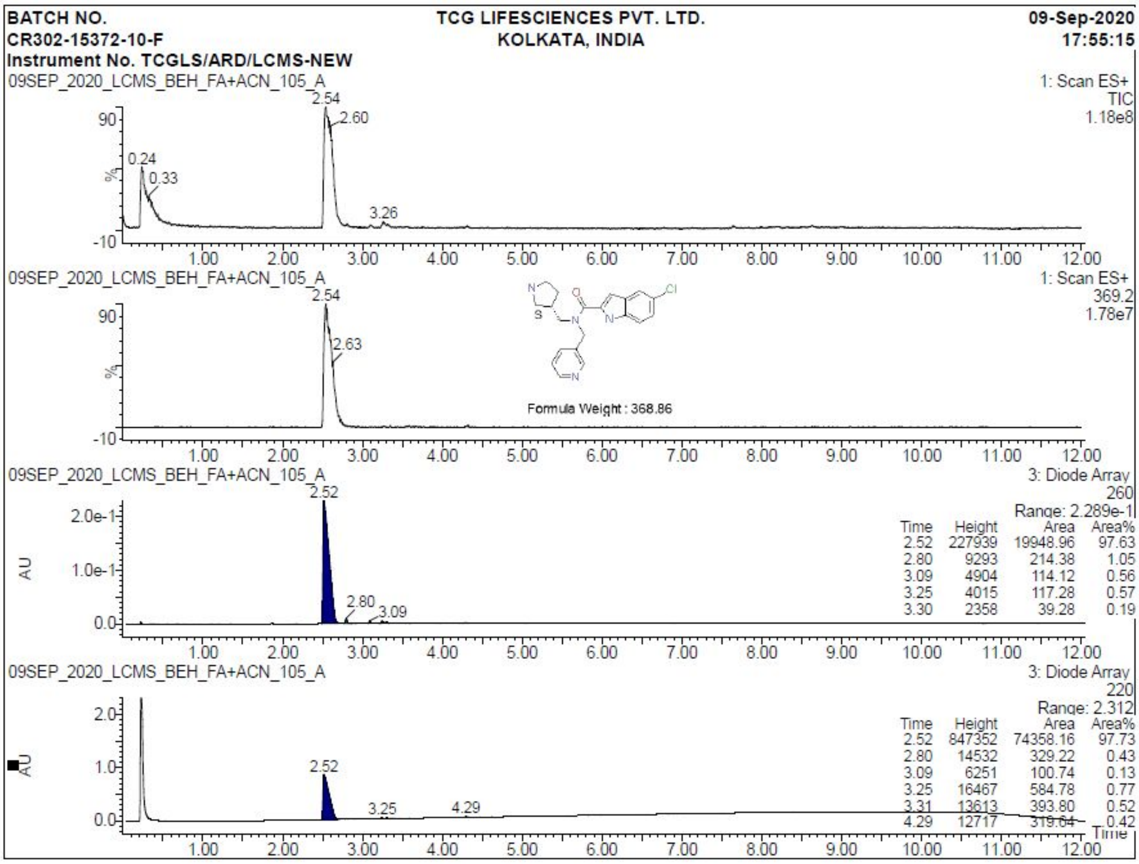
BATCH NO.
CR302-15372-10-F
Instrument No. TCGLS/ARD/LCMS-NEW
TCG LIFESCIENCES PVT. LTD.
KOLKATA, INDIA
09-Sep-2020
17:55:15
09SEP_2020_LCMS_BEH_FA+ACN_105_A
1: Scan ES+
TIC
1.18e8
1: Scan ES+
369.2
1.78e7
Formula Weight : 368.86
3: Diode Array
260
Range: 2.289e-1
Time Height Area Area%
2.52 227939 19948.96 97.63
2.80 9293 214.38 1.05
3.09 4904 114.12 0.56
3.25 4015 117.28 0.57
3.30 2358 39.28 0.19
3: Diode Array
220
Range: 2.312
Time Height Area Area%
2.52 847352 74358.16 97.73
2.80 14532 329.22 0.43
3.09 6251 100.74 0.13
3.25 16467 584.78 0.77
3.31 13613 393.80 0.52
4.29 12717 319.64 0.42
Time

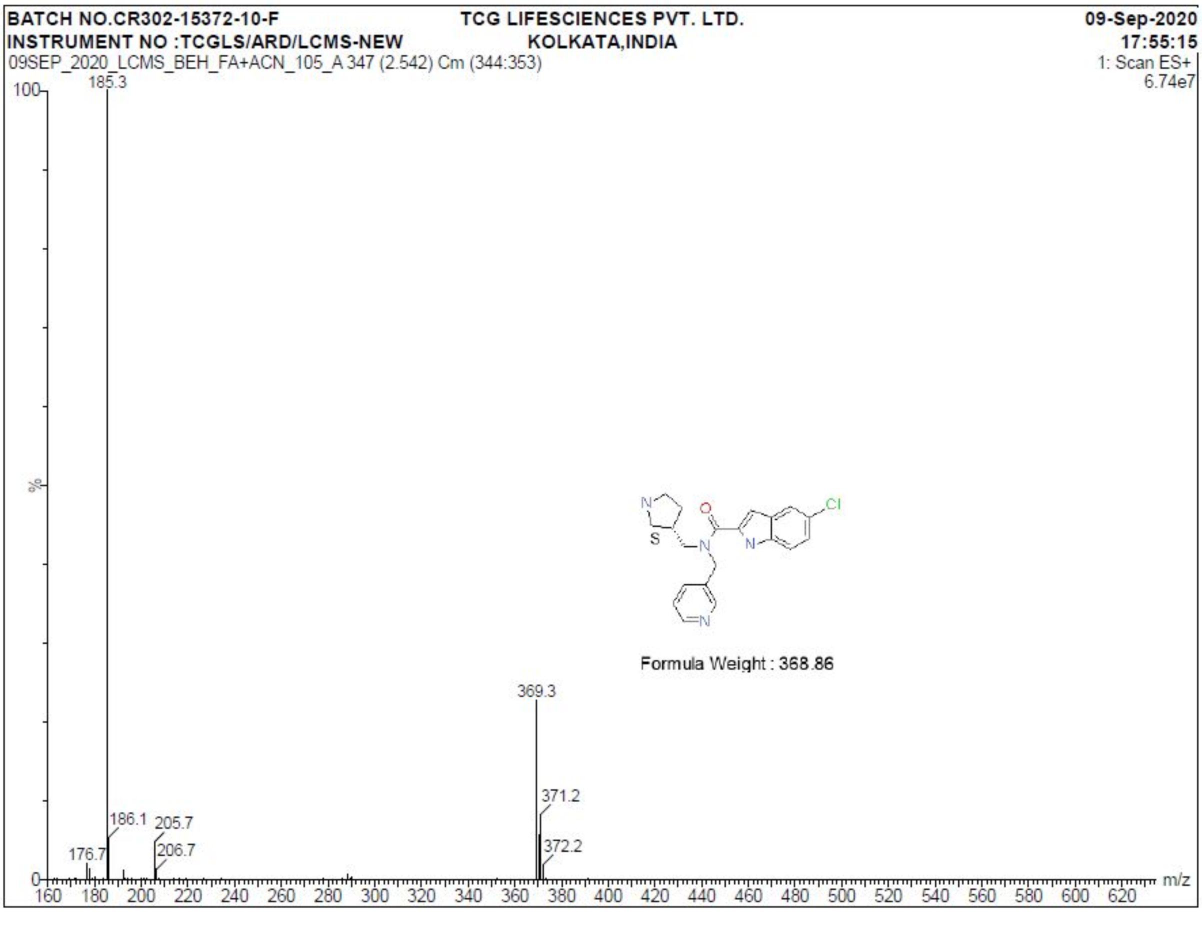
BATCH NO.CR302-15372-10-F
INSTRUMENT NO :TCGLS/ARD/LCMS-NEW
TCG LIFESCIENCES PVT. LTD.
KOLKATA,INDIA
09-Sep-2020
17:55:15
09SEP_2020_LCMS_BEH_FA+ACN_105_A 347 (2.542) Cm (344:353)
1: Scan ES+
6.74e7
185.3
186.1
205.7
206.7
176.7
369.3
371.2
372.2
Formula Weight : 368.86
m/z

LCMS-6a(S)

	Resultados HRMS		
	Responsável:	Diego C Andrade	
	Data:	03/11/22	
	**Laboratório Institucional de Espectrometria de Massas**		

Prof. Dr. Luiz Carlos Dias
Aluna Mariana Ferrer
Amostra MMV315

Espectro completo de 80 a 750 m/z em modo positivo

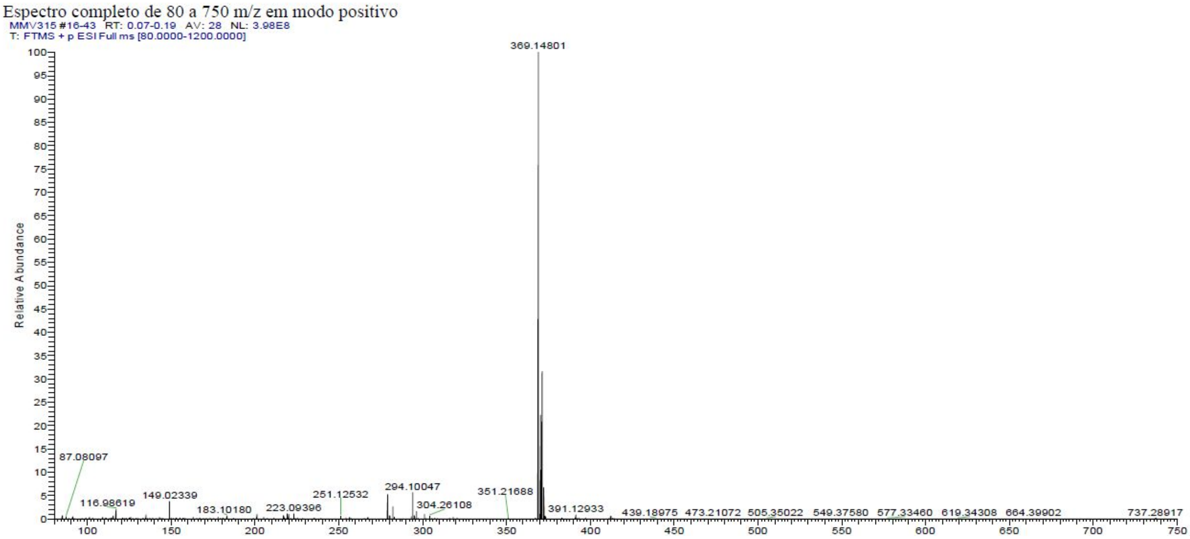

HRMS-6a(S)

	Resultados HRMS		
	Responsável:	Diego C Andrade	
	Data:	03/11/22	
	**Laboratório Institucional de Espectrometria de Massas**		

Comparação entre espectro real (superior) e simulado (inferior) para C20H21ClN4OH+

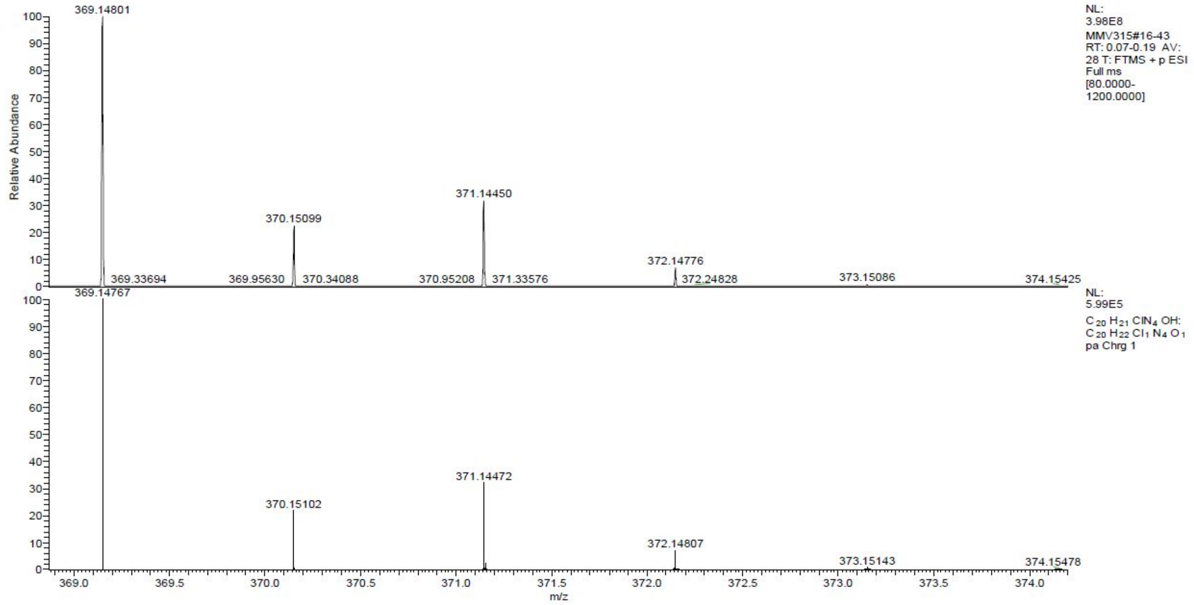

HRMS-6a(S)

N-(3-pyridylmethyl)-N-(pyrrolidin-3-ylmethyl)-1H-indole-2-carboxamide (6b):

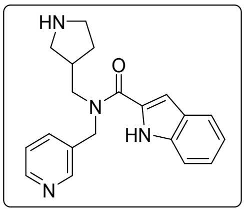

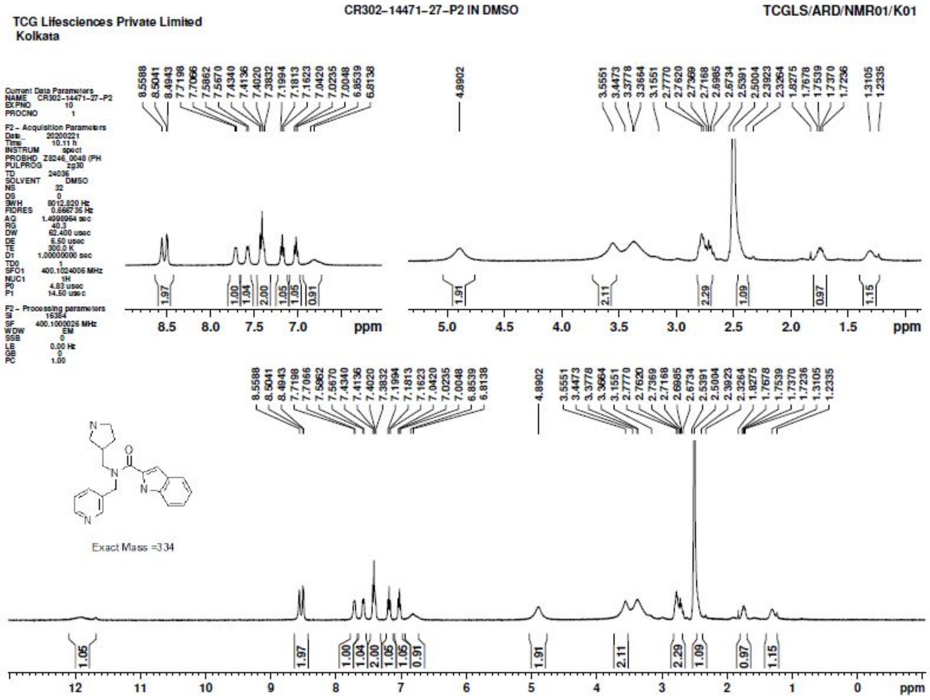

^{1}H NMR (400 MHz, DMSO-d_6)-6b

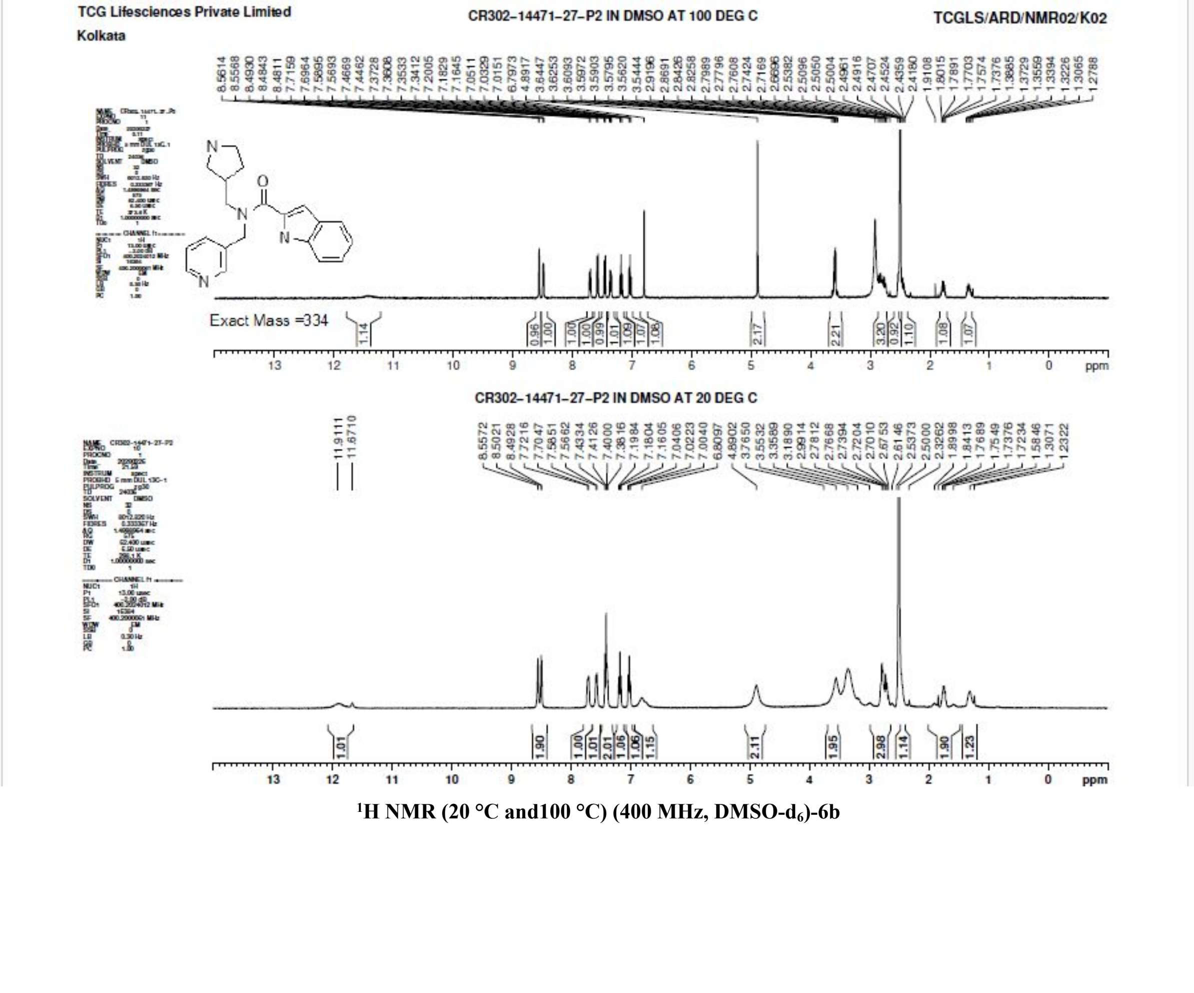

^{1}H NMR (20 °C and100 °C) (400 MHz, DMSO-d_6)-6b

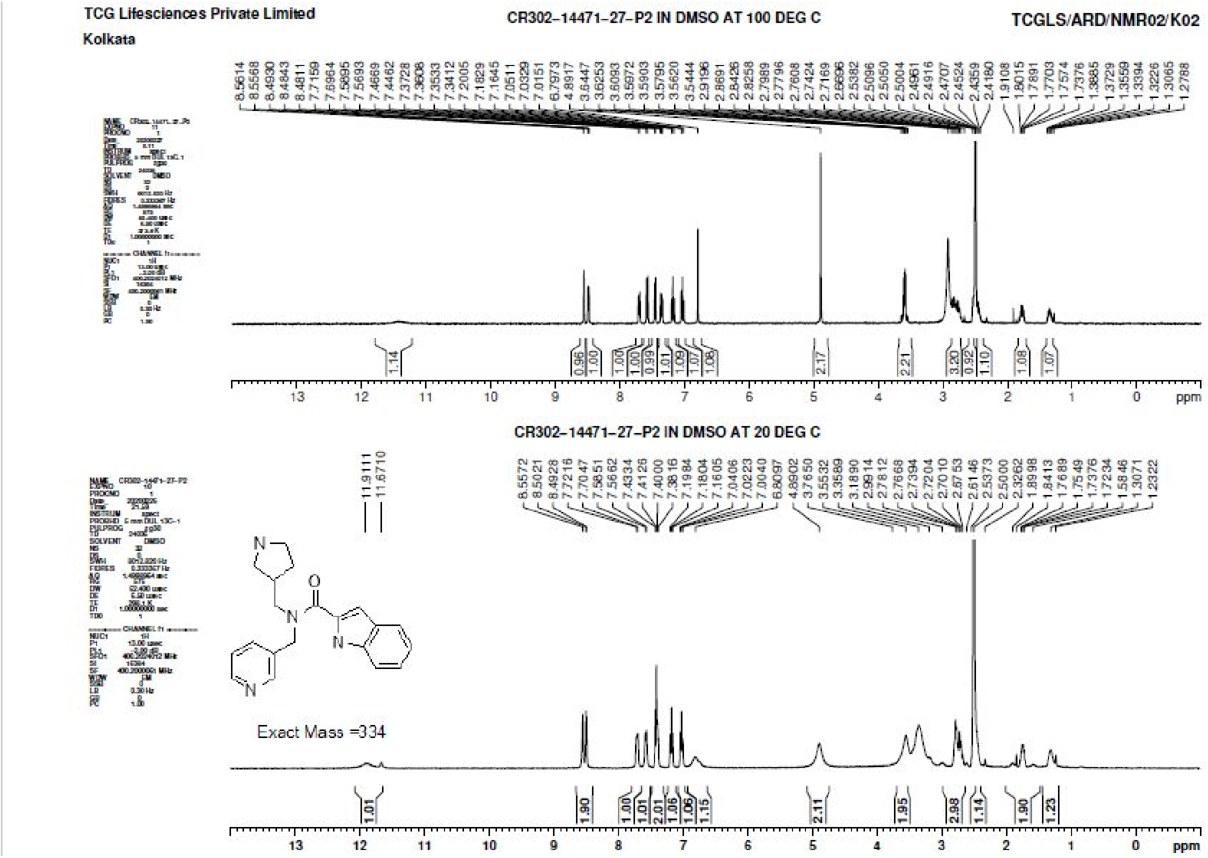

^1H NMR (20 °C and100 °C) (400 MHz, DMSO-d_6)-6b

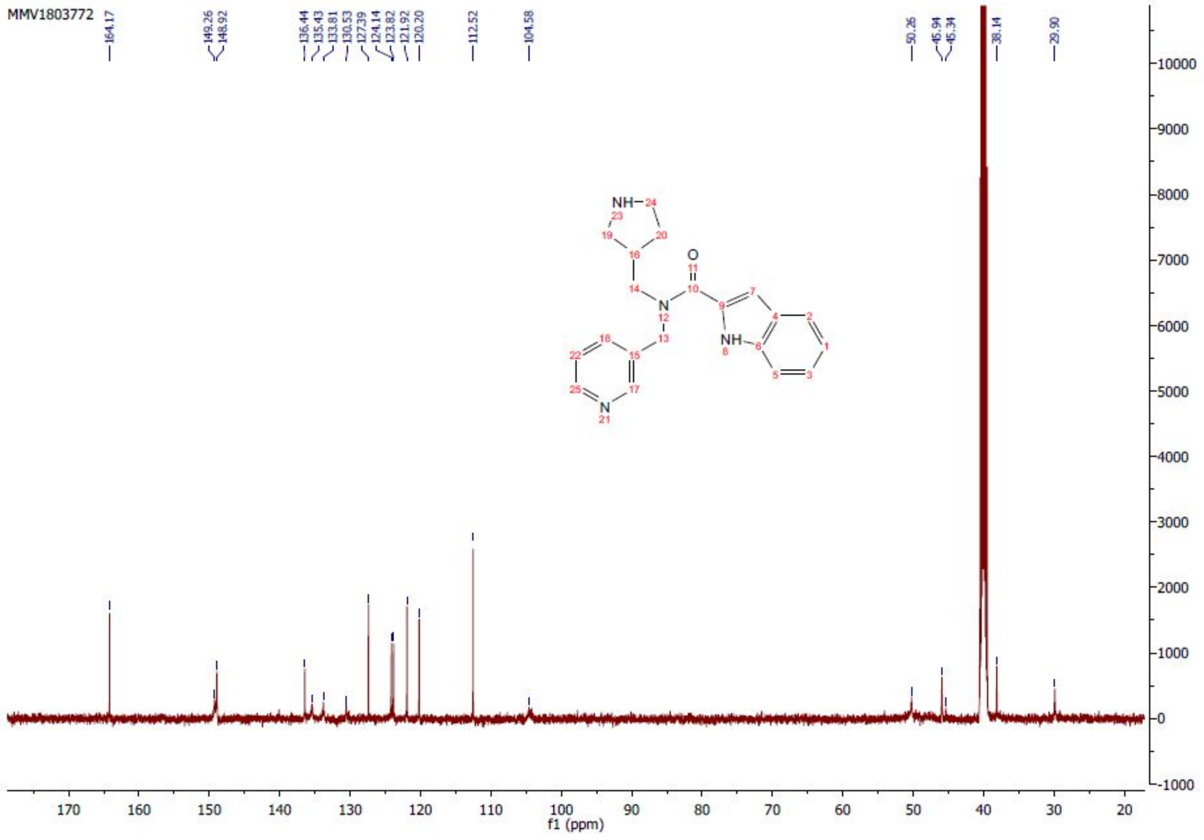

^{13}C NMR (125 MHz, DMSO-d_6)-6b

KOLKATA, INDIA

Sample Name: CR302-14471-27-P2-2 Acq. Time: 10:32 Acq. Date: Monday, February 24, 2020

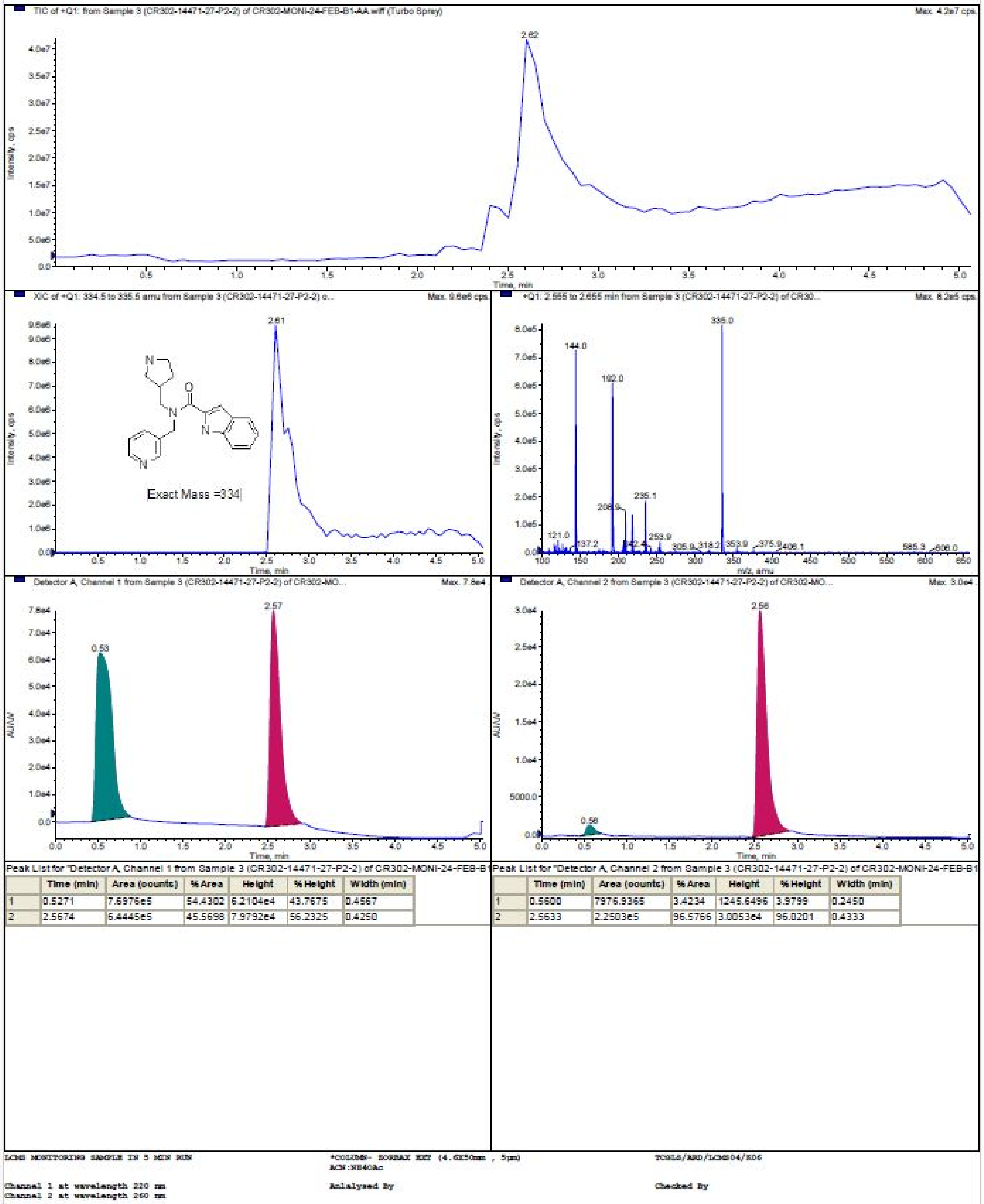

Peak List for "Detector A, Channel 1 from Sample 3 (CR302-14471-27-P2-2) of CR302-MONI-24-FEB-B1

	Time (min)	Area (counts)	%Area	Height	%Height	Width (min)
1	0.5271	7.6976e5	54.4302	6.2104e4	43.7675	0.4567
2	2.5674	6.4445e5	45.5698	7.9792e4	56.2325	0.4250

Peak List for "Detector A, Channel 2 from Sample 3 (CR302-14471-27-P2-2) of CR302-MONI-24-FEB-B1

	Time (min)	Area (counts)	%Area	Height	%Height	Width (min)
1	0.5600	7976.9365	3.4234	1245.6496	3.9799	0.2450
2	2.5633	2.2503e5	96.5766	3.0053e4	96.0201	0.4333

LCMS MONITORING SAMPLE IN 5 MIN RUN

*COLUMN- ZORBAX EXT (4.6X50mm , 5µm)
ACN:NH4OAc

TCGLS/ARD/LCMS04/KD6

Channel 1 at wavelength 220 nm
Channel 2 at wavelength 260 nm

Anlalysed By

Checked By

LCMS-6b

	Resultados HRMS	
	Responsável:	Diego C Andrade
	Data:	19/12/22

Laboratório Institucional de Espectrometria de Massas

Prof. Dr. Luiz Carlos Dias
Aluna Mariana Ferrer Casal
Amostra MMV772

Espectro completo de 80 a 1200 m/z em modo positivo

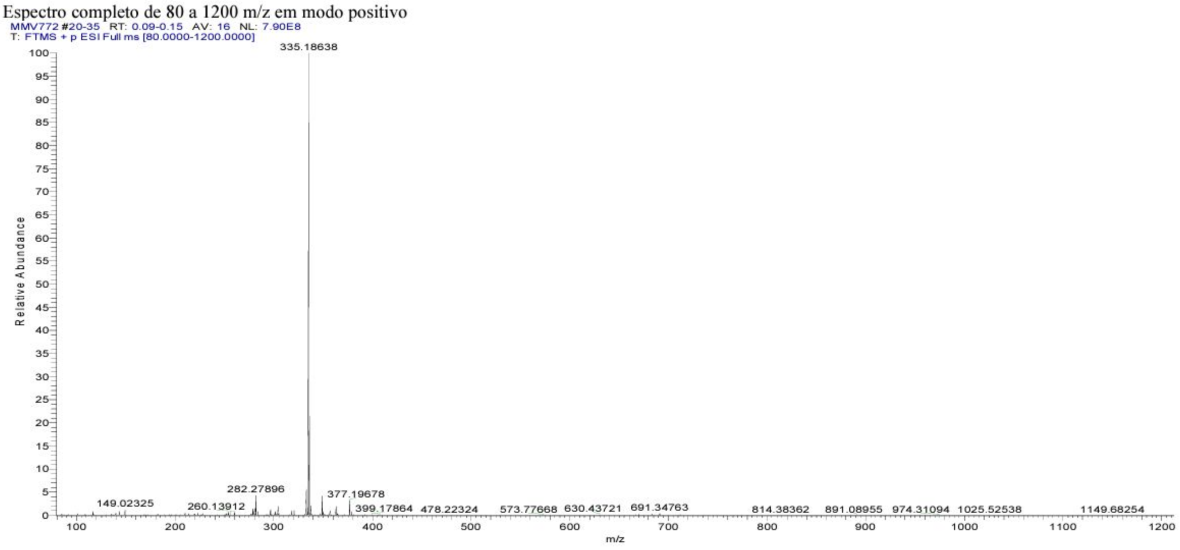

HRMS-6b

	Resultados HRMS	
	Responsável:	Diego C Andrade
	Data:	19/12/22

Laboratório Institucional de Espectrometria de Massas

Comparação entre espectro real (superior) e simulado (inferior) para C20H22N4OH+

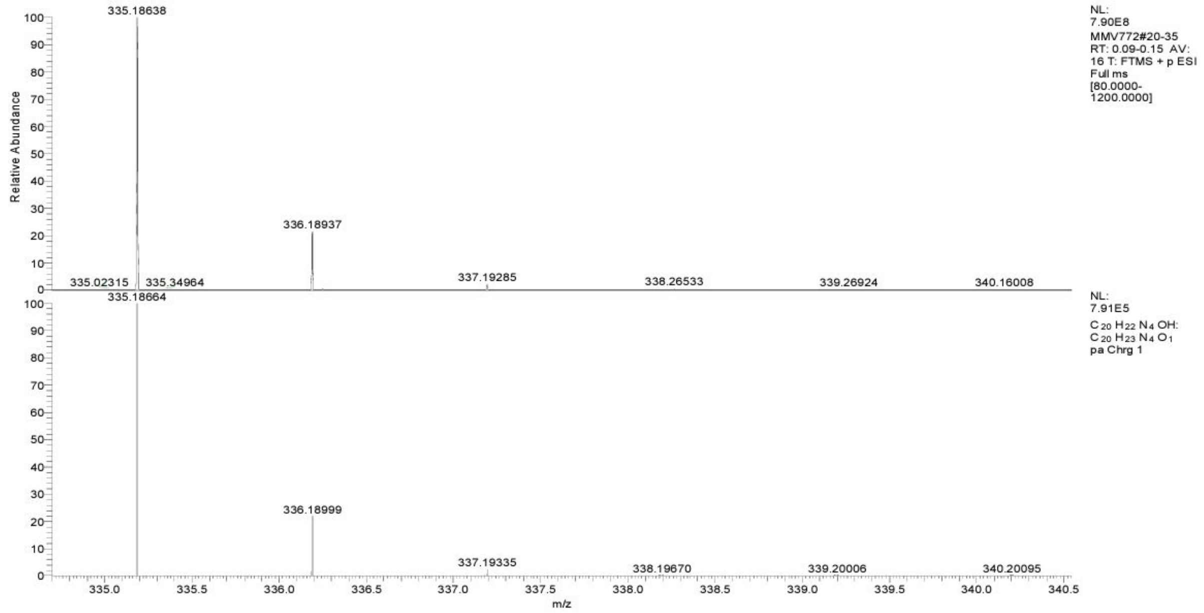

HRMS-6b

7-chloro-N-(3-pyridylmethyl)-N-(pyrrolidin-3-ylmethyl)-1H-indole-2-carboxamide (6c):

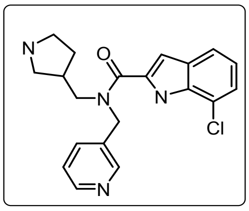

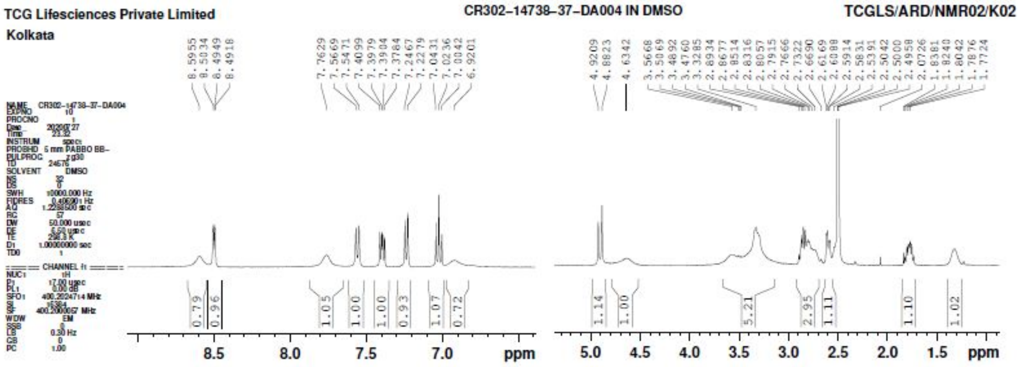

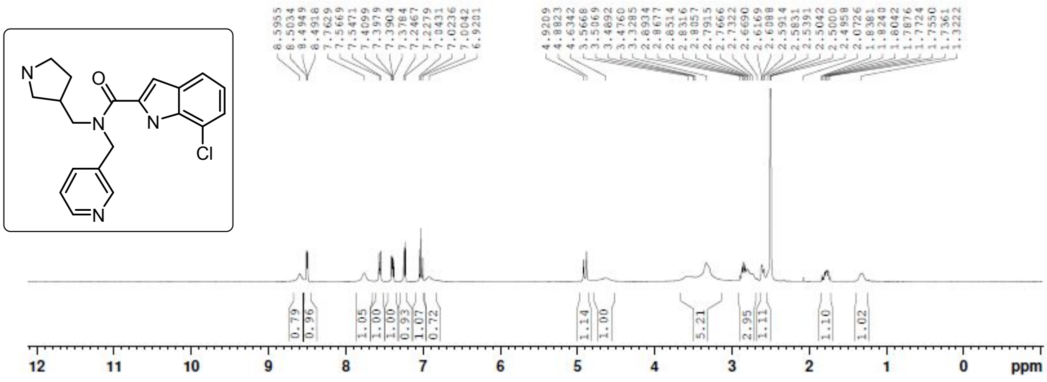

^{1}H NMR (400 MHz, DMSO-d$_6$)-6c

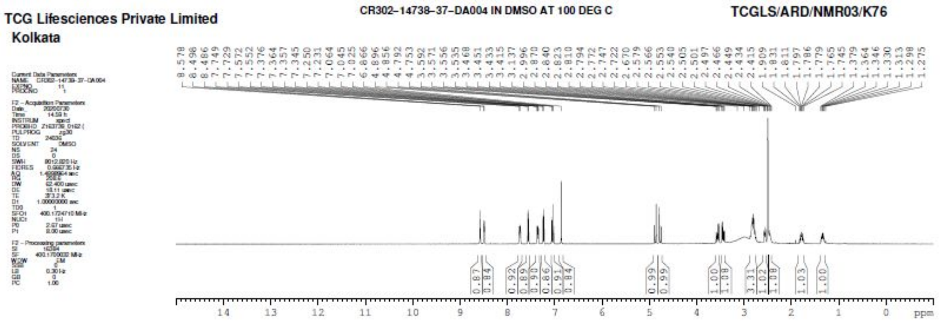

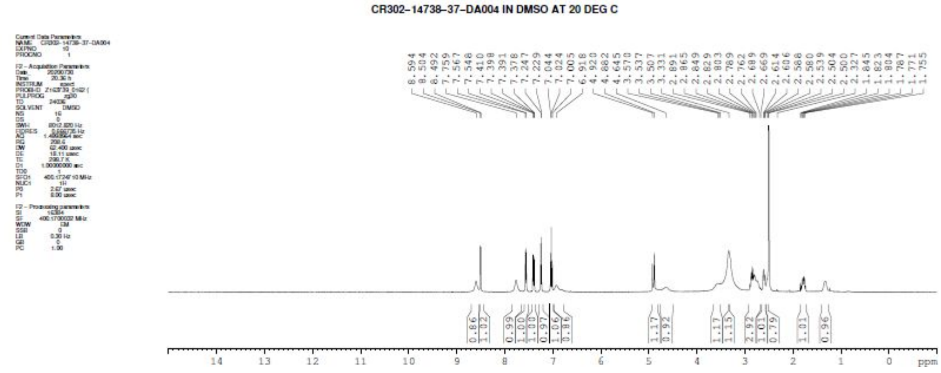

^{1}H NMR (20 °C and100 °C) (400 MHz, DMSO-d$_6$)-6c

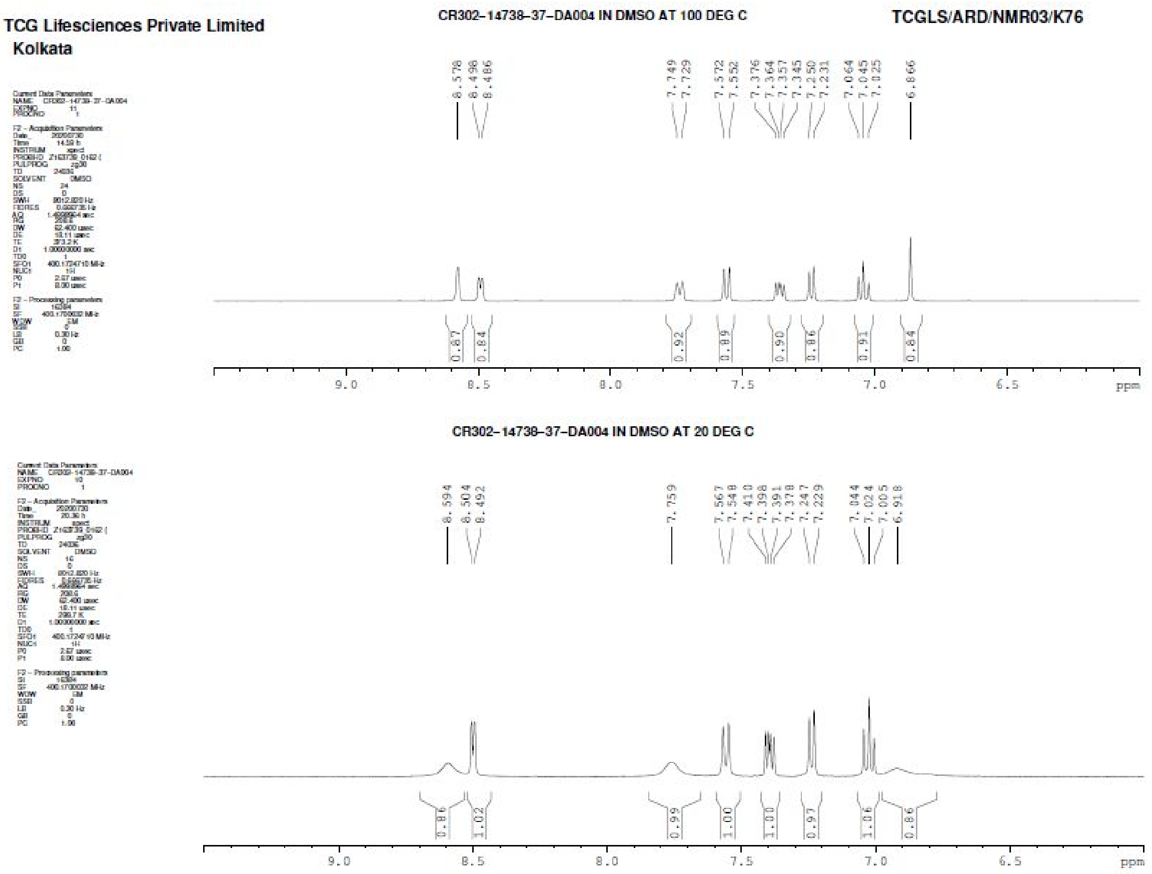

^{1}H NMR (20 °C and100 °C) (400 MHz, DMSO-d_6)-6c

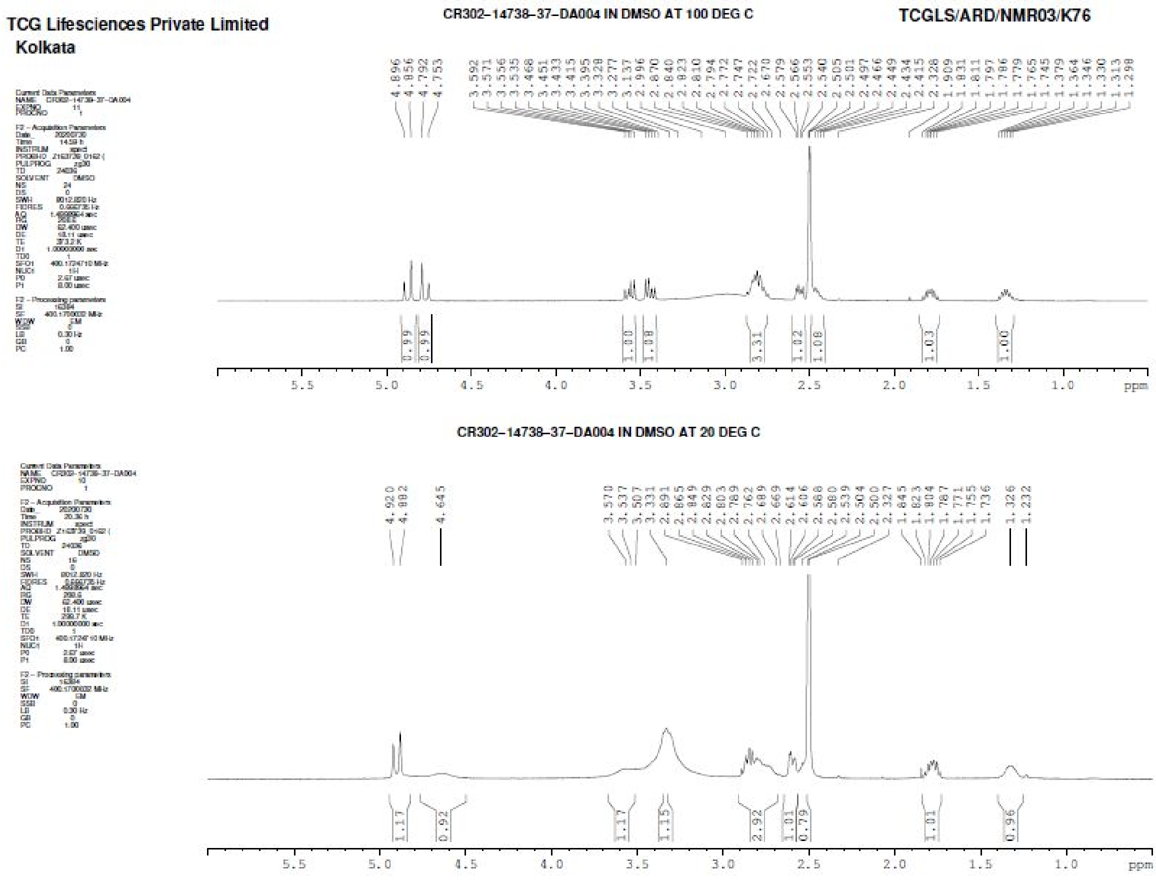

^{1}H NMR (20 °C and100 °C) (400 MHz, DMSO-d_6)-6c

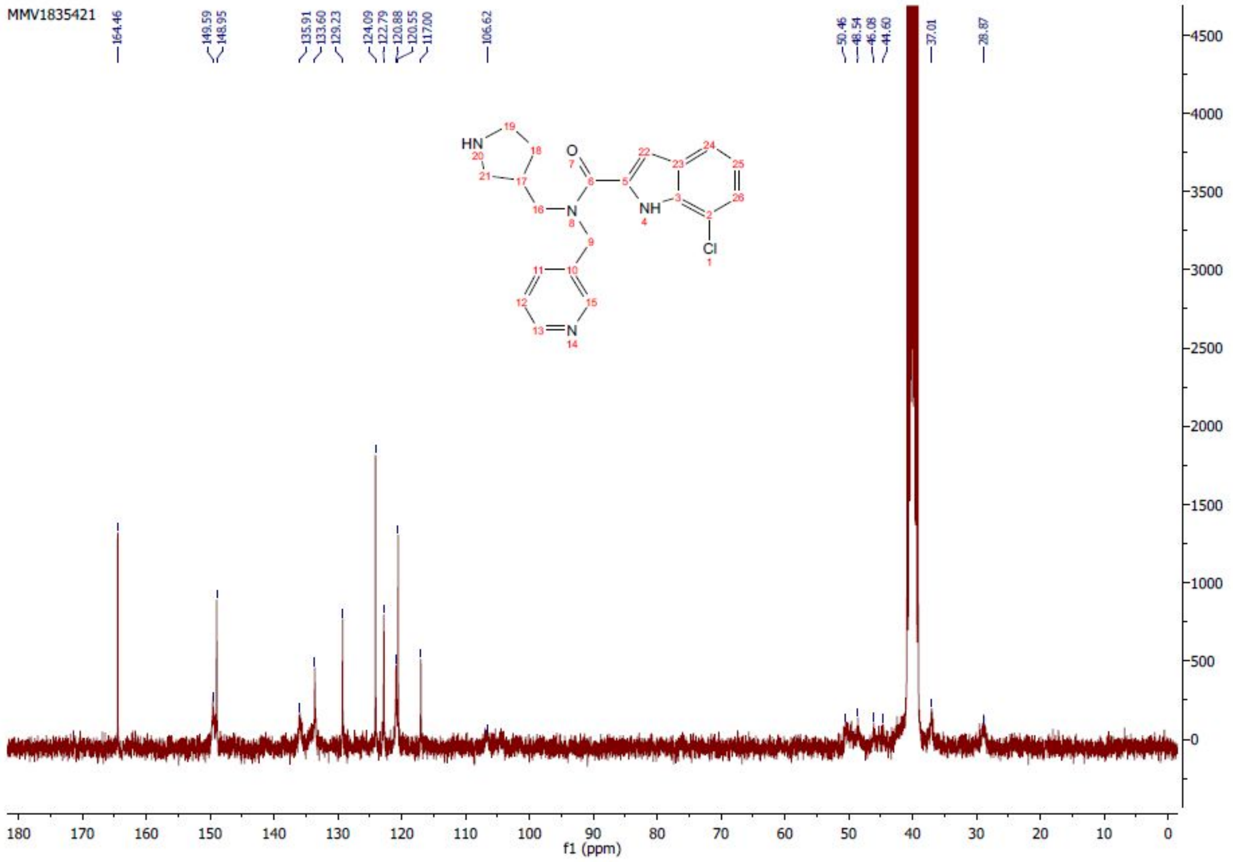

^{13}C NMR (75 MHz, DMSO-d$_6$)-6c

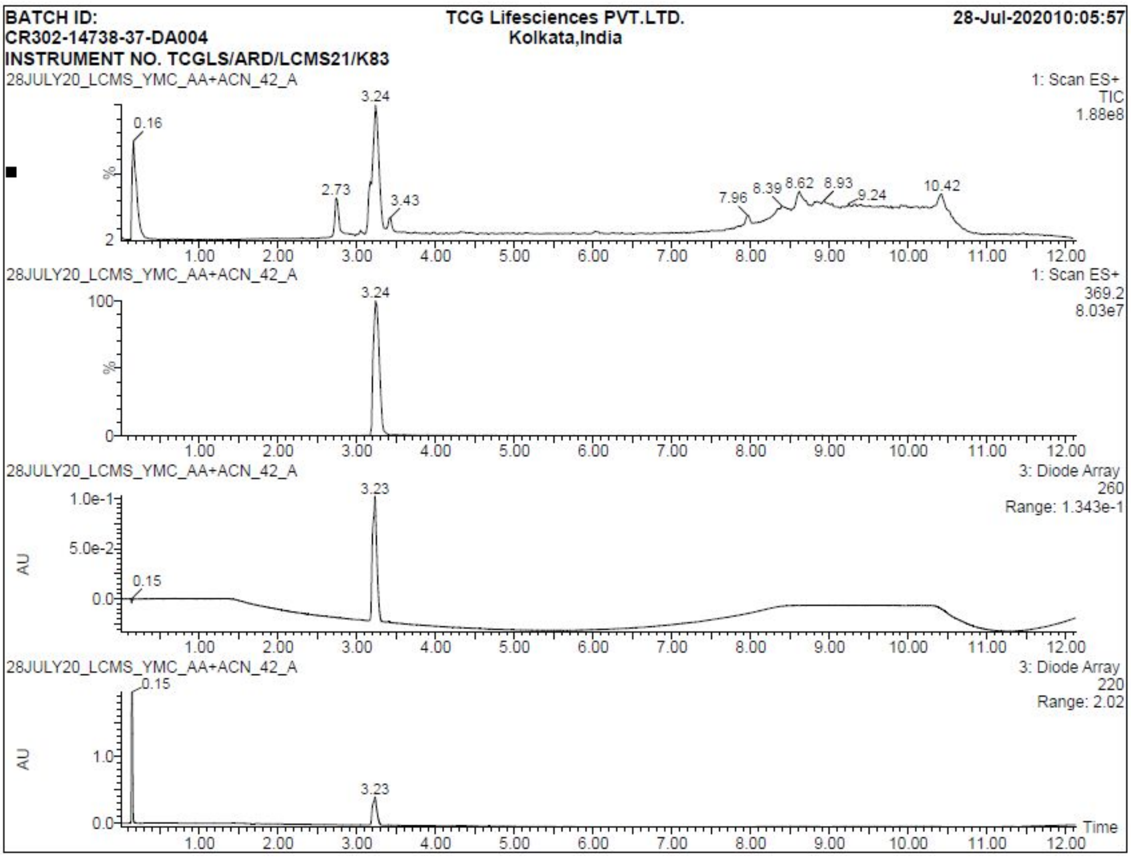

LCMS-6c

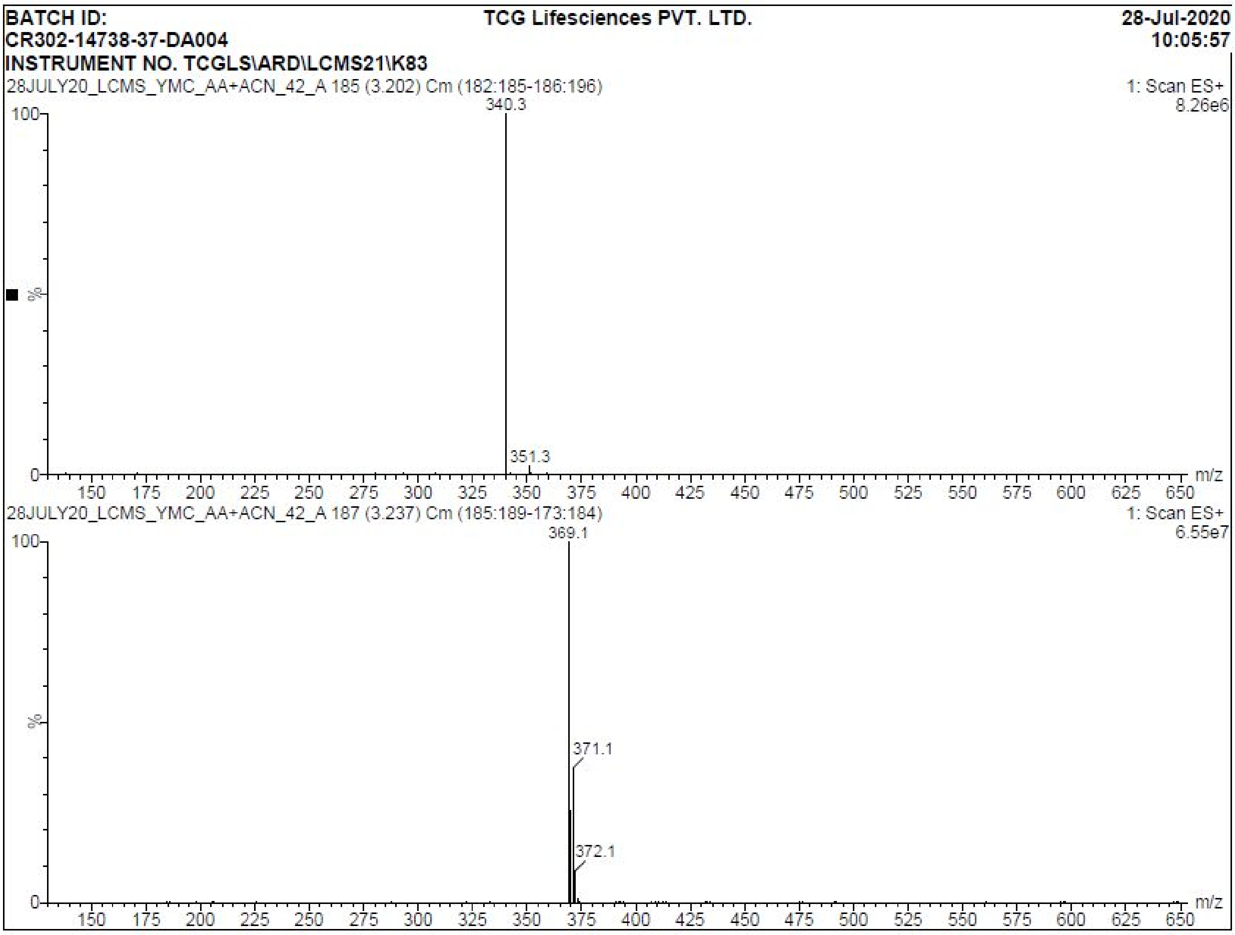

LCMS-6c

	Resultados HRMS	
Instituto de Química	Responsável:	Diego C Andrade
	Data:	06/10/22
	**Laboratório Institucional de Espectrometria de Massas**	LIE Laboratório Institucional de Espectrometria de Massas

Prof. Dr. Luiz Carlos Dias
Aluno Anwar Shamim
Amostra MMV421

Espectro completo de 50 a 750 m/z em modo positivo

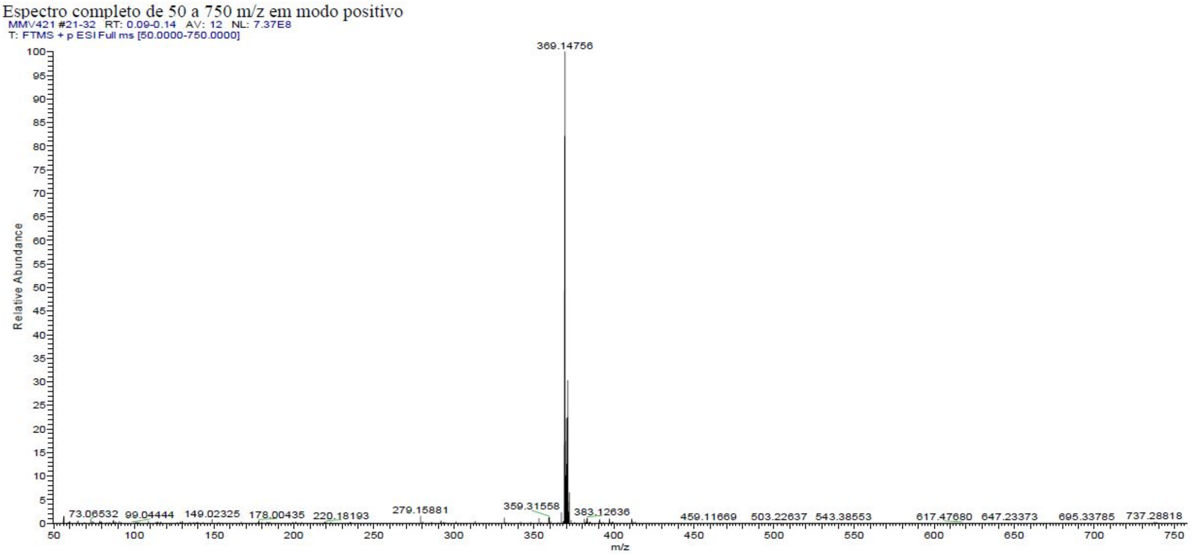

HRMS-6c

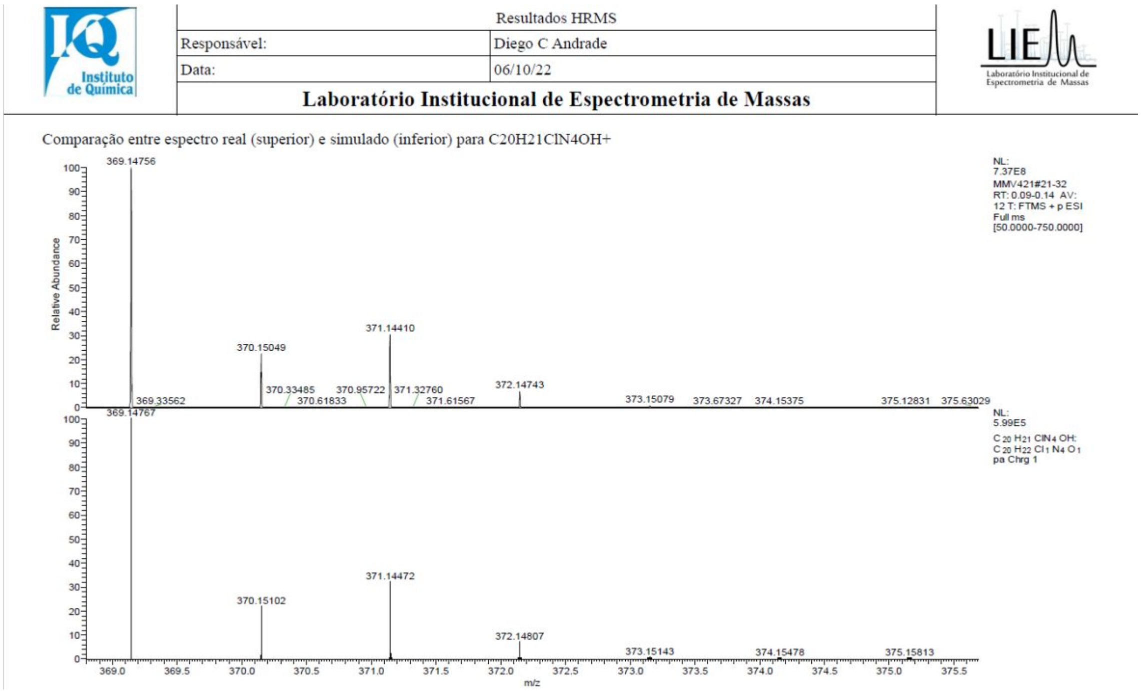

HRMS-6c

5-methoxy-N-(3-pyridylmethyl)-N-(pyrrolidin-3-ylmethyl)-1H-indole-2-carboxamide (6d):

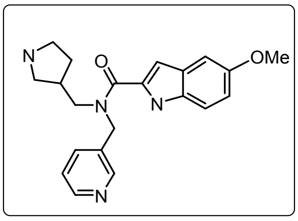

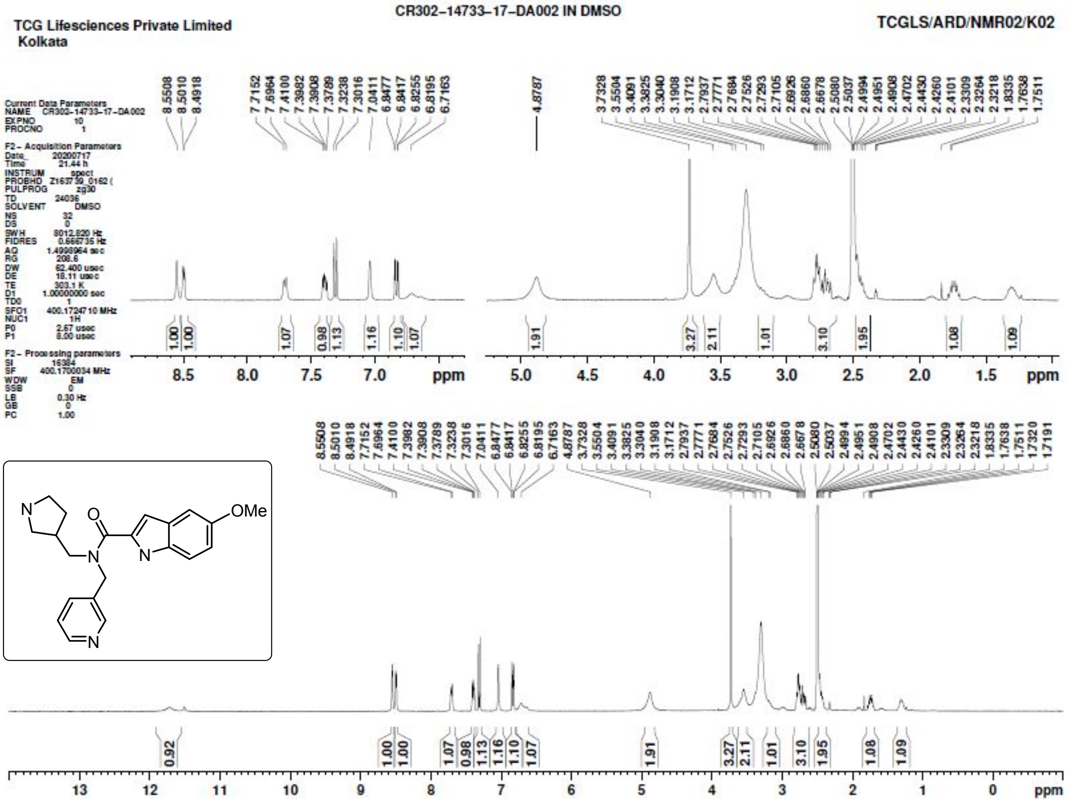

^{1}H NMR (400 MHz, DMSO-d_6)-6d

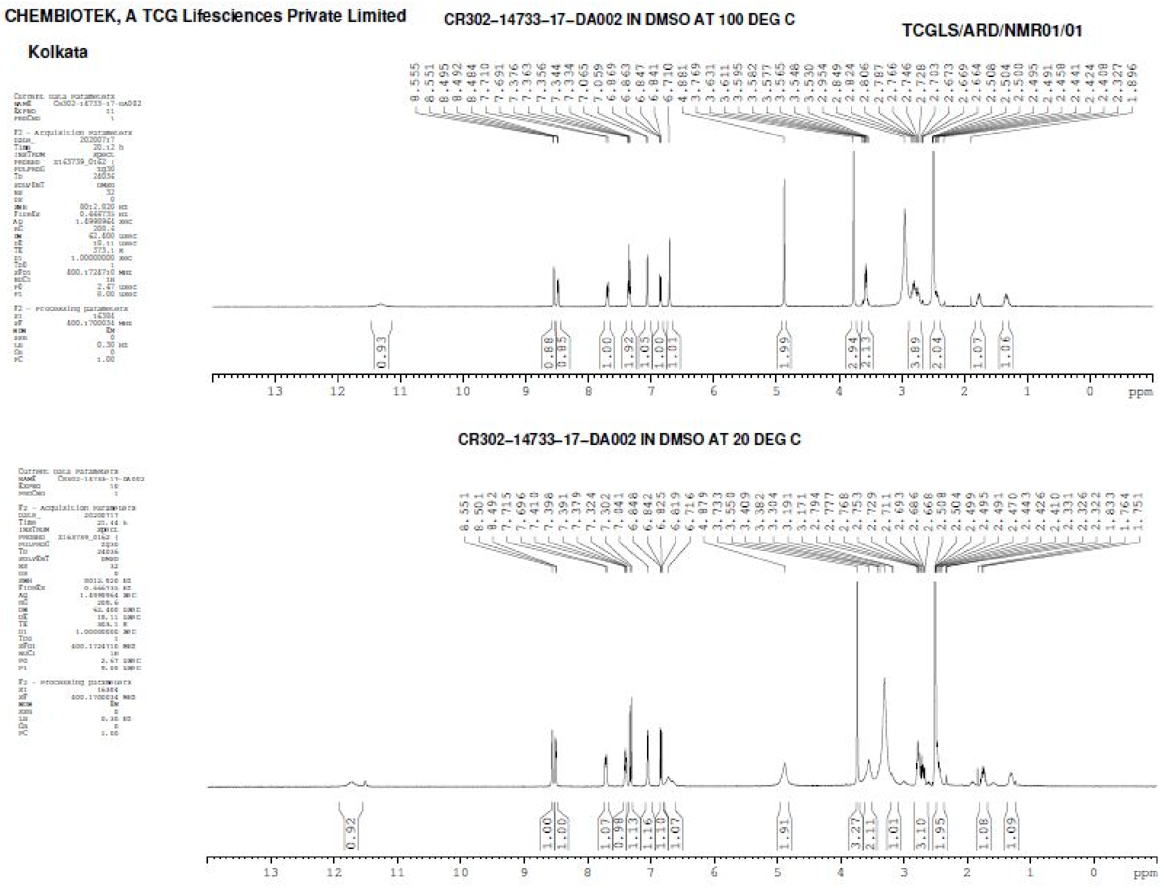

^{1}H NMR (20 °C and100 °C) (400 MHz, DMSO-d_6)-6d

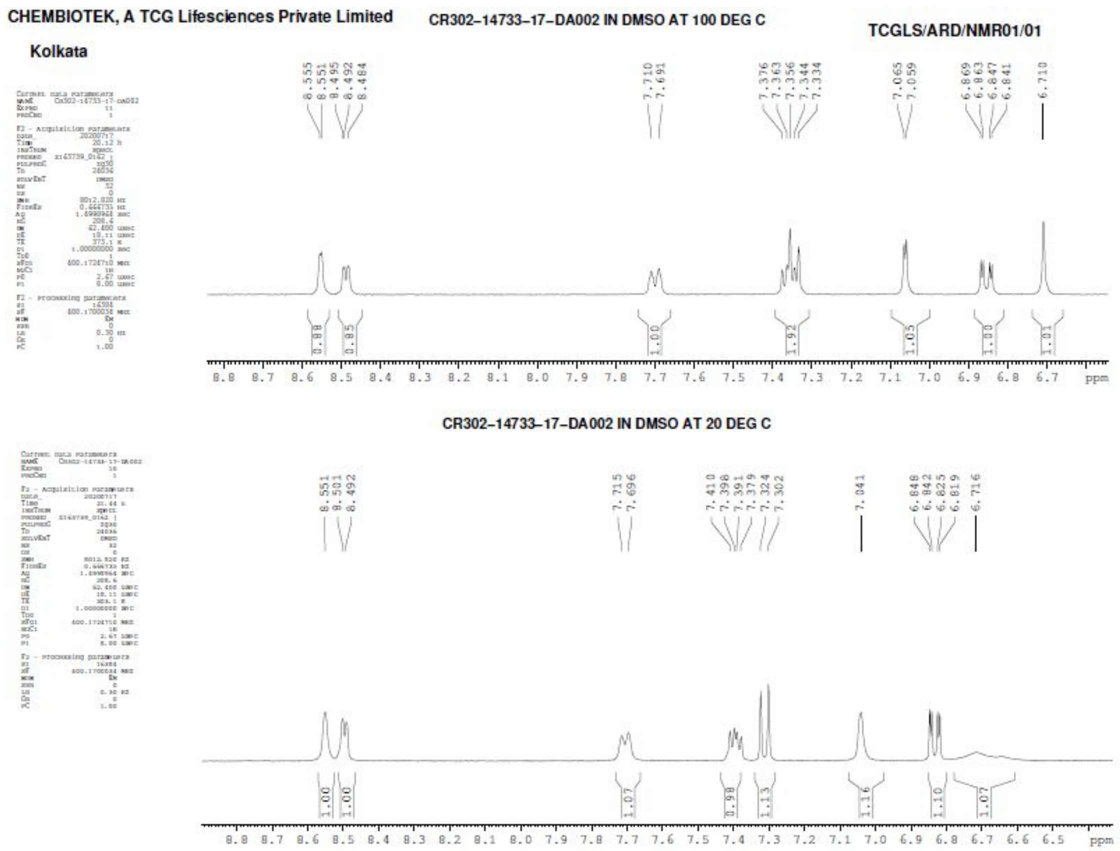

^{1}H NMR (20 °C and100 °C) (400 MHz, DMSO-d_6)-6d

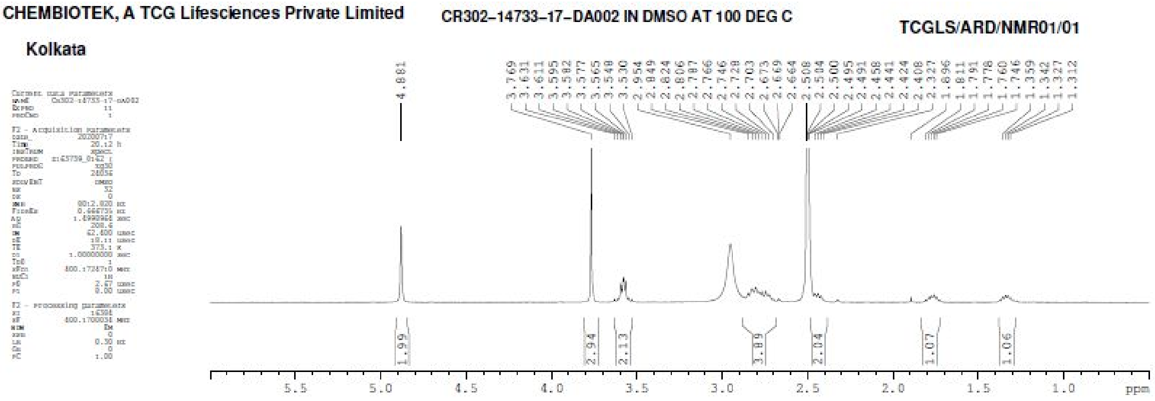

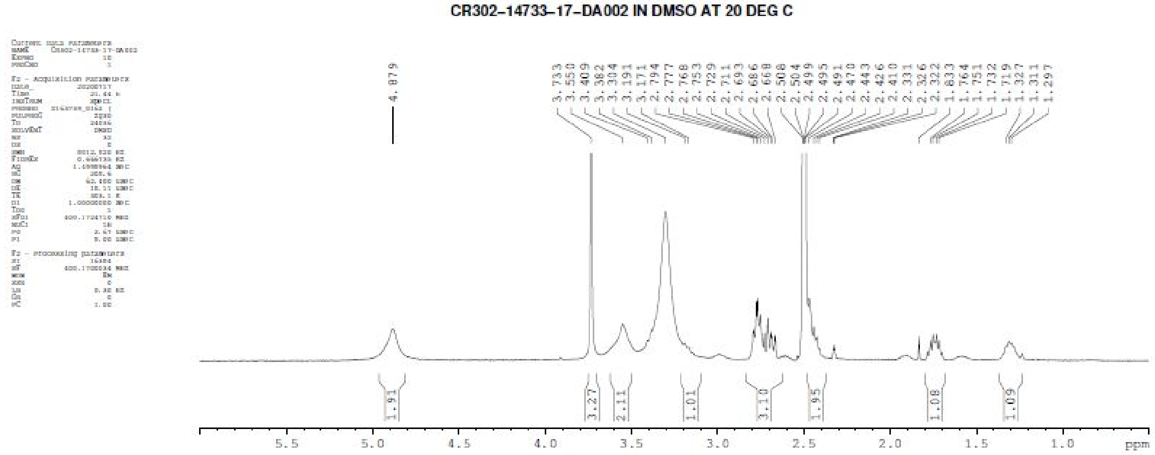

^{1}H NMR (20 °C and100 °C) (400 MHz, DMSO-d_6)-6d

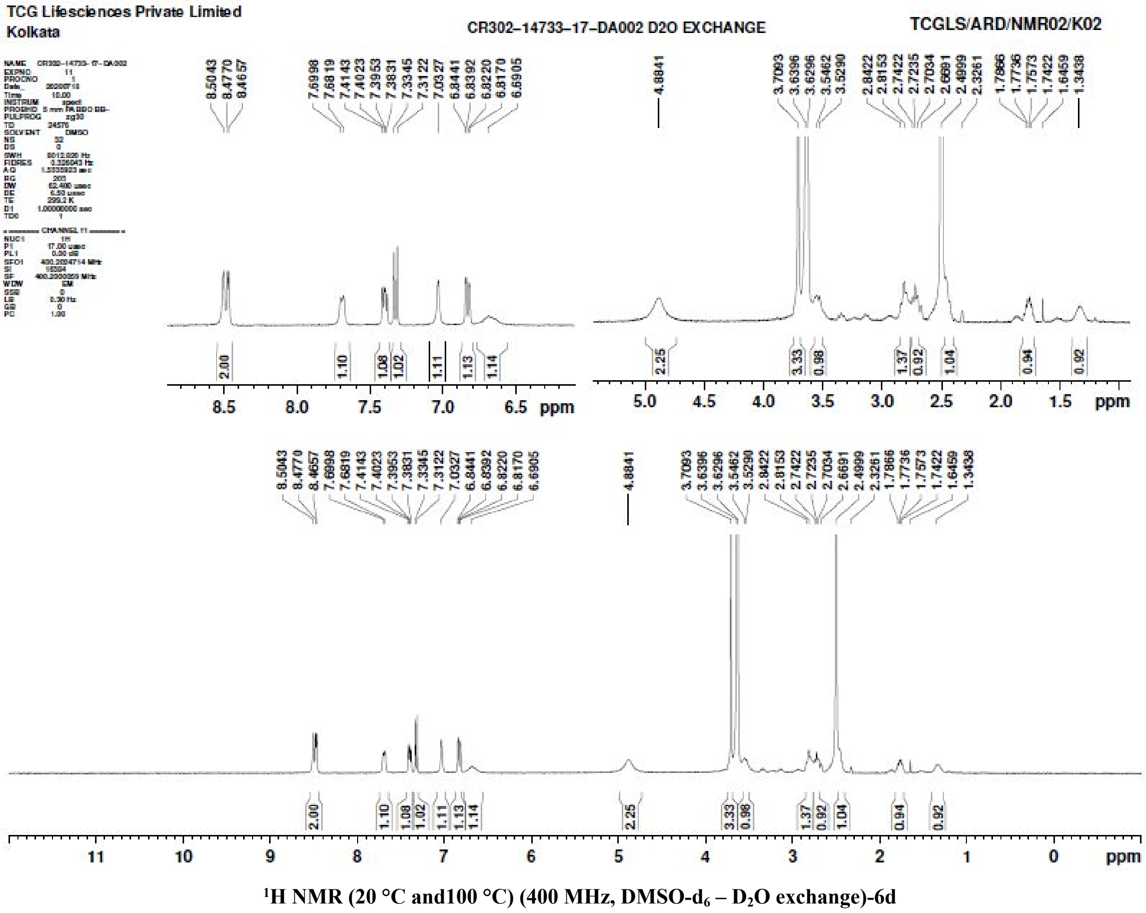

^{1}H NMR (20 °C and100 °C) (400 MHz, DMSO-d_6 – D_2O exchange)-6d

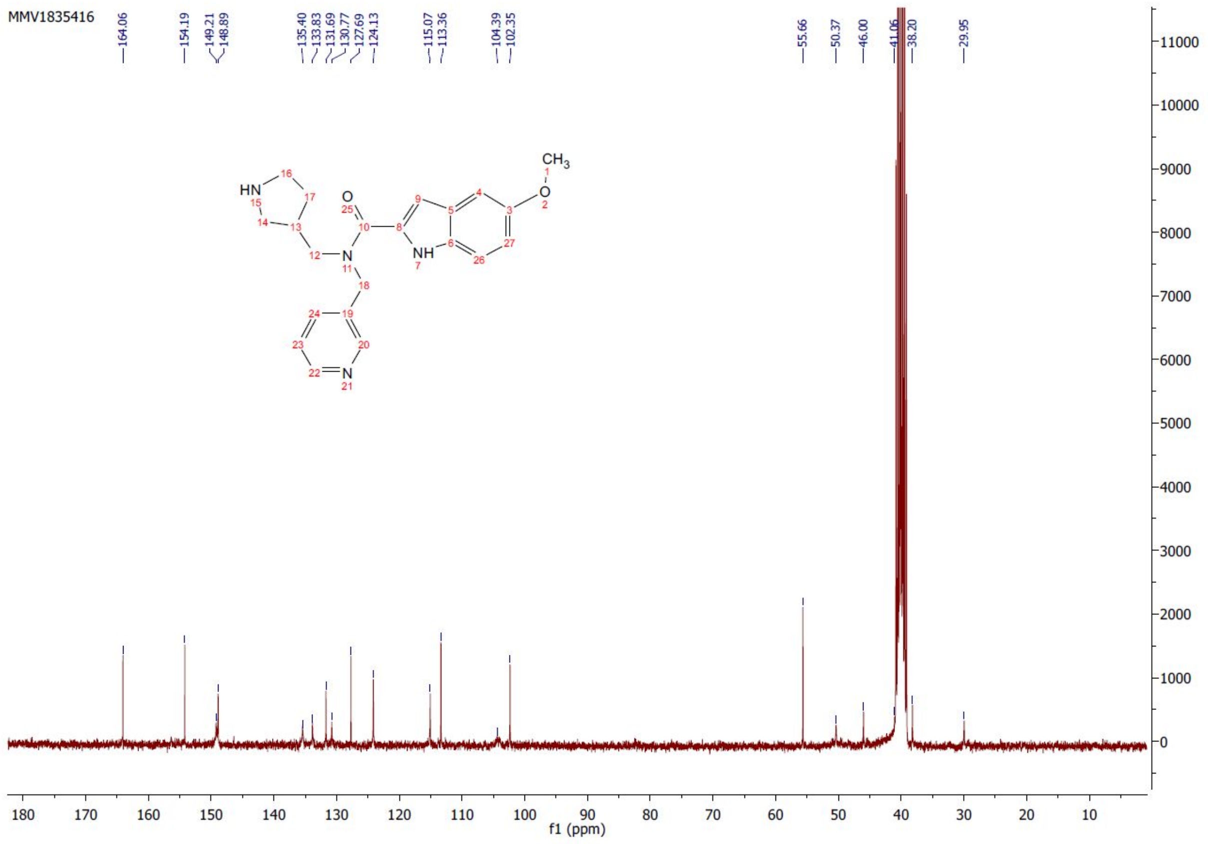

^{13}C NMR (75 MHz, DMSO-d_6)-6d

TCG Lifesciences Pvt Ltd , Kolkata , India

Data file:	D:\LCMS 2019\DATA\JULY-2020\1772020_J 2020-07-17 16-25-01\CR302-14733-17-DA002.D		
Sample name:	**CR302-14733-17-DA002**		
Instrument:	TCGLS_ARD_LCMS20_K79	Location:	D1F-A7
Injection date:	7/17/2020 4:52:13 PM	Injection volume:	0.800
Acq. method:	Moni +ve-ve_FA.M		
Description:	Column-YMC TRIART C18(33x2.1mm,3μ)-FAF		

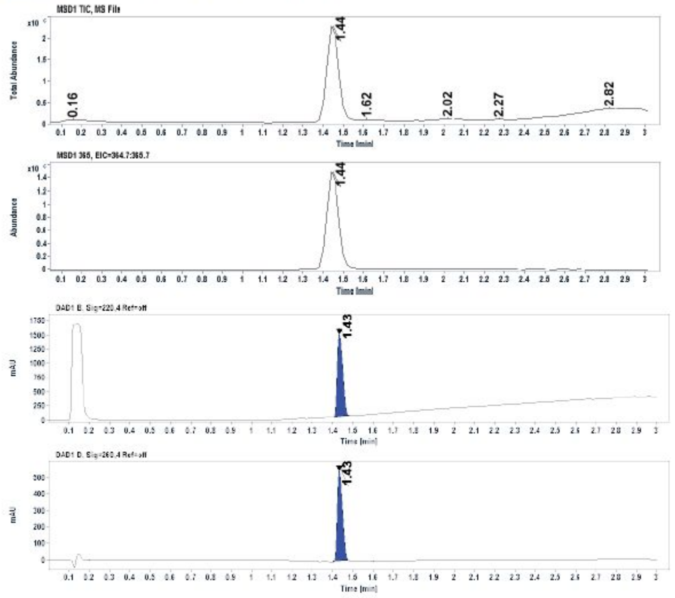

Signal: DAD1 B, Sig=220,4 Ref=off

RT [min]	Type	Width [min]	Area	Height	Area%	Name
1.43	MM	0.0310	2716.5840	1460.1840	100.0000	

Signal: DAD1 D, Sig=260,4 Ref=off

RT [min]	Type	Width [min]	Area	Height	Area%	Name
1.43	MM	0.0290	949.1382	546.0670	100.0000	

LCMS-6d

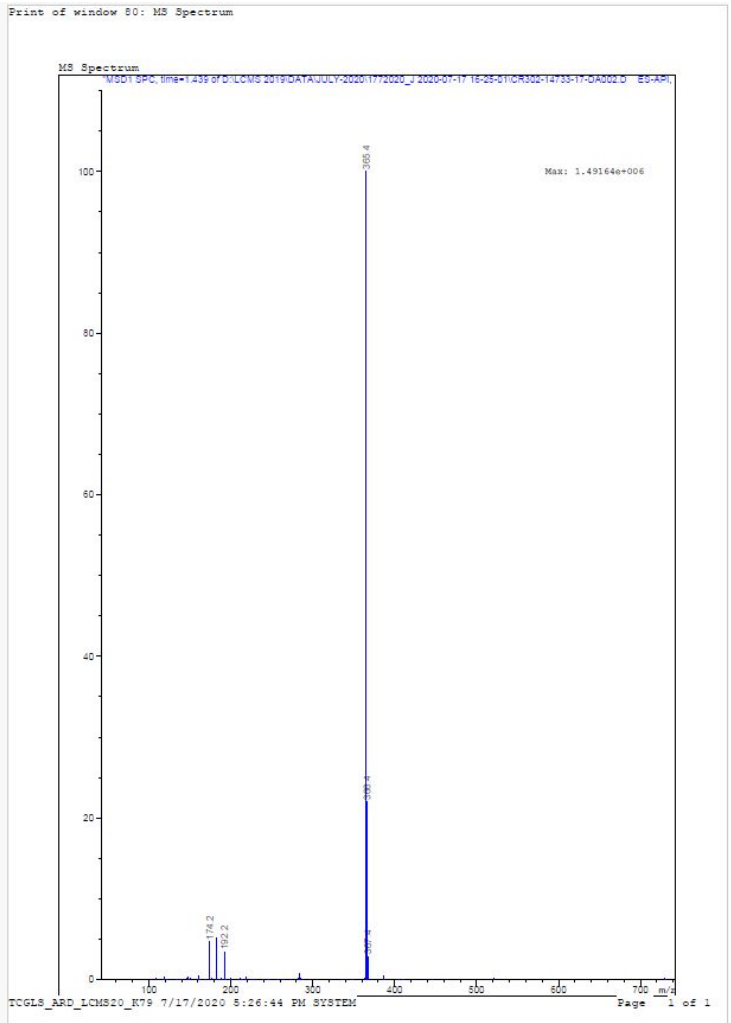

LCMS-6d

	Resultados HRMS		
Instituto de Química	Responsável:	Diego C Andrade	LIE Laboratório Institucional de Espectrometria de Massas
	Data:	06/10/22	
	Laboratório Institucional de Espectrometria de Massas		

Prof. Dr. Luiz Carlos Dias
Aluno Anwar Shamim
Amostra MMV416

Espectro completo de 50 a 750 m/z em modo positivo

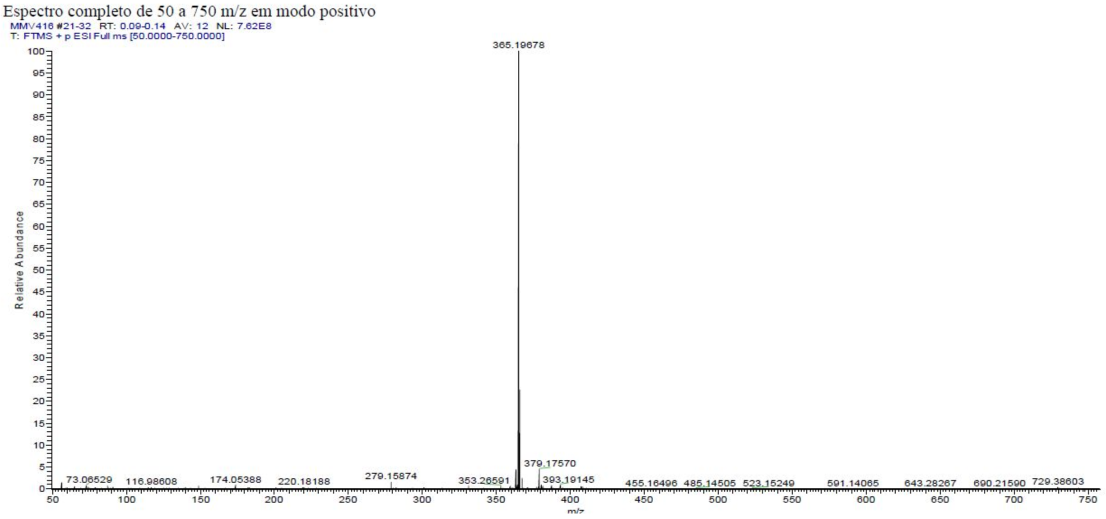

HRMS-6d

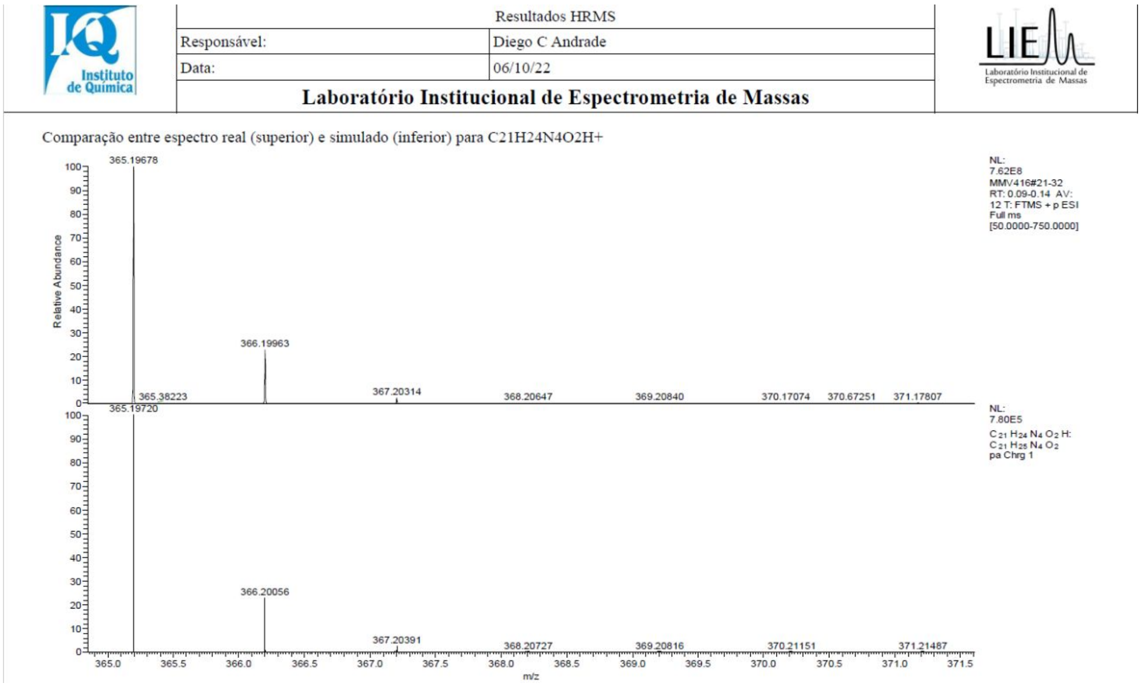

HRMS-6d

5-fluoro-N-(3-pyridylmethyl)-N-(pyrrolidin-3-ylmethyl)-1H-indole-2-carboxamide (6e):

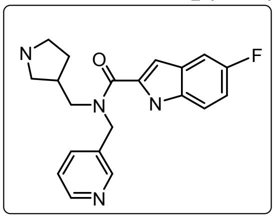

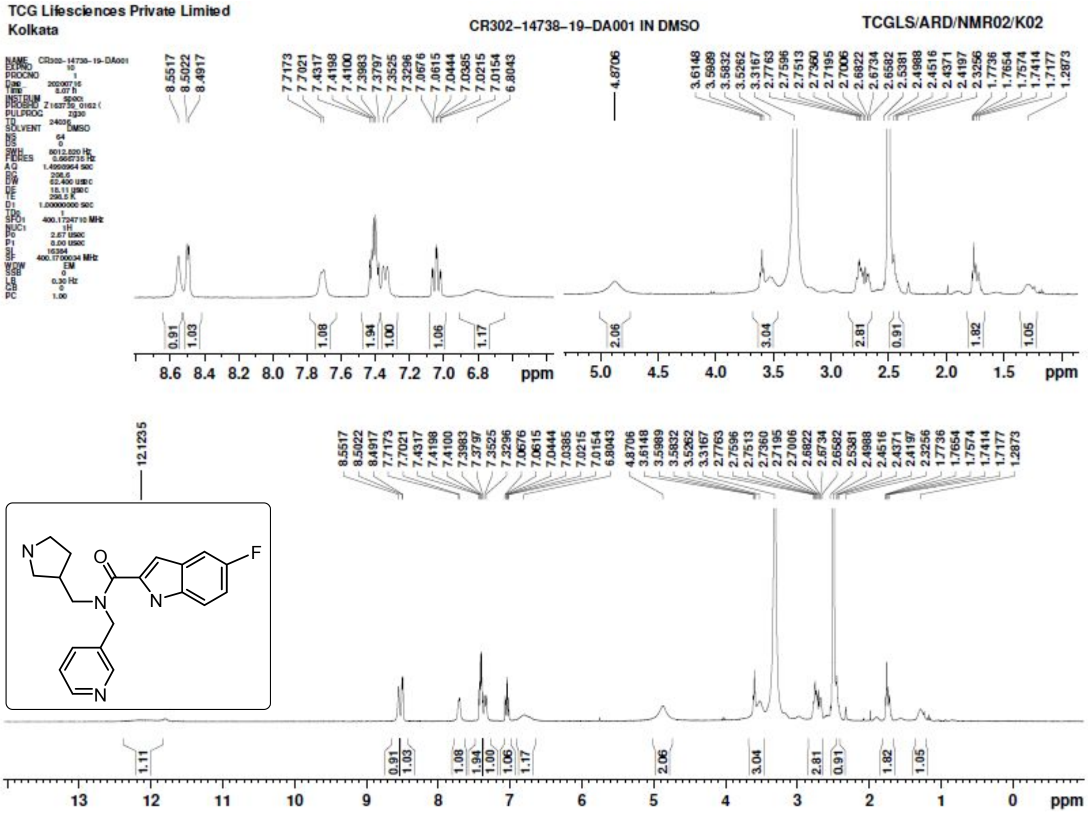

^{1}H NMR (400 MHz, DMSO-d_6)-6e

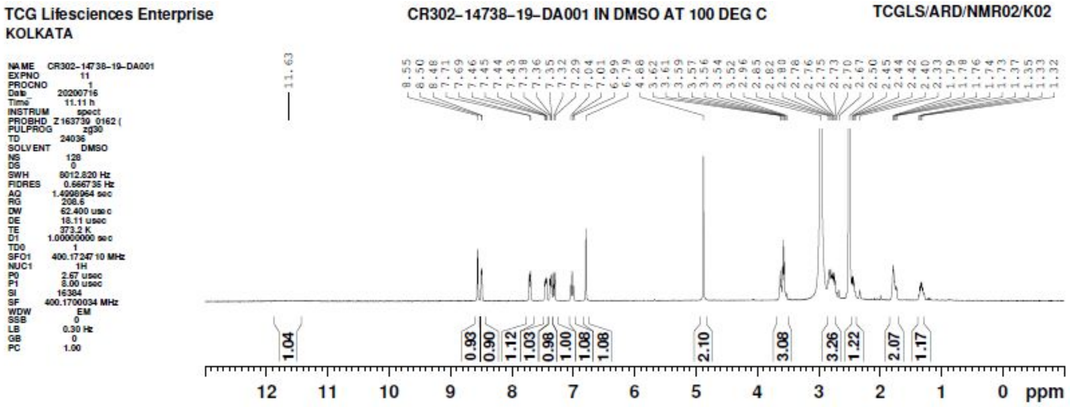

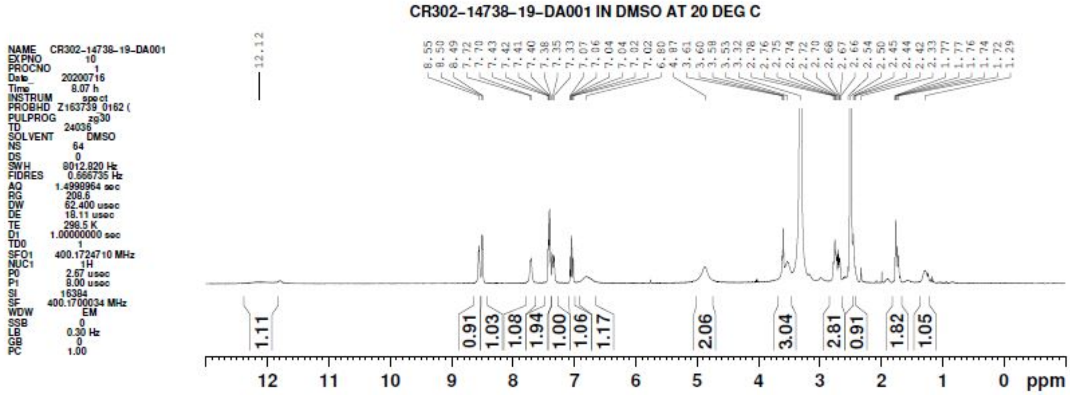

^{1}H NMR (20 °C and100 °C) (400 MHz, DMSO-d_6)-6e

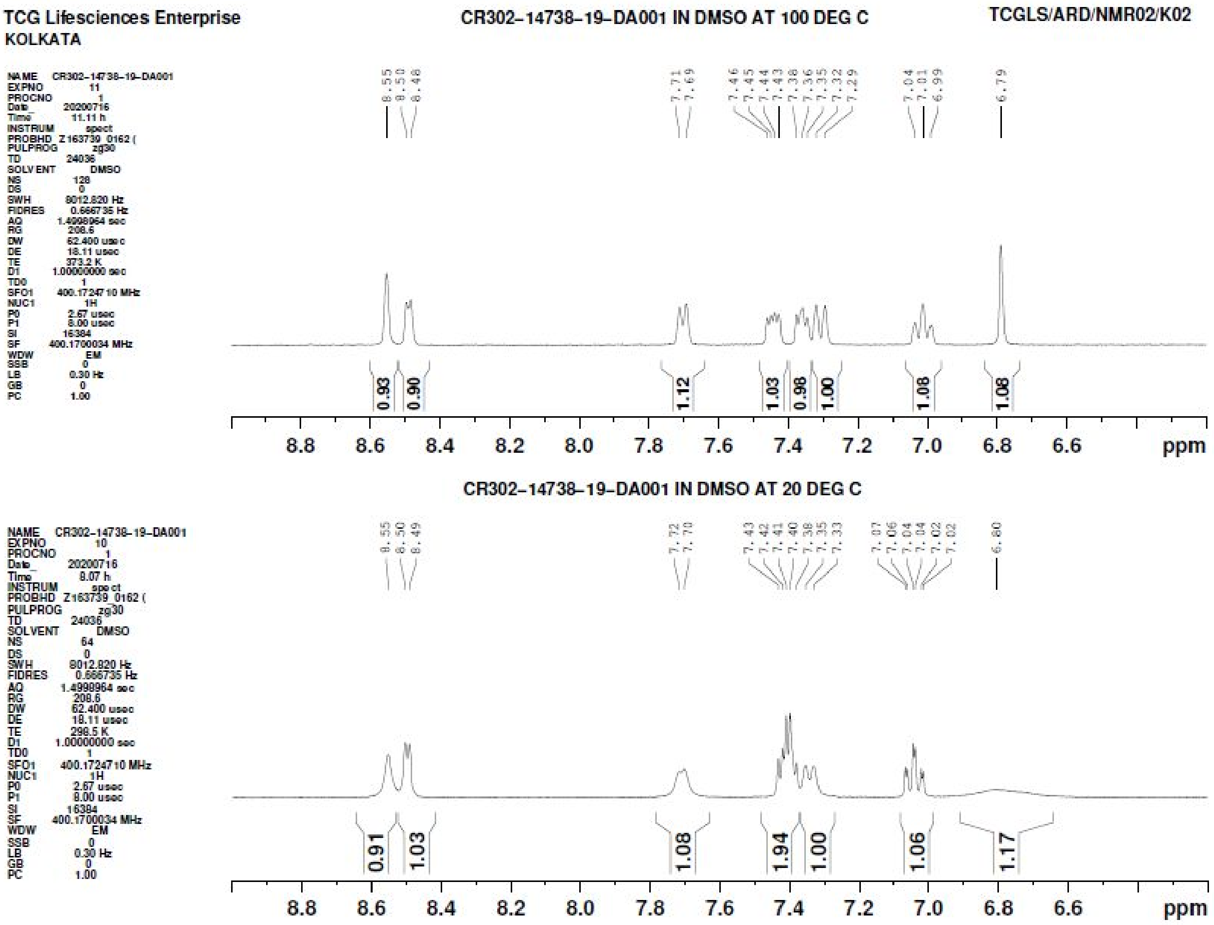

^{1}H NMR (20 °C and100 °C) (400 MHz, DMSO-d$_6$)-6e

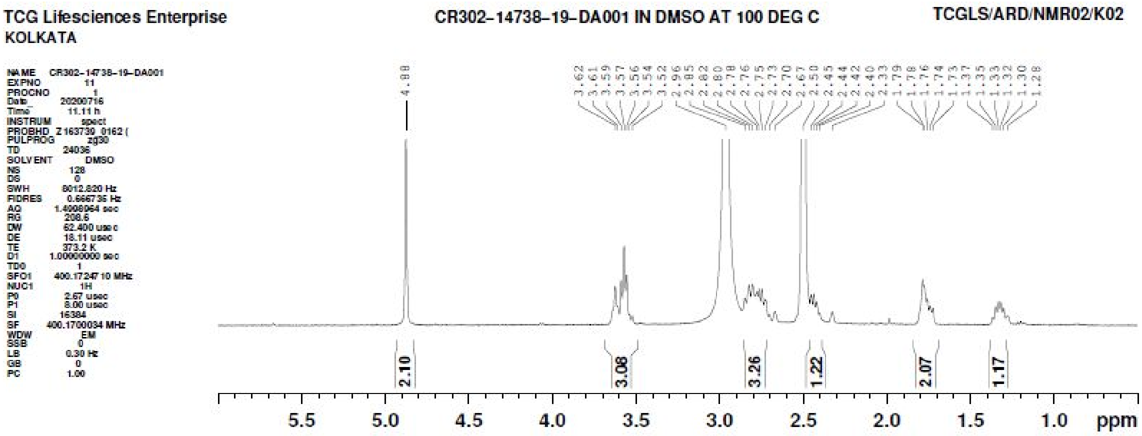

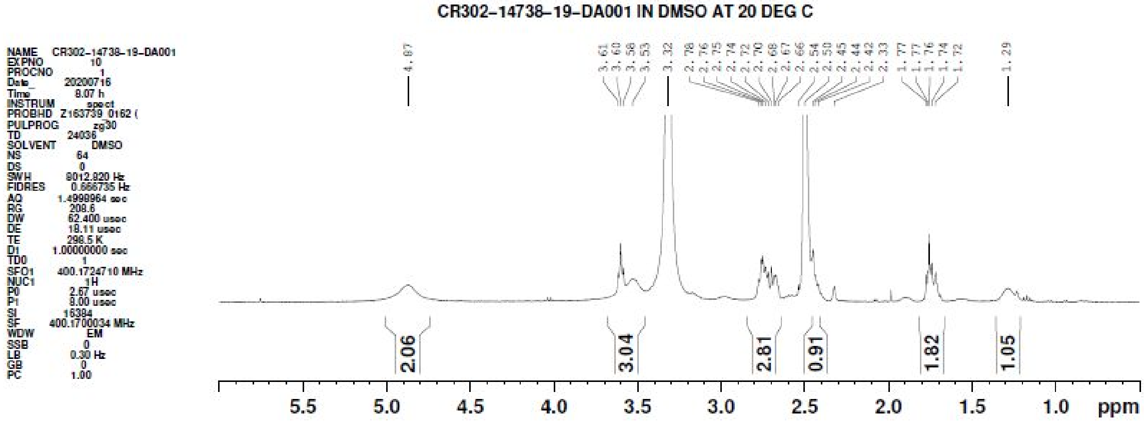

^{1}H NMR (20 °C and100 °C) (400 MHz, DMSO-d_6)-6e

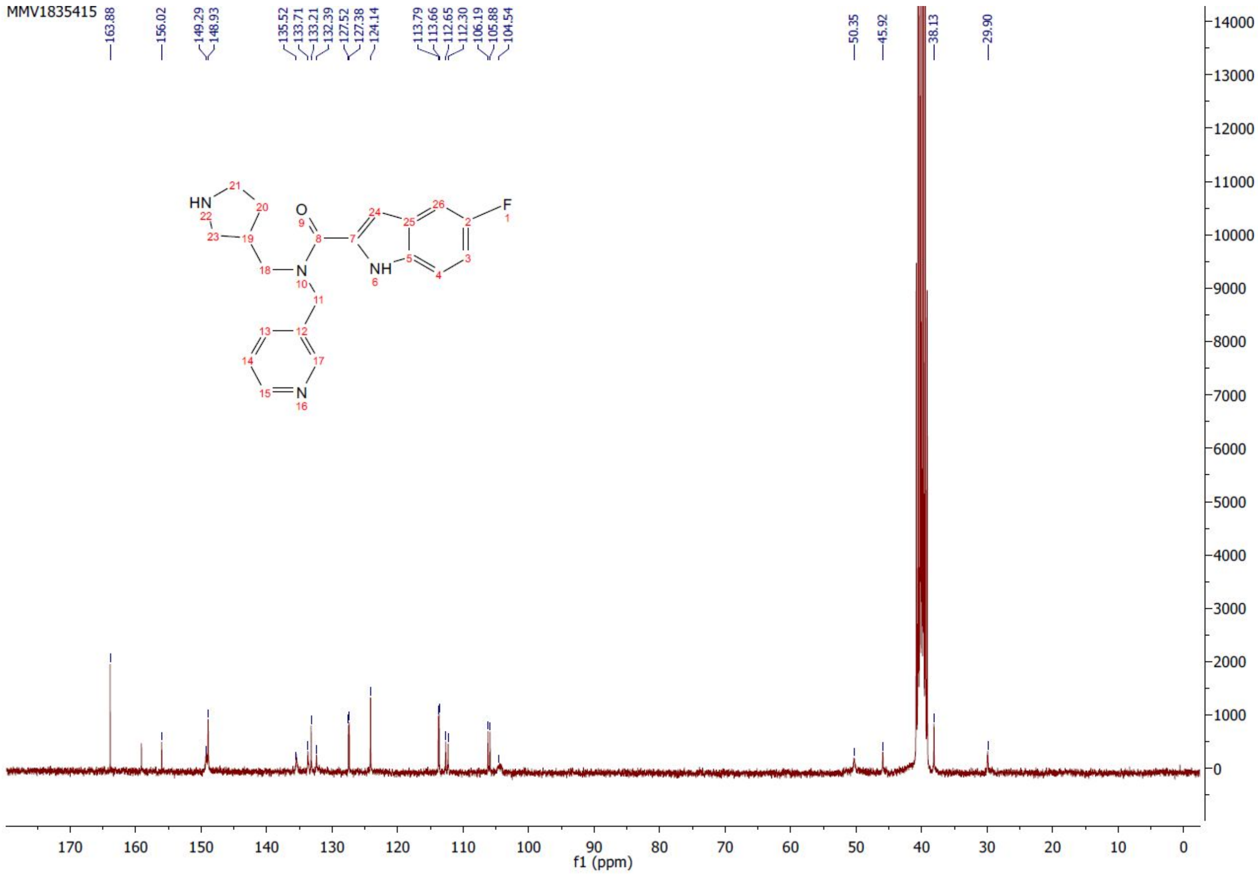

^{13}C NMR (75 MHz, DMSO-d_6)-6e

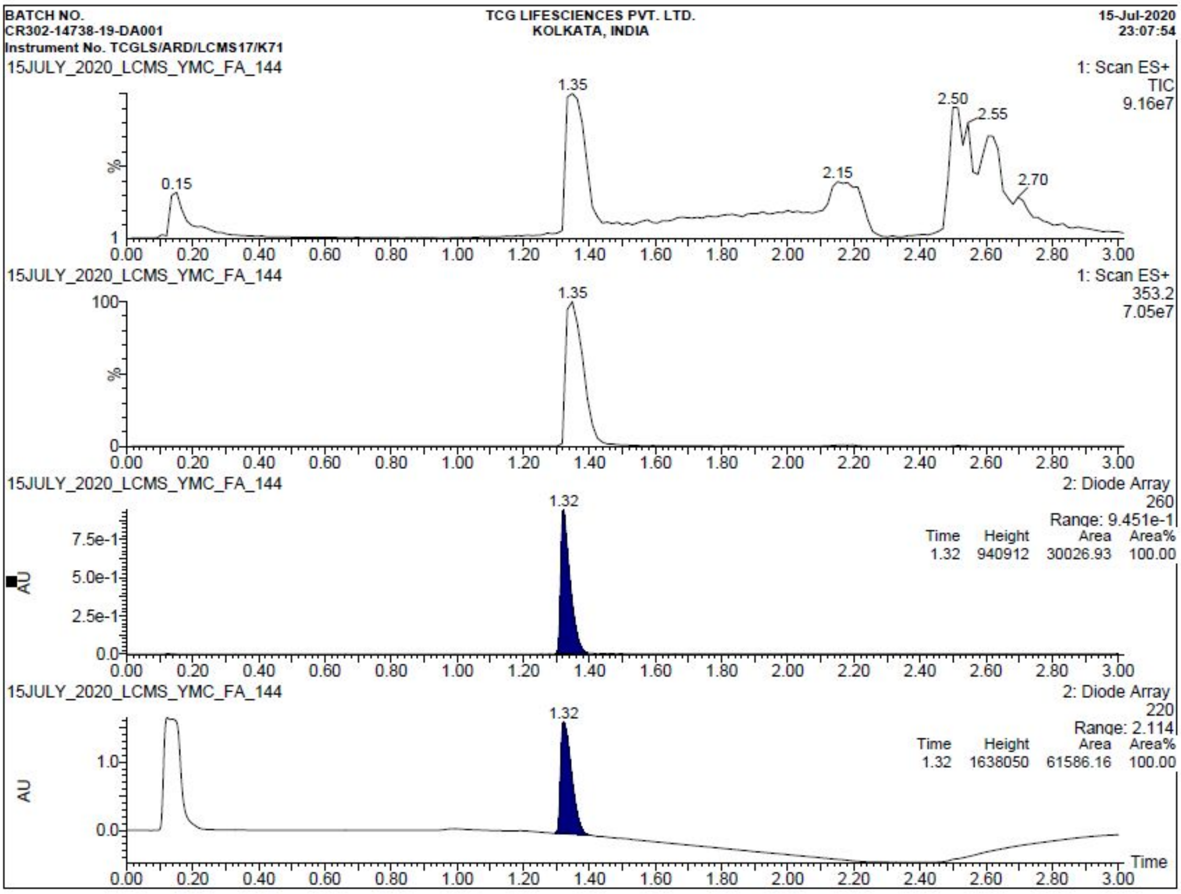
BATCH NO.
CR302-14738-19-DA001
Instrument No. TCGLS/ARD/LCMS17/K71
TCG LIFESCIENCES PVT. LTD.
KOLKATA, INDIA
15-Jul-2020
23:07:54
15JULY_2020_LCMS_YMC_FA_144
1: Scan ES+
TIC
9.16e7
1: Scan ES+
353.2
7.05e7
2: Diode Array
260
Range: 9.451e-1
Time Height Area Area%
1.32 940912 30026.93 100.00
2: Diode Array
220
Range: 2.114
Time Height Area Area%
1.32 1638050 61586.16 100.00
AU
Time

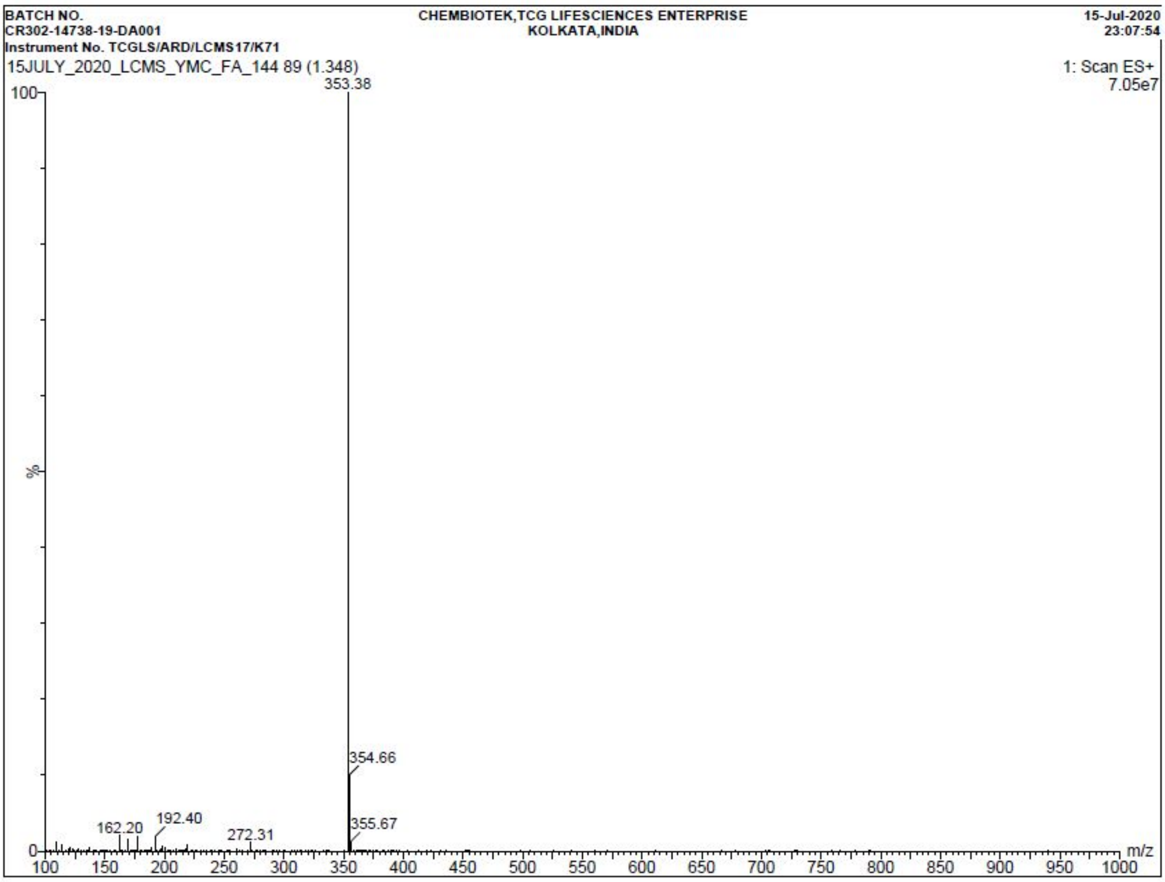
BATCH NO.
CR302-14738-19-DA001
Instrument No. TCGLS/ARD/LCMS17/K71
CHEMBIOTEK,TCG LIFESCIENCES ENTERPRISE
KOLKATA,INDIA
15-Jul-2020
23:07:54
15JULY_2020_LCMS_YMC_FA_144 89 (1.348)
1: Scan ES+
7.05e7
353.38
354.66
355.67
162.20
192.40
272.31
m/z

LCMS-6e

	Resultados HRMS	
	Responsável:	Diego C Andrade
	Data:	06/10/22

Laboratório Institucional de Espectrometria de Massas

Prof. Dr. Luiz Carlos Dias
Aluno Anwar Shamim
Amostra MMV415

Espectro completo de 50 a 750 m/z em modo positivo

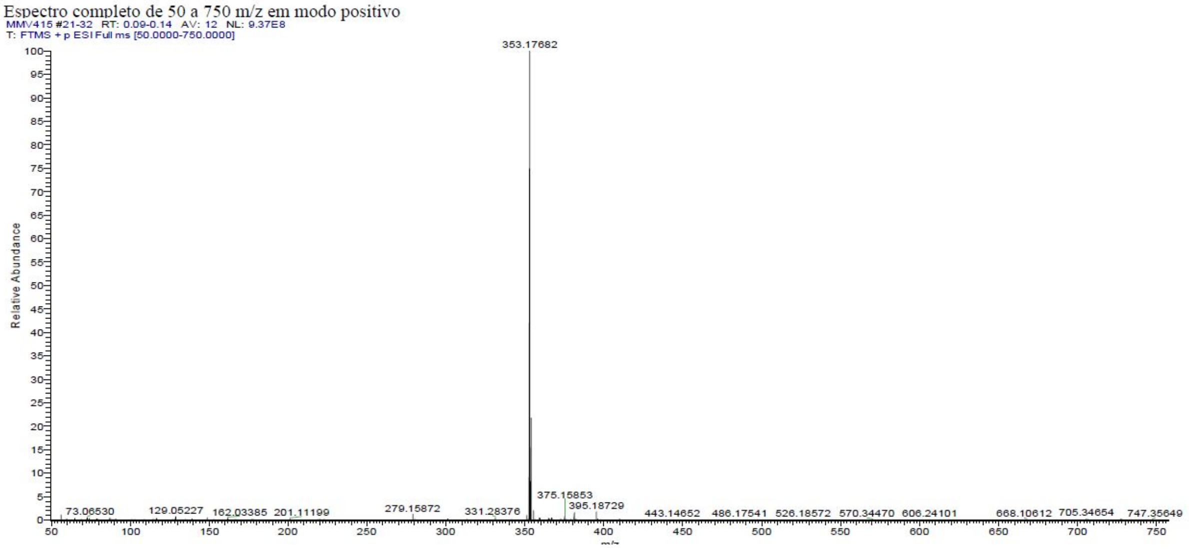

HRMS-6e

	Resultados HRMS	
	Responsável:	Diego C Andrade
	Data:	06/10/22

Laboratório Institucional de Espectrometria de Massas

Comparação entre espectro real (superior) e simulado (inferior) para C20H21FN4OH+

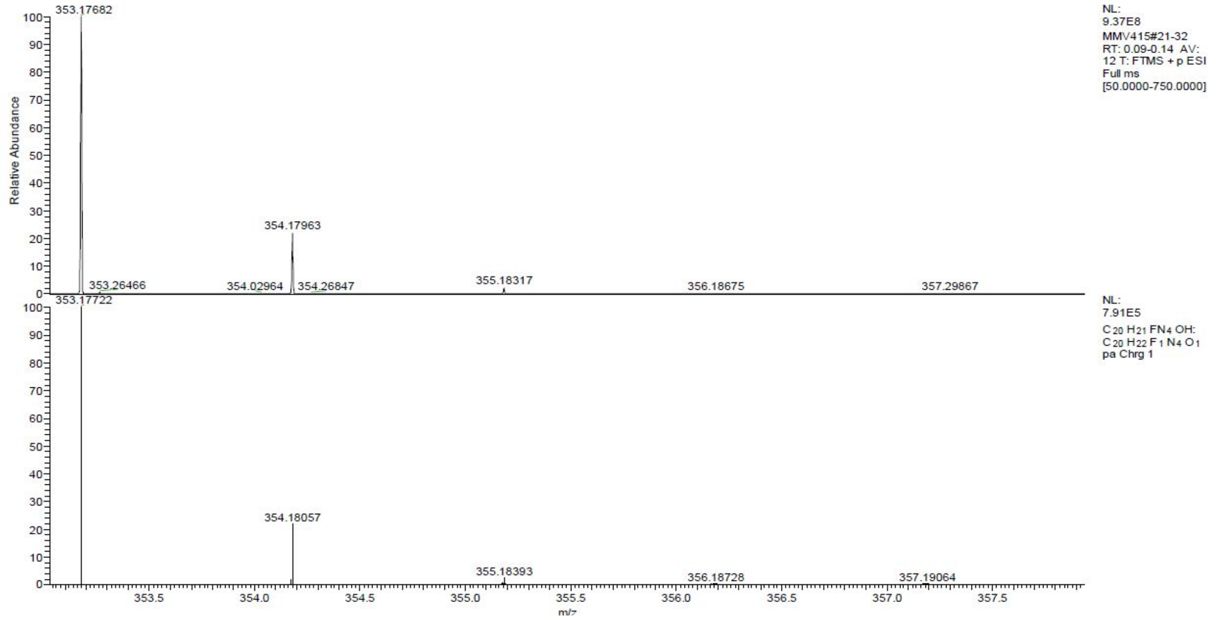

HRMS-6e

N-(3-pyridylmethyl)-N-(pyrrolidin-3-ylmethyl)-5-(trifluoromethyl)-1H-indole-2-carboxamide (6f):

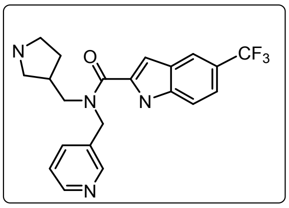

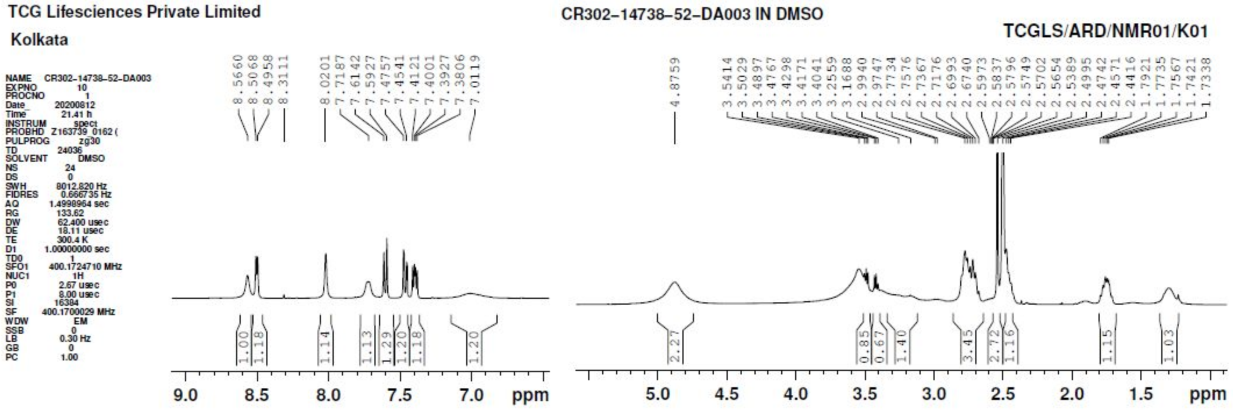

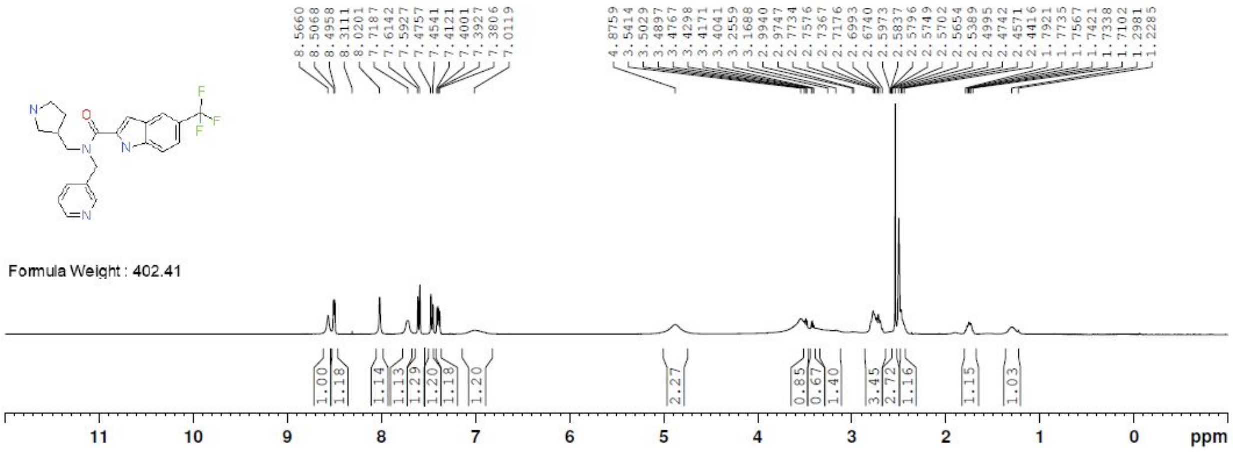

^{1}H NMR (400 MHz, DMSO-d_6)-6f

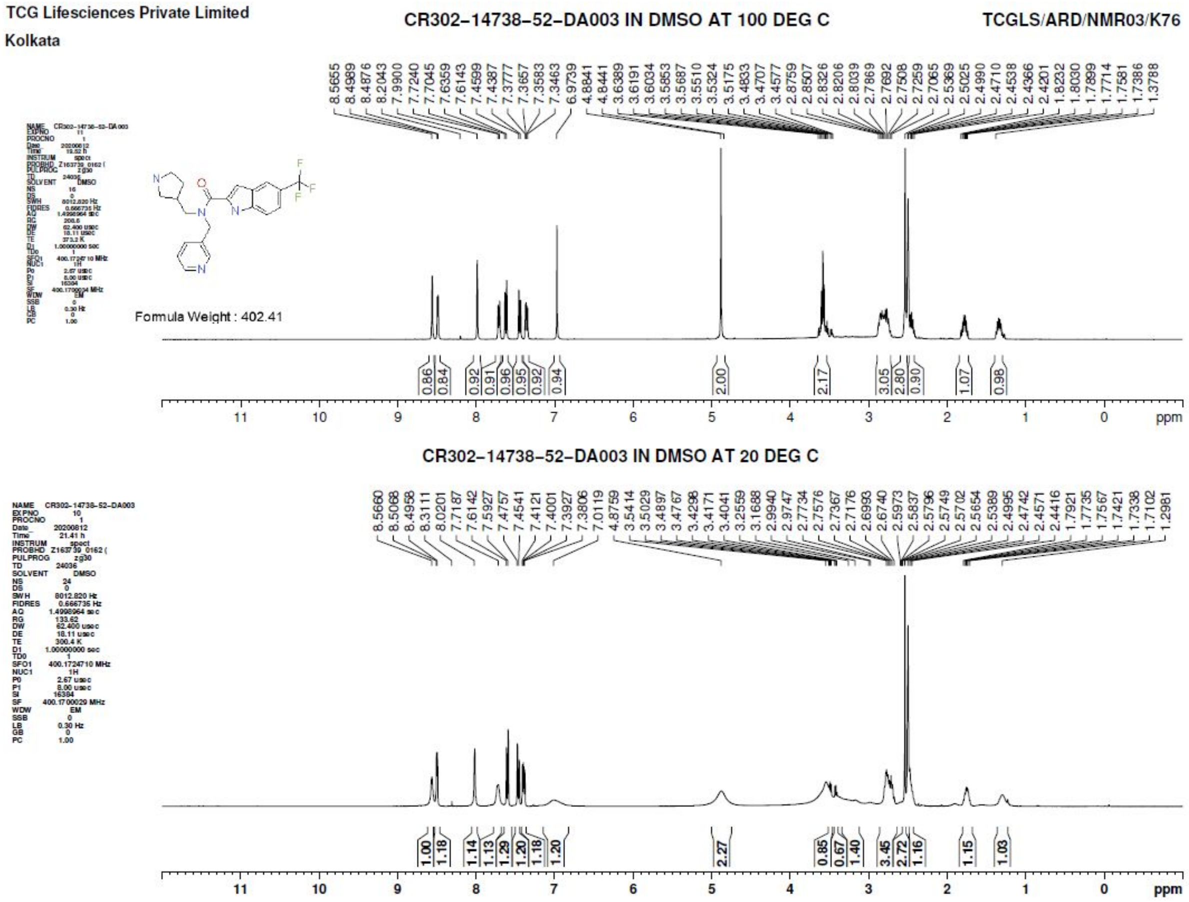

^{1}H NMR (20 °C and100 °C) (400 MHz, DMSO-d$_6$)-6f

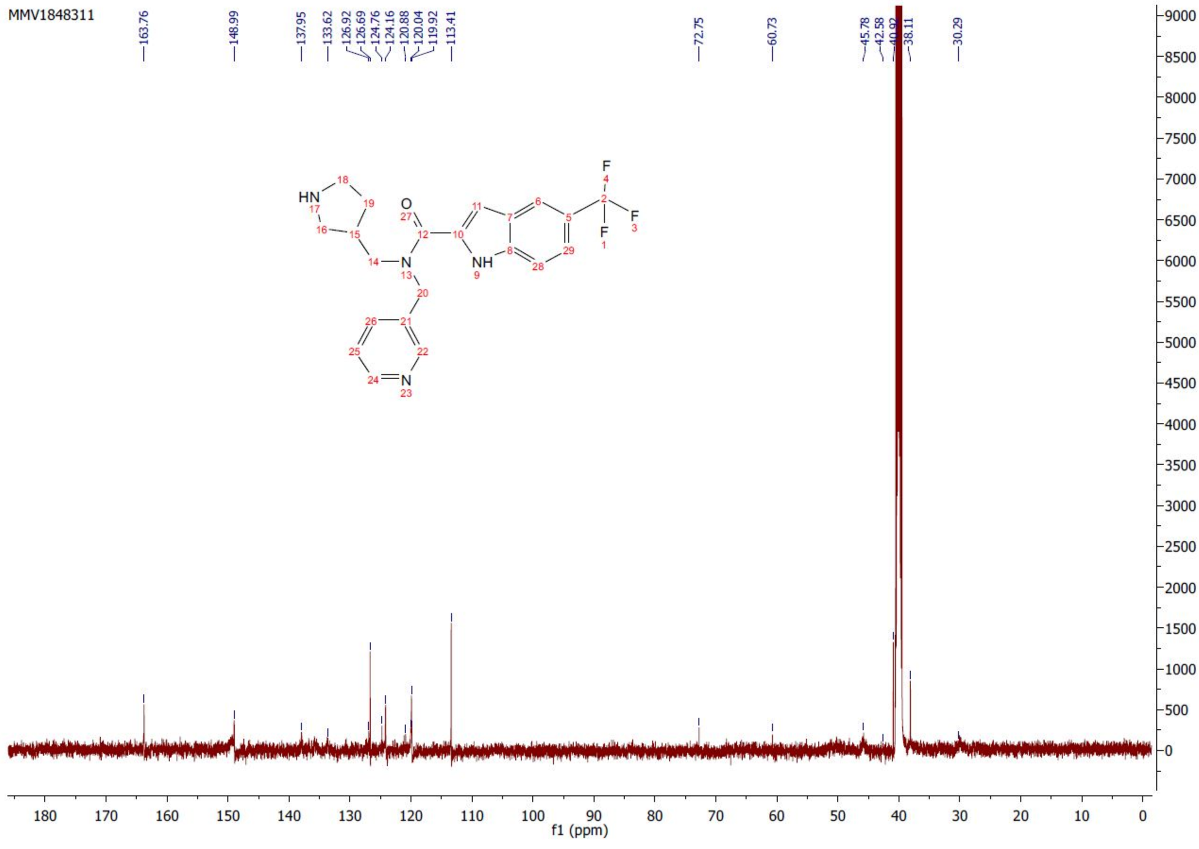

^{13}C NMR (125 MHz, DMSO-d$_6$)-6f

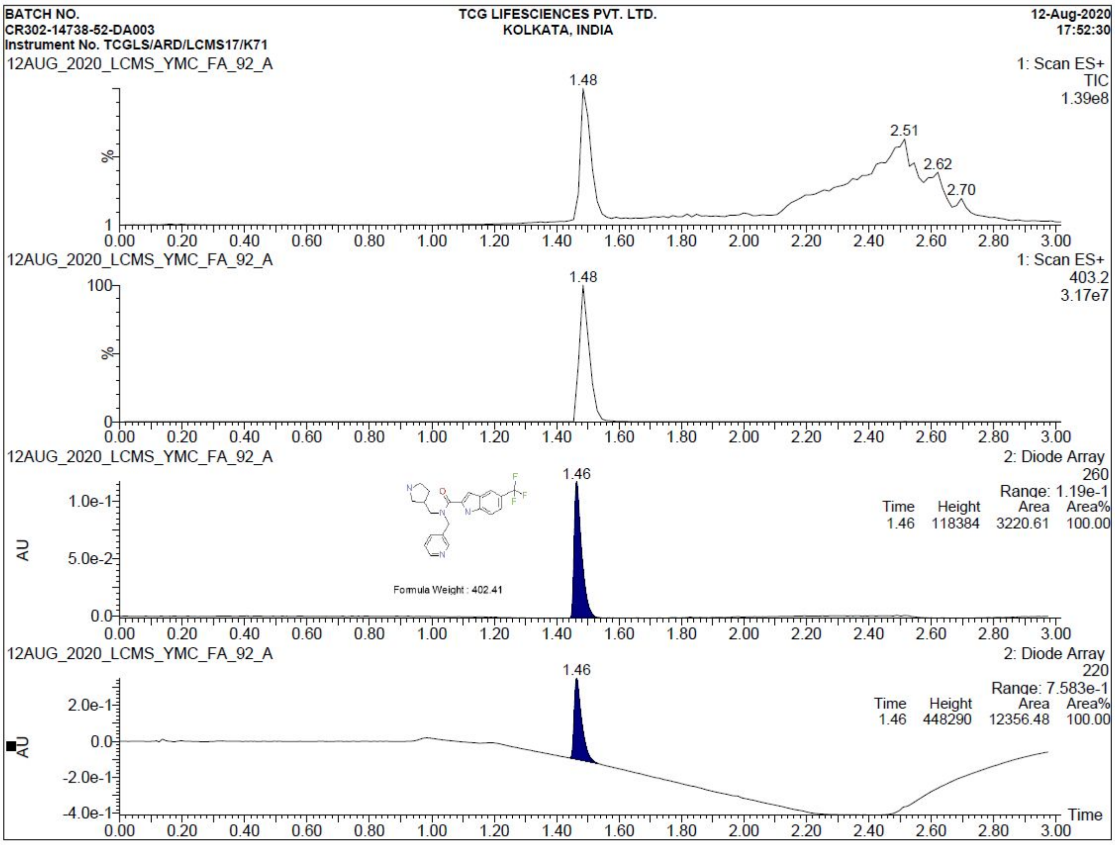
BATCH NO.
CR302-14738-52-DA003
Instrument No. TCGLS/ARD/LCMS17/K71
TCG LIFESCIENCES PVT. LTD.
KOLKATA, INDIA
12-Aug-2020
17:52:30
12AUG_2020_LCMS_YMC_FA_92_A
1: Scan ES+
TIC
1.39e8
1: Scan ES+
403.2
3.17e7
2: Diode Array
260
Range: 1.19e-1
Time Height Area Area%
1.46 118384 3220.61 100.00
Formula Weight : 402.41
2: Diode Array
220
Range: 7.583e-1
Time Height Area Area%
1.46 448290 12356.48 100.00
Time

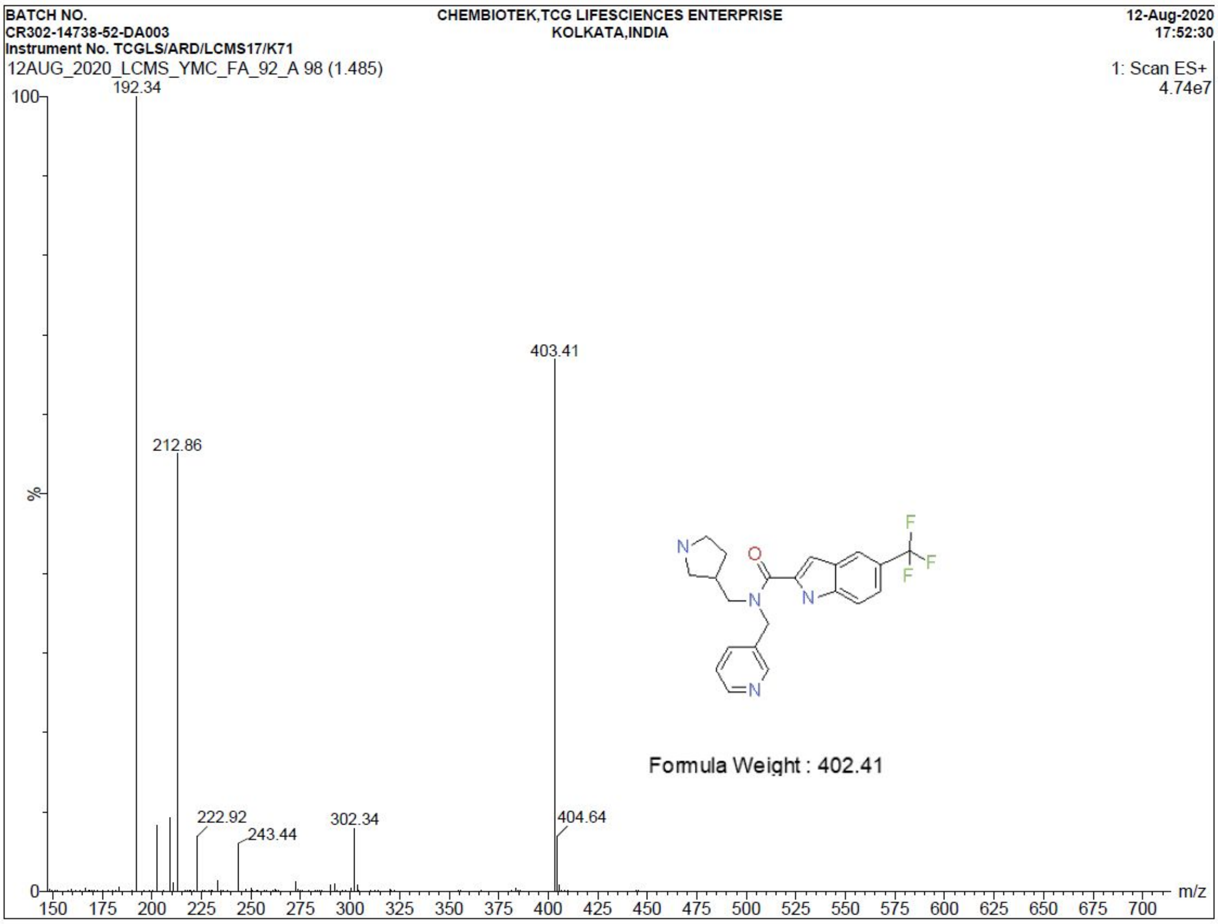
BATCH NO.
CR302-14738-52-DA003
Instrument No. TCGLS/ARD/LCMS17/K71
CHEMBIOTEK,TCG LIFESCIENCES ENTERPRISE
KOLKATA,INDIA
12-Aug-2020
17:52:30
12AUG_2020_LCMS_YMC_FA_92_A 98 (1.485)
1: Scan ES+
4.74e7
192.34
403.41
212.86
222.92
243.44
302.34
404.64
Formula Weight : 402.41
m/z

LCMS-6f

	Resultados HRMS		
	Responsável:	Diego C Andrade	
	Data:	03/11/22	
	**Laboratório Institucional de Espectrometria de Massas**		

Prof. Dr. Luiz Carlos Dias
Aluna Mariana Ferrer
Amostra MMV311

Espectro completo de 80 a 750 m/z em modo positivo

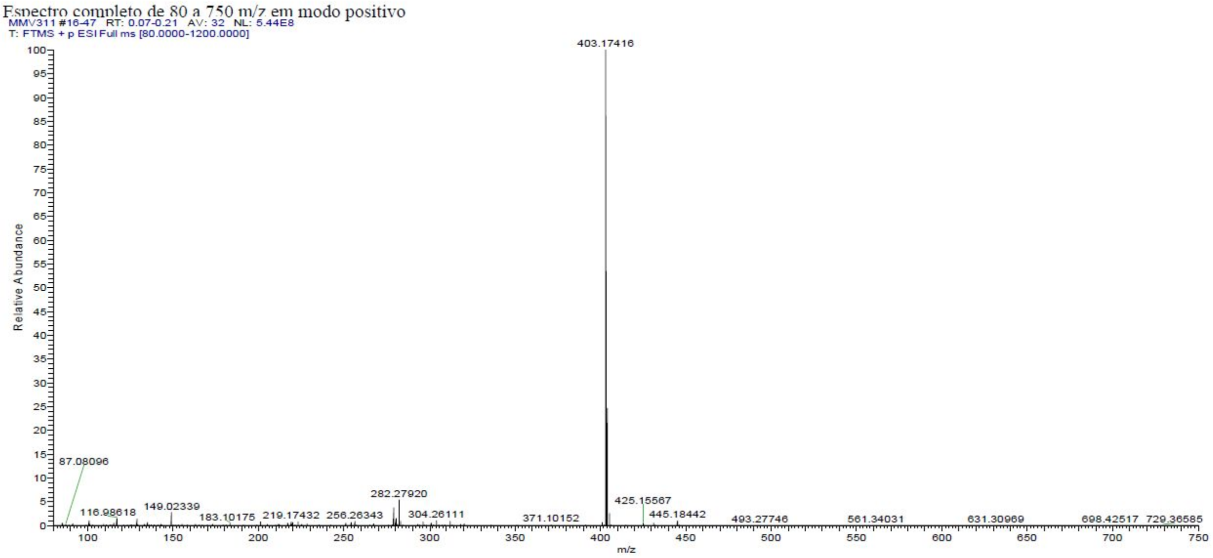

HRMS-6f

	Resultados HRMS		
	Responsável:	Diego C Andrade	
	Data:	03/11/22	
	**Laboratório Institucional de Espectrometria de Massas**		

Comparação entre espectro real (superior) e simulado (inferior) para C21H21F3N4OH+

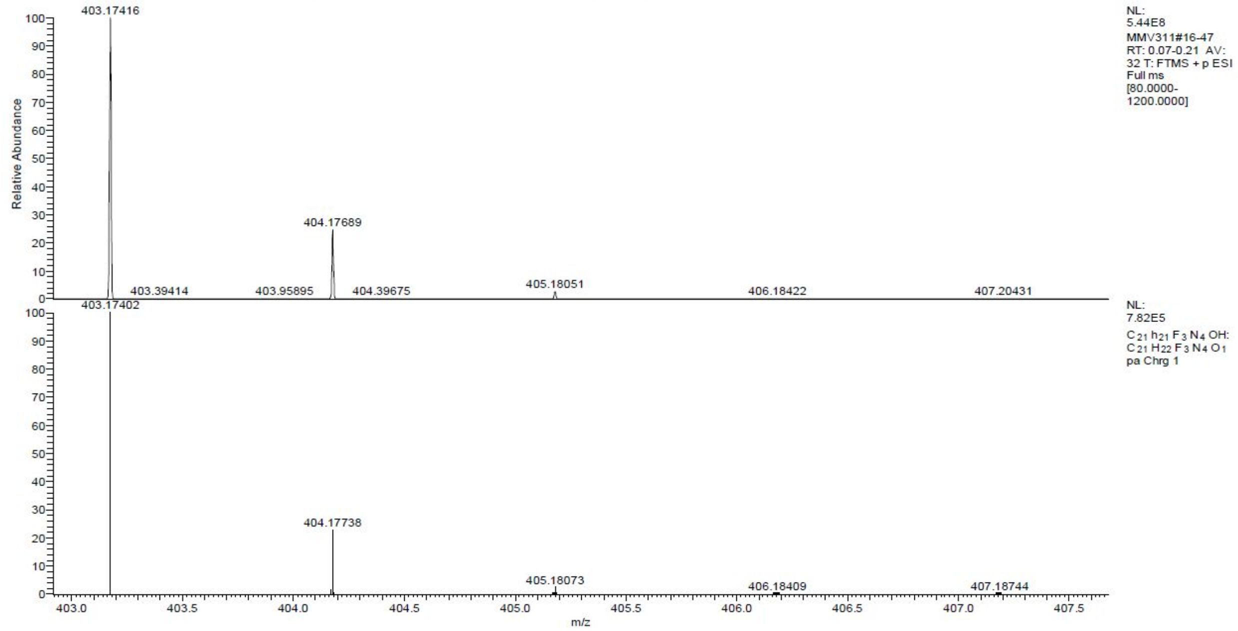

HRMS-6f

5-chloro-3-methyl-N-(3-pyridylmethyl)-N-(pyrrolidin-3-ylmethyl)-1H-indole-2-carboxamide (6g):

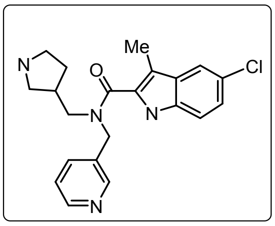

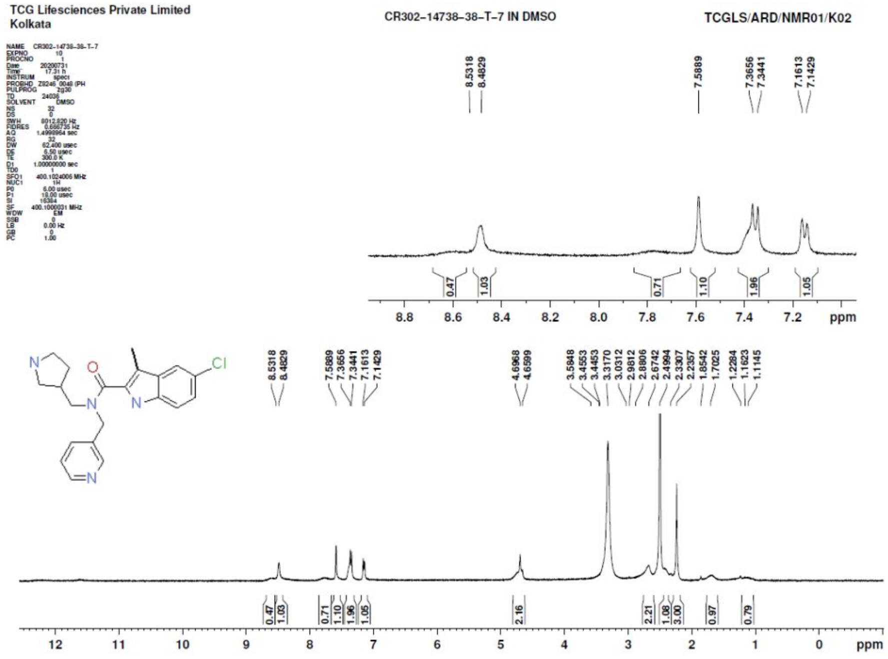

^{1}H NMR (400 MHz, DMSO-d$_6$)-6g

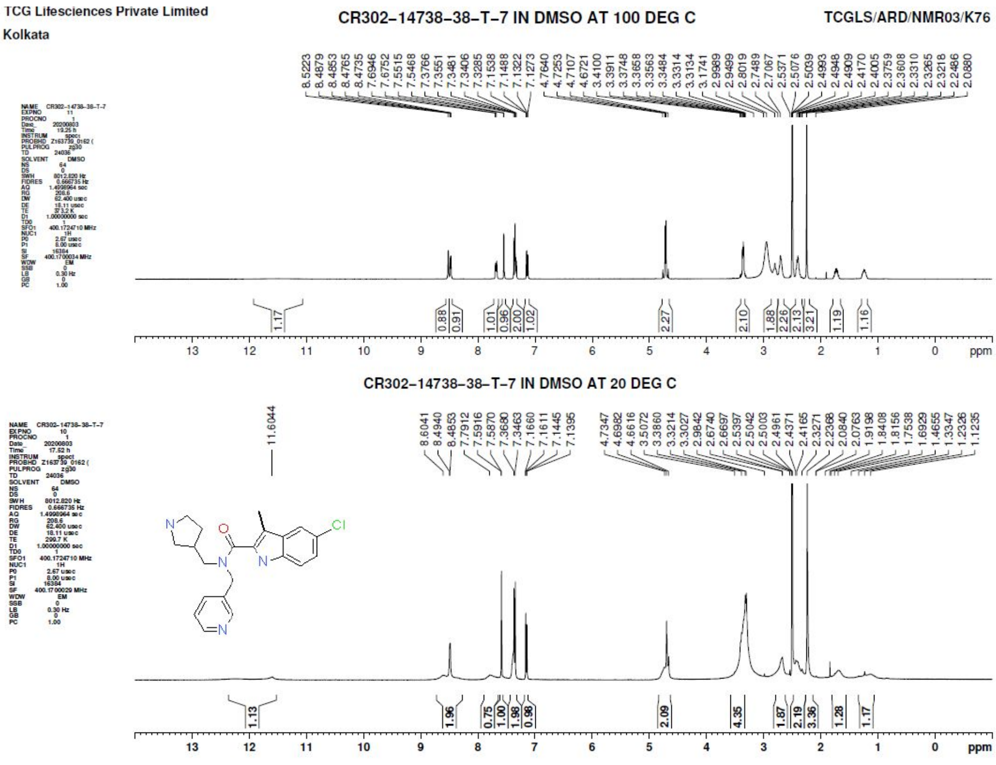

^{1}H NMR (20 °C and100 °C) (400 MHz, DMSO-d$_6$)-6g

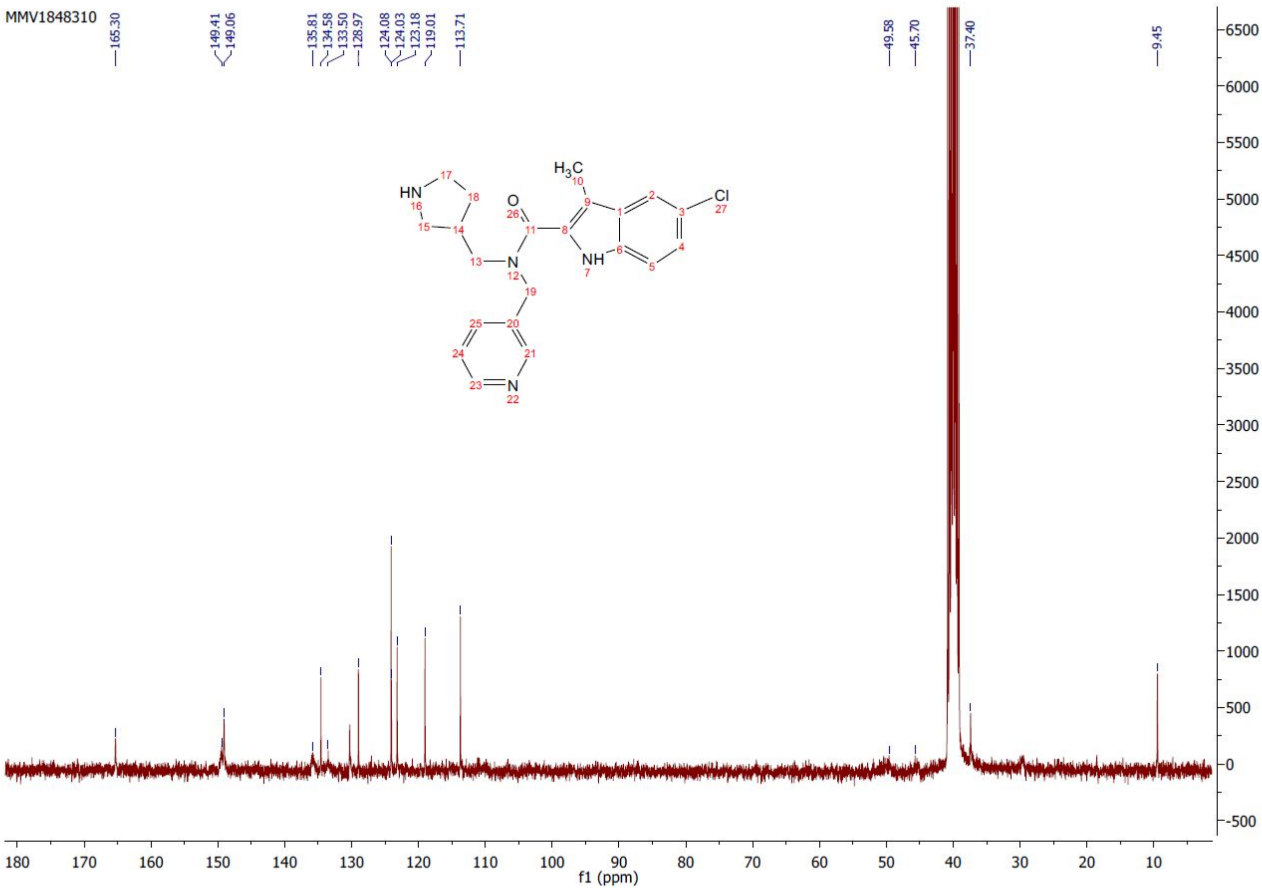

^{13}C NMR (75 MHz, DMSO-d$_6$)-6g

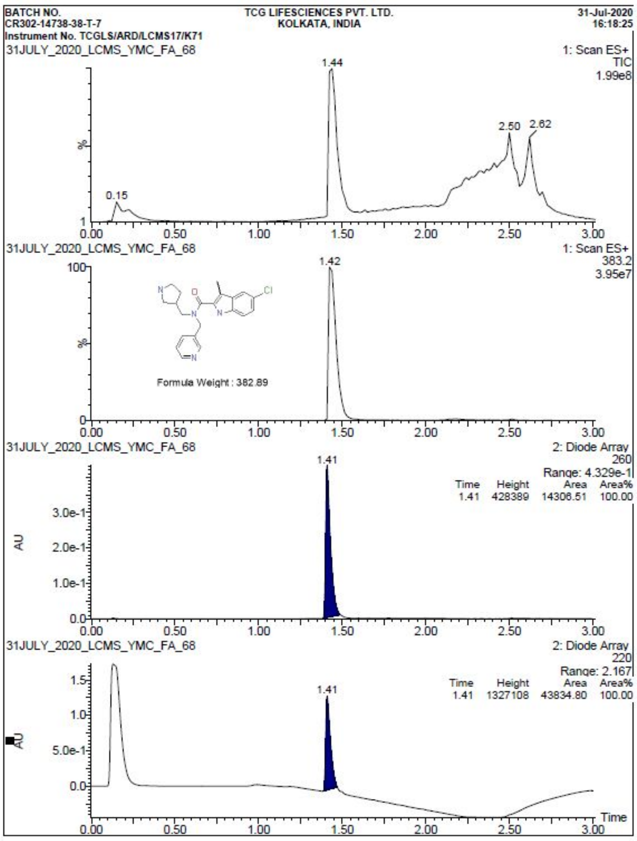

LCMS-6g

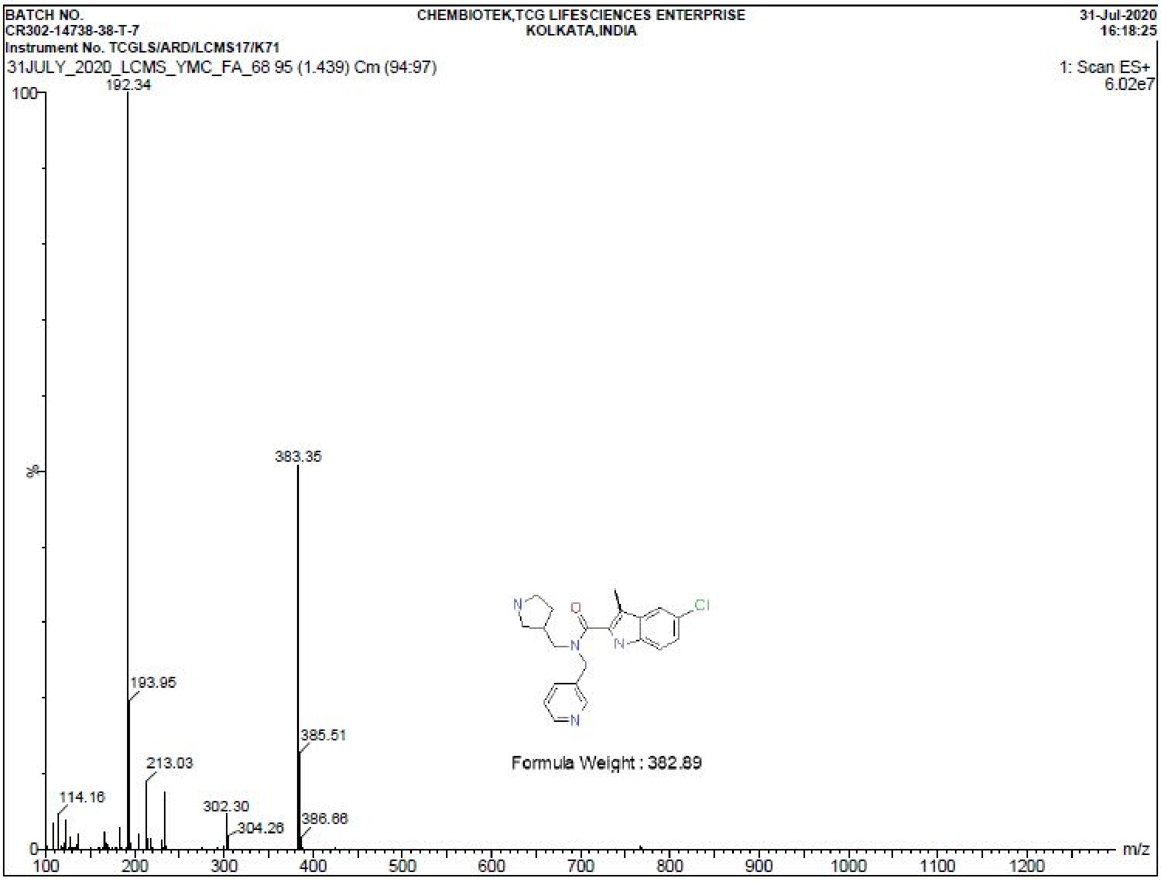

LCMS-6g

	Resultados HRMS		
Instituto de Química	Responsável:	Diego C Andrade	LIE Laboratório Institucional de Espectrometria de Massas
	Data:	06/10/22	
	**Laboratório Institucional de Espectrometria de Massas**		

Prof. Dr. Luiz Carlos Dias
Aluno Anwar Shamim
Amostra MMV310

Espectro completo de 50 a 750 m/z em modo positivo

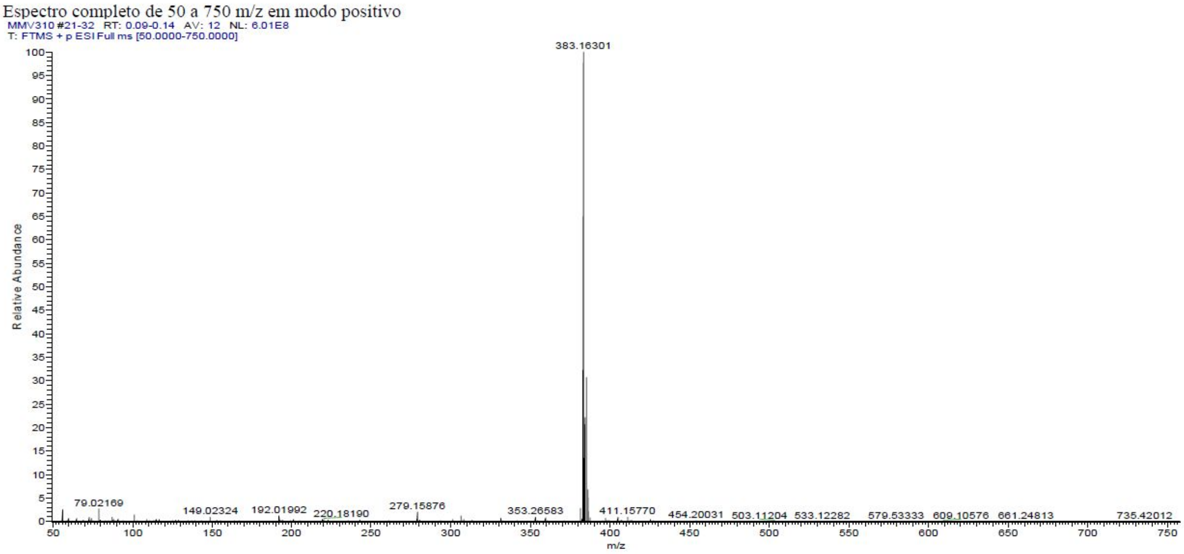

HRMS-6g

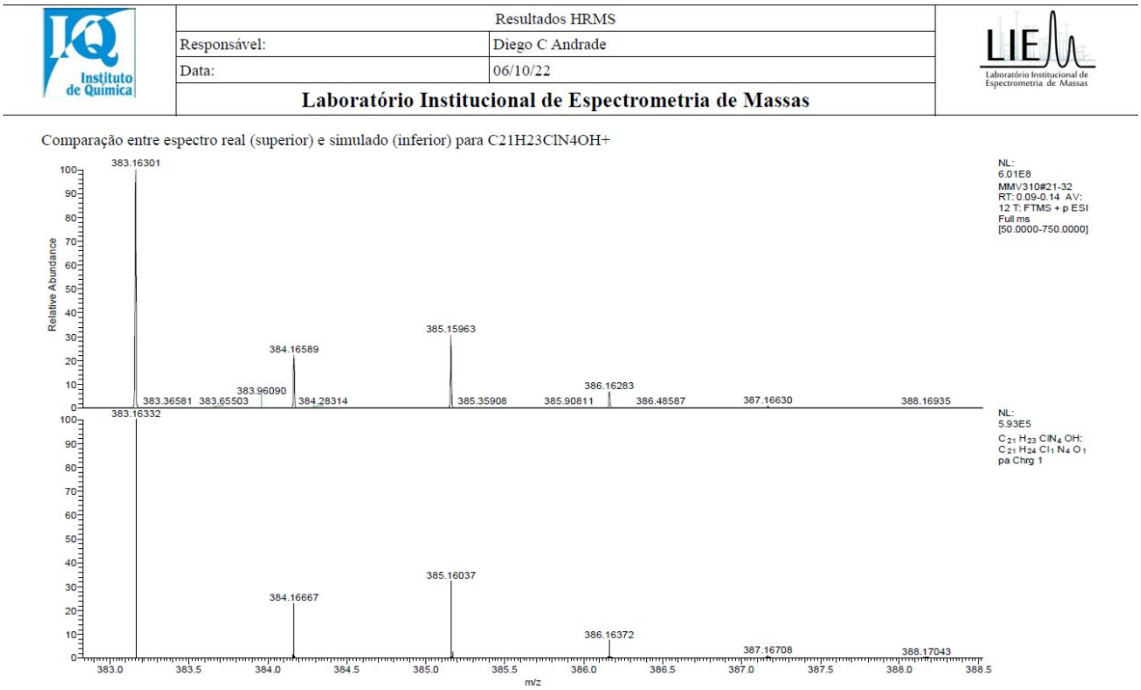

HRMS-6g

5-chloro-N-(3-pyridylmethyl)-N-(pyrrolidin-3-ylmethyl)-1H-indole-3-carboxamide (6h):

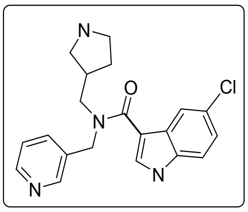

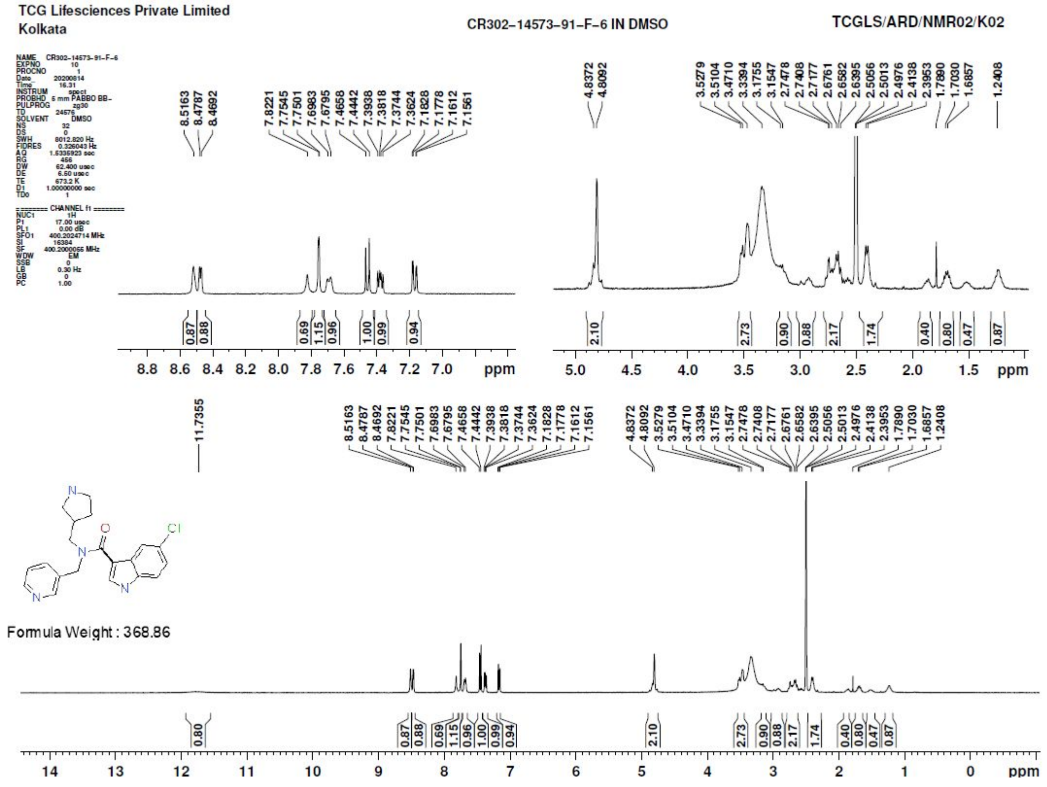

^{1}H NMR (400 MHz, DMSO-d_6)-6h

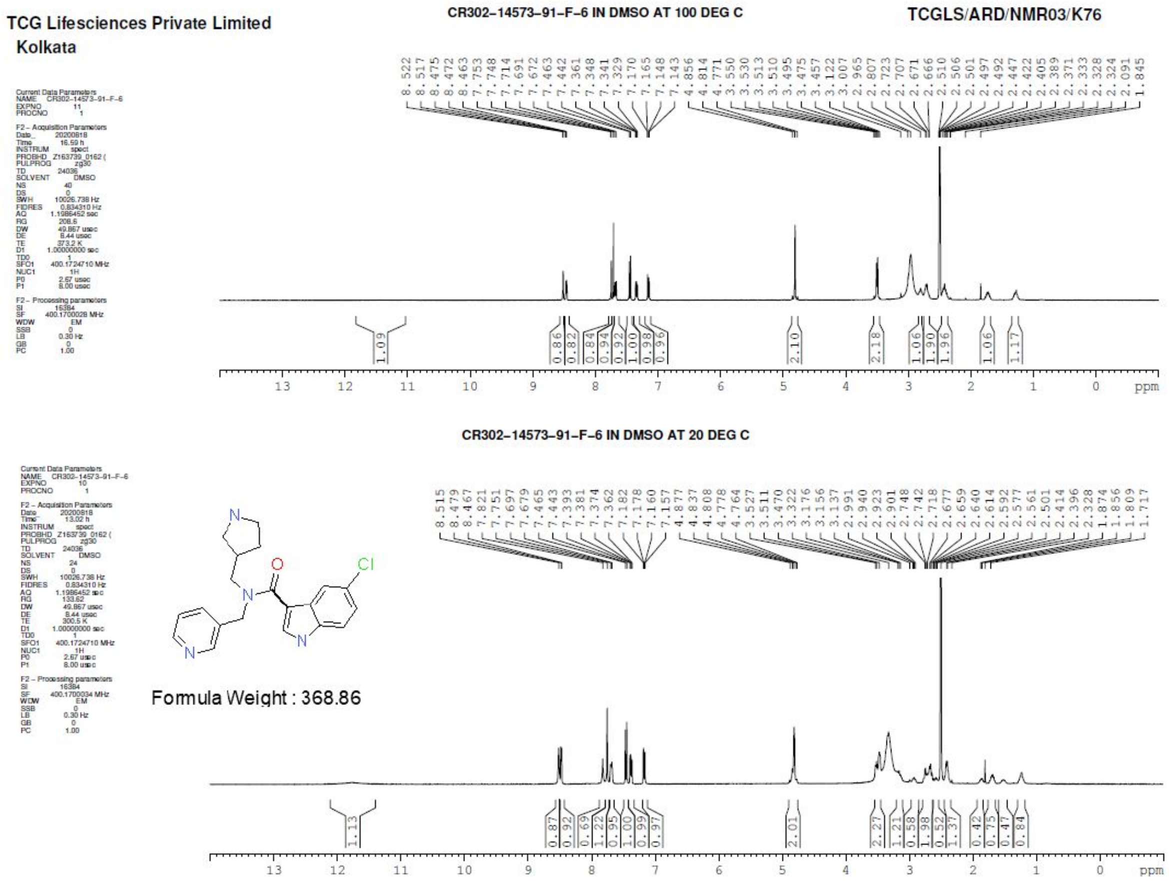

¹H NMR (20 °C and100 °C) (400 MHz, DMSO-d$_6$)-6h

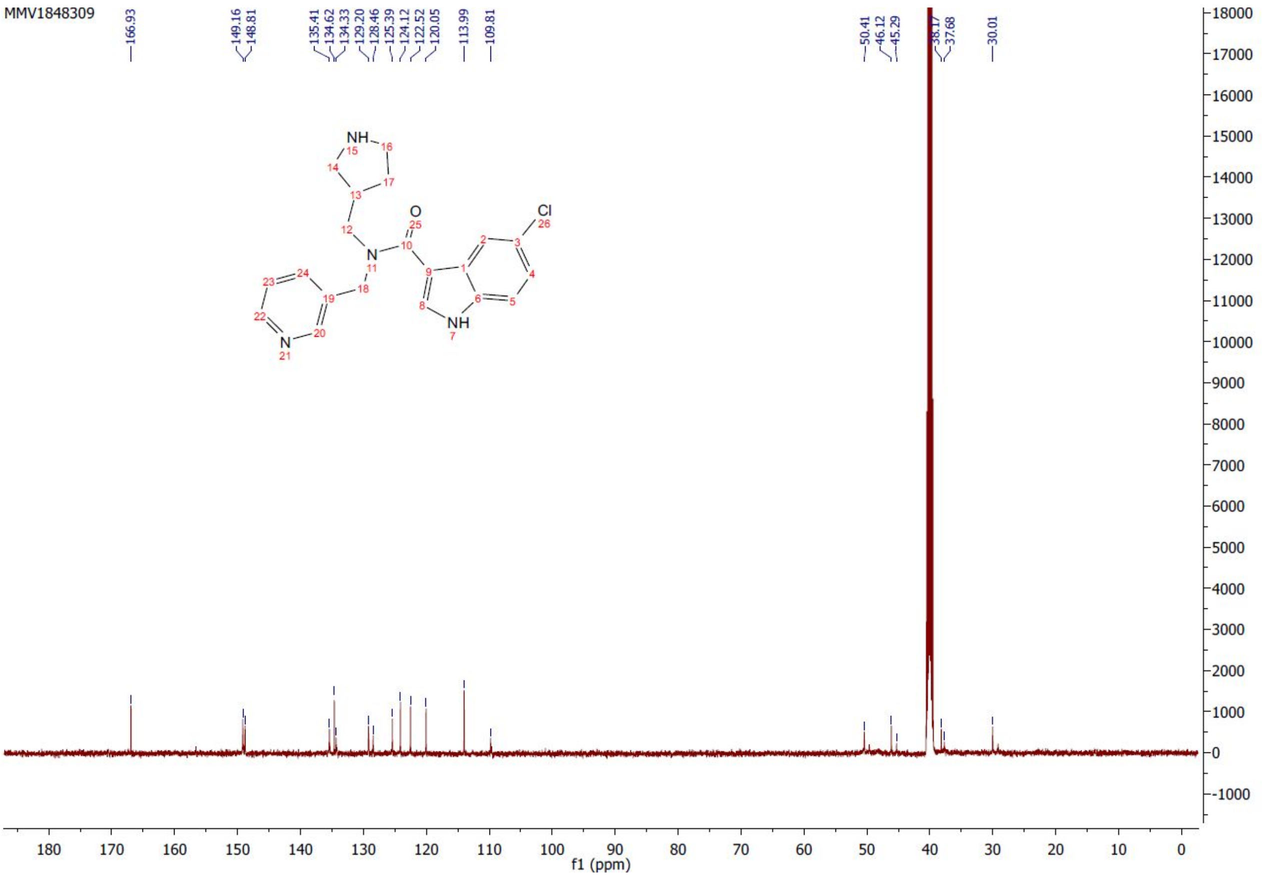

¹³C NMR (125 MHz, DMSO-d$_6$)-6h

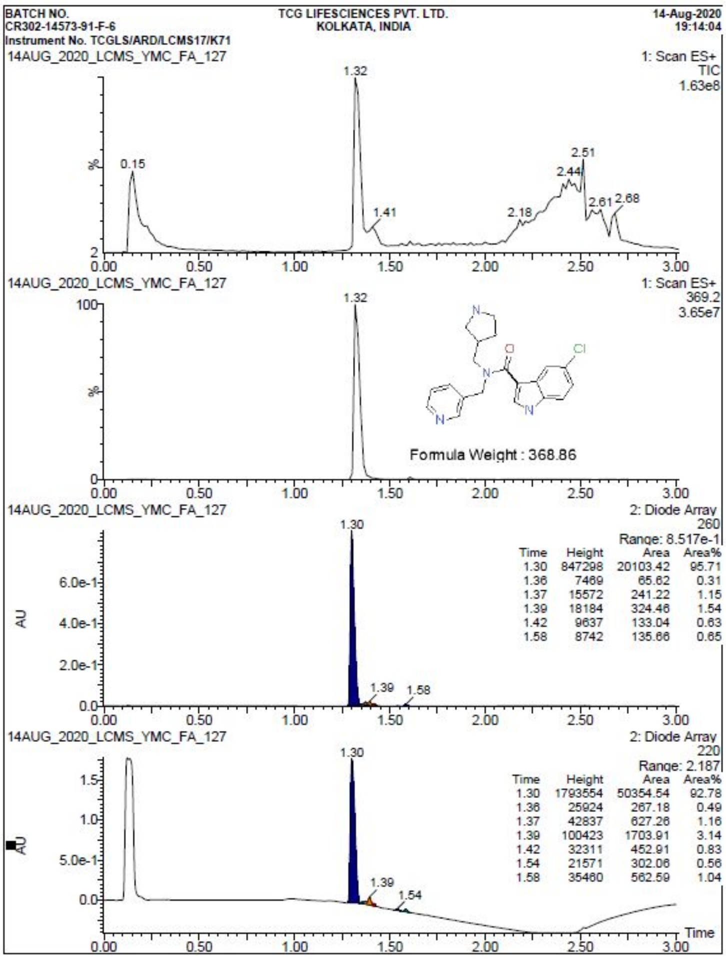
BATCH NO.
CR302-14573-91-F-6
Instrument No. TCGLS/ARD/LCMS17/K71
TCG LIFESCIENCES PVT. LTD.
KOLKATA, INDIA
14-Aug-2020
19:14:04
14AUG_2020_LCMS_YMC_FA_127
1: Scan ES+
TIC
1.63e8
1: Scan ES+
369.2
3.65e7
Formula Weight : 368.86
2: Diode Array
260
Range: 8.517e-1
Time Height Area Area%
1.30 847298 20103.42 95.71
1.36 7469 65.62 0.31
1.37 15572 241.22 1.15
1.39 18184 324.46 1.54
1.42 9637 133.04 0.63
1.58 8742 135.66 0.65
2: Diode Array
220
Range: 2.187
Time Height Area Area%
1.30 1793554 50354.54 92.78
1.36 25924 267.18 0.49
1.37 42837 627.28 1.16
1.39 100423 1703.91 3.14
1.42 32311 452.91 0.83
1.54 21571 302.06 0.56
1.58 35460 562.59 1.04
Time

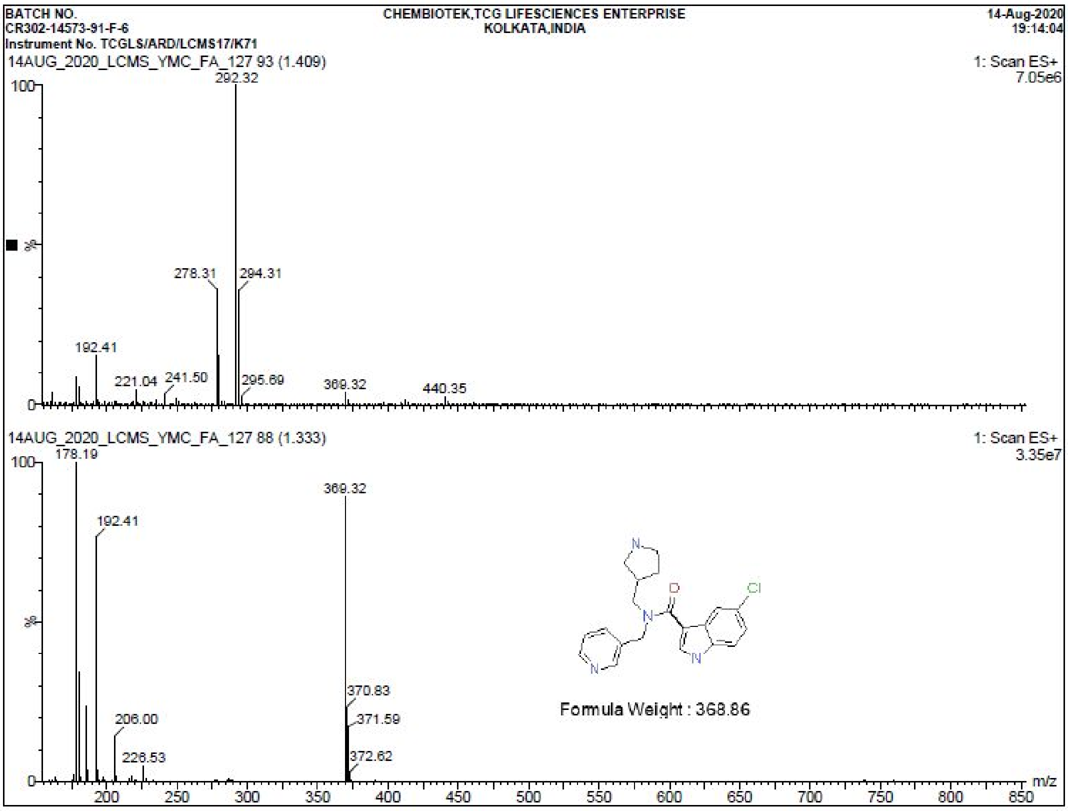
BATCH NO.
CR302-14573-91-F-6
Instrument No. TCGLS/ARD/LCMS17/K71
CHEMBIOTEK,TCG LIFESCIENCES ENTERPRISE
KOLKATA,INDIA
14-Aug-2020
19:14:04
14AUG_2020_LCMS_YMC_FA_127 93 (1.409)
1: Scan ES+
7.05e6
14AUG_2020_LCMS_YMC_FA_127 88 (1.333)
1: Scan ES+
3.35e7
Formula Weight : 368.86
m/z

LCMS-6h

	Resultados HRMS	
	Responsável:	Diego C Andrade
	Data:	03/11/22

Laboratório Institucional de Espectrometria de Massas

Prof. Dr. Luiz Carlos Dias
Aluna Mariana Ferrer
Amostra MMV309

Espectro completo de 80 a 750 m/z em modo positivo

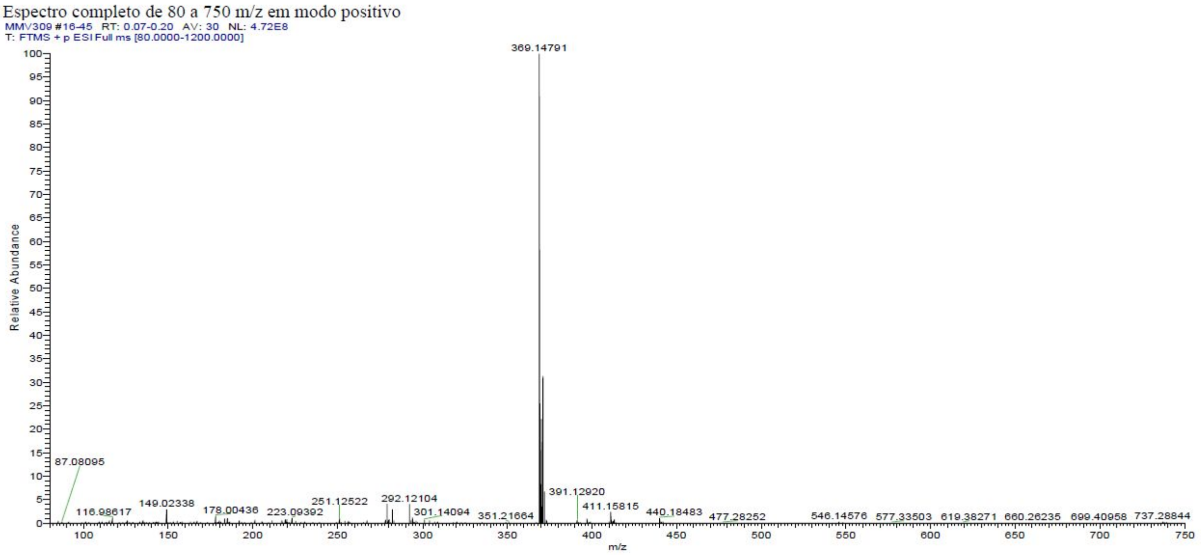

HRMS-6h

	Resultados HRMS	
	Responsável:	Diego C Andrade
	Data:	03/11/22

Laboratório Institucional de Espectrometria de Massas

Comparação entre espectro real (superior) e simulado (inferior) para C20H21ClN4OH+

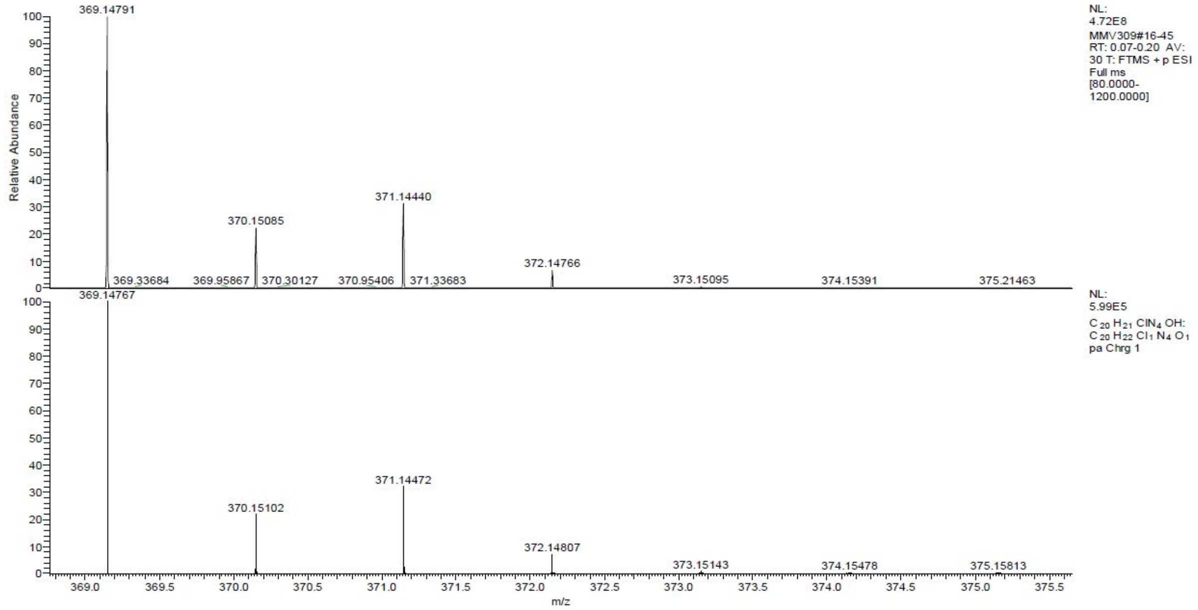

HRMS-6h

5-chloro-N-(3-pyridylmethyl)-N-(pyrrolidin-3-ylmethyl)-1H-benzimidazole-2-carboxamide (6i):

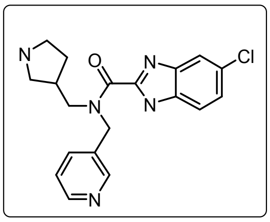

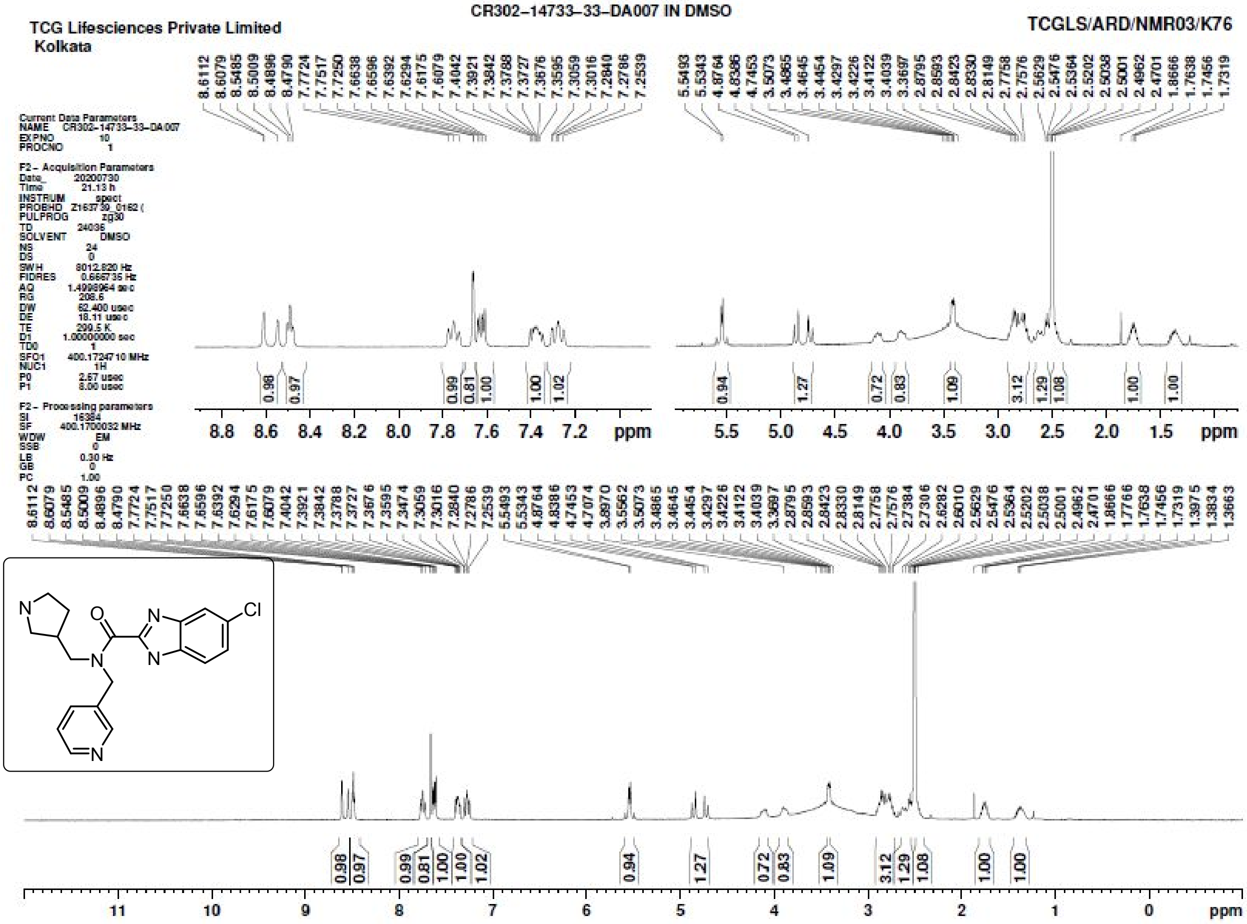

^{1}H NMR (400 MHz, DMSO-d$_6$)-6i

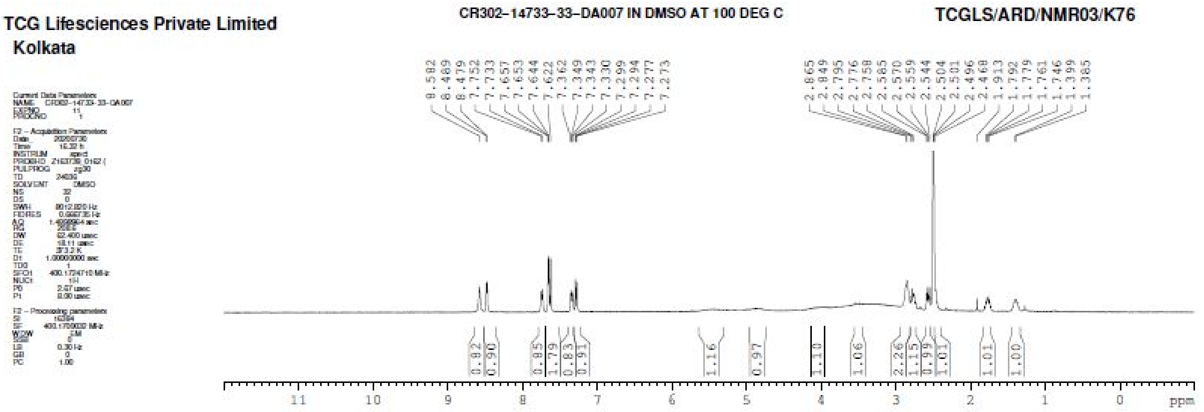

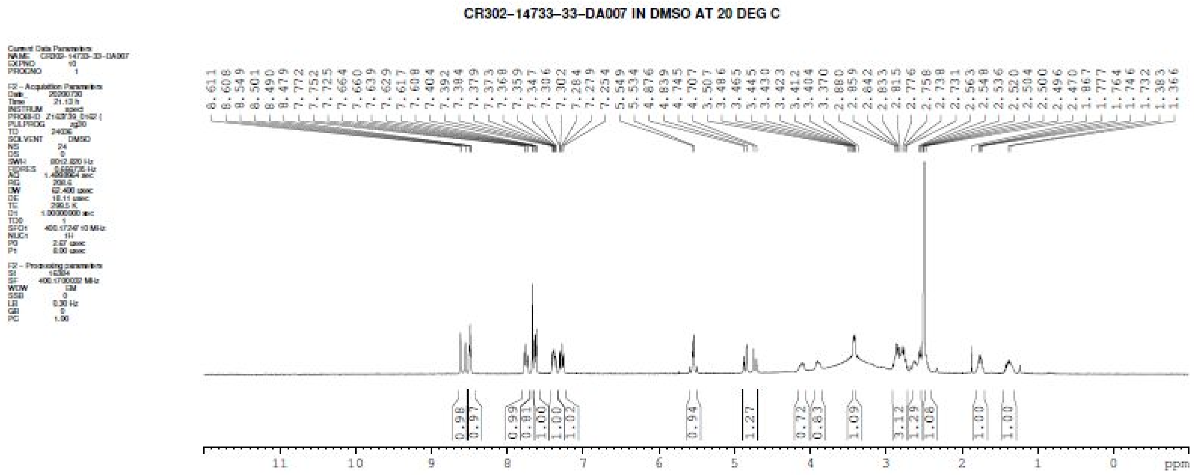

^{1}H NMR (20 °C and100 °C) (400 MHz, DMSO-d_6)-6i

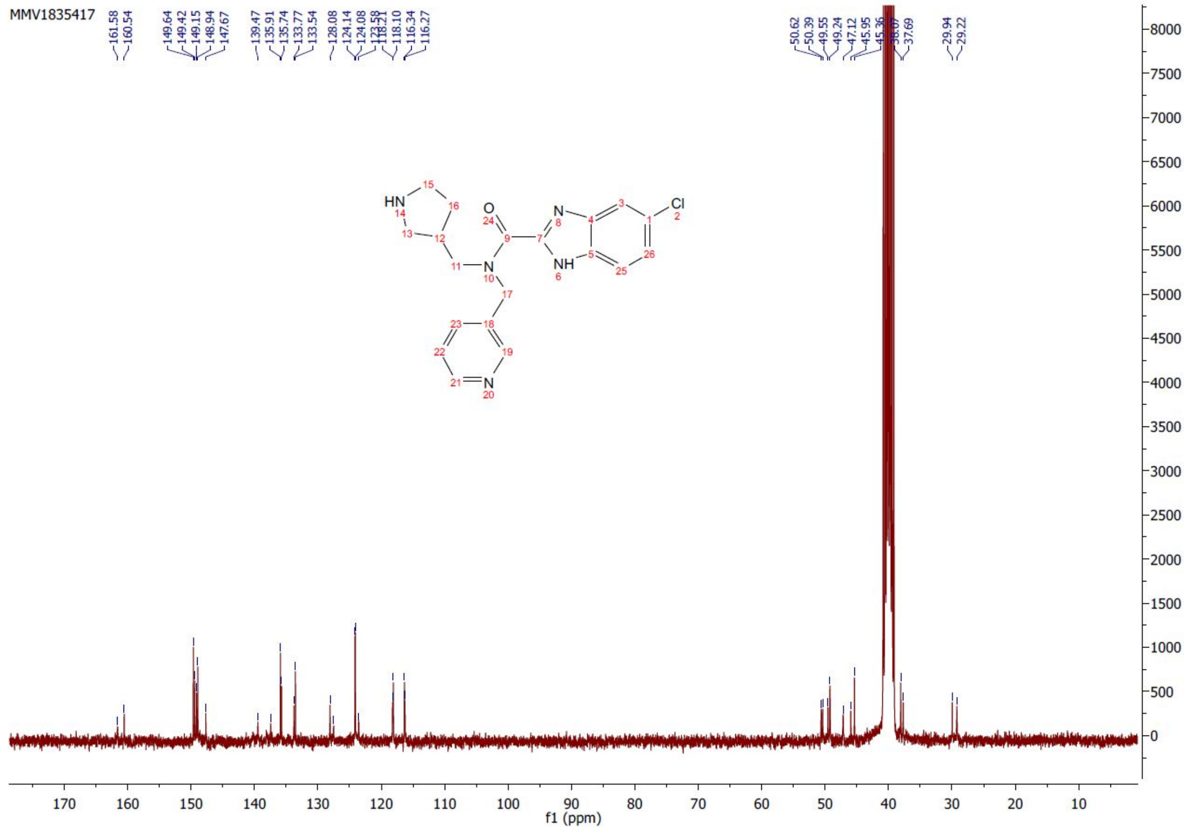

^{13}C NMR (75 MHz, DMSO-d_6)-6i

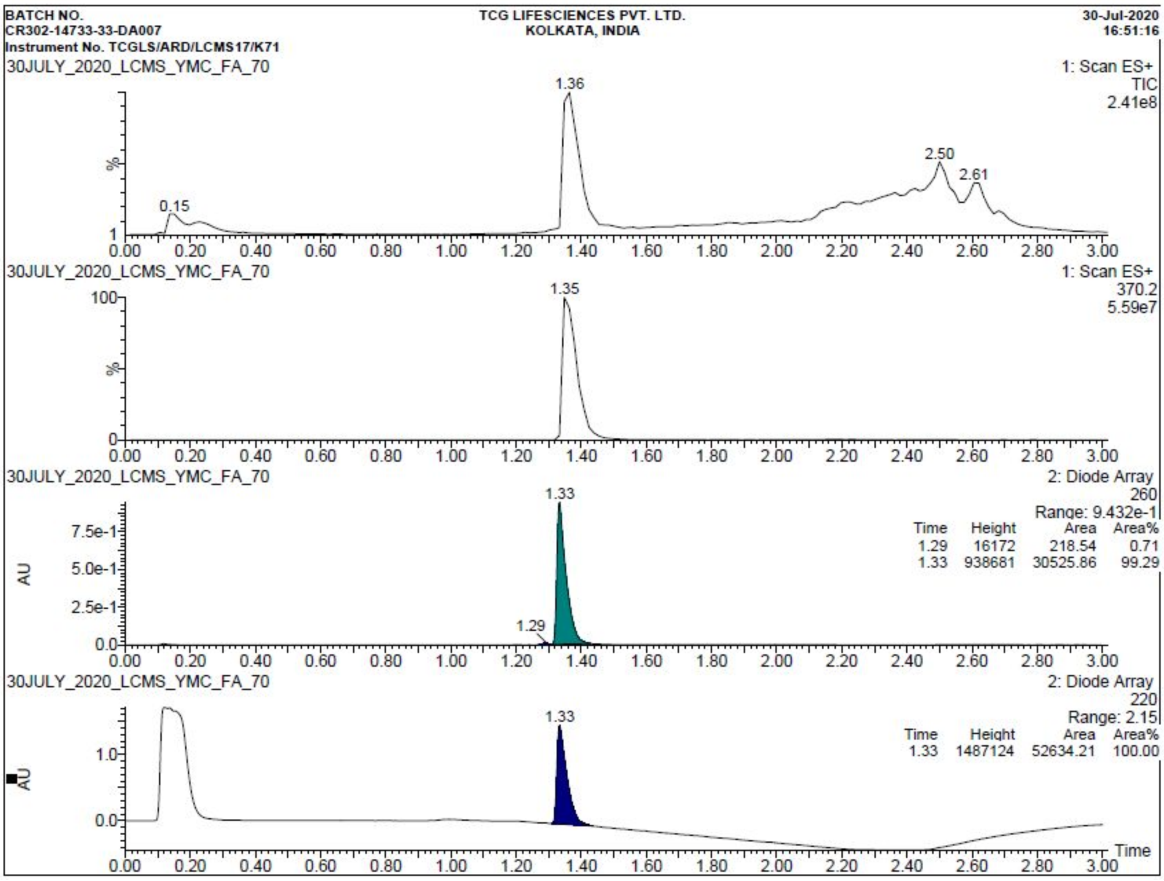
BATCH NO.
CR302-14733-33-DA007
Instrument No. TCGLS/ARD/LCMS17/K71
TCG LIFESCIENCES PVT. LTD.
KOLKATA, INDIA
30-Jul-2020
16:51:16
30JULY_2020_LCMS_YMC_FA_70
1: Scan ES+
TIC
2.41e8
1.36
2.50
2.61
0.15
370.2
5.59e7
1.35
2: Diode Array
260
Range: 9.432e-1
1.33
1.29
Time Height Area Area%
1.29 16172 218.54 0.71
1.33 938681 30525.86 99.29
220
Range: 2.15
Time Height Area Area%
1.33 1487124 52634.21 100.00
AU
Time

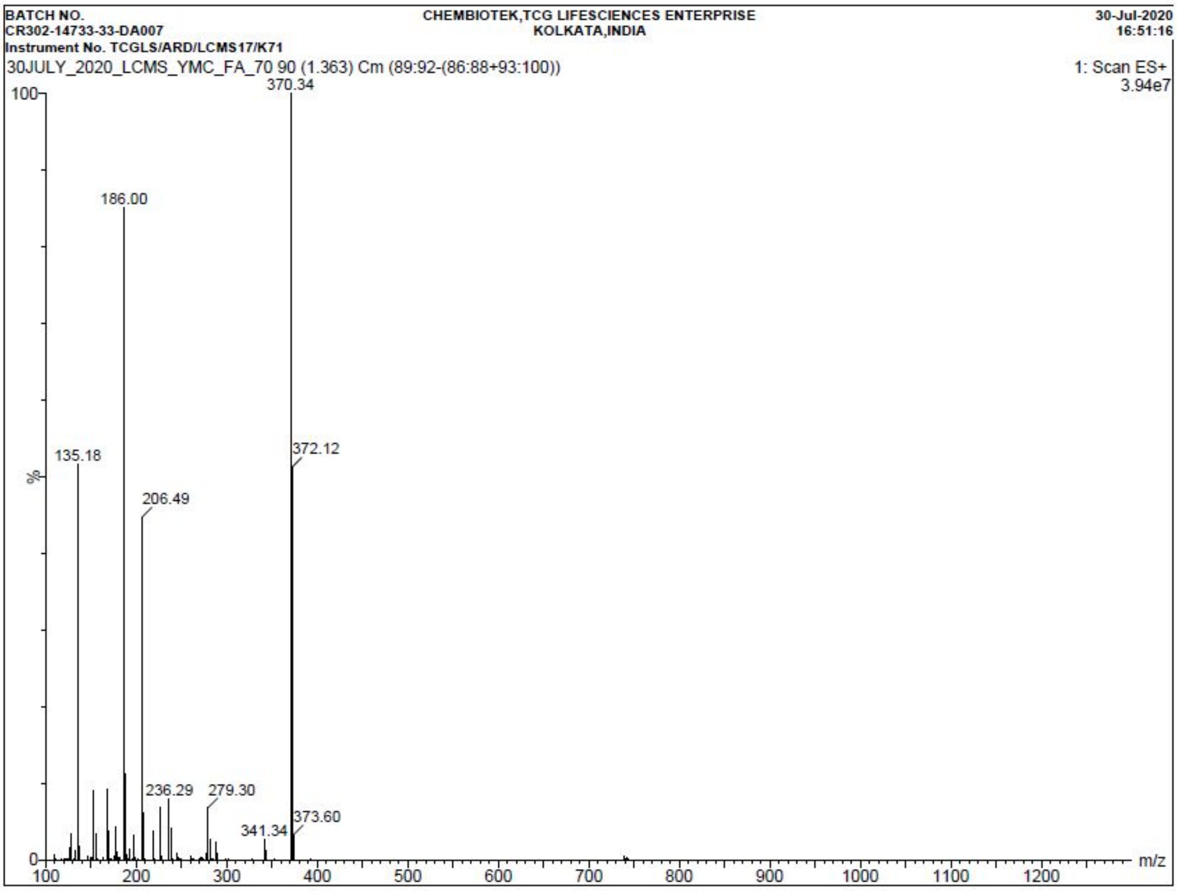
BATCH NO.
CR302-14733-33-DA007
Instrument No. TCGLS/ARD/LCMS17/K71
CHEMBIOTEK,TCG LIFESCIENCES ENTERPRISE
KOLKATA,INDIA
30-Jul-2020
16:51:16
30JULY_2020_LCMS_YMC_FA_70 90 (1.363) Cm (89:92-(86:88+93:100))
1: Scan ES+
3.94e7
370.34
186.00
135.18
372.12
206.49
236.29
279.30
373.60
341.34
m/z

LCMS-6i

	Resultados HRMS		
	Responsável:	Diego C Andrade	
	Data:	06/10/22	
	**Laboratório Institucional de Espectrometria de Massas**		

Prof. Dr. Luiz Carlos Dias
Aluno Anwar Shamim
Amostra MMV417

Espectro completo de 50 a 750 m/z em modo positivo

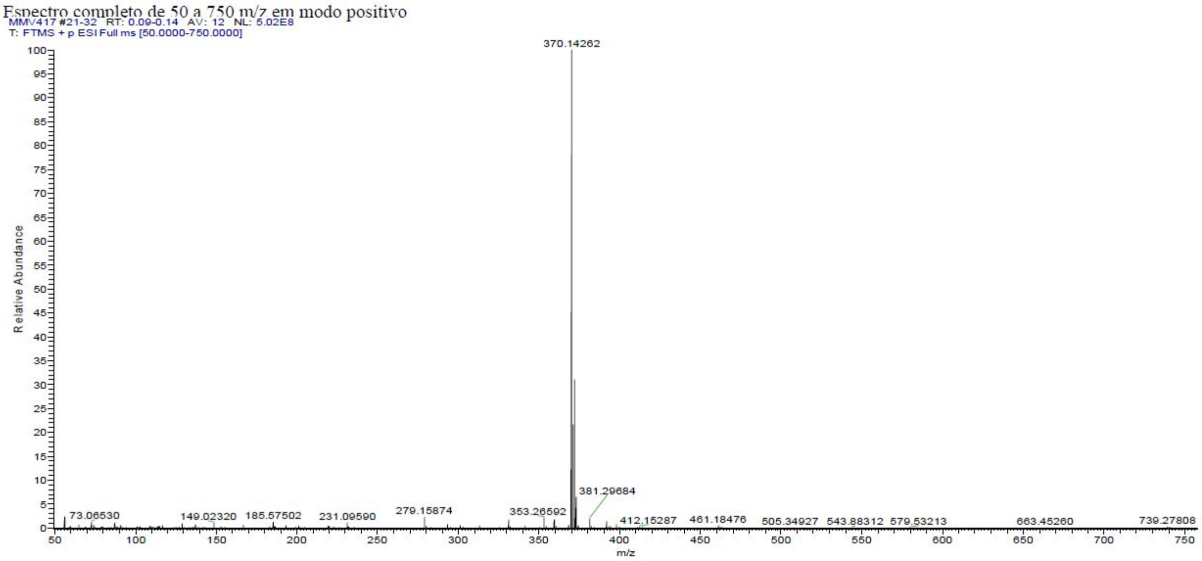

HRMS-6i

	Resultados HRMS		
	Responsável:	Diego C Andrade	
	Data:	06/10/22	
	**Laboratório Institucional de Espectrometria de Massas**		

Comparação entre espectro real (superior) e simulado (inferior) para C19H20ClN5OH+

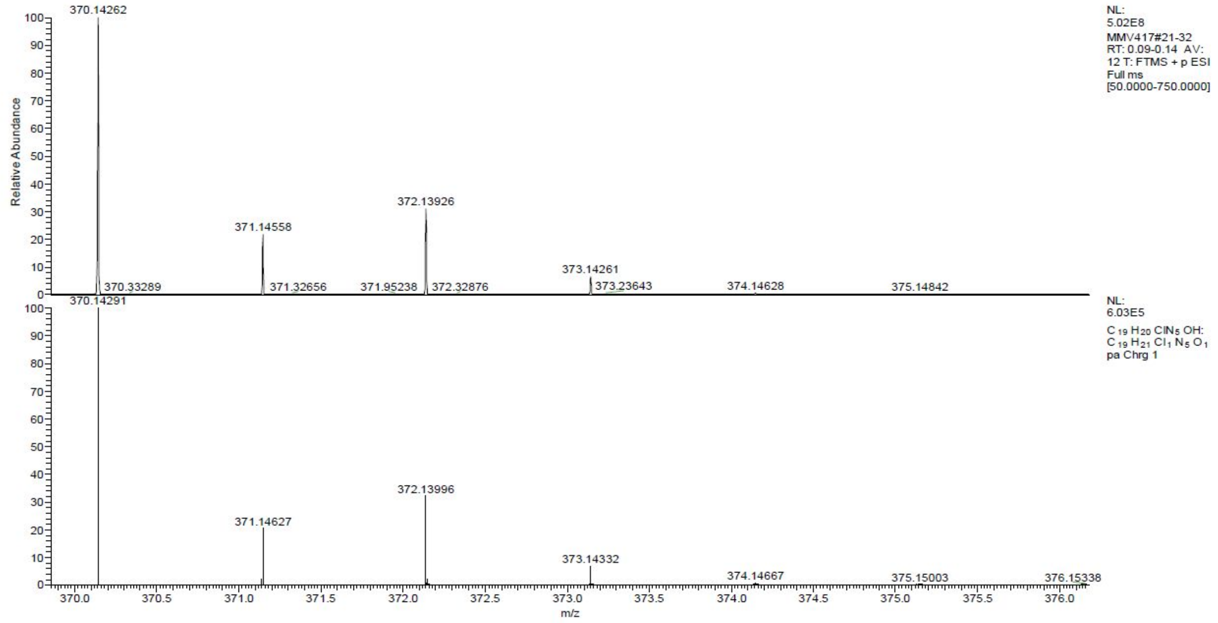

HRMS-6i

3-chloro-N-(pyridin-3-ylmethyl)-N-(pyrrolidin-3-ylmethyl)benzamide (6j)

The Following compound was purchased (Recorded only LC-MS and HRMS for registration)

Code	Targets	Purity (LC-MS)	LC-MS m/z:
**6j**	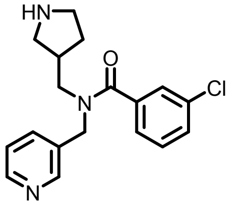	95.43%	330.3 [M + H]+. HRMS: $[M+H]^+$ Calcd for $C_{18}H_{21}ClN_3O$ = 330.1373; Found = 330.1364

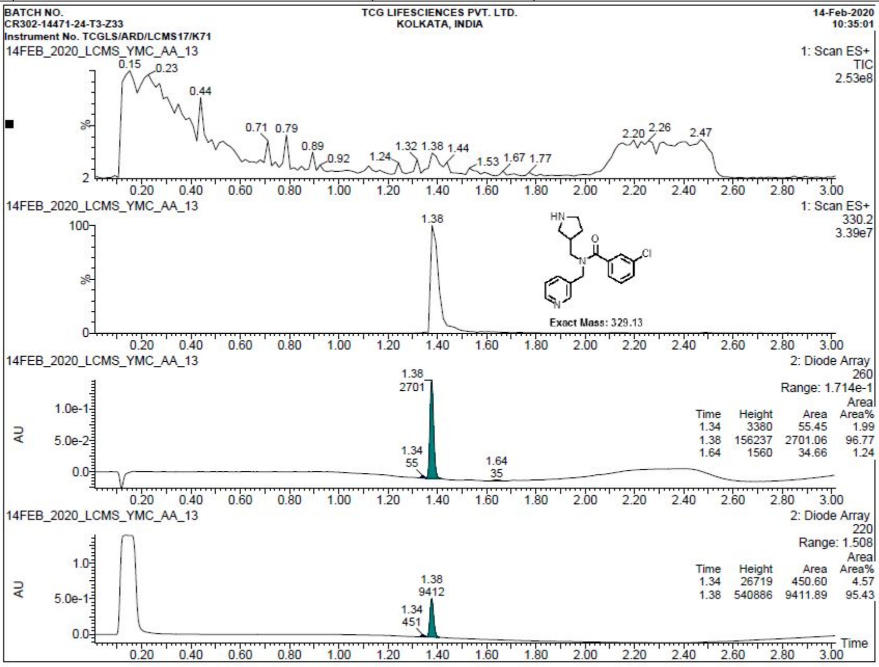

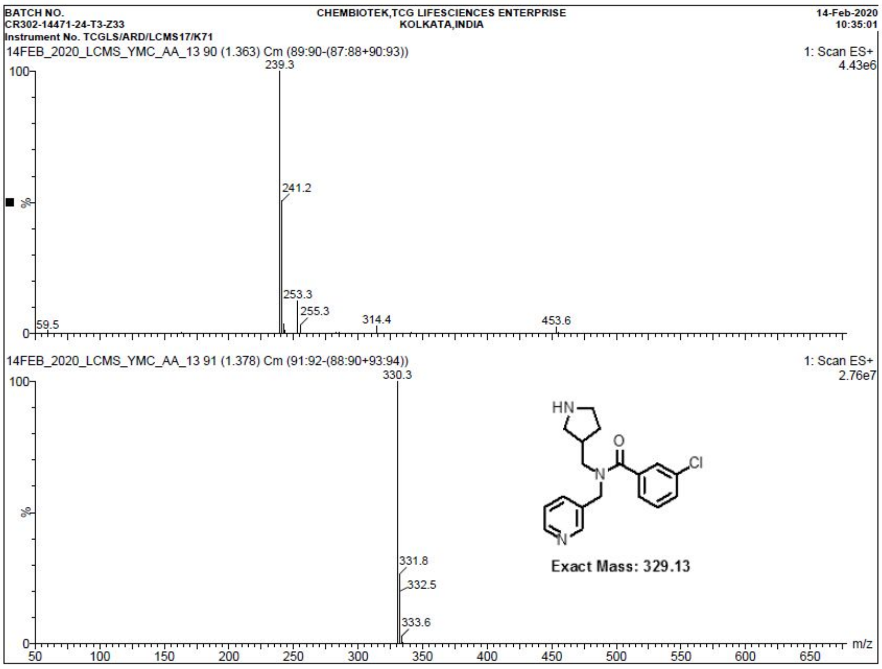

LCMS-6j

N-benzyl-5-chloro-N-(pyrrolidin-3-ylmethyl)-1H-indole-2-carboxamide (6k):

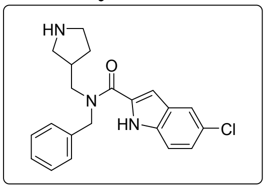

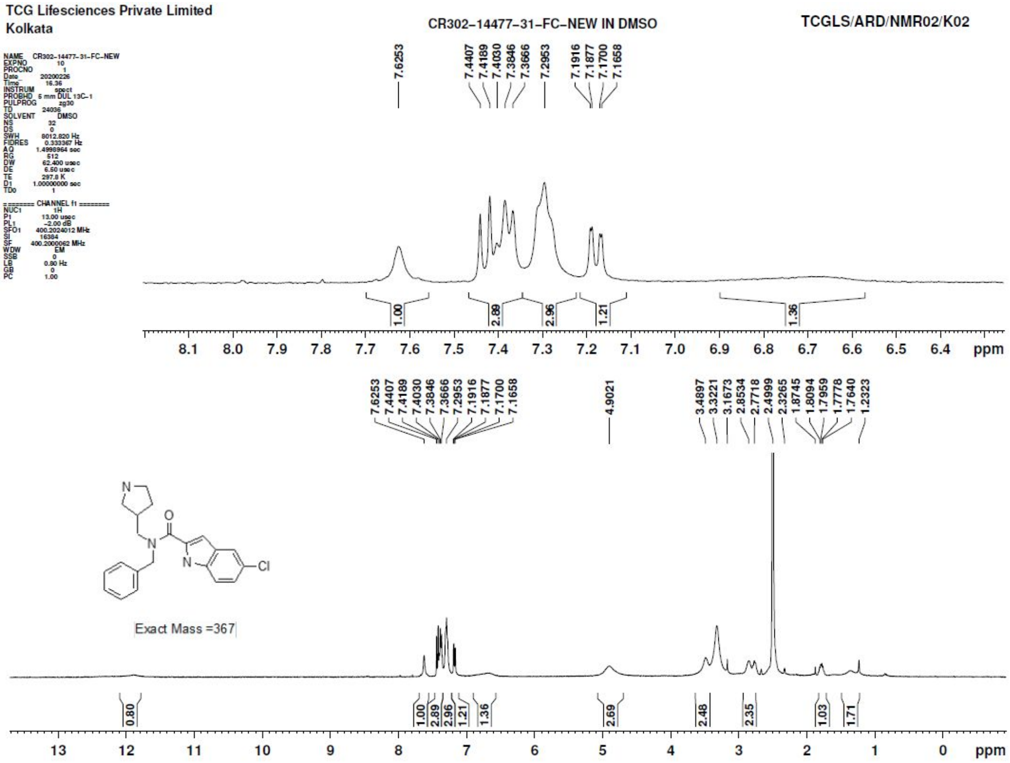

^{1}H NMR (400 MHz, DMSO-d_6)-6k

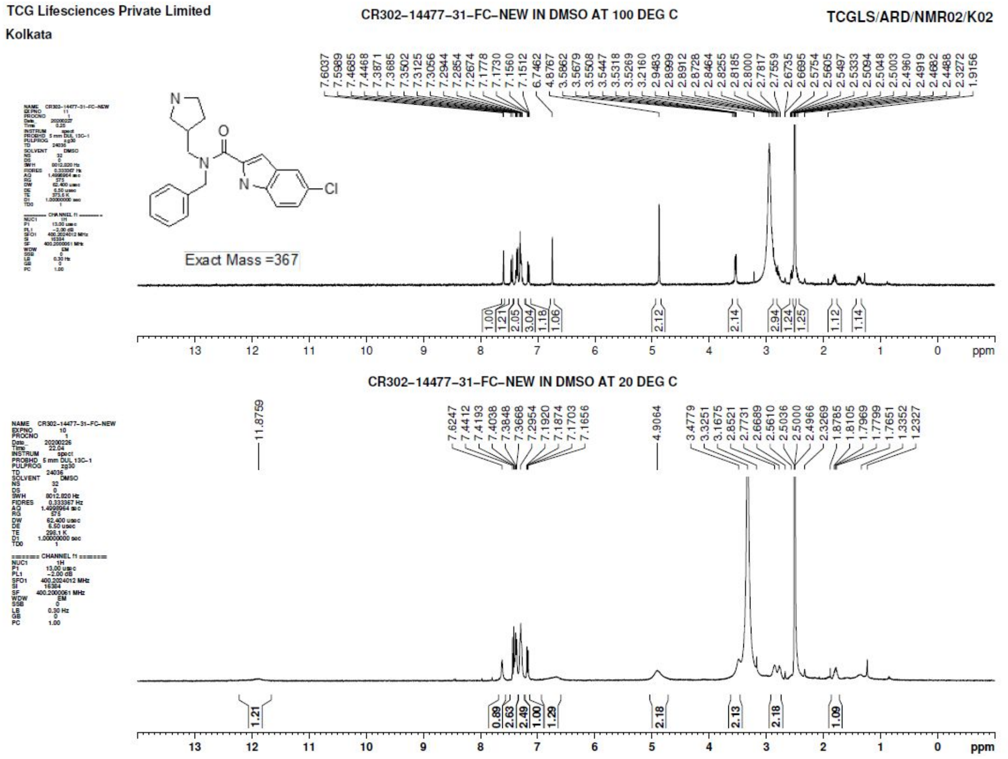

^{1}H NMR (20 °C and 100 °C) (400 MHz, DMSO-d_6)-6k

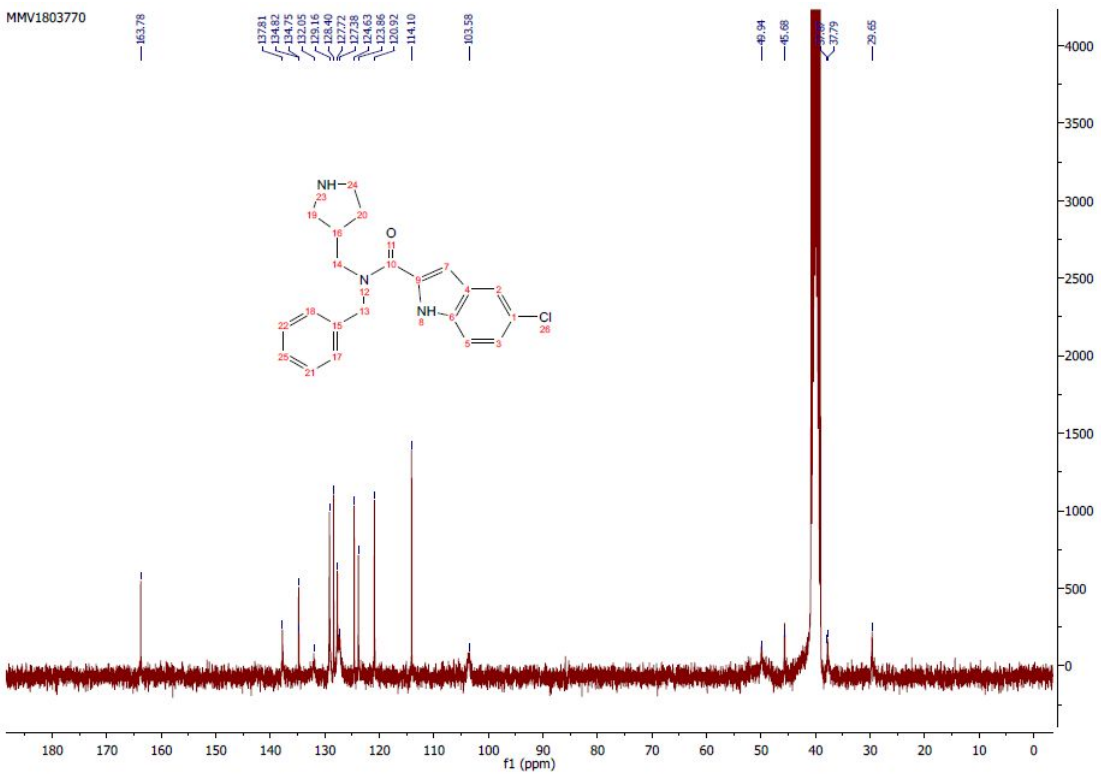

^{13}C NMR (75 MHz, DMSO-d$_6$)-6k

TCG Lifesciences Pvt Ltd , Kolkata , India

Data file:	D:\LCMS 2019\DATA\FEBRUARY-2020\27022020 2020-02-27 09-36-27\CR302-14477-31-FC-NEW.D
Sample name:	**CR302-14477-31-FC-NEW**
Instrument:	TCGLS_ARD_LCMS20_K79
Location:	D1F-B1
Injection date:	2/27/2020 11:42:55 AM
Injection volume:	0.700
Acq. method:	Moni +ve-ve_FA_5MIN.M
Description:	Column:YMC TRIART C18(33x2.1mm,3µ)-FAF

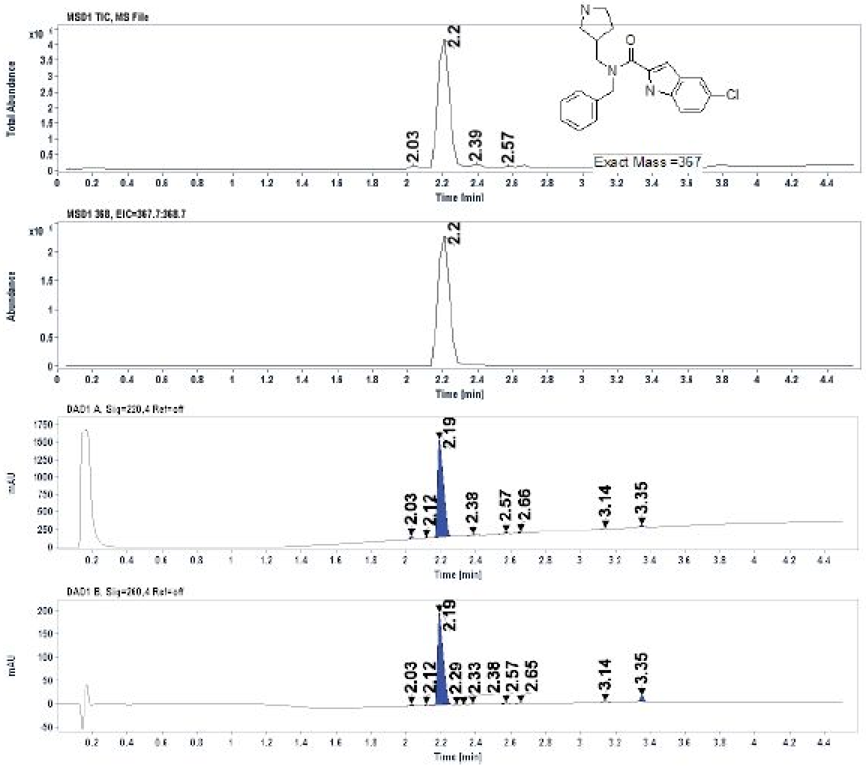

LCMS-6k

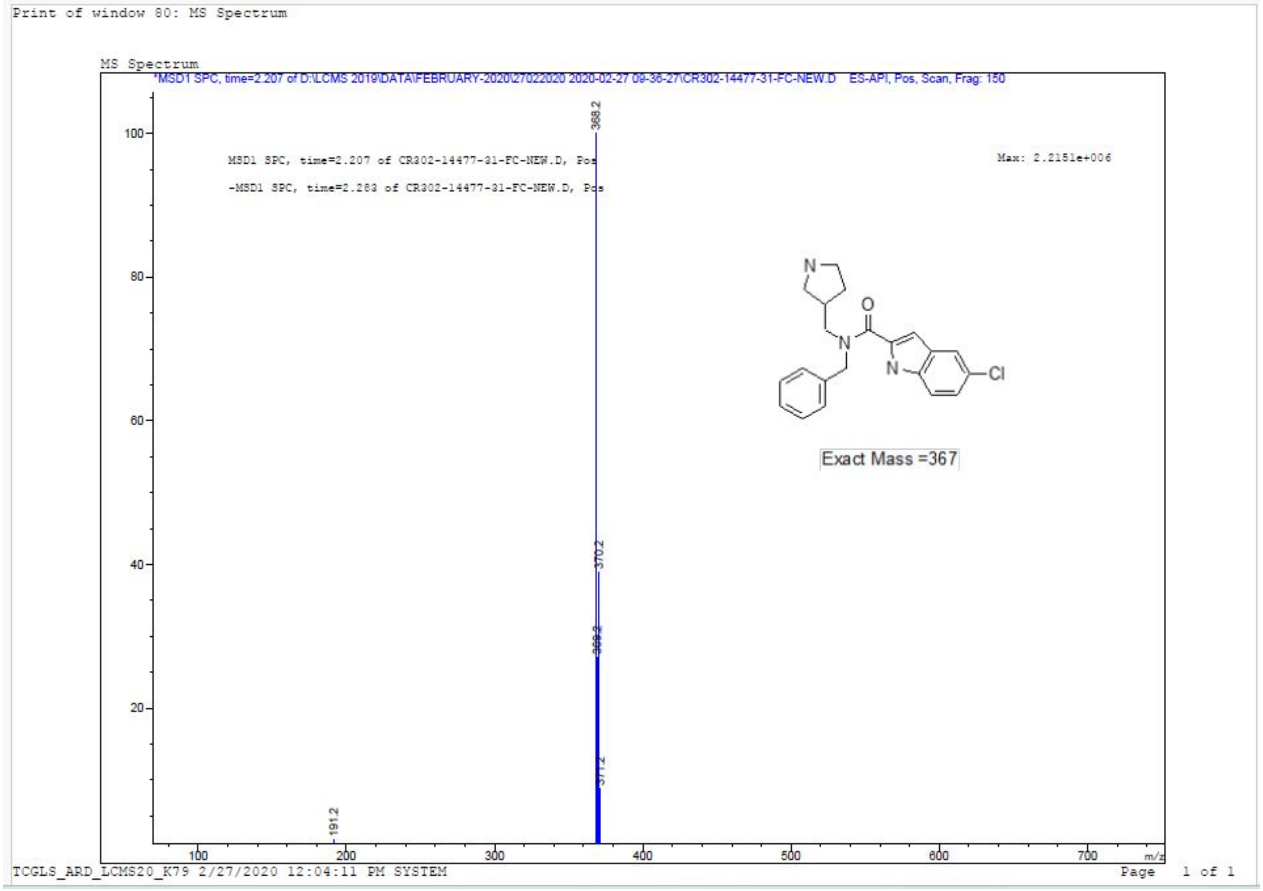

LCMS-6k

Resultados HRMS	
Responsável:	Diego C Andrade
Data:	06/10/22
**Laboratório Institucional de Espectrometria de Massas**	

Prof. Dr. Luiz Carlos Dias
Aluno Anwar Shamim
Amostra MMV770

Espectro completo de 50 a 750 m/z em modo positivo

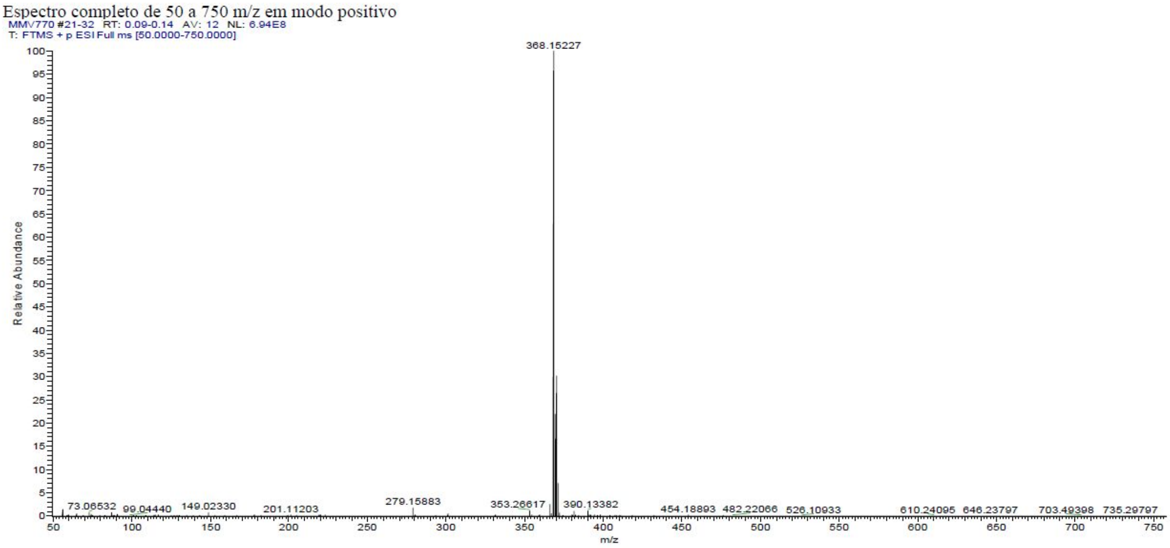

HRMS-6k

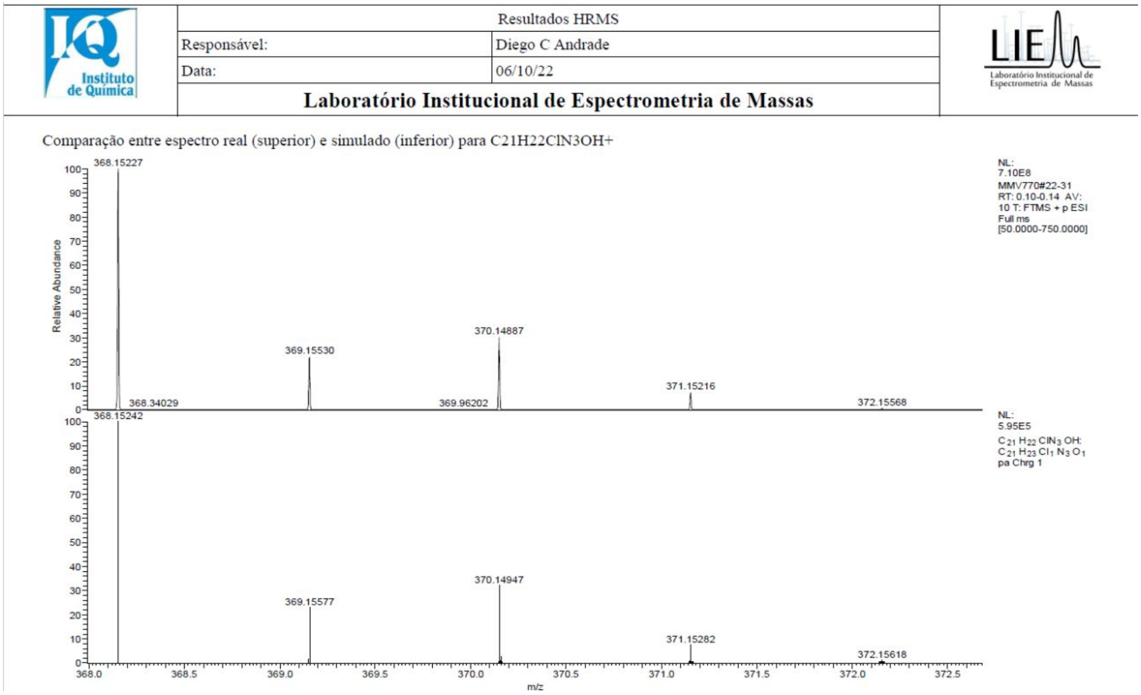

HRMS-6k

5-chloro-N-(4-pyridylmethyl)-N-(pyrrolidin-3-ylmethyl)-1H-indole-2-carboxamide (6l):

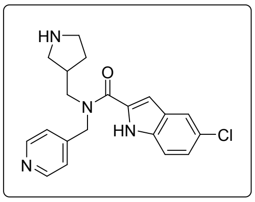

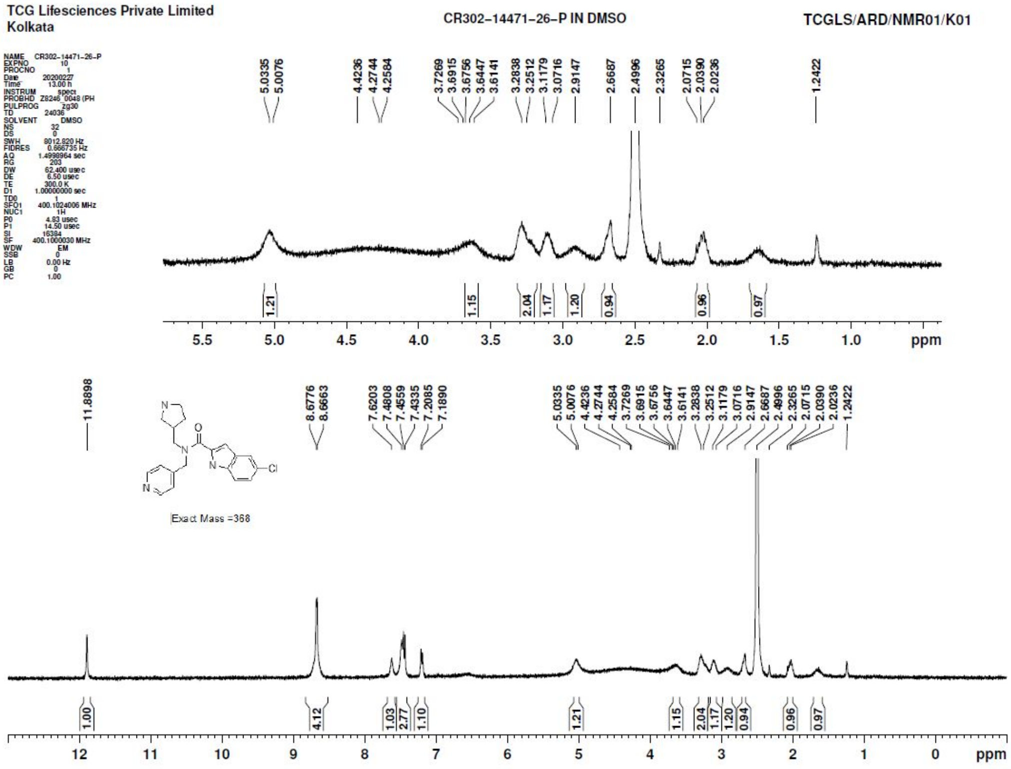

^{1}H NMR (100 °C) (400 MHz, DMSO-d$_6$)-6l

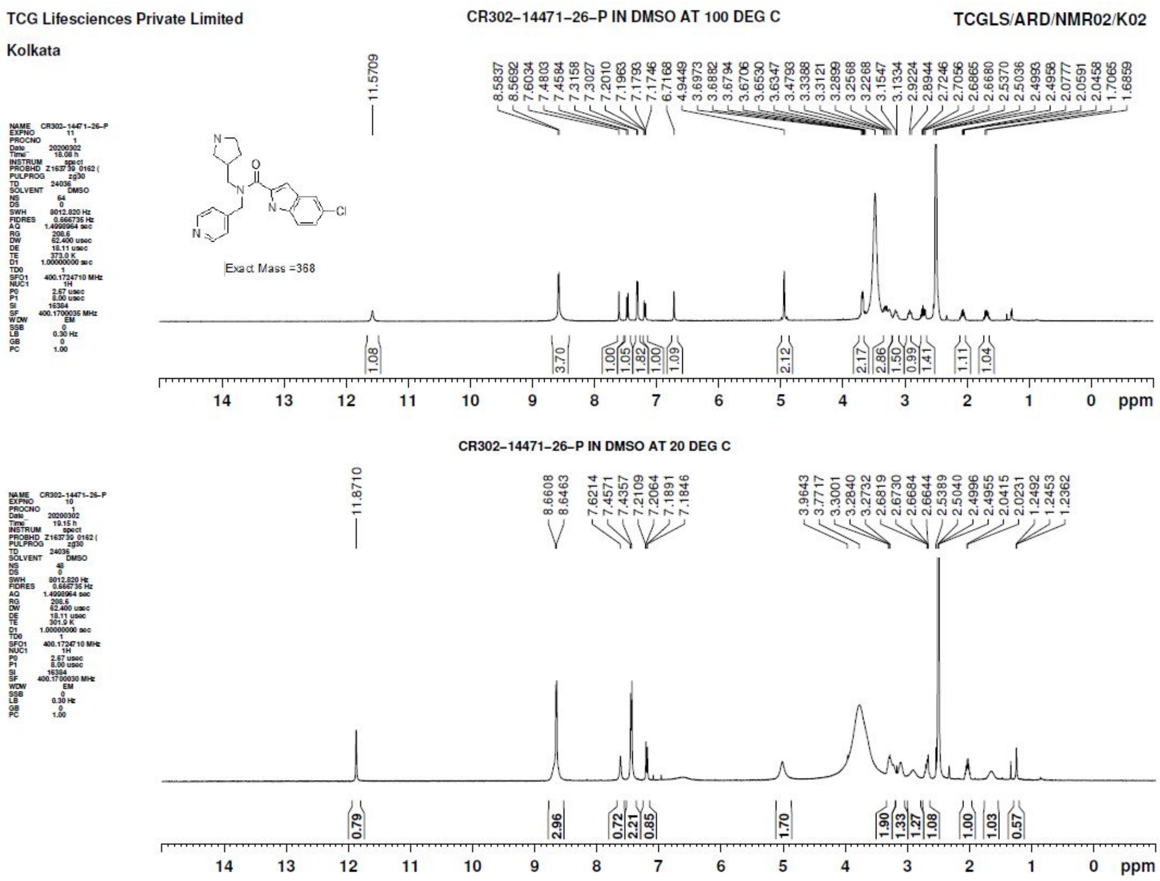

^{1}H NMR (20 °C and 100 °C) (400 MHz, DMSO-d_6)-6l

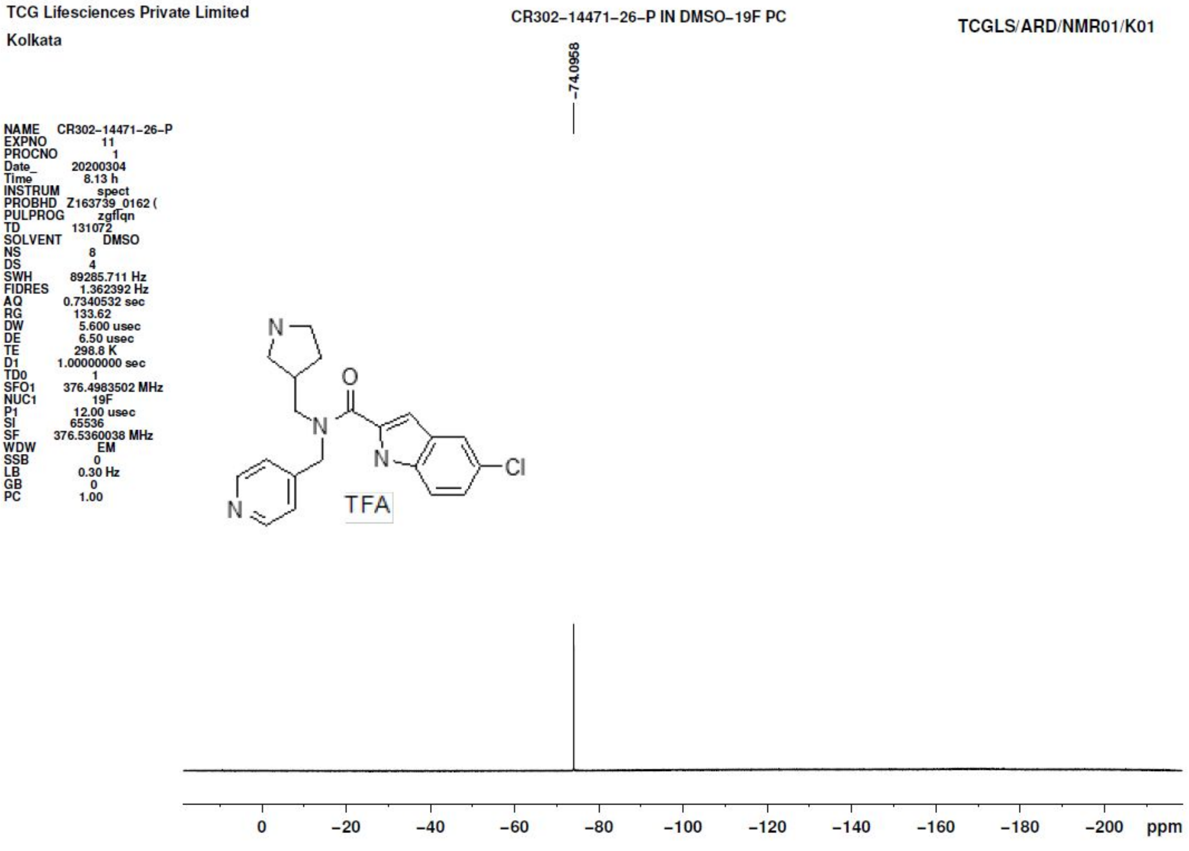

^{19} FNMR (400 MHz, DMSO-d_6)-6l

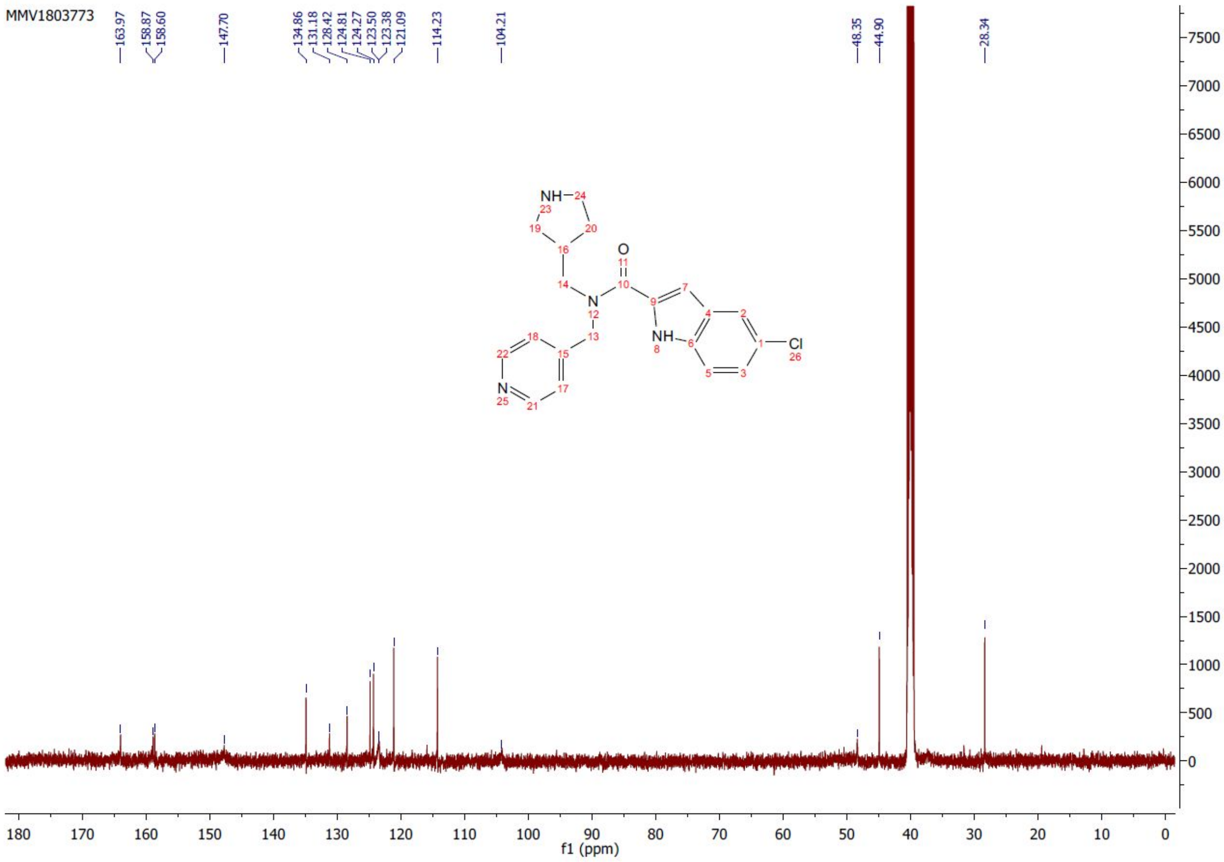

^{13}C NMR (125 MHz, DMSO-d_6)-6l

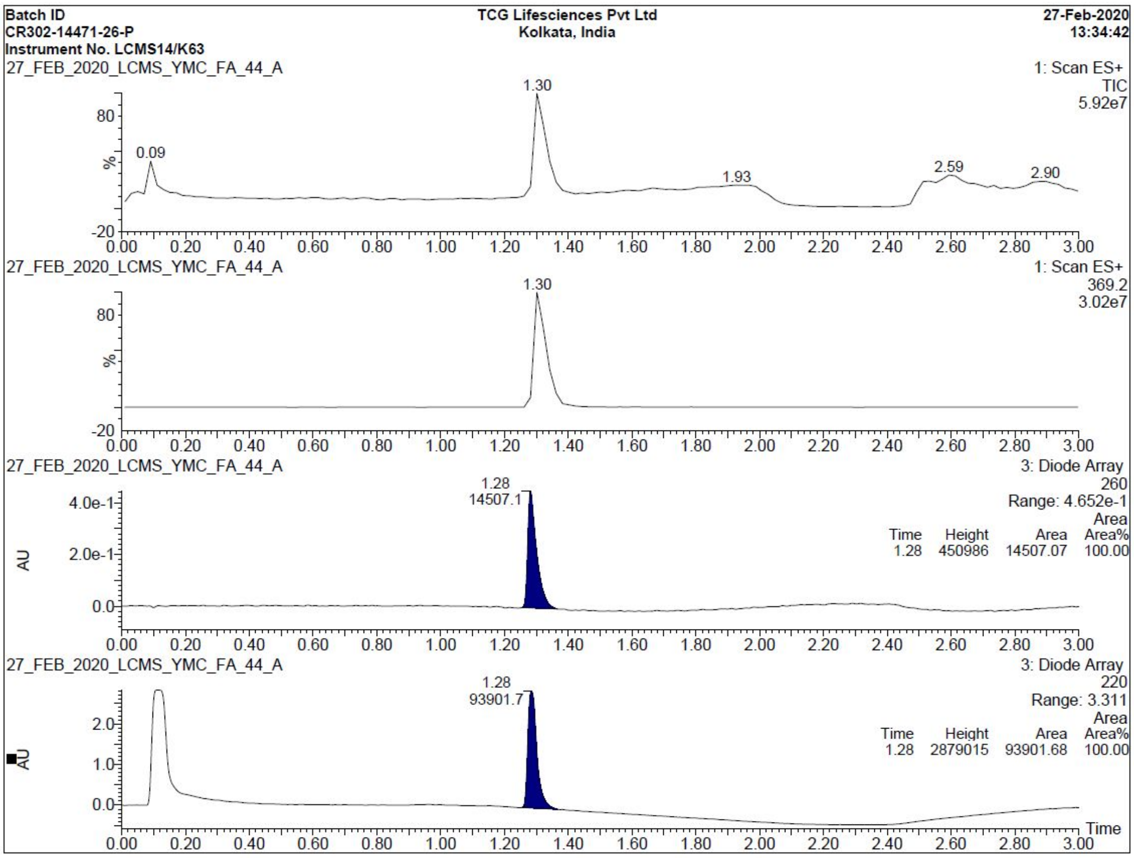

LCMS-6l

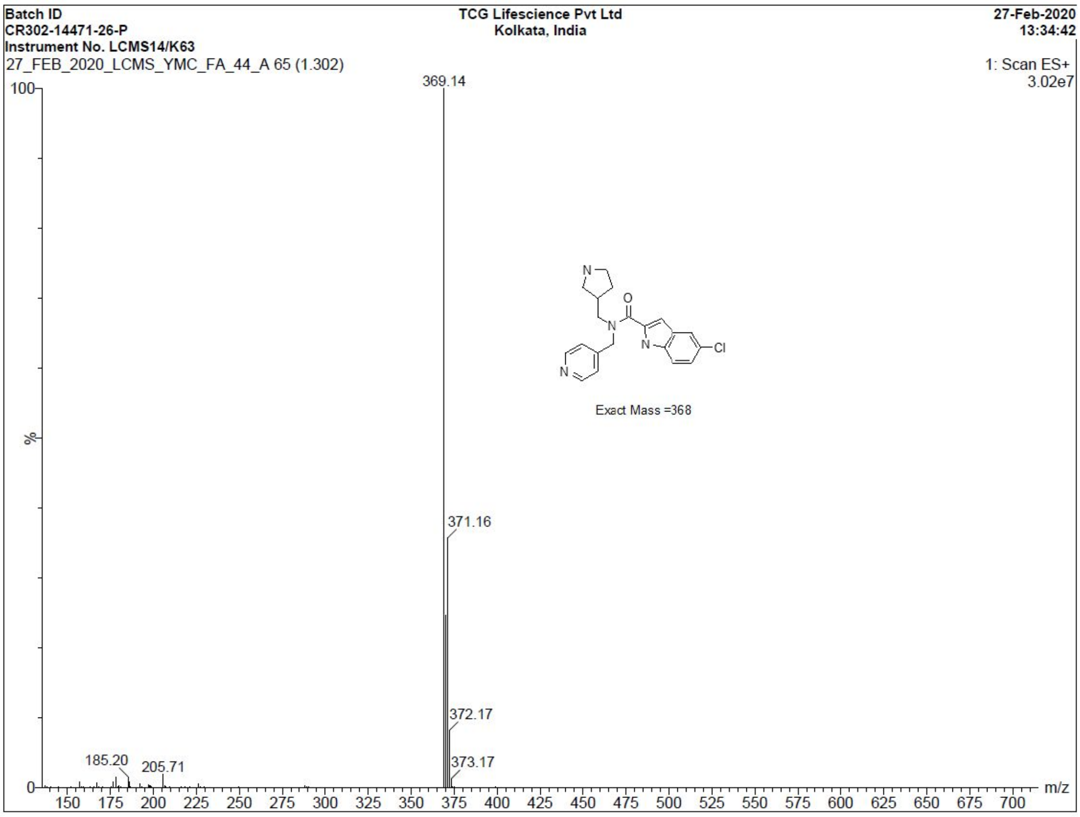

LCMS-6l

	Resultados HRMS
Responsável:	Diego C Andrade
Data:	19/12/22

Laboratório Institucional de Espectrometria de Massas

Prof. Dr. Luiz Carlos Dias
Aluna Mariana Ferrer Casal
Amostra MMV773

Espectro completo de 80 a 1200 m/z em modo positivo

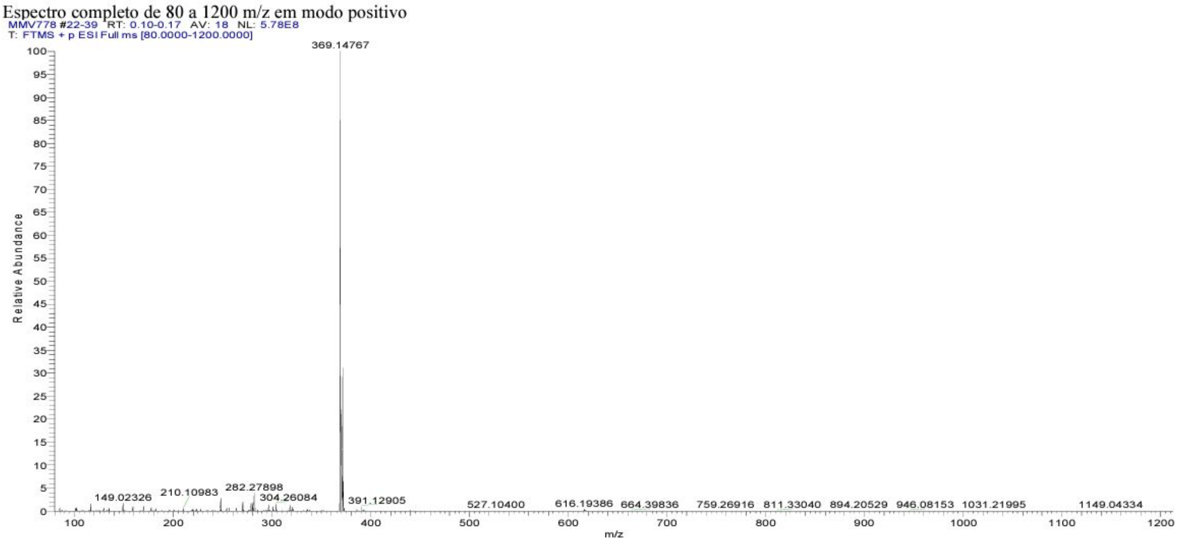

HRMS-6l

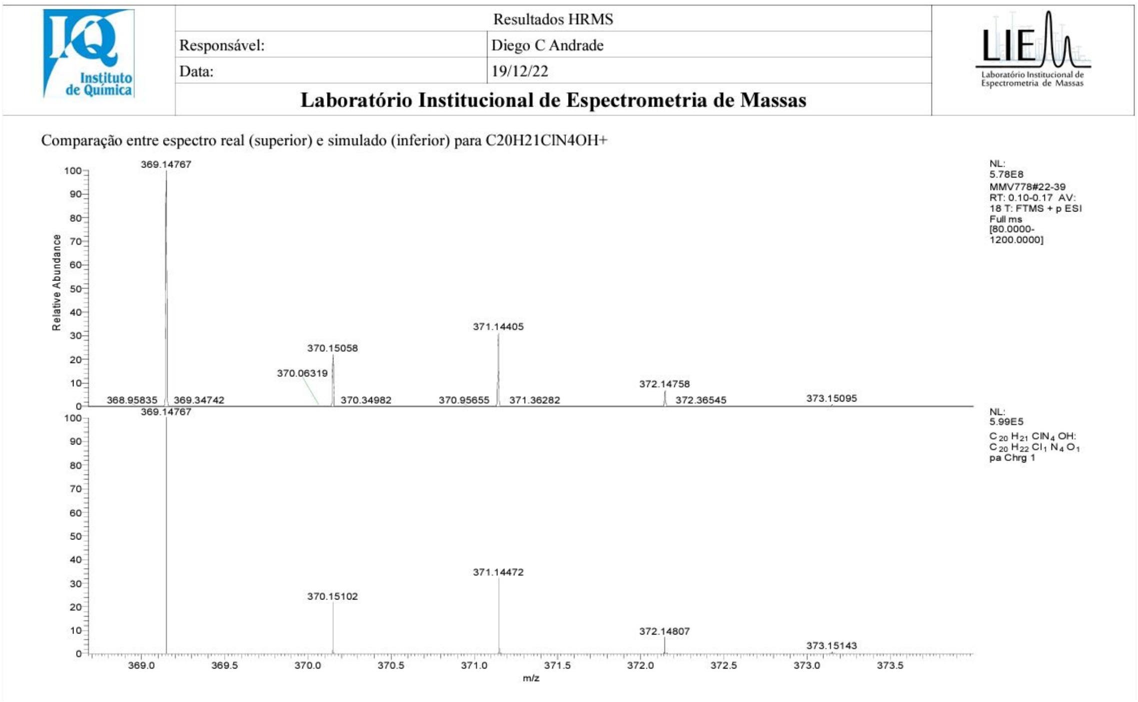

HRMS-6l

5-chloro-N-(2-pyridylmethyl)-N-(pyrrolidin-3-ylmethyl)-1H-indole-2-carboxamide (6m):

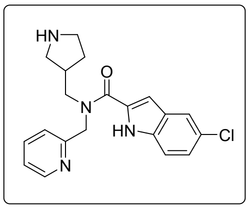

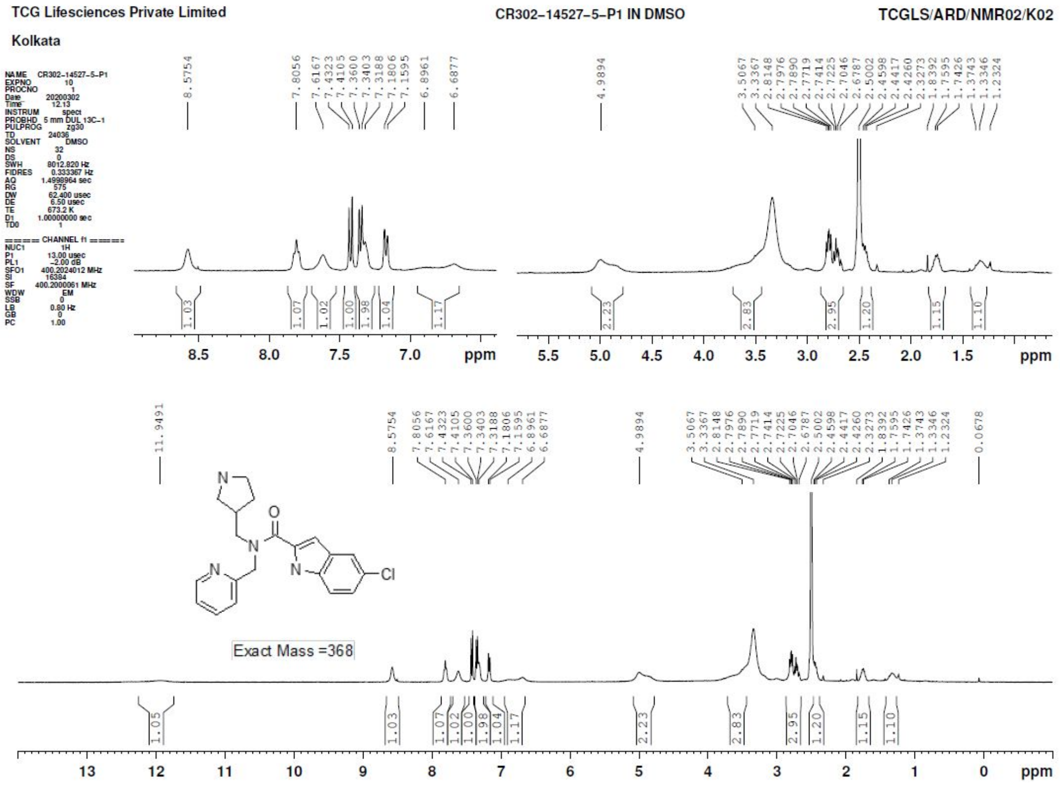

^{1}H NMR (400 MHz, DMSO-d$_6$)-6m

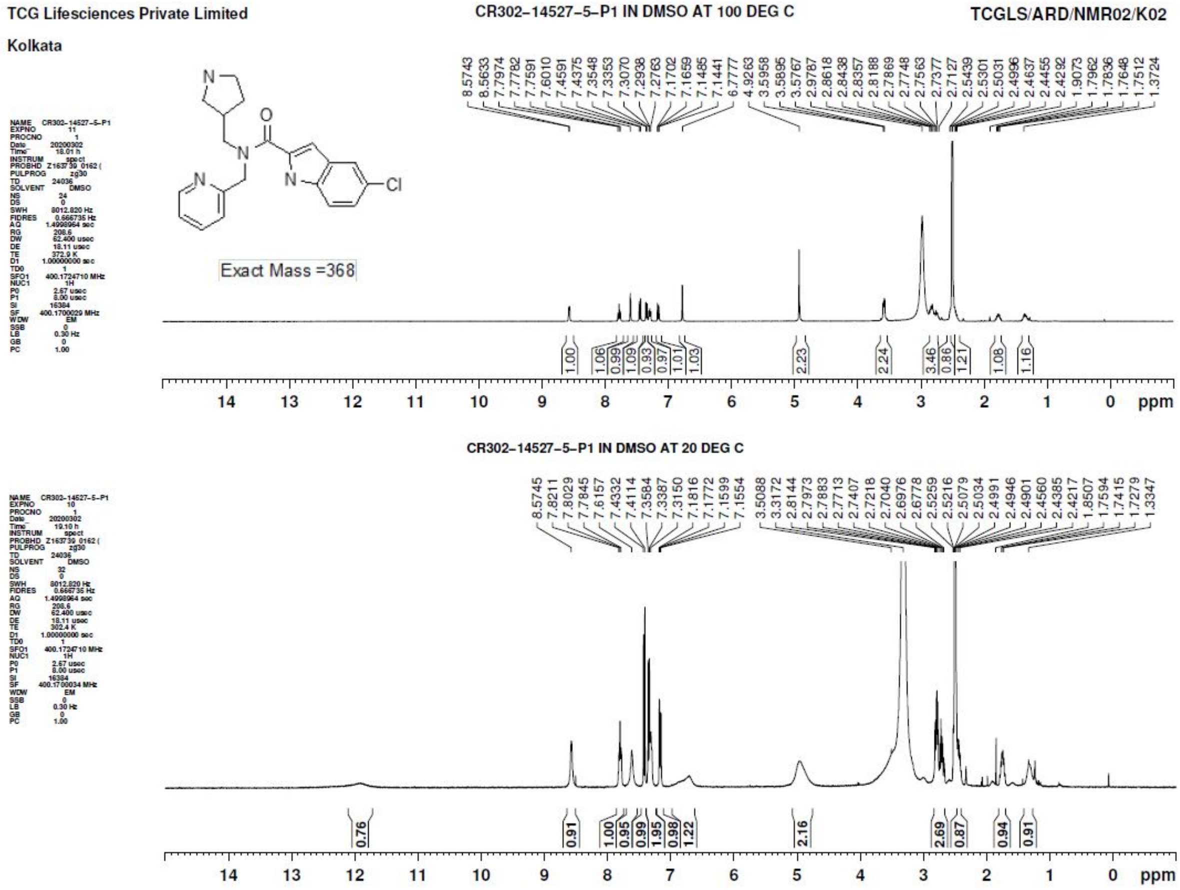

^{1}H NMR (20 °C and 100 °C) (400 MHz, DMSO-d_6)-6m

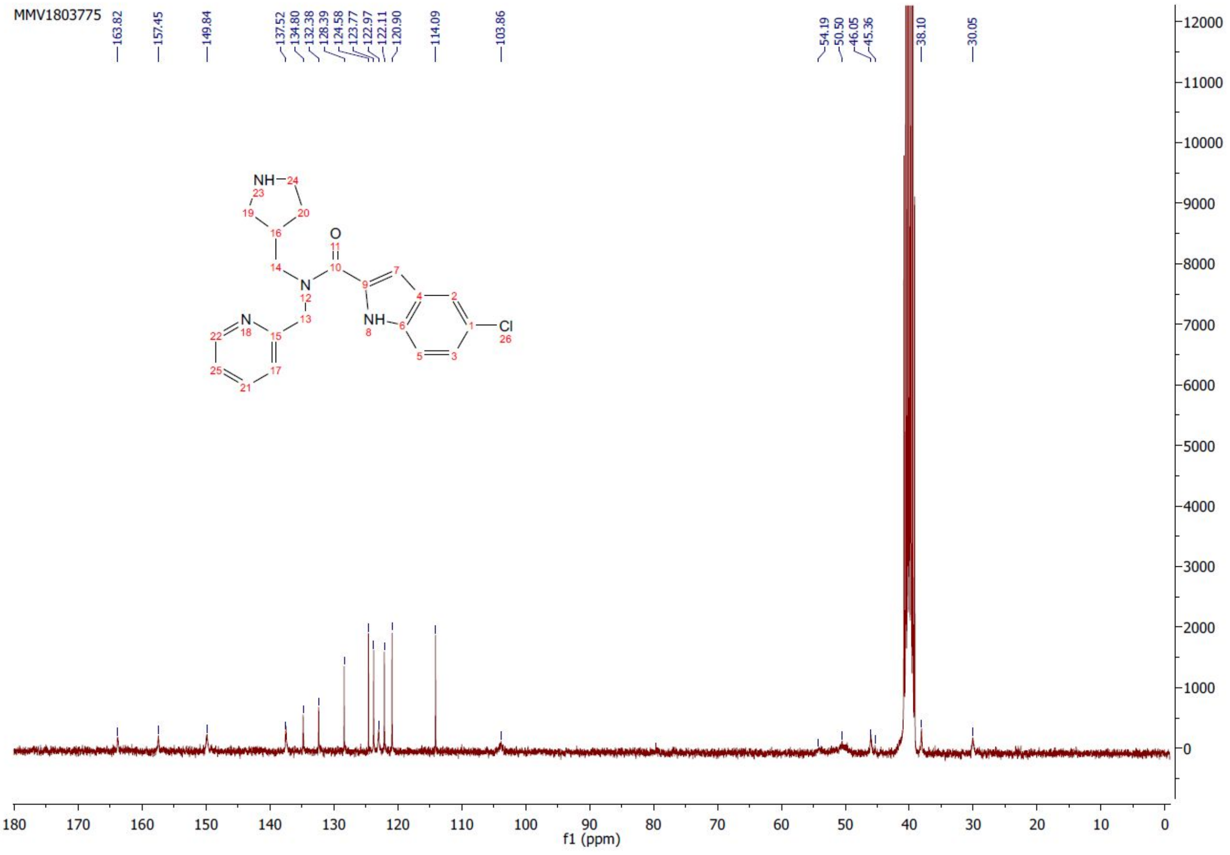

^{13}C NMR (75 MHz, DMSO-d_6)-6m

TCG Lifesciences Pvt Ltd , Kolkata , India

Data file:	D:\LCMS 2019\DATA\MARCH-2020\02032020 2020-03-02 09-56-43\CR302-14527-5-P1.D		
Sample name:	**CR302-14527-5-P1**		
Instrument:	TCGLS_ARD_LCMS20_K79	Location:	D1F-C5
Injection date:	3/2/2020 1:32:11 PM	Injection volume:	0.600
Acq. method:	Moni +ve-ve_FA_5MIN.M		
Description:	Column:YMC TRIART C18(33x2.1mm,3µ)-FAF		

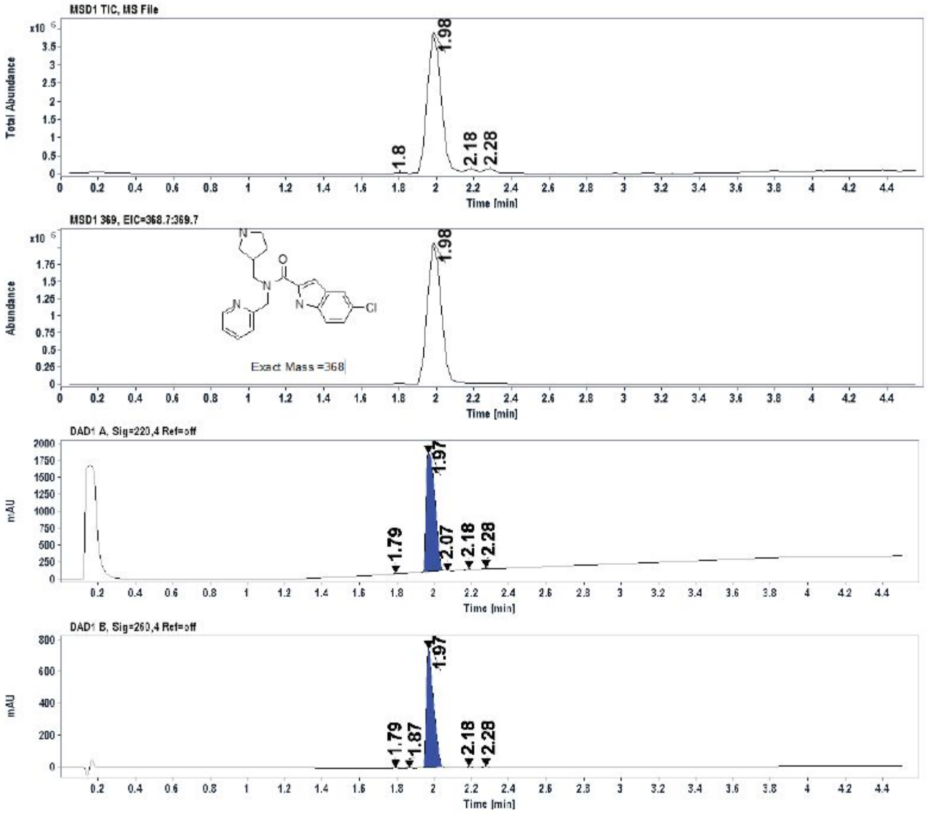

Print of window 80: MS Spectrum

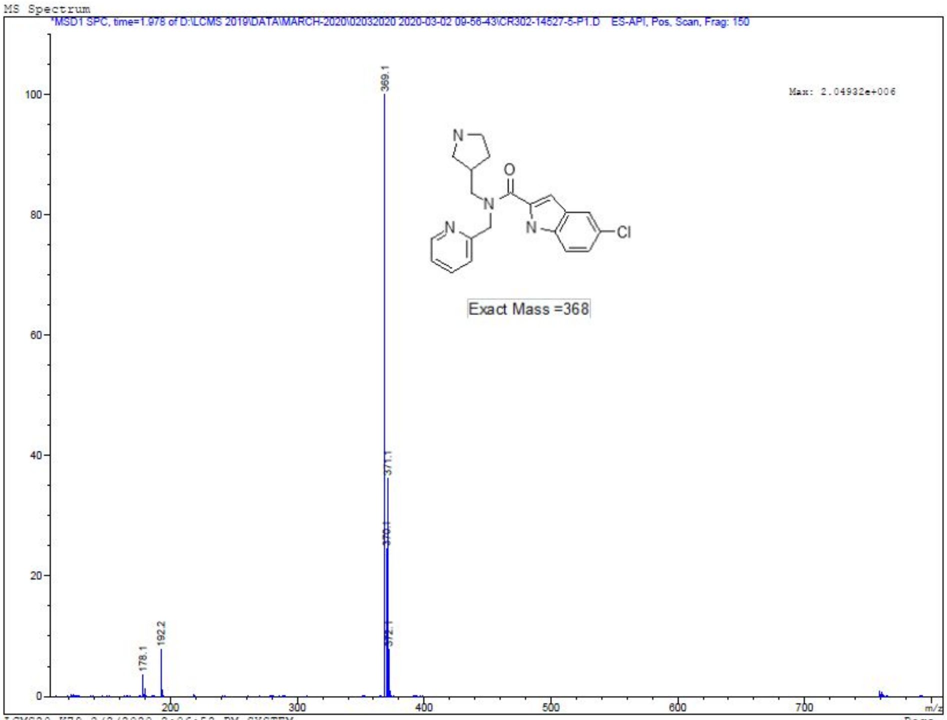

TCGLS_ARD_LCMS20_K79 3/2/2020 2:06:52 PM SYSTEM Page 1 of 1

LCMS-6m

	Resultados HRMS	
Responsável:	Diego C Andrade	
Data:	06/10/22	
Laboratório Institucional de Espectrometria de Massas		

Prof. Dr. Luiz Carlos Dias
Aluno Anwar Shamim
Amostra MMV775

Espectro completo de 50 a 750 m/z em modo positivo

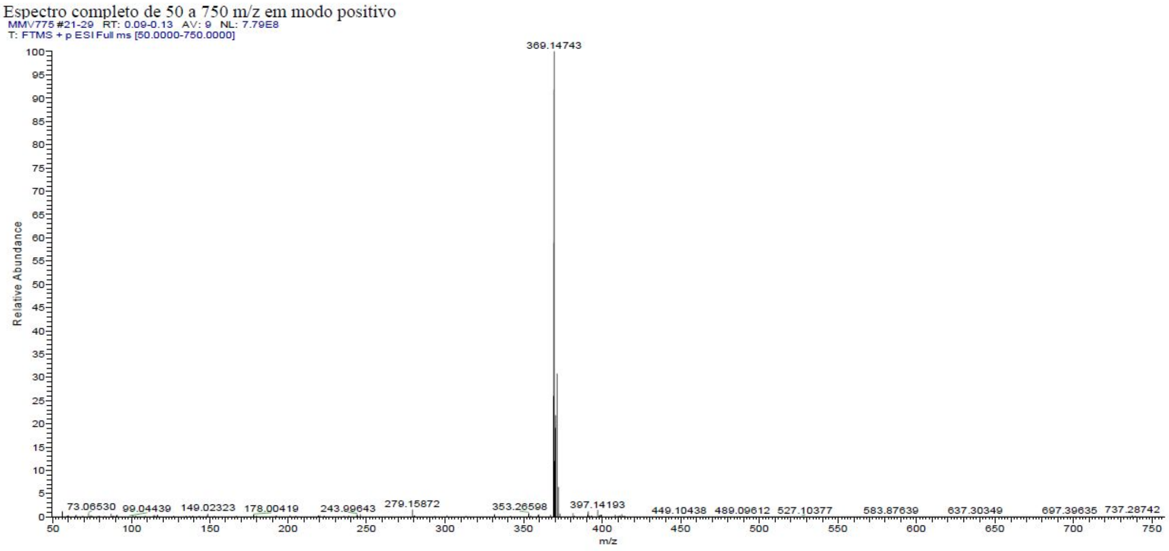

HRMS-6m

	Resultados HRMS	
Responsável:	Diego C Andrade	
Data:	06/10/22	
Laboratório Institucional de Espectrometria de Massas		

Comparação entre espectro real (superior) e simulado (inferior) para C20H21ClN4OH+

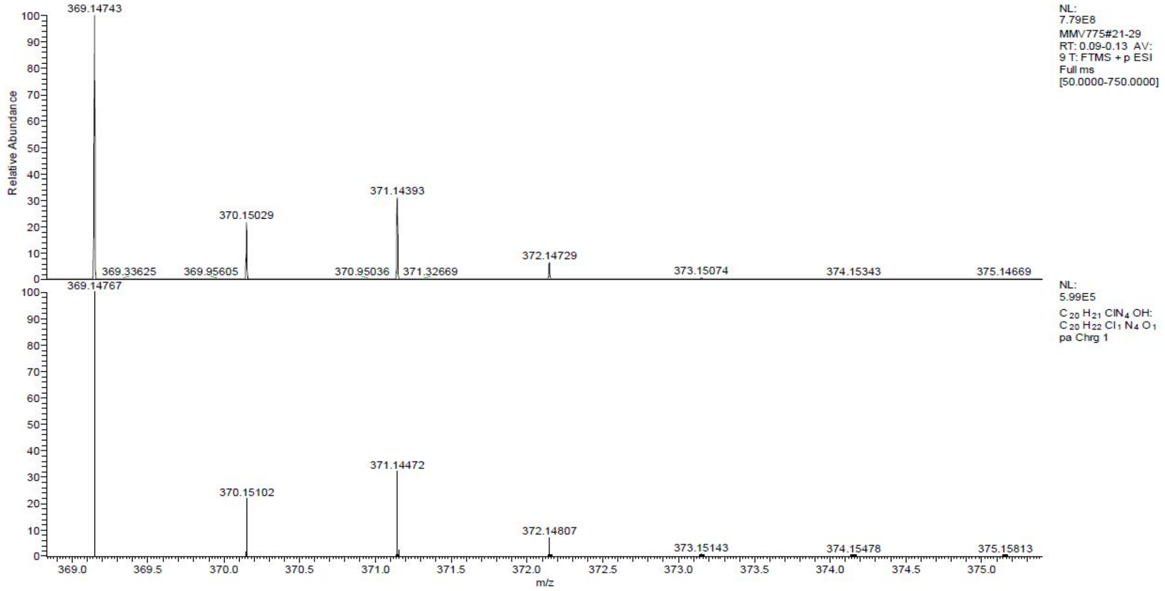

HRMS-6m

5-chloro-N-(pyrazin-2-ylmethyl)-N-(pyrrolidin-3-ylmethyl)-1H-indole-2-carboxamide(6n):

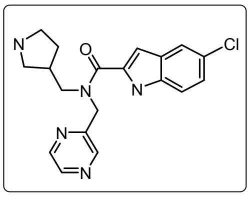

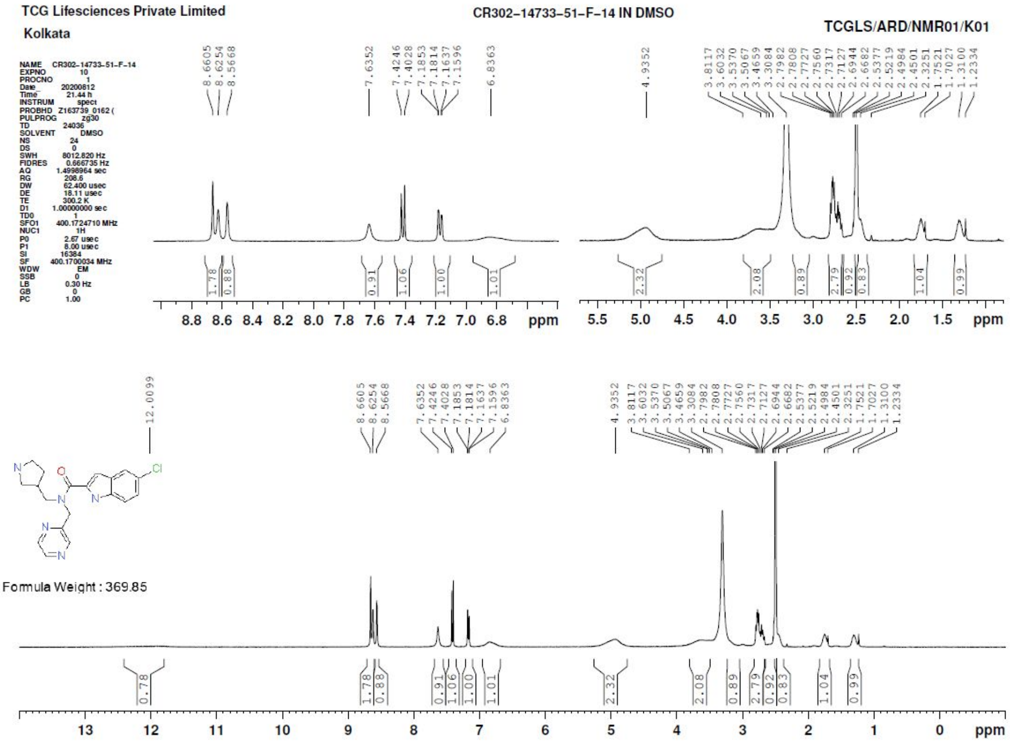

^{1}H NMR (400 MHz, DMSO-d$_6$)-6n

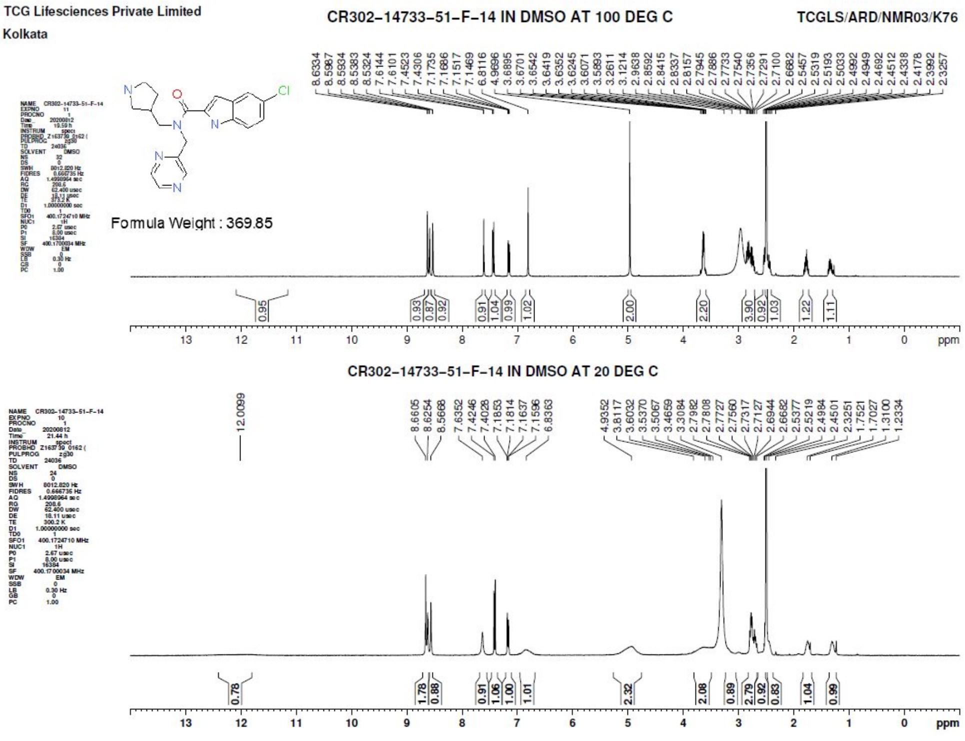

^{1}H NMR (20 °C and 100 °C) (400 MHz, DMSO-d$_6$)-6n

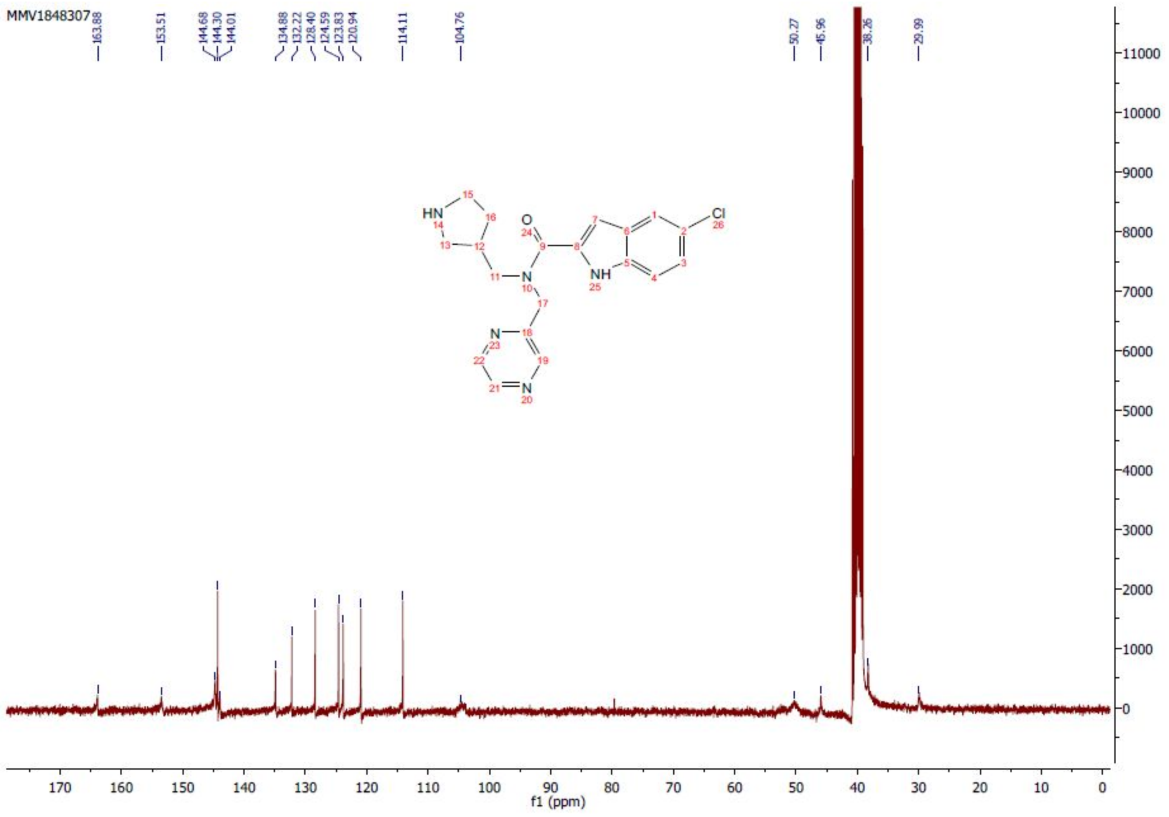

^{13}C NMR (75 MHz, DMSO-d$_6$)-6n

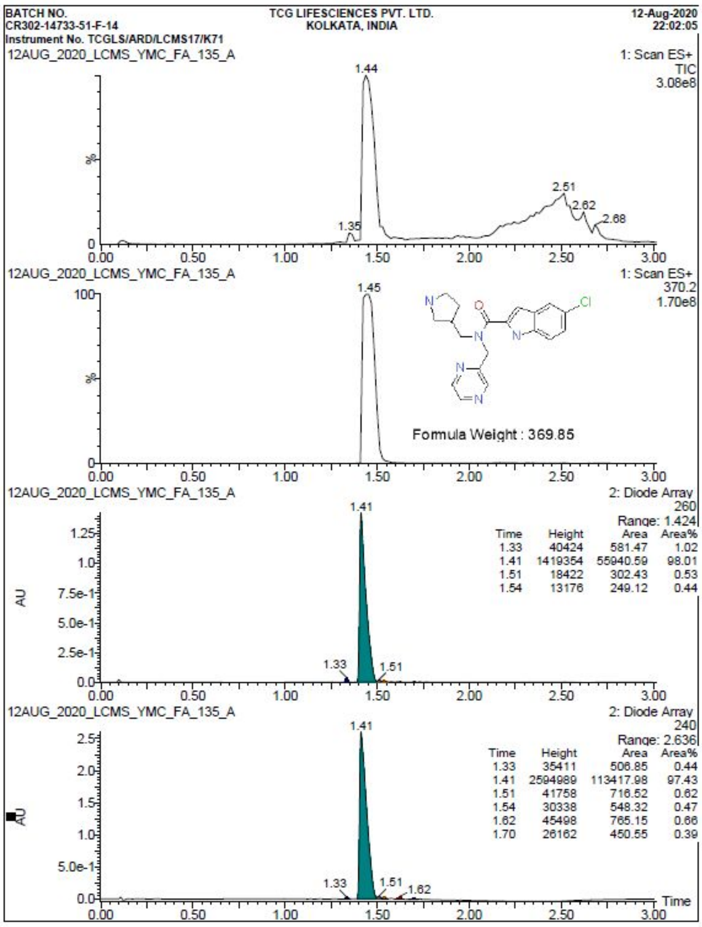

LCMS-6n

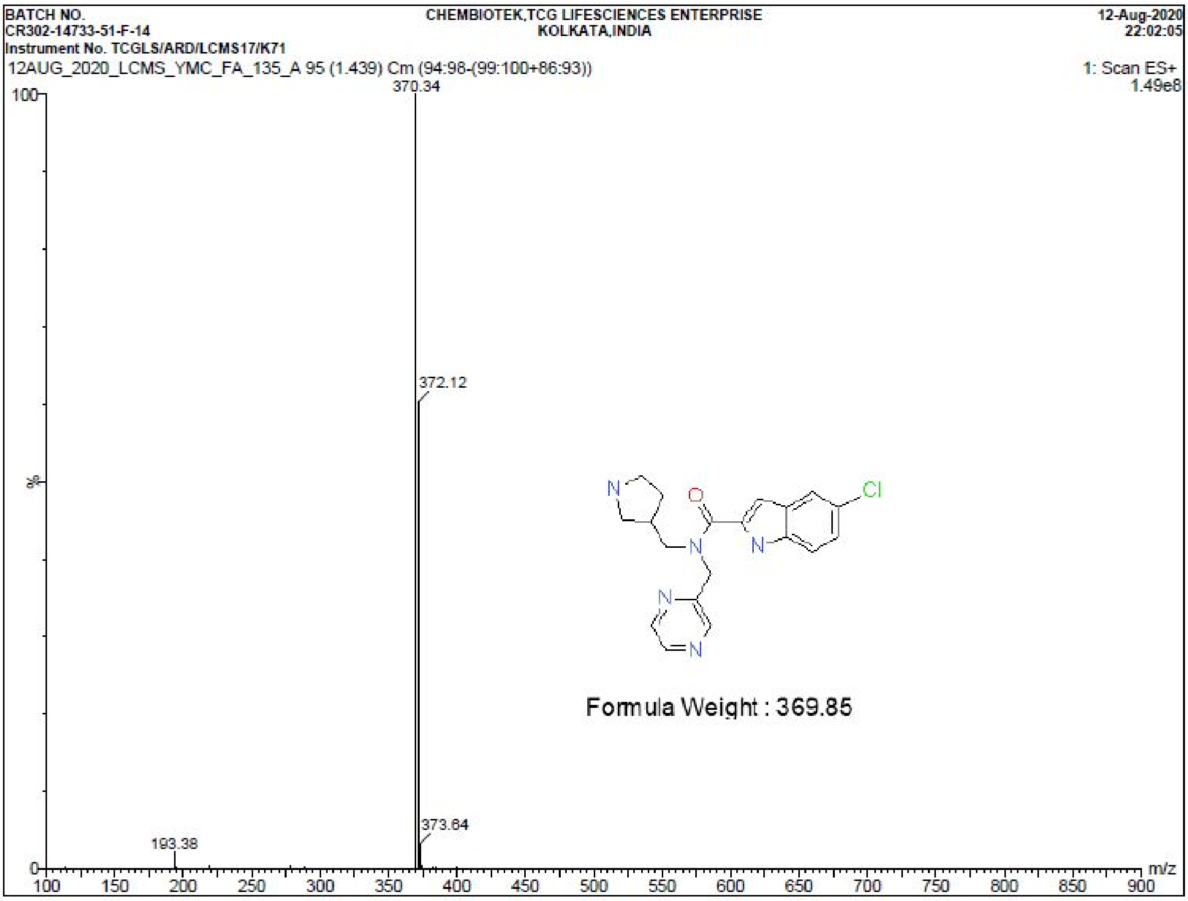

LCMS-6n

	Resultados HRMS		
Instituto de Química	Responsável:	Diego C Andrade	LIE Laboratório Institucional de Espectrometria de Massas
	Data:	06/10/22	
	**Laboratório Institucional de Espectrometria de Massas**		

Prof. Dr. Luiz Carlos Dias
Aluno Anwar Shamim
Amostra MMV307

Espectro completo de 50 a 750 m/z em modo positivo

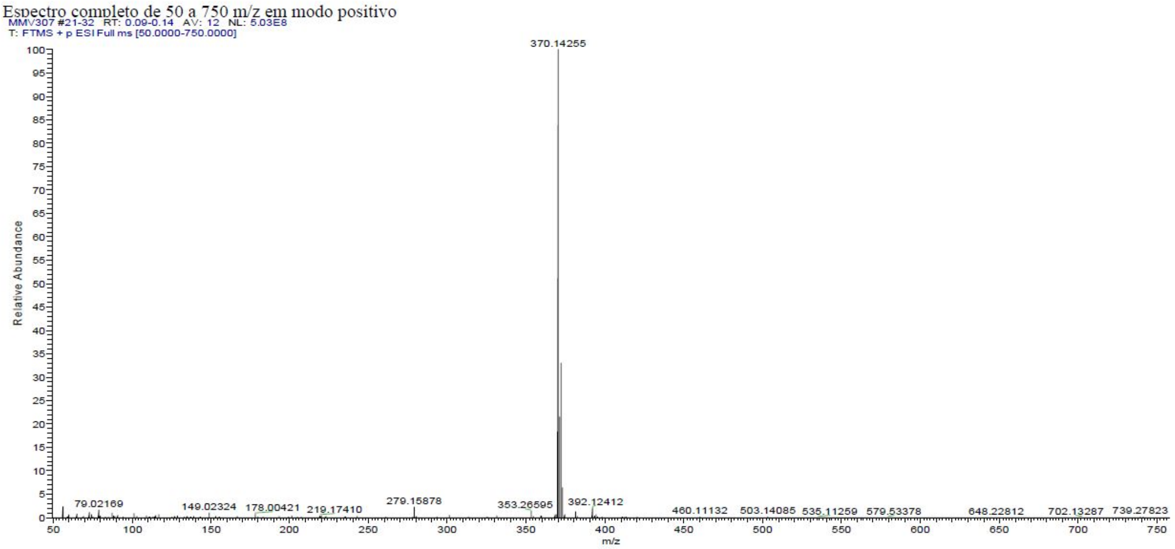

HRMS-6n

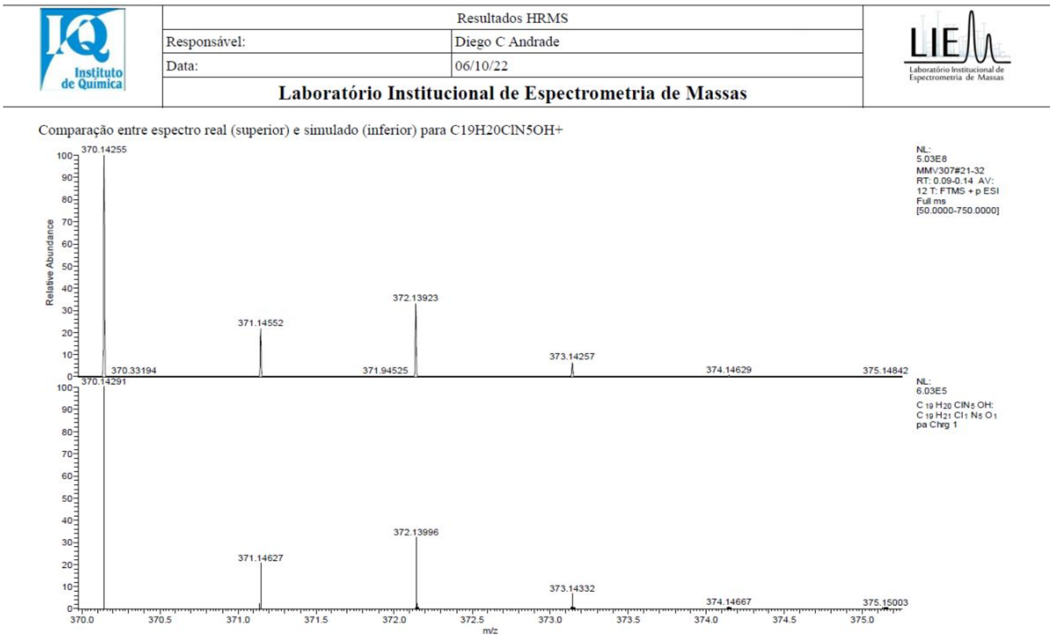

HRMS-6n

5-chloro-N-(pyrrolidin-3-ylmethyl)-N-[[6-(trifluoromethyl)-3-pyridyl]methyl]-1H-indole-2-carboxamide (6o):

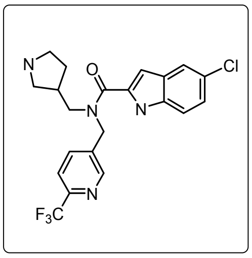

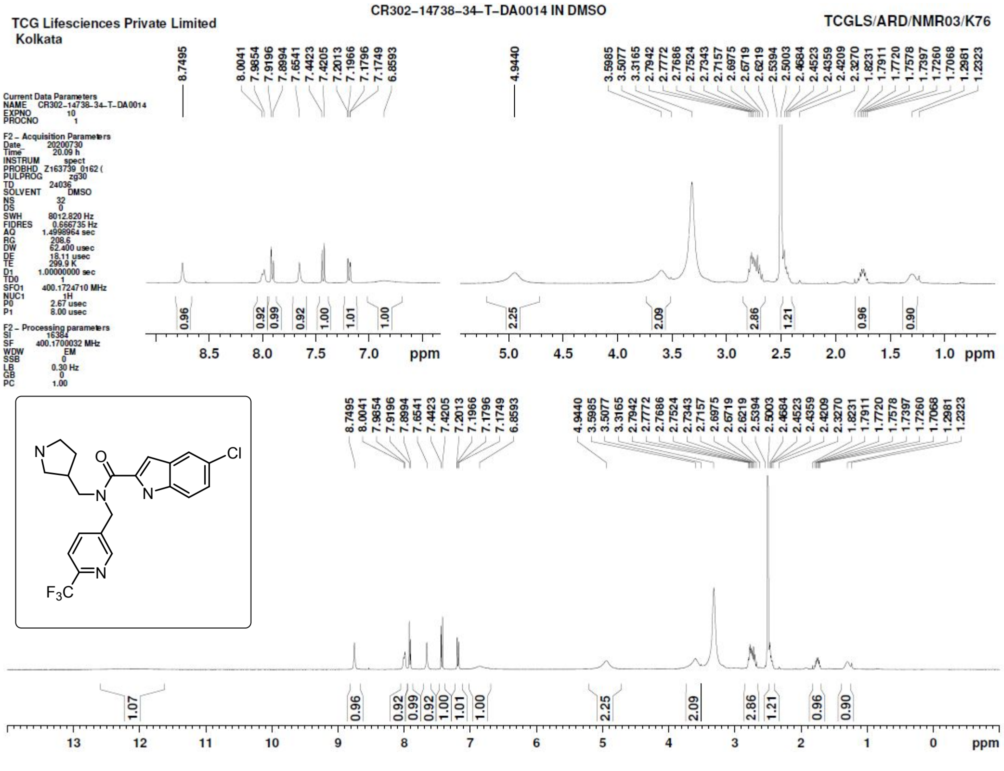

^{1}H NMR (400 MHz, DMSO-d$_6$)-6o

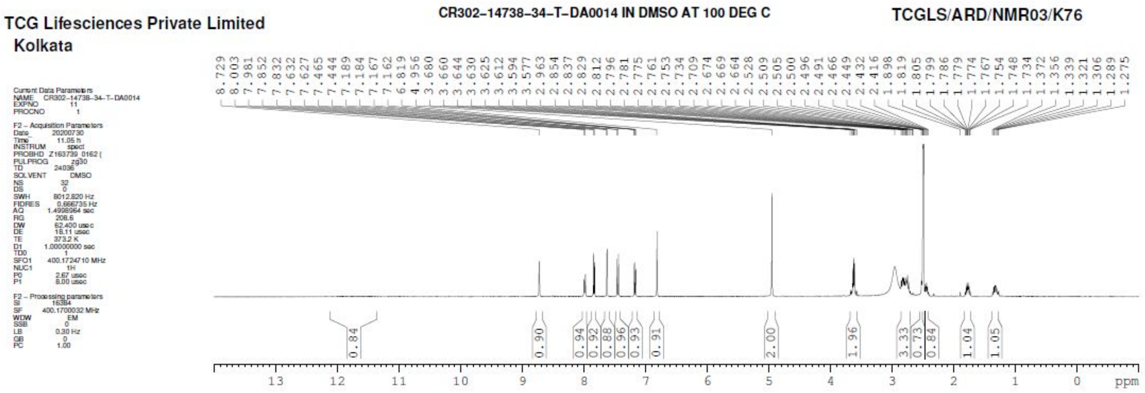

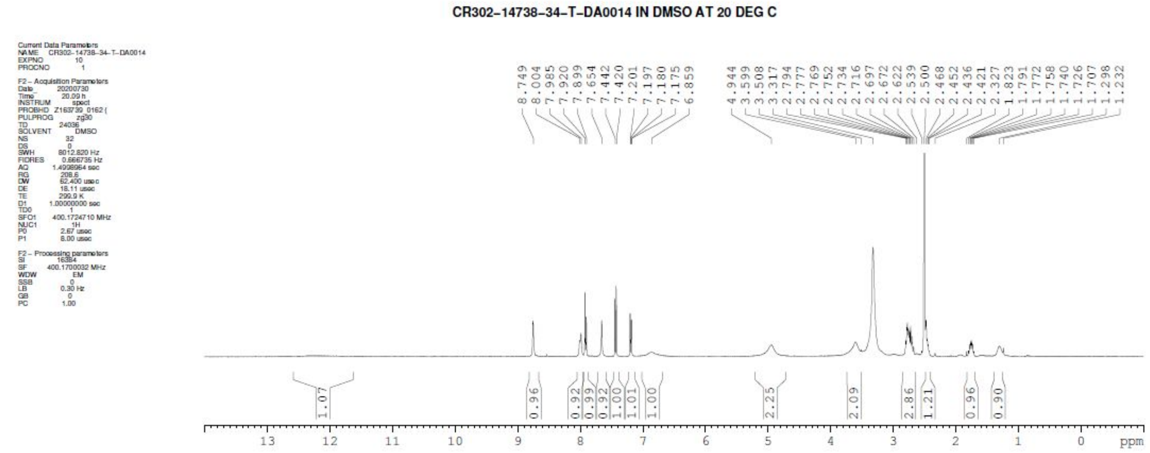

^{1}H NMR (20 °C and 100 °C) (400 MHz, DMSO-d_6)-6o

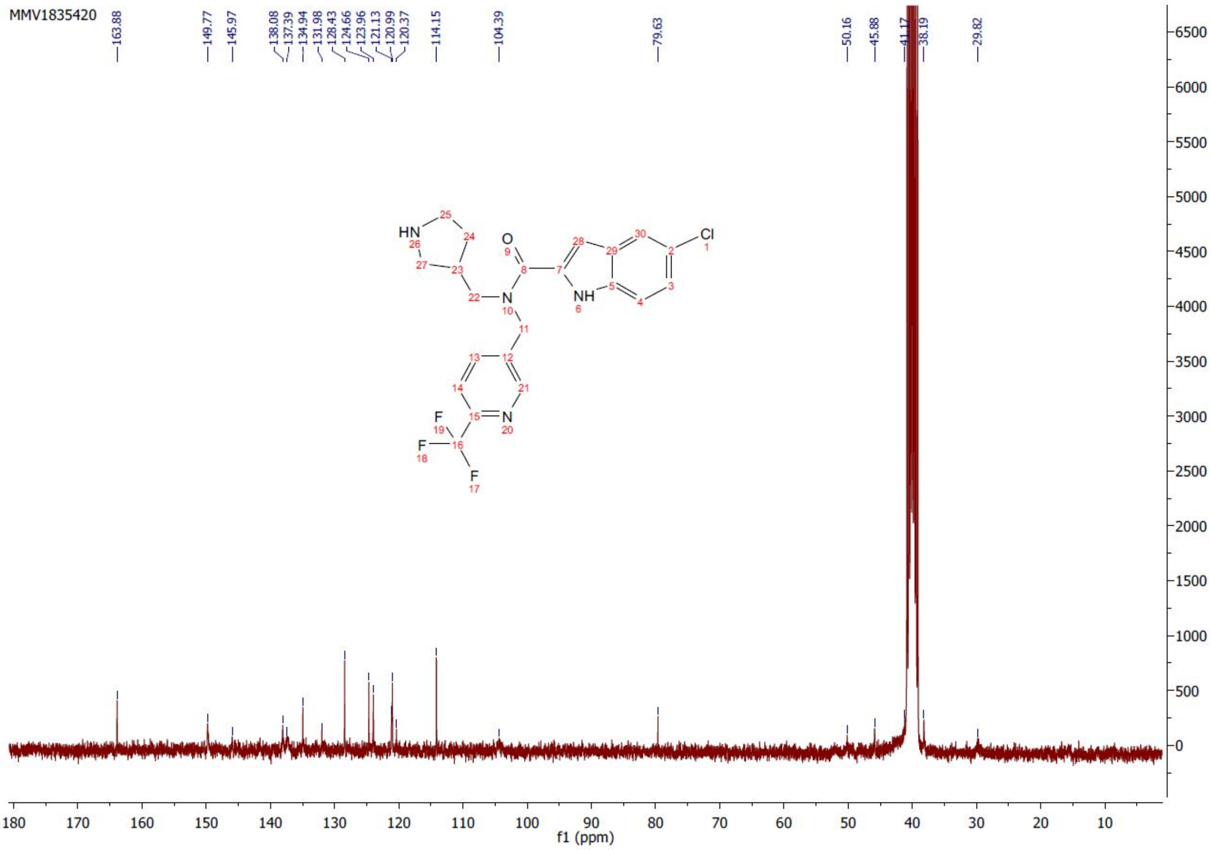

^{13}C NMR (75 MHz, DMSO-d_6)-6º

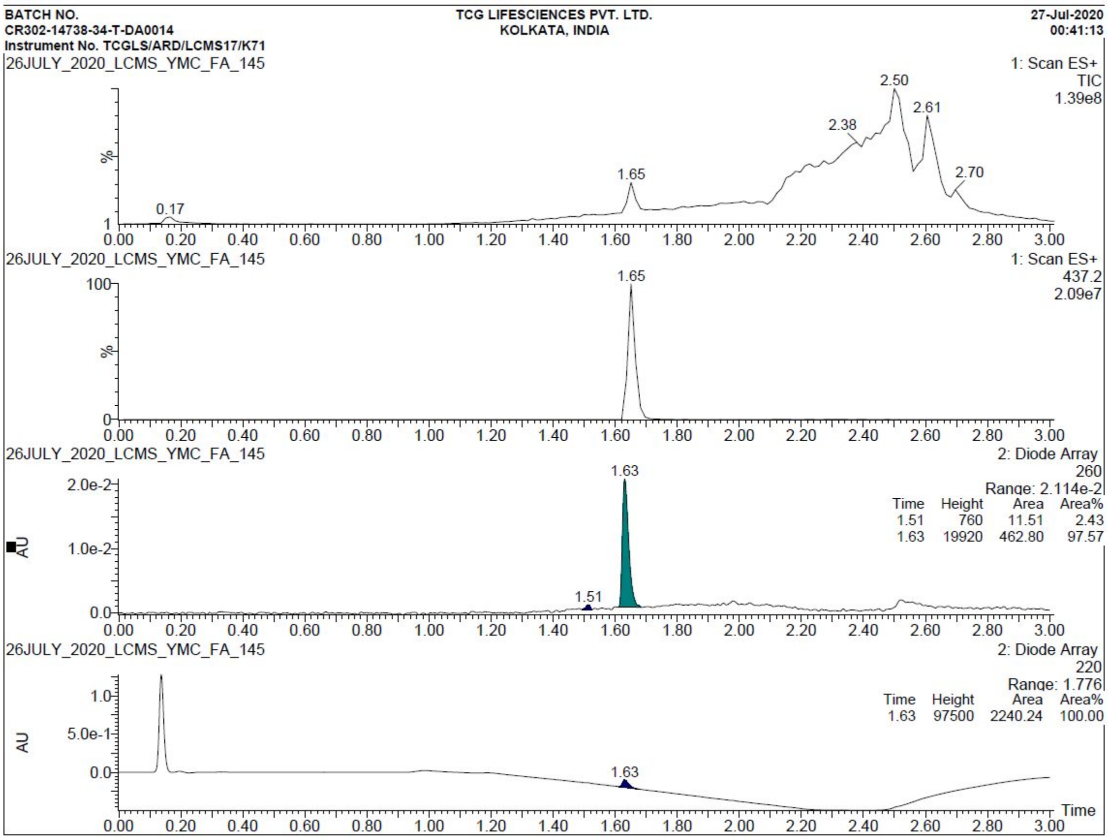
BATCH NO.
CR302-14738-34-T-DA0014
Instrument No. TCGLS/ARD/LCMS17/K71
TCG LIFESCIENCES PVT. LTD.
KOLKATA, INDIA
27-Jul-2020
00:41:13
26JULY_2020_LCMS_YMC_FA_145
1: Scan ES+
TIC
1.39e8
0.17
1.65
2.38
2.50
2.61
2.70
1: Scan ES+
437.2
2.09e7
1.65
2: Diode Array
260
Range: 2.114e-2
Time Height Area Area%
1.51 760 11.51 2.43
1.63 19920 462.80 97.57
1.51
1.63
2: Diode Array
220
Range: 1.776
Time Height Area Area%
1.63 97500 2240.24 100.00
1.63
AU
%
Time

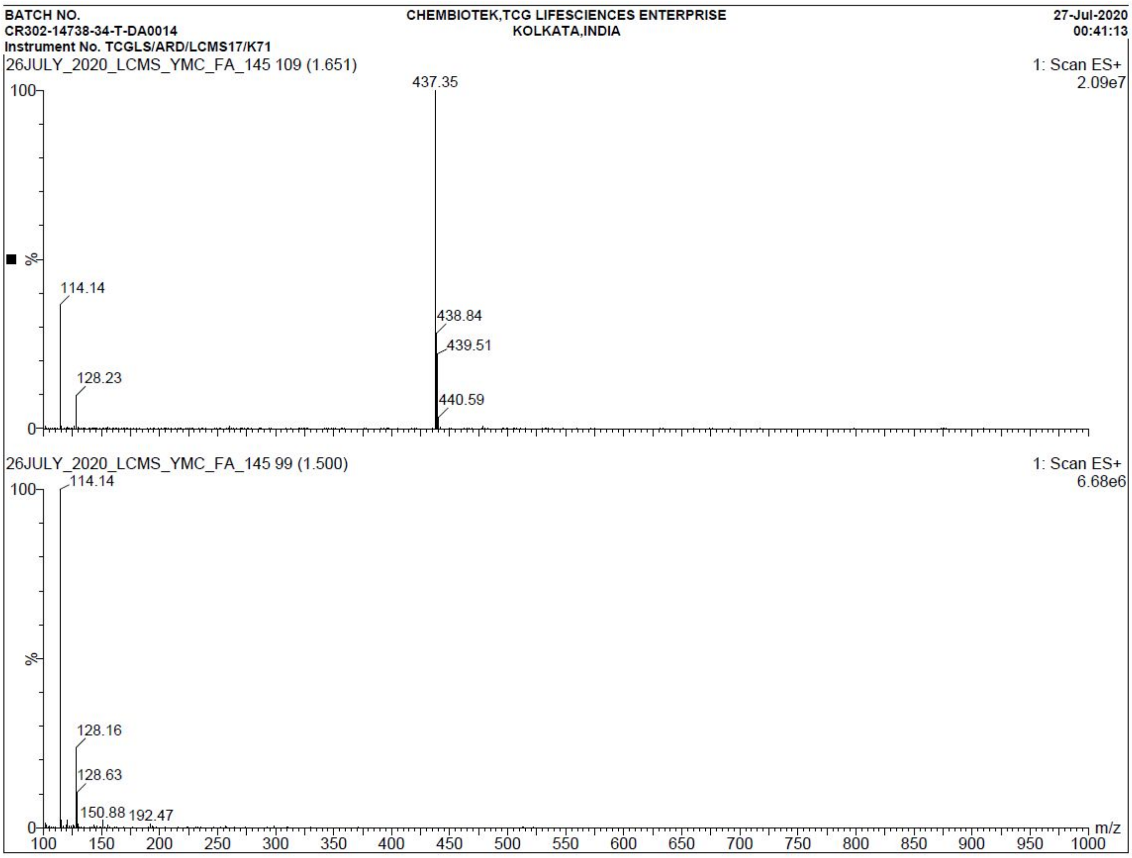
BATCH NO.
CR302-14738-34-T-DA0014
Instrument No. TCGLS/ARD/LCMS17/K71
CHEMBIOTEK,TCG LIFESCIENCES ENTERPRISE
KOLKATA,INDIA
27-Jul-2020
00:41:13
26JULY_2020_LCMS_YMC_FA_145 109 (1.651)
1: Scan ES+
2.09e7
437.35
114.14
438.84
439.51
128.23
440.59
26JULY_2020_LCMS_YMC_FA_145 99 (1.500)
1: Scan ES+
6.68e6
114.14
128.16
128.63
150.88
192.47
m/z

LCMS-6o

	Resultados HRMS		
	Responsável:	Diego C Andrade	
	Data:	06/10/22	
	**Laboratório Institucional de Espectrometria de Massas**		

Prof. Dr. Luiz Carlos Dias
Aluno Anwar Shamim
Amostra MMV420

Espectro completo de 50 a 750 m/z em modo positivo

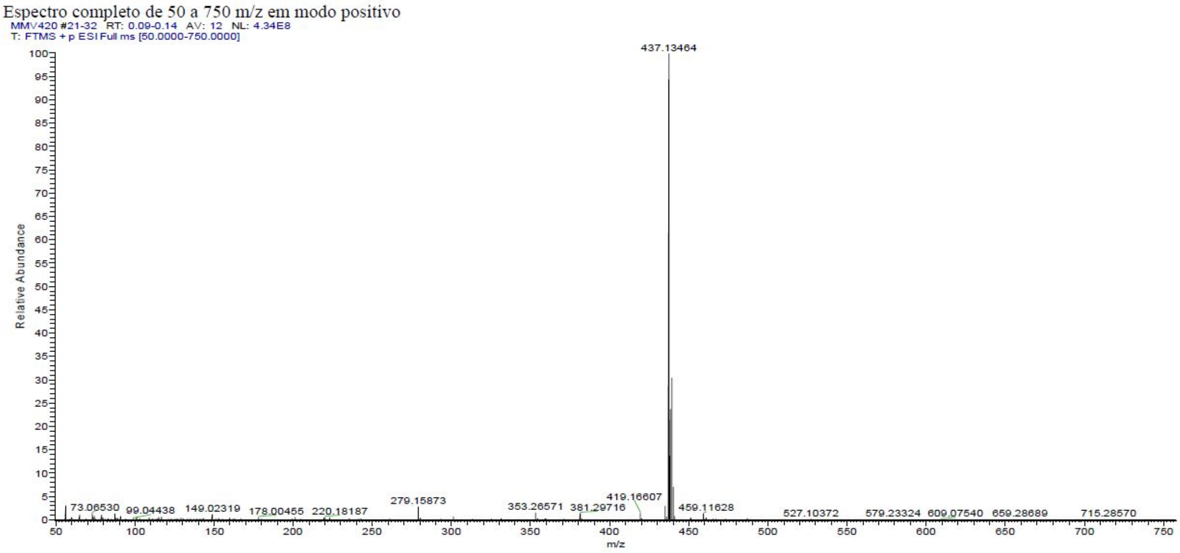

HRMS-6o

	Resultados HRMS		
	Responsável:	Diego C Andrade	
	Data:	06/10/22	
	**Laboratório Institucional de Espectrometria de Massas**		

Comparação entre espectro real (superior) e simulado (inferior) para C21H20ClF3N4OH+

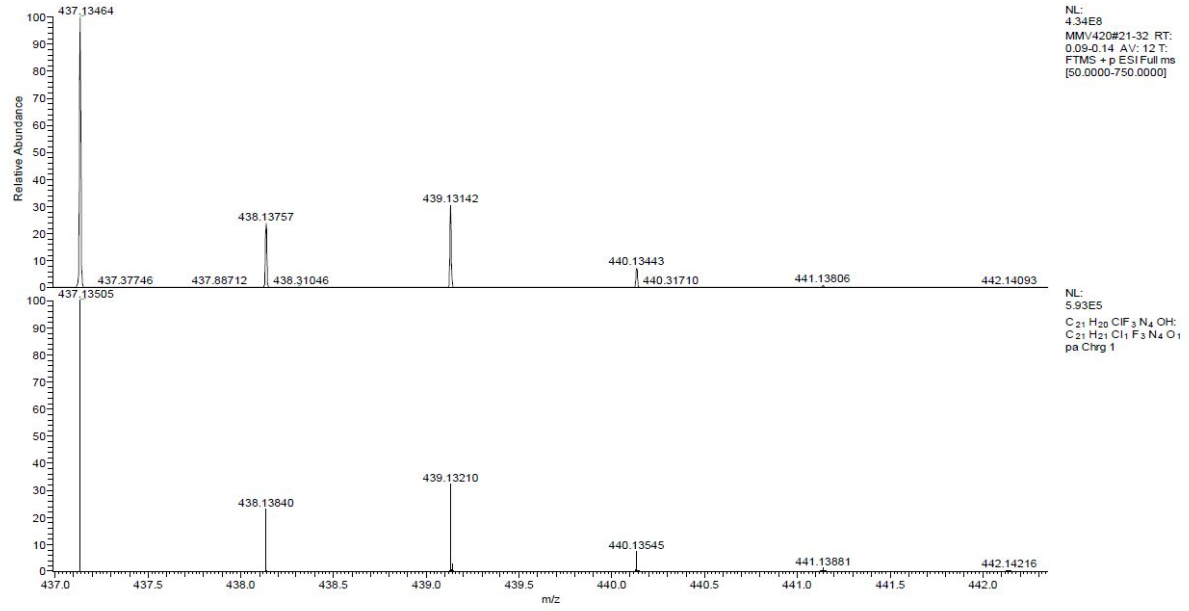

HRMS-6o

5-chloro-N-[(6-methylsulfonyl-3-pyridyl)methyl]-N-(pyrrolidin-3-ylmethyl)-1H-indole-2-carboxamide(6p)

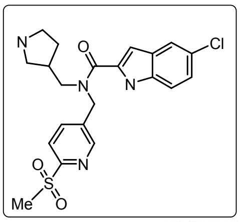

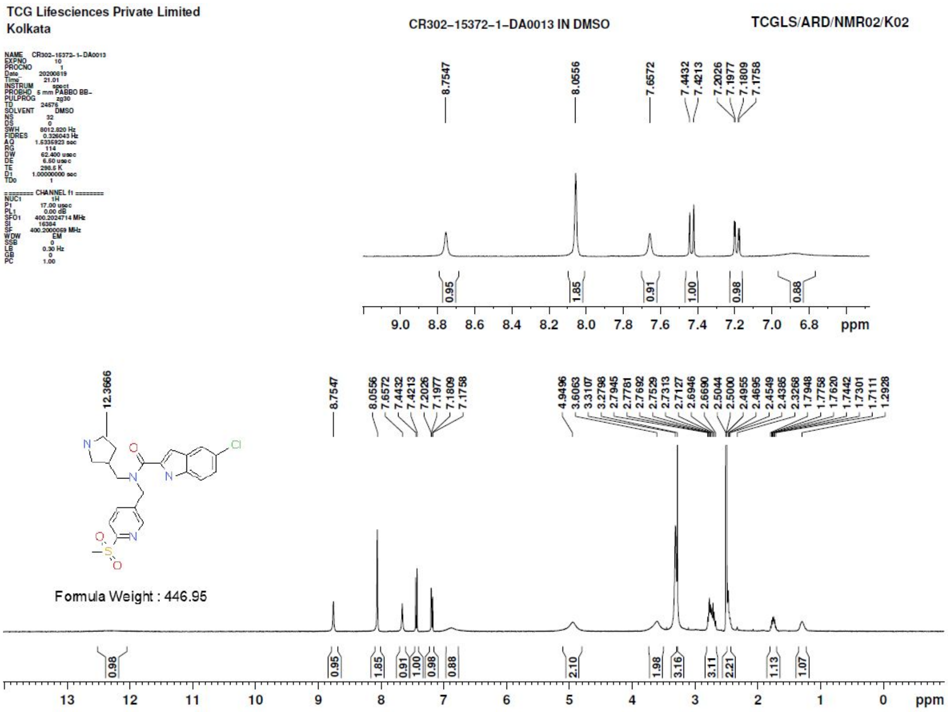

^{1}H NMR (400 MHz, DMSO-d$_6$)-6p

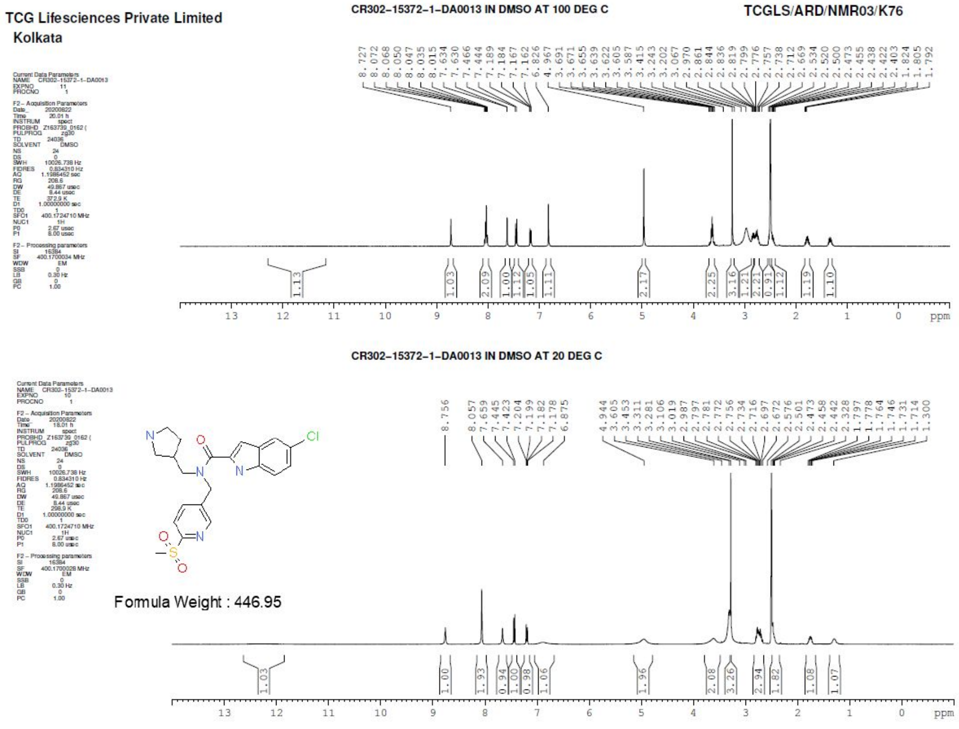

^{1}H NMR (20 °C and 100 °C) (400 MHz, DMSO-d$_6$)-6p

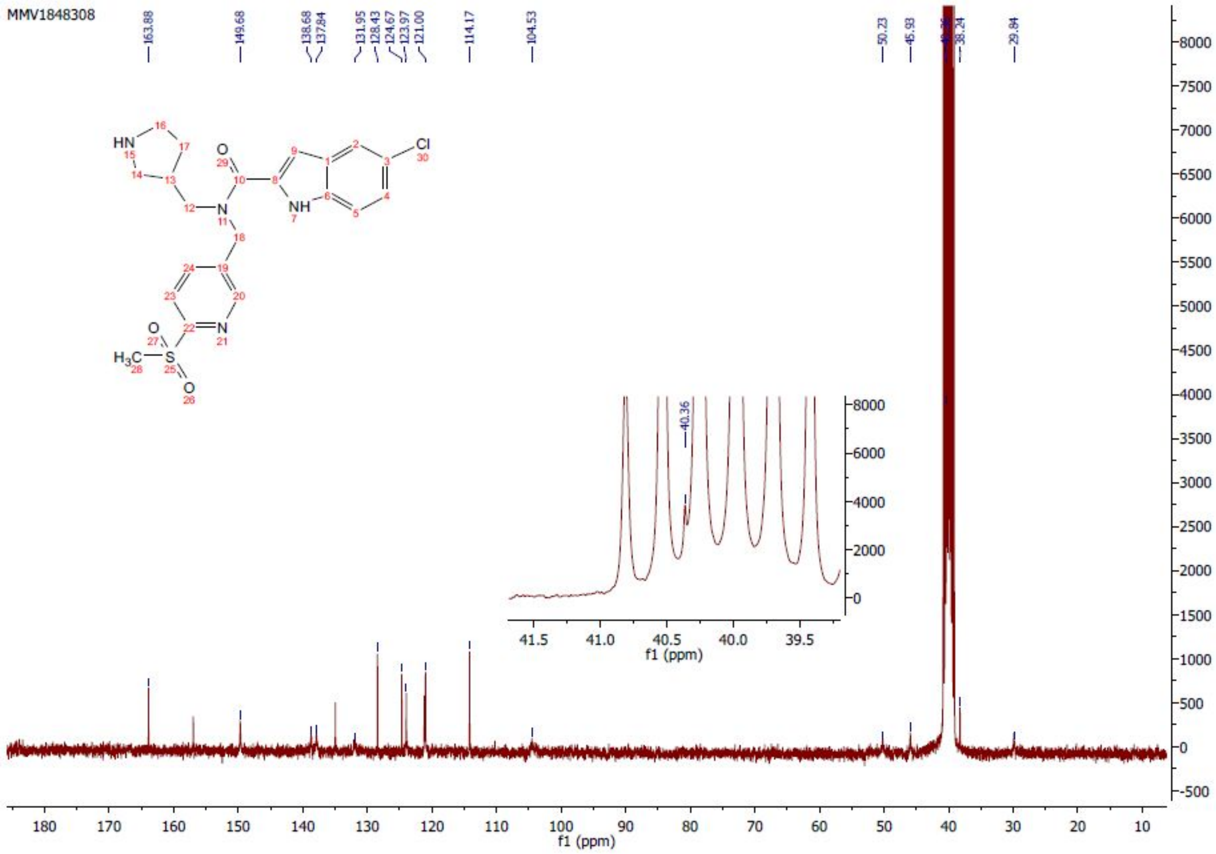

^{13}C NMR (75 MHz, DMSO-d_6)-6p

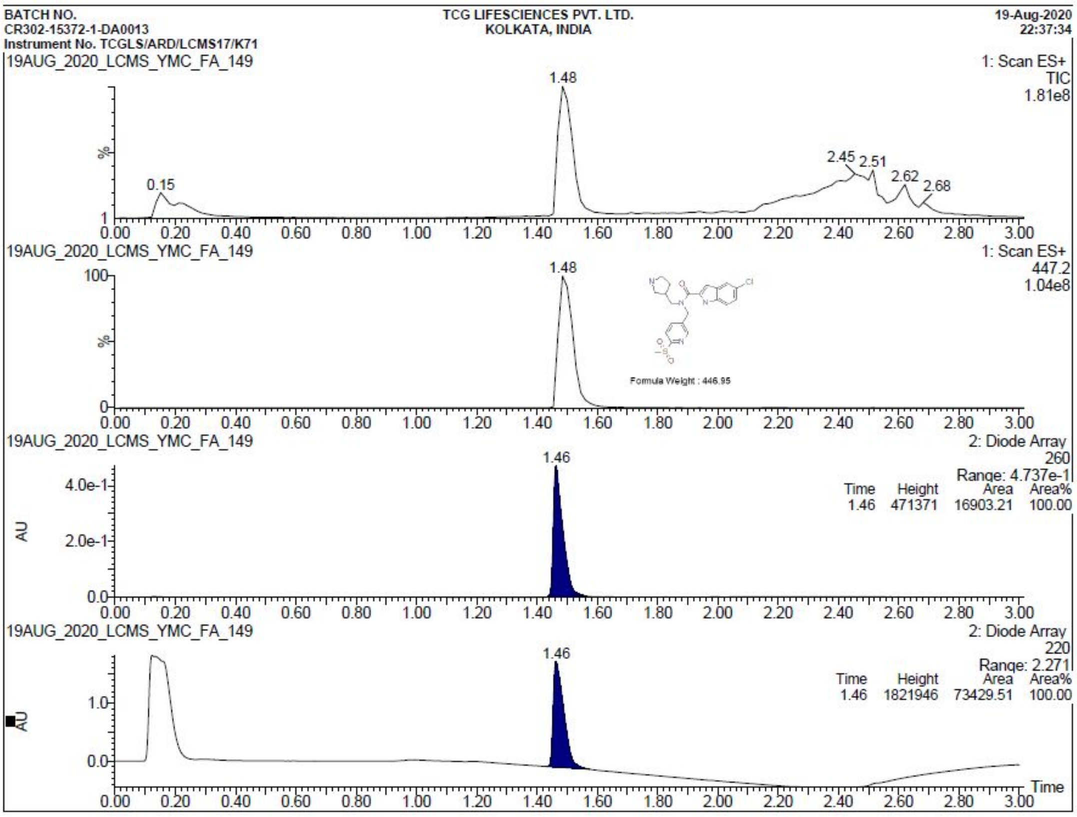

LCMS-6p

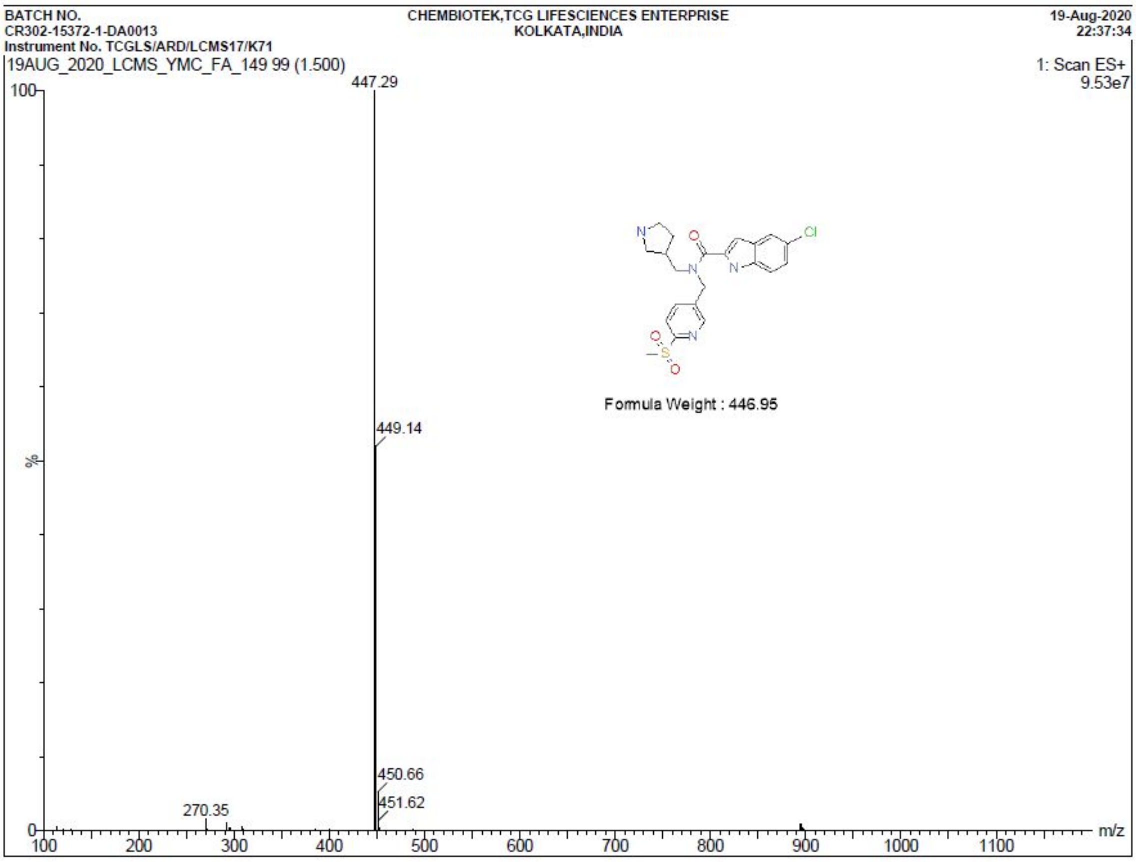

LCMS-6p

Instituto de Química	Resultados HRMS		LIE Laboratório Institucional de Espectrometria de Massas
	Responsável:	Diego C Andrade	
	Data:	06/10/22	
	**Laboratório Institucional de Espectrometria de Massas**		

Prof. Dr. Luiz Carlos Dias
Aluno Anwar Shamim
Amostra MMV308

Espectro completo de 50 a 750 m/z em modo positivo

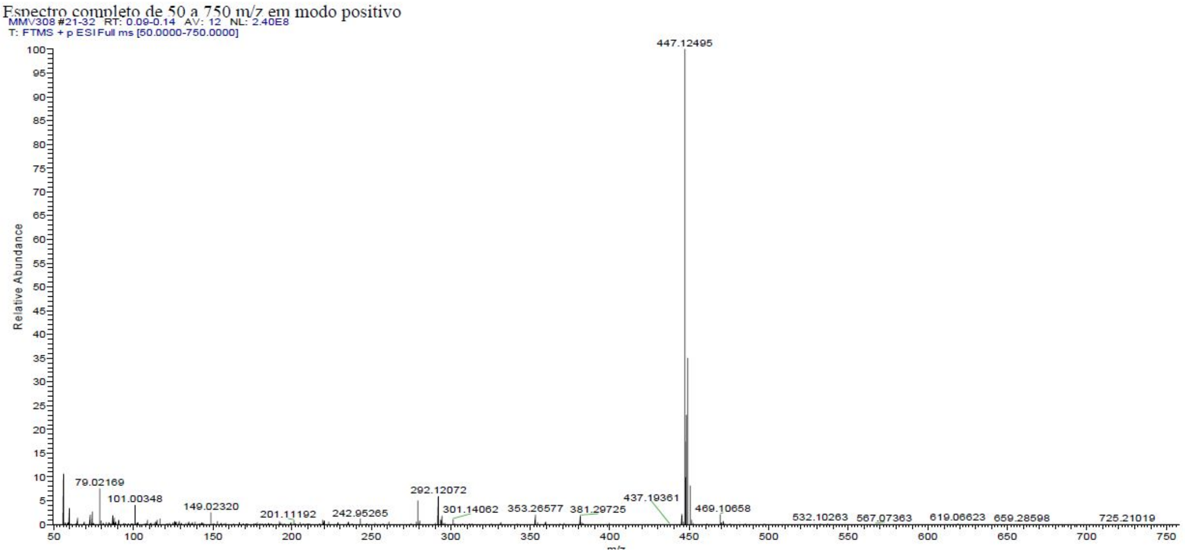

HRMS-6p

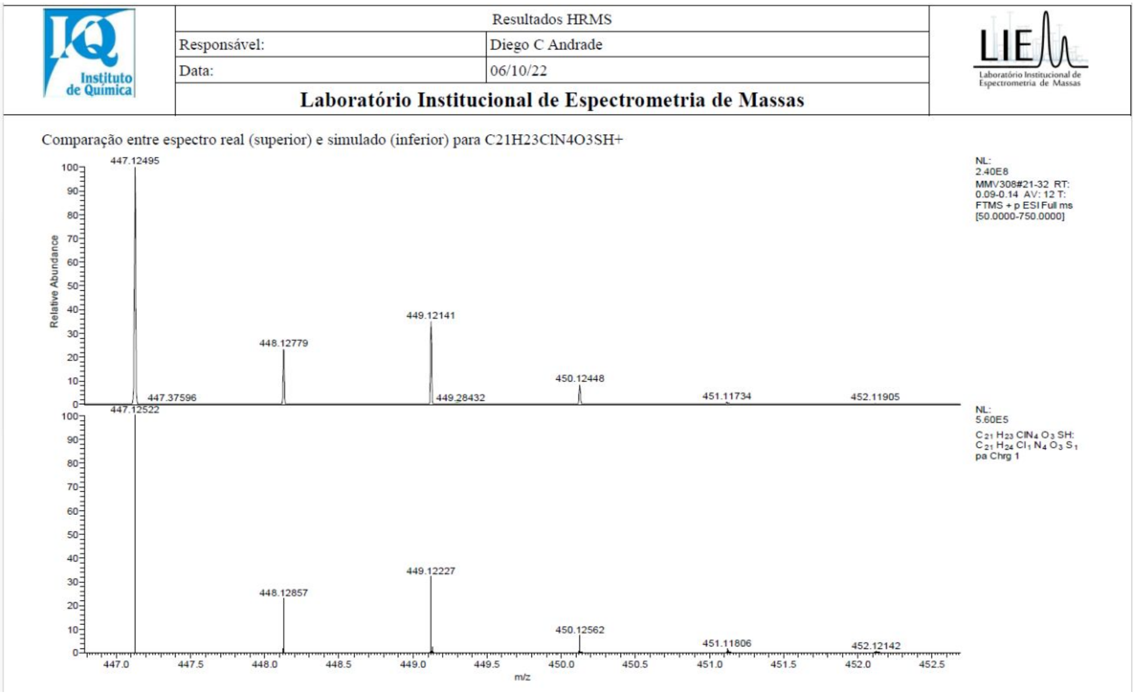

HRMS-6p

5-chloro-N-[2-(methylamino)ethyl]-N-(pyrrolidin-3-ylmethyl)-1H-indole-2-carboxamide (6q)

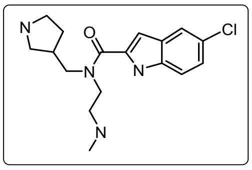

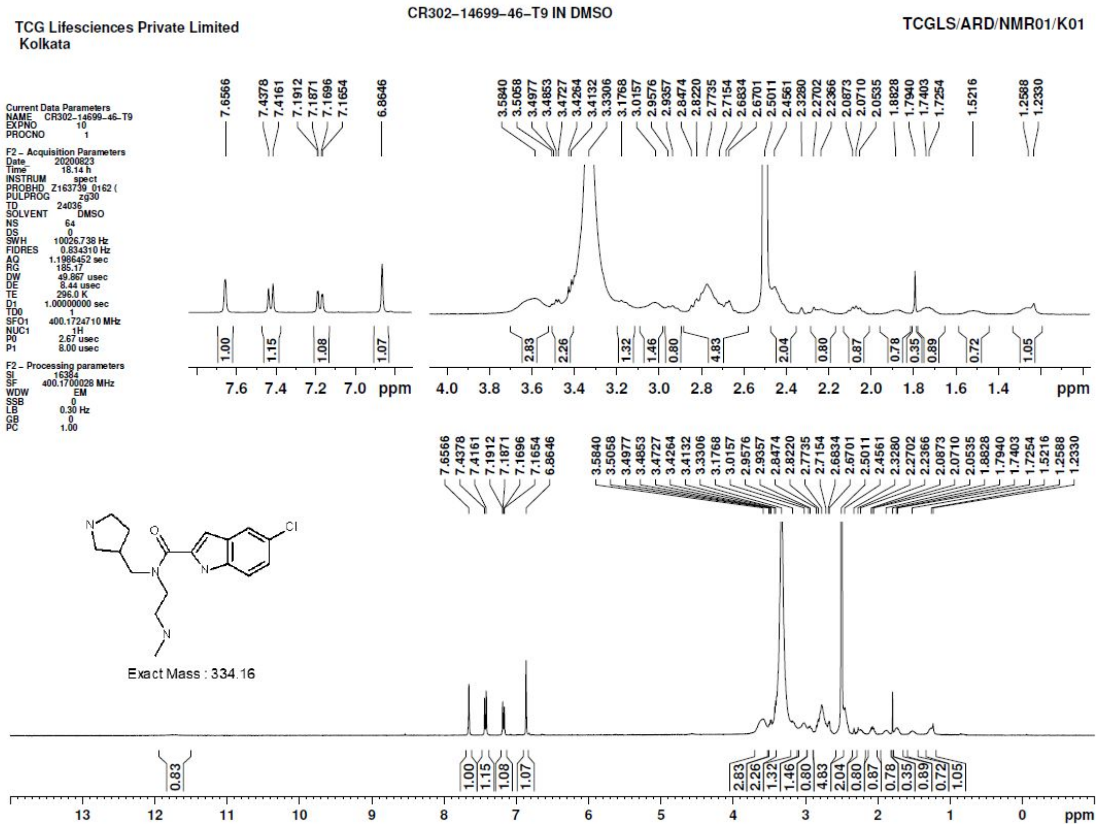

^{1}H NMR (400 MHz, DMSO-d$_6$)-6q

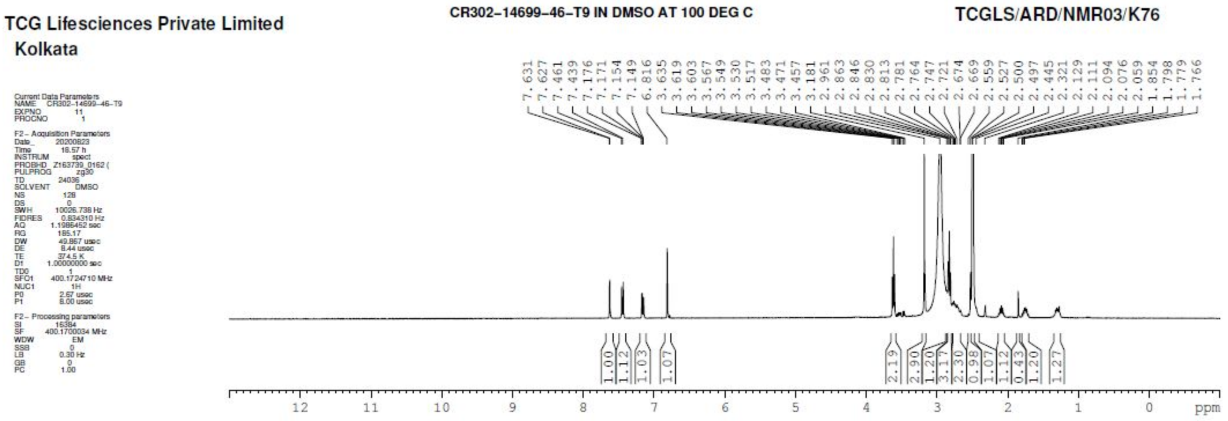

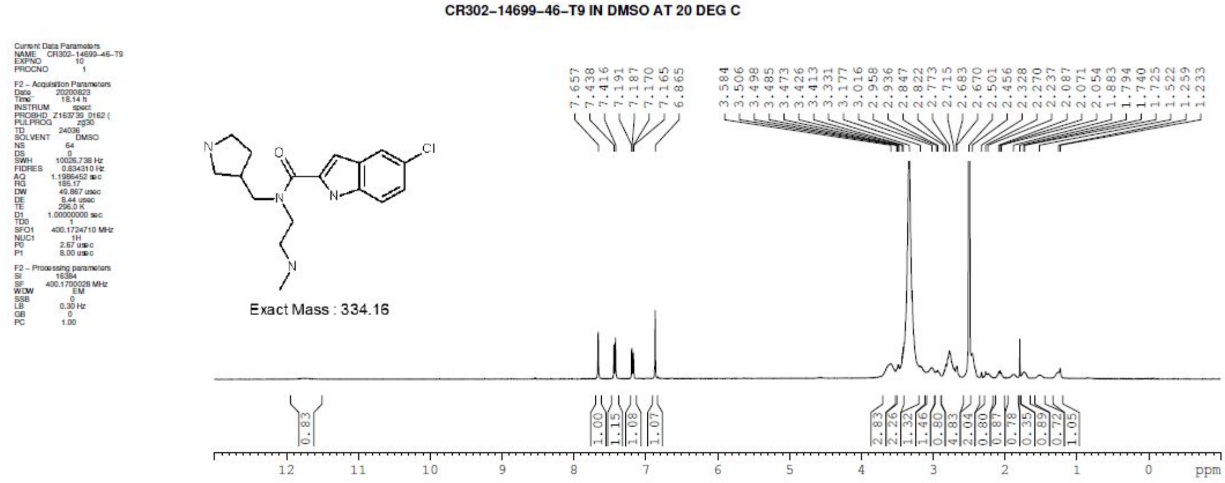

^{1}H NMR (20 °C and 100 °C) (400 MHz, DMSO-d$_6$)-6q

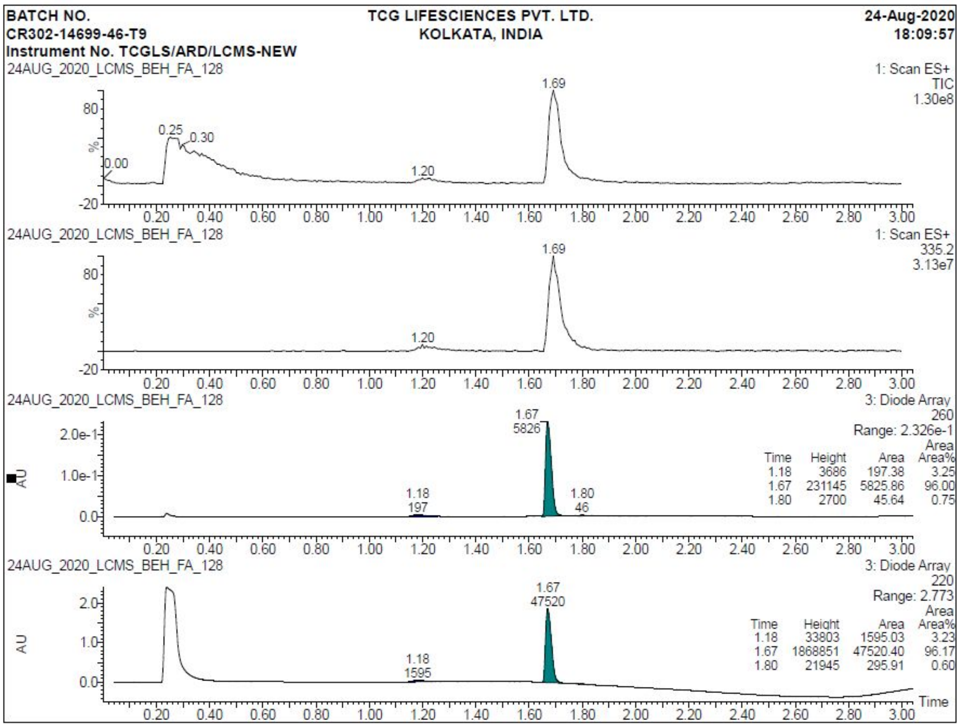

LCMS-6q

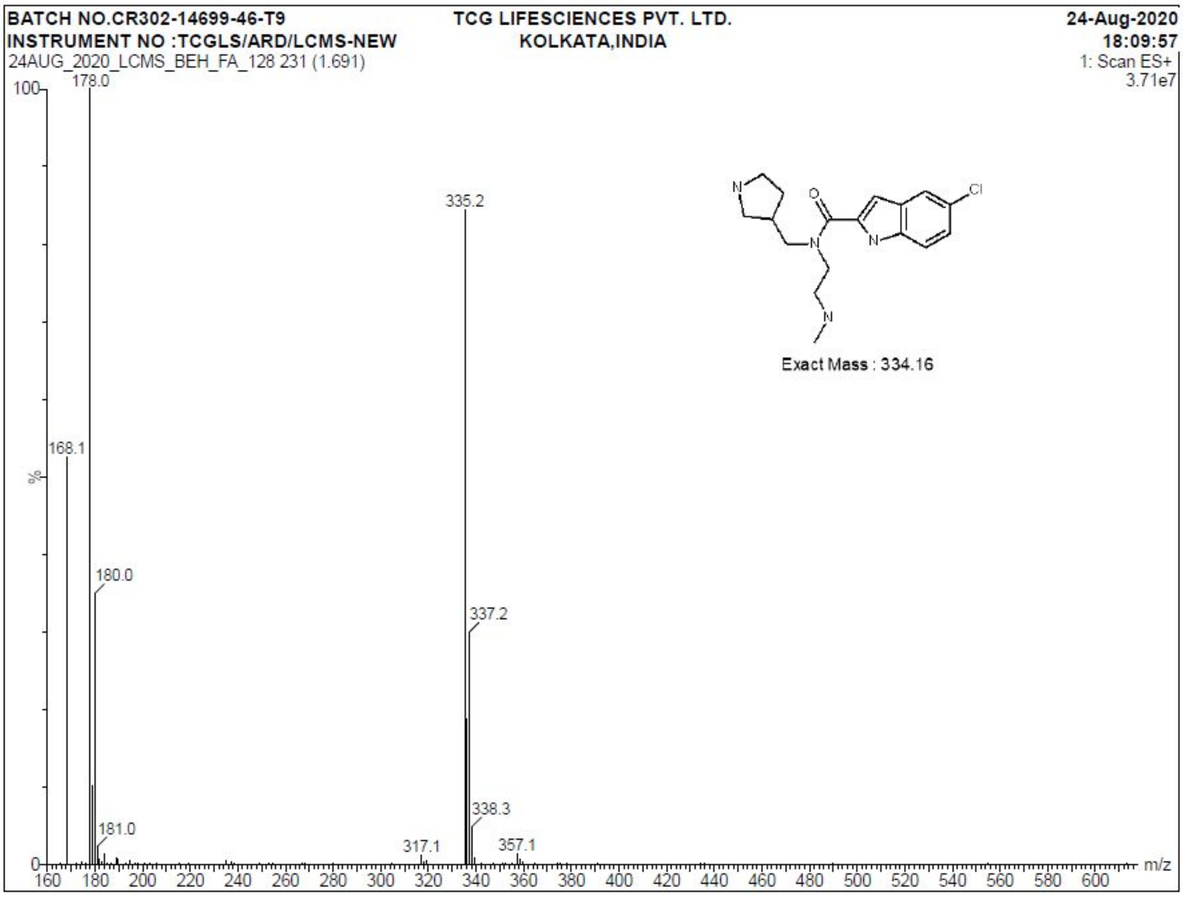

LCMS-6q

Instituto de Química	Resultados HRMS		LIE Laboratório Institucional de Espectrometria de Massas
	Responsável:	Diego C Andrade	
	Data:	06/10/22	
	**Laboratório Institucional de Espectrometria de Massas**		

Prof. Dr. Luiz Carlos Dias
Aluno Anwar Shamim
Amostra MMV318

Espectro completo de 50 a 750 m/z em modo positivo

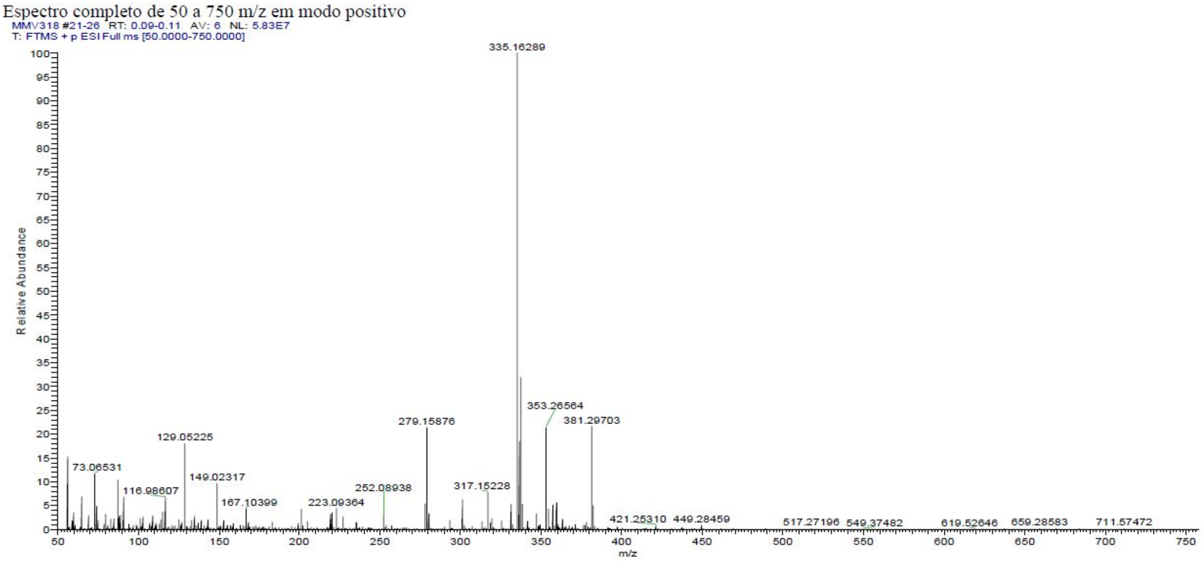

HRMS-6q

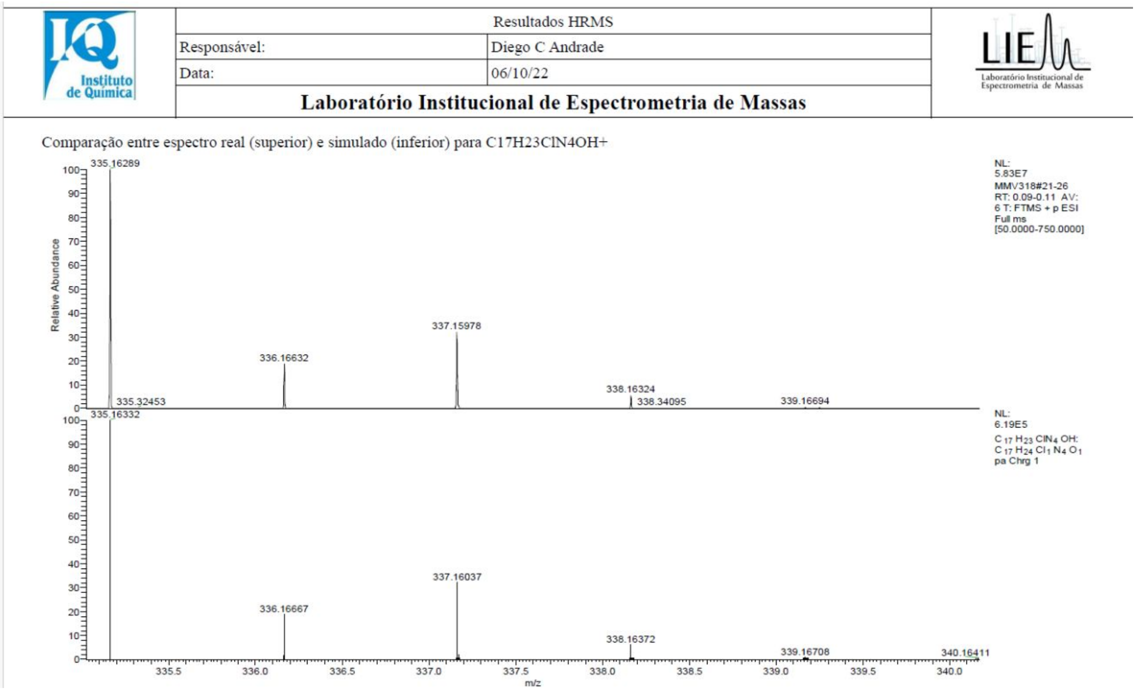

HRMS-6q

5-chloro-N-[2-(methylamino)ethyl]-N-(3-pyridylmethyl)-1H-indole-2-carboxamide (6u)

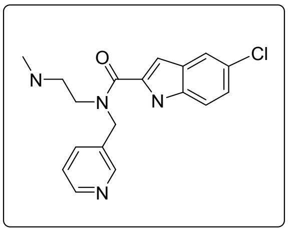

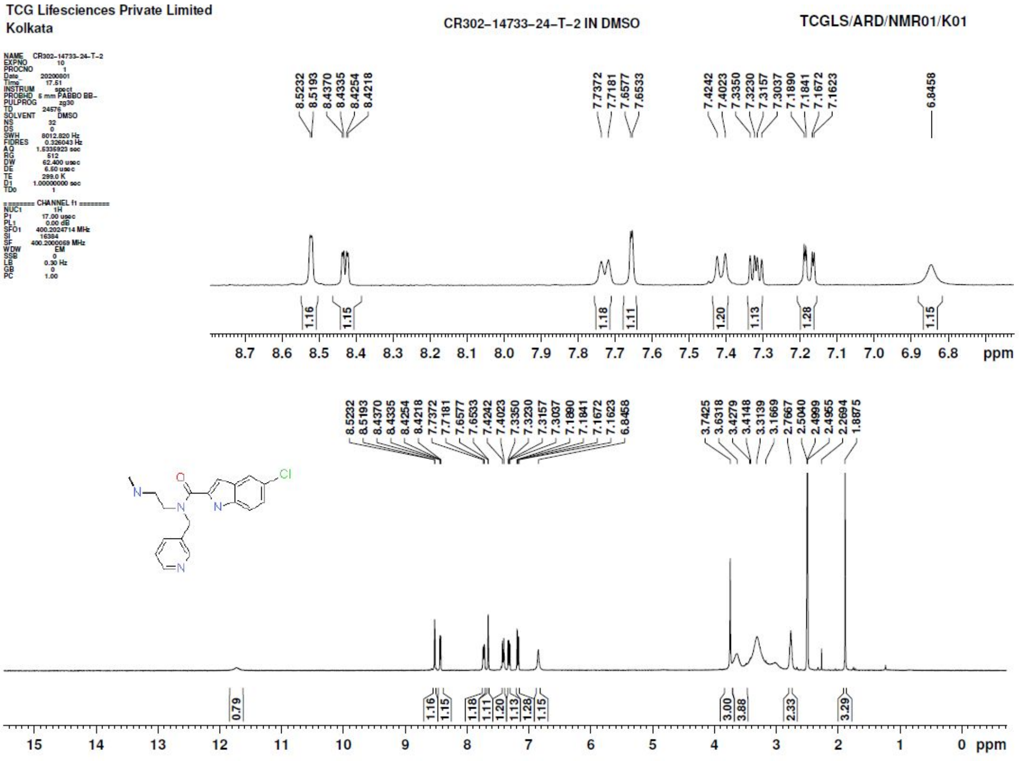

^{1}H NMR (400 MHz, DMSO-d$_6$)-6u

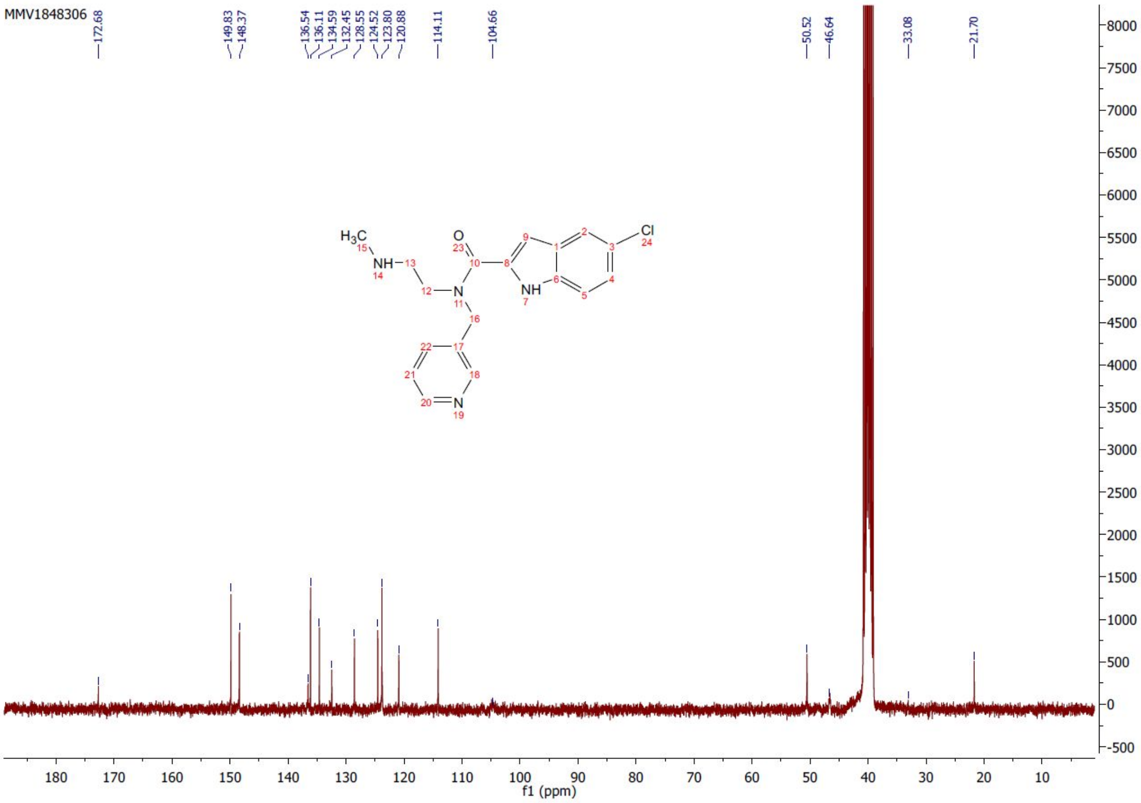

^{13}C NMR (75 MHz, DMSO-d$_6$)-6u

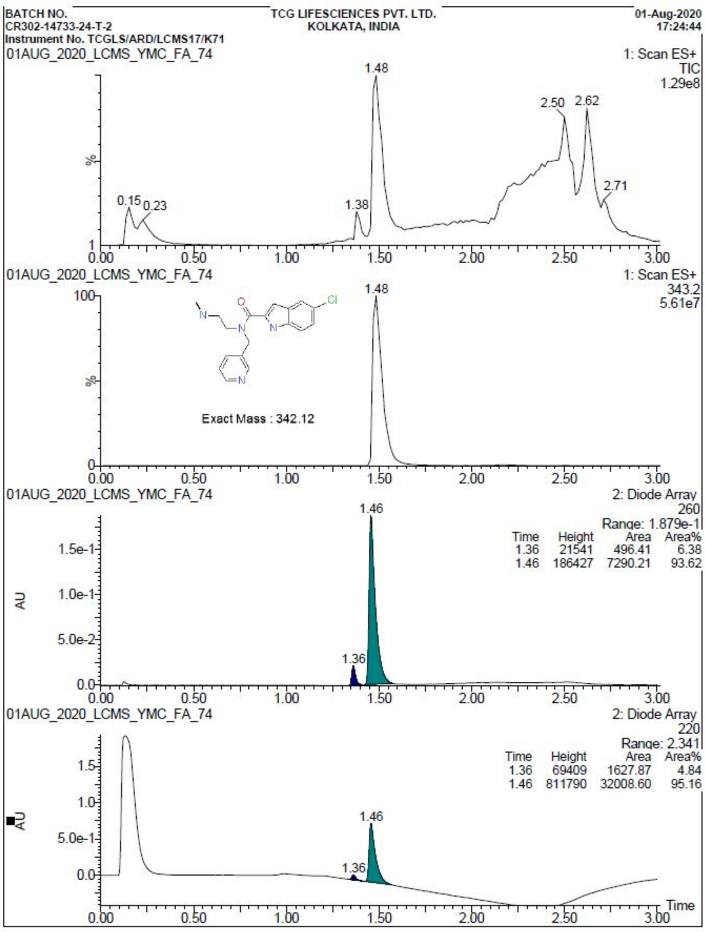

LCMS-6u

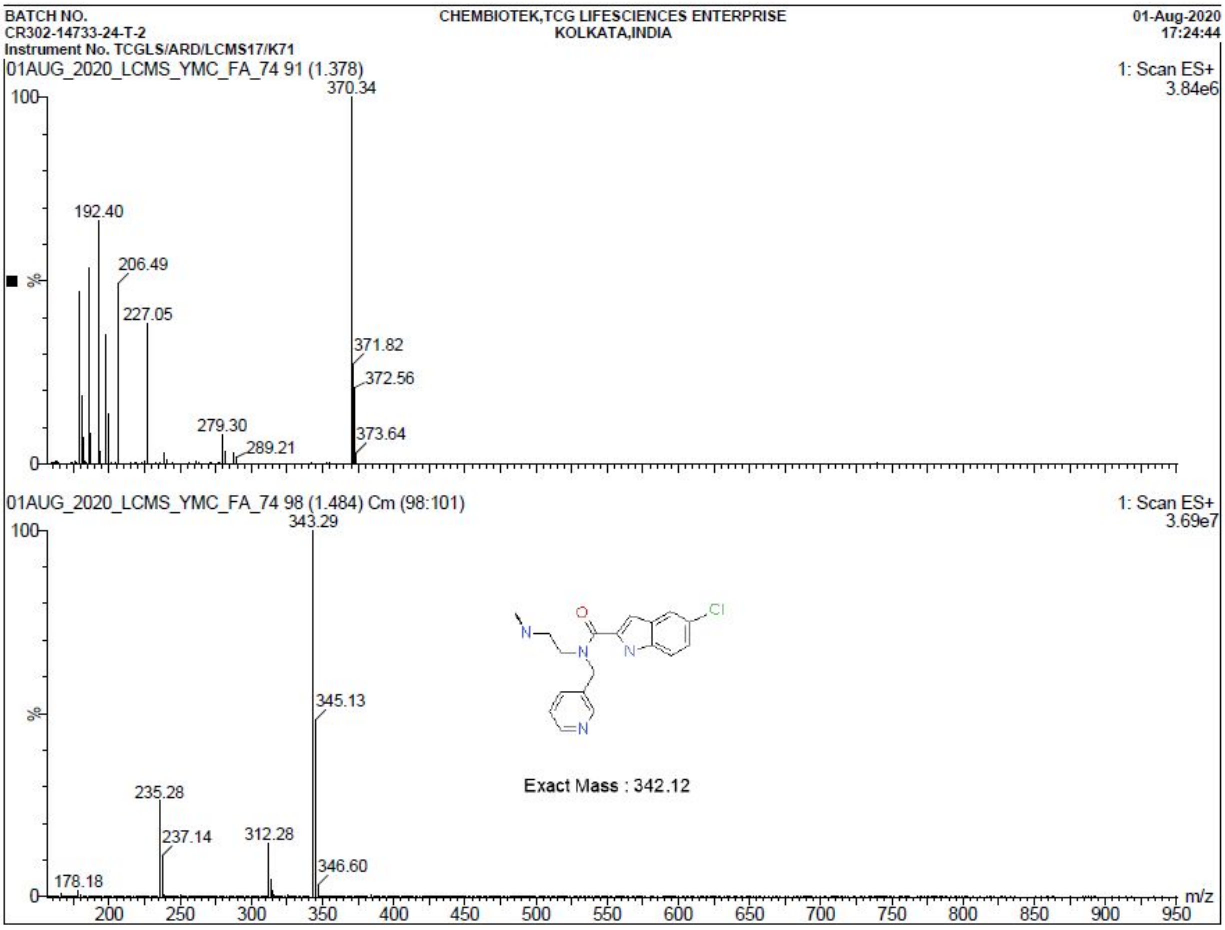

LCMS-6u

	Resultados HRMS	
	Responsável:	Diego C Andrade
	Data:	06/10/22
	Laboratório Institucional de Espectrometria de Massas	

Prof. Dr. Luiz Carlos Dias
Aluno Anwar Shamim
Amostra MMV306

Espectro completo de 50 a 750 m/z em modo positivo

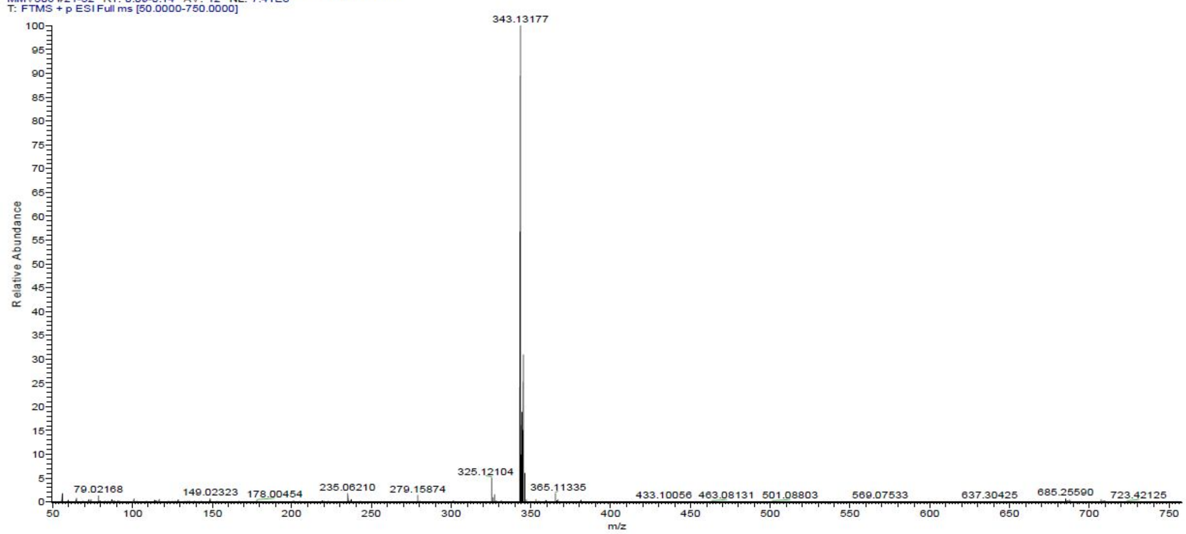

HRMS-6u

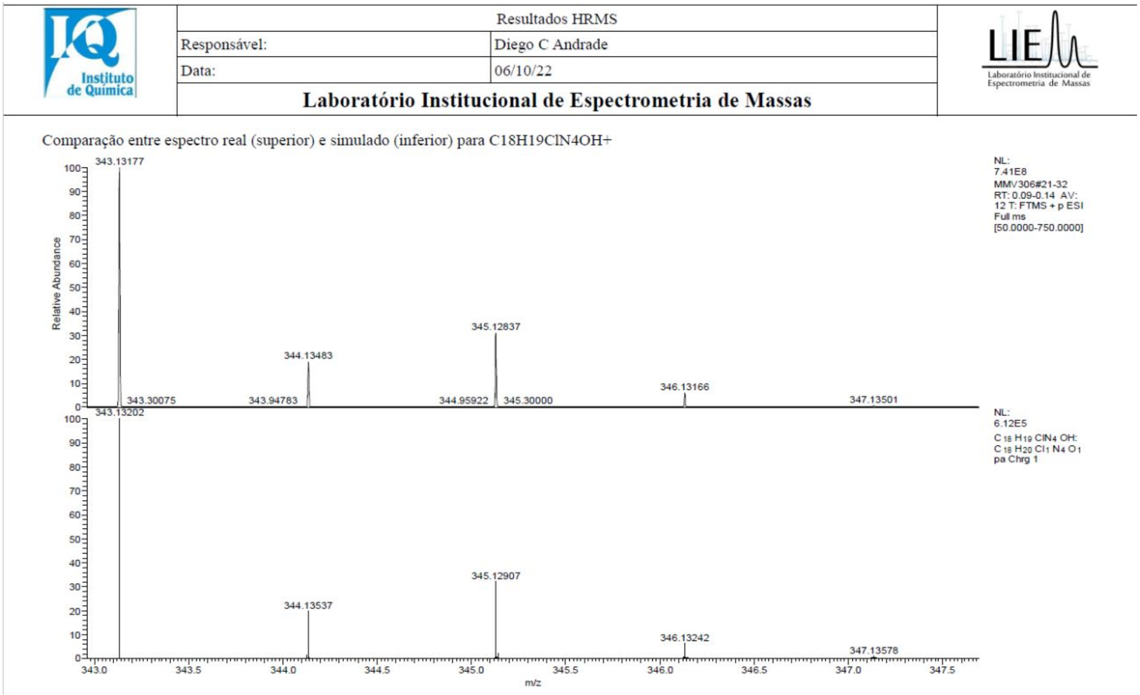

HRMS-6u

5-chloro-N-(3-pyridylmethyl)-N-pyrrolidin-3-yl-1H-indole-2-carboxamide (6v)

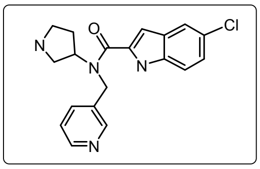

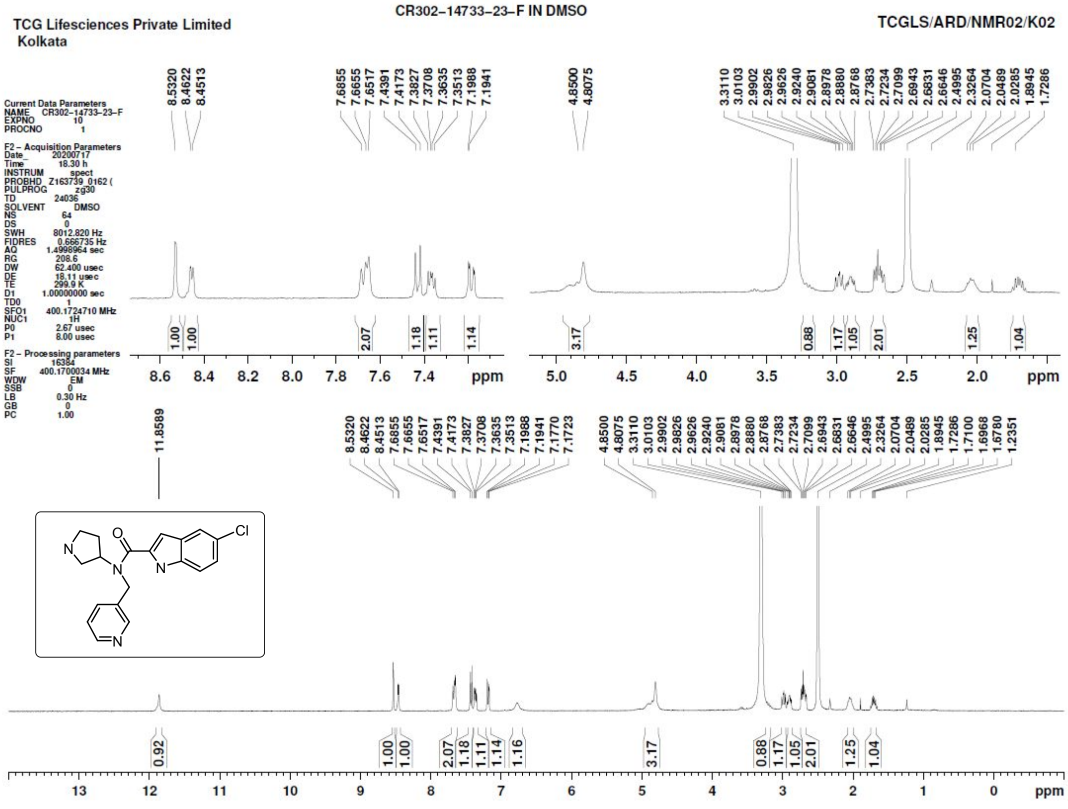

^{1}H NMR (400 MHz, DMSO-d$_6$)-6v

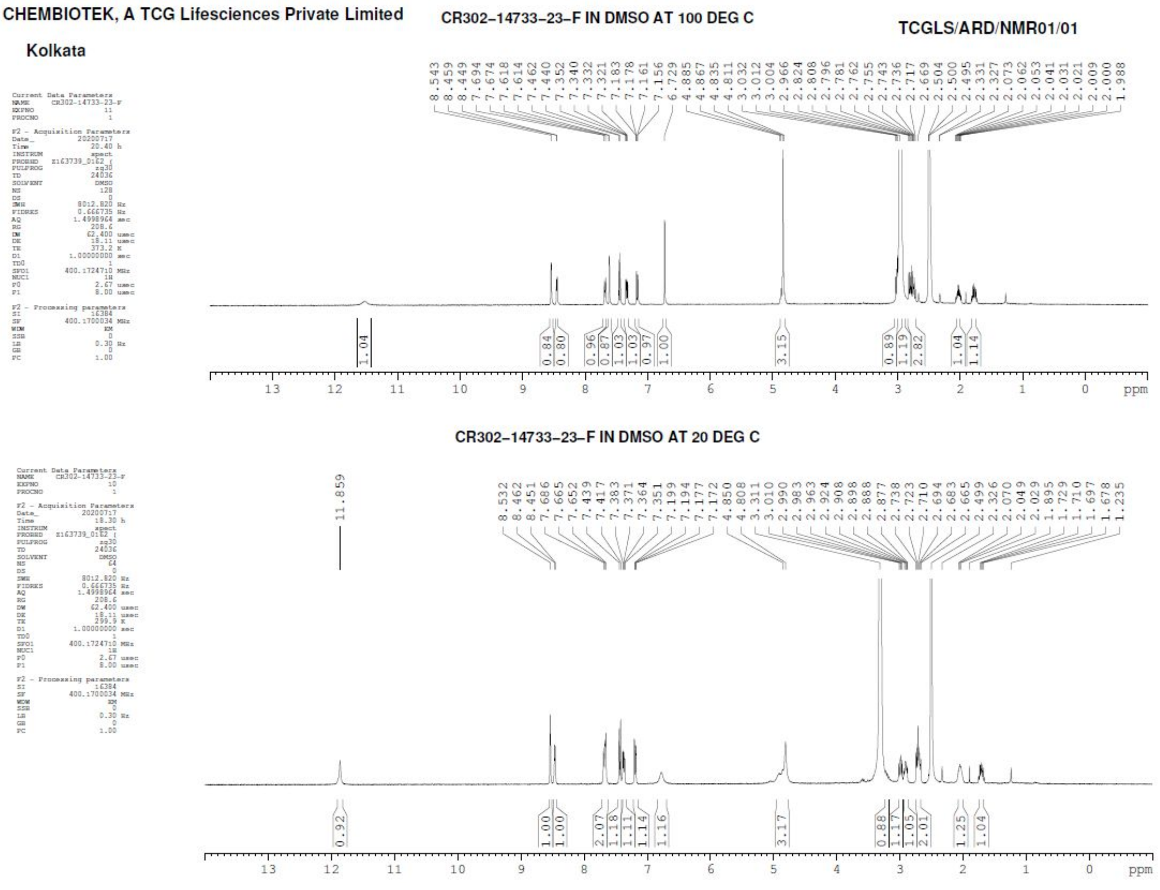

^{1}H NMR (20 °C and 100 °C) (400 MHz, DMSO-d$_6$)-6v

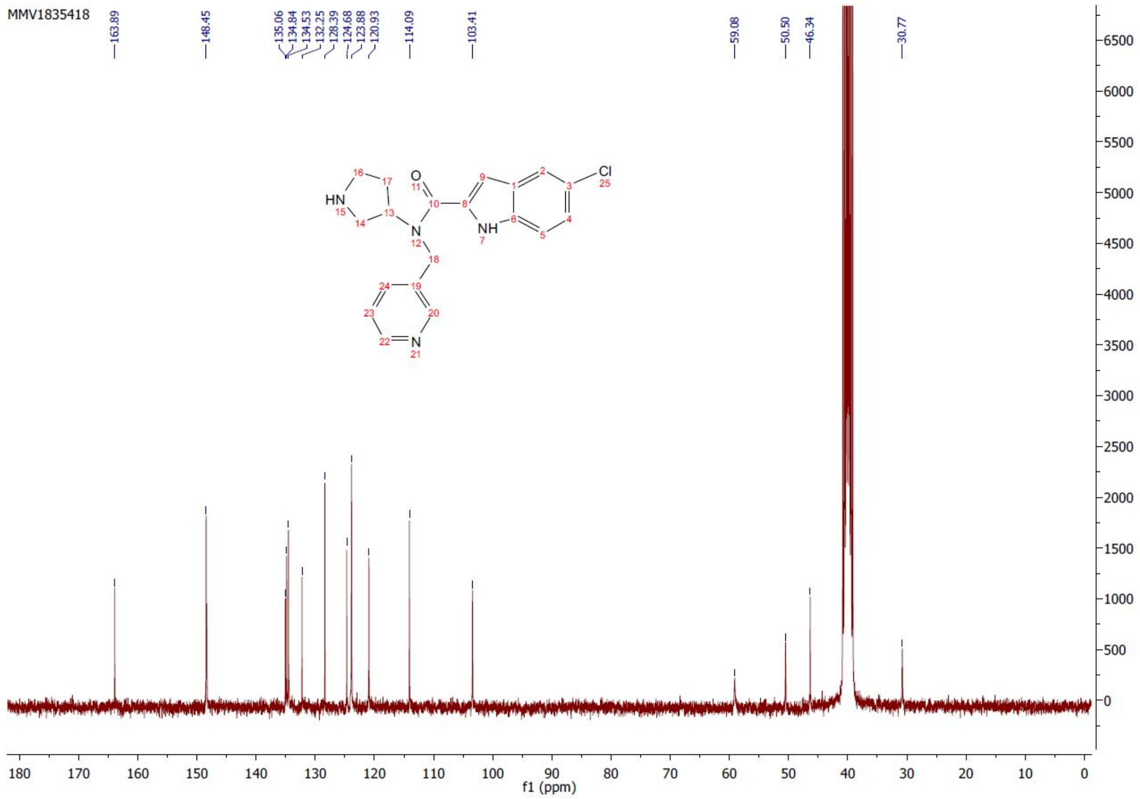

^{13}C NMR (75 MHz, DMSO-d$_6$)-6v

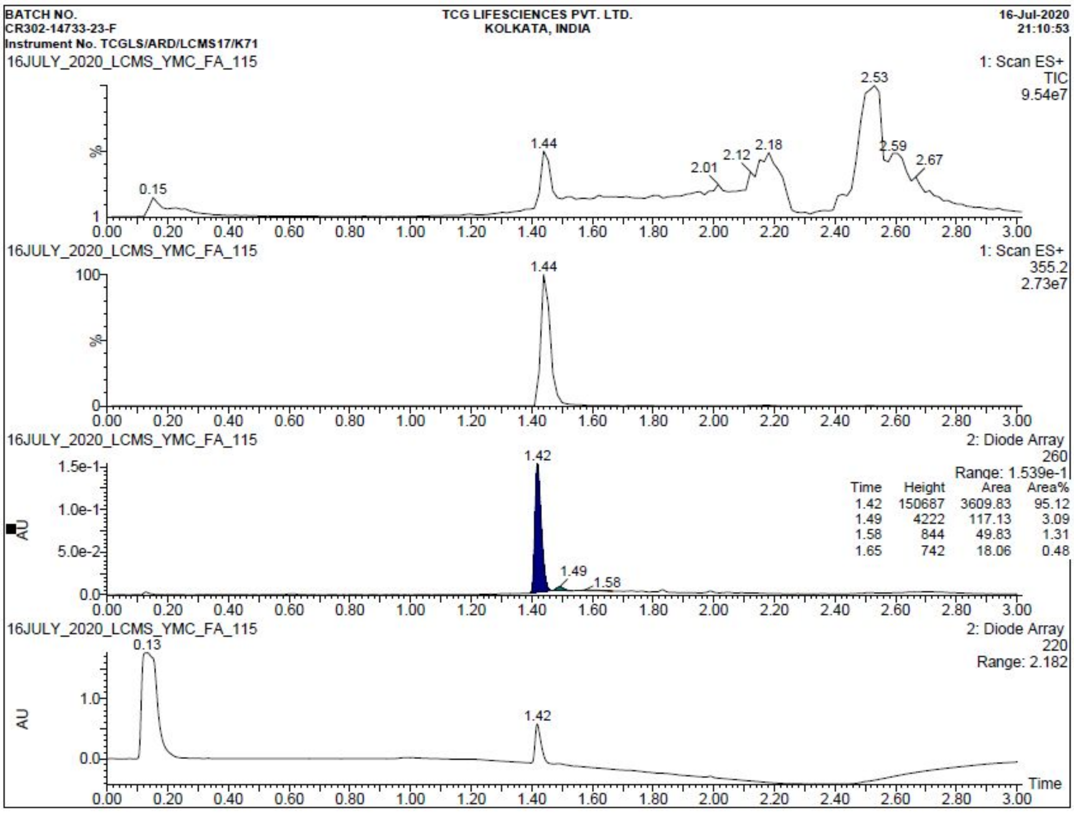
BATCH NO.
CR302-14733-23-F
Instrument No. TCGLS/ARD/LCMS17/K71
TCG LIFESCIENCES PVT. LTD.
KOLKATA, INDIA
16-Jul-2020
21:10:53
16JULY_2020_LCMS_YMC_FA_115
1: Scan ES+
TIC
9.54e7
355.2
2.73e7
2: Diode Array
260
Range: 1.539e-1
Time Height Area Area%
1.42 150687 3609.83 95.12
1.49 4222 117.13 3.09
1.58 844 49.83 1.31
1.65 742 18.06 0.48
220
Range: 2.182
Time

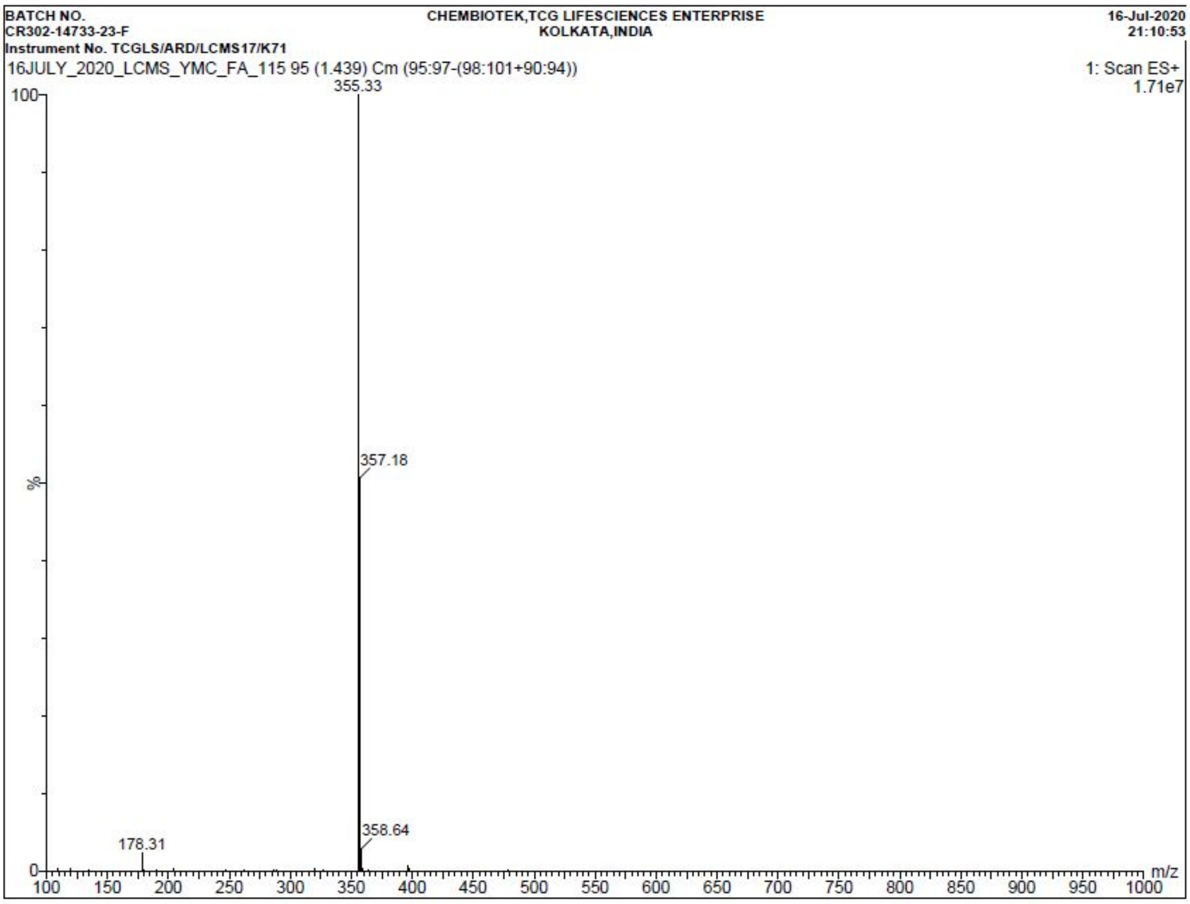
BATCH NO.
CR302-14733-23-F
Instrument No. TCGLS/ARD/LCMS17/K71
CHEMBIOTEK,TCG LIFESCIENCES ENTERPRISE
KOLKATA,INDIA
16-Jul-2020
21:10:53
16JULY_2020_LCMS_YMC_FA_115 95 (1.439) Cm (95:97-(98:101+90:94))
1: Scan ES+
1.71e7
355.33
357.18
358.64
178.31
m/z

LCMS-6v

	Resultados HRMS		
	Responsável:	Diego C Andrade	
	Data:	06/10/22	
	**Laboratório Institucional de Espectrometria de Massas**		

Prof. Dr. Luiz Carlos Dias
Aluno Anwar Shamim
Amostra MMV418

Espectro completo de 50 a 750 m/z em modo positivo

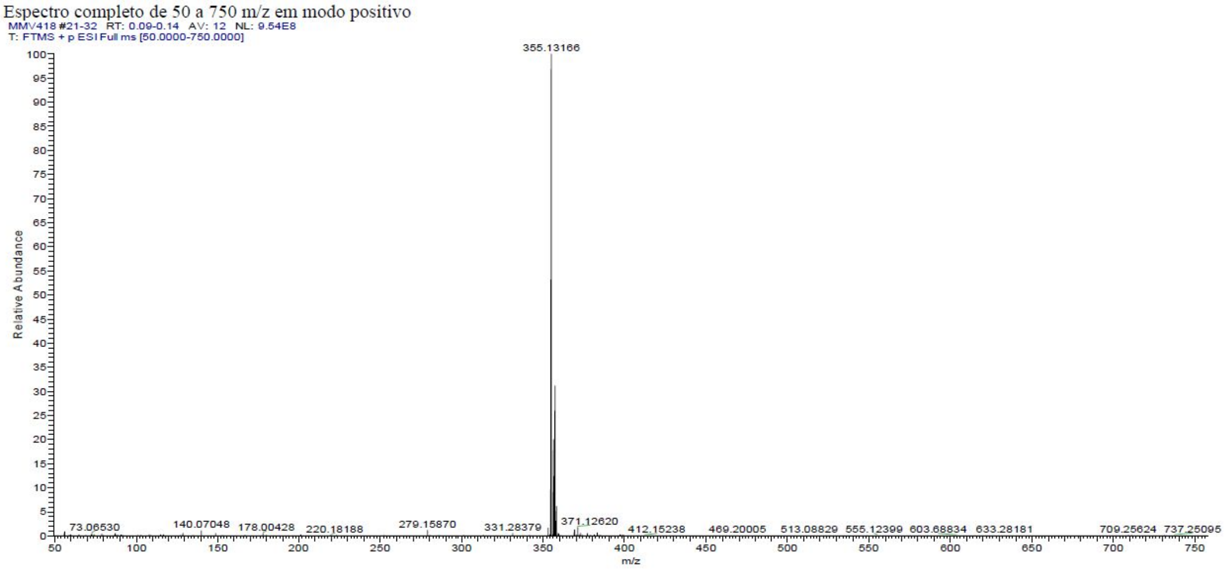

HRMS-6v

	Resultados HRMS		
	Responsável:	Diego C Andrade	
	Data:	06/10/22	
	**Laboratório Institucional de Espectrometria de Massas**		

Comparação entre espectro real (superior) e simulado (inferior) para C19H19ClN4OH+

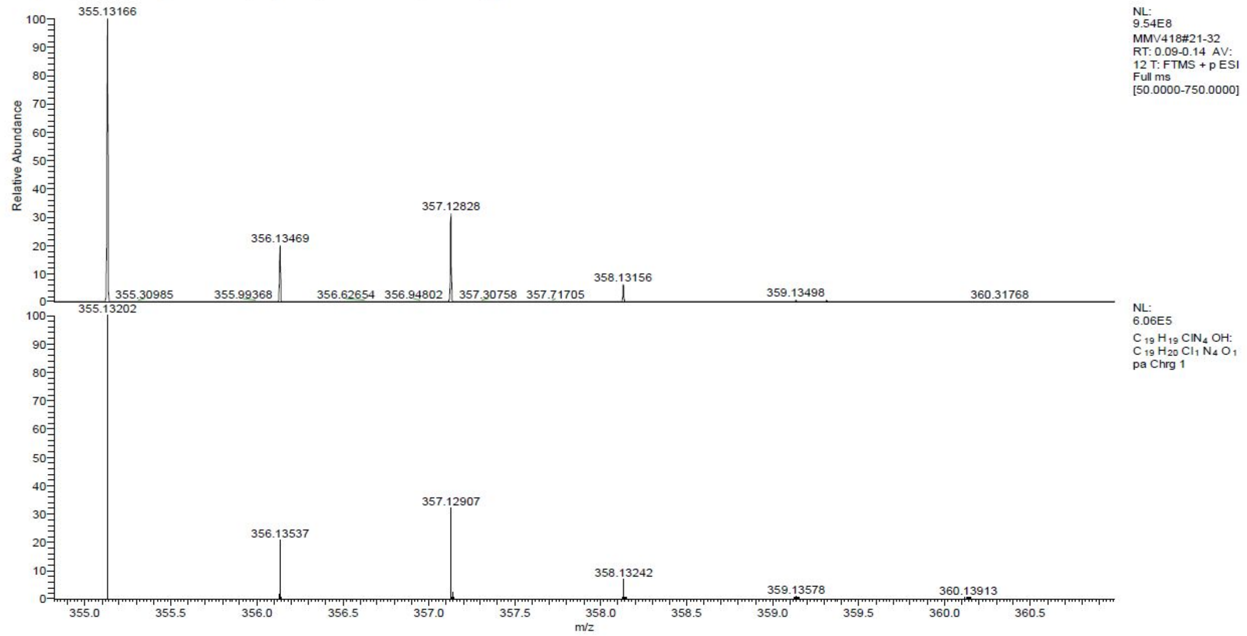

HRMS-6v

5-chloro-N-(4-piperidylmethyl)-N-(3-pyridylmethyl)-1H-indole-2-carboxamide (6w):

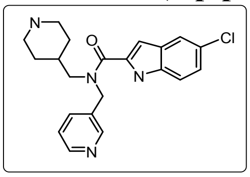

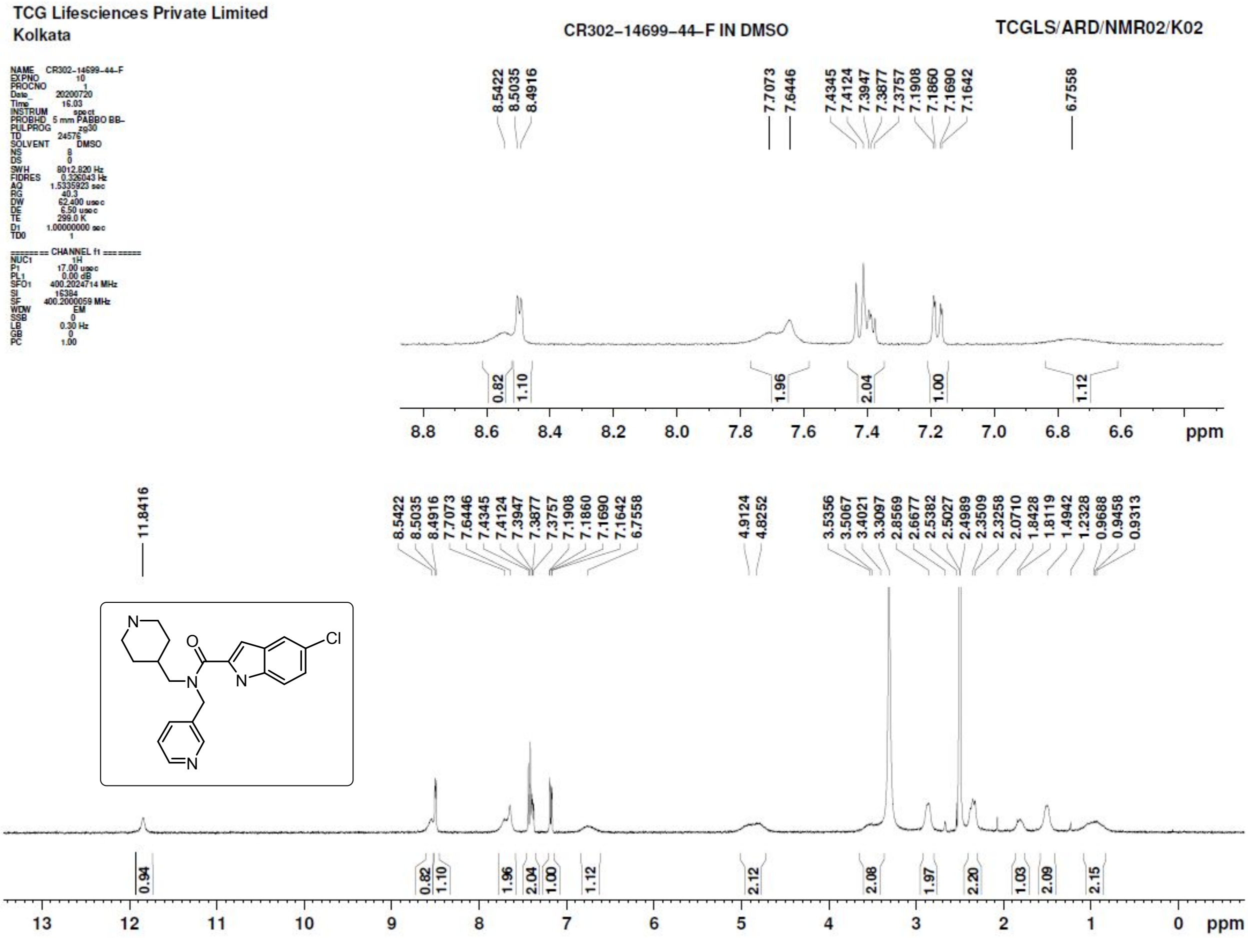

^{1}H NMR (400 MHz, DMSO-d_6)-6w

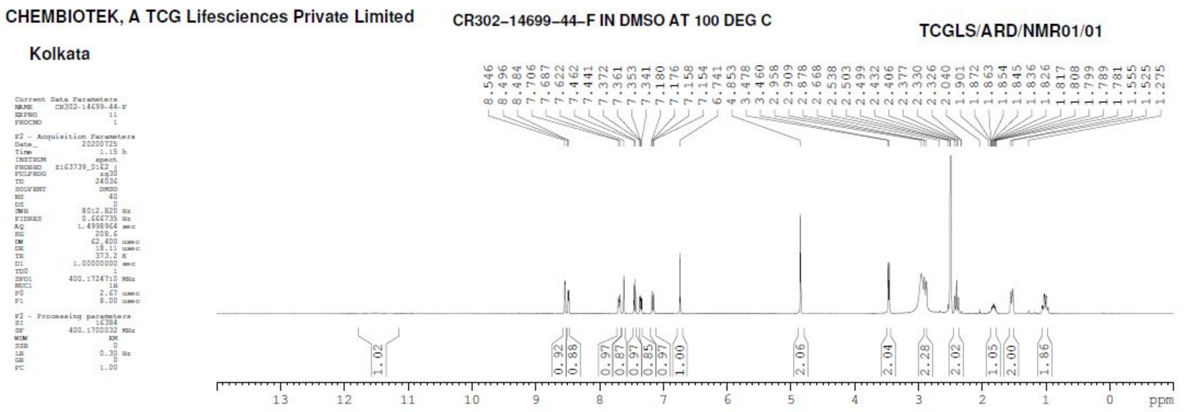

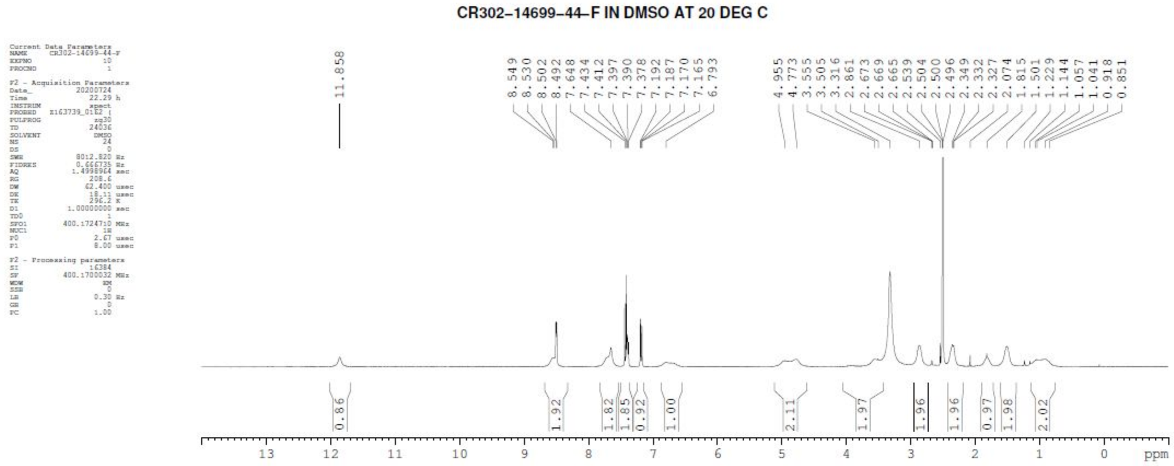

^{1}H NMR (20 °C and 100 °C) (400 MHz, DMSO-d_6)-6w

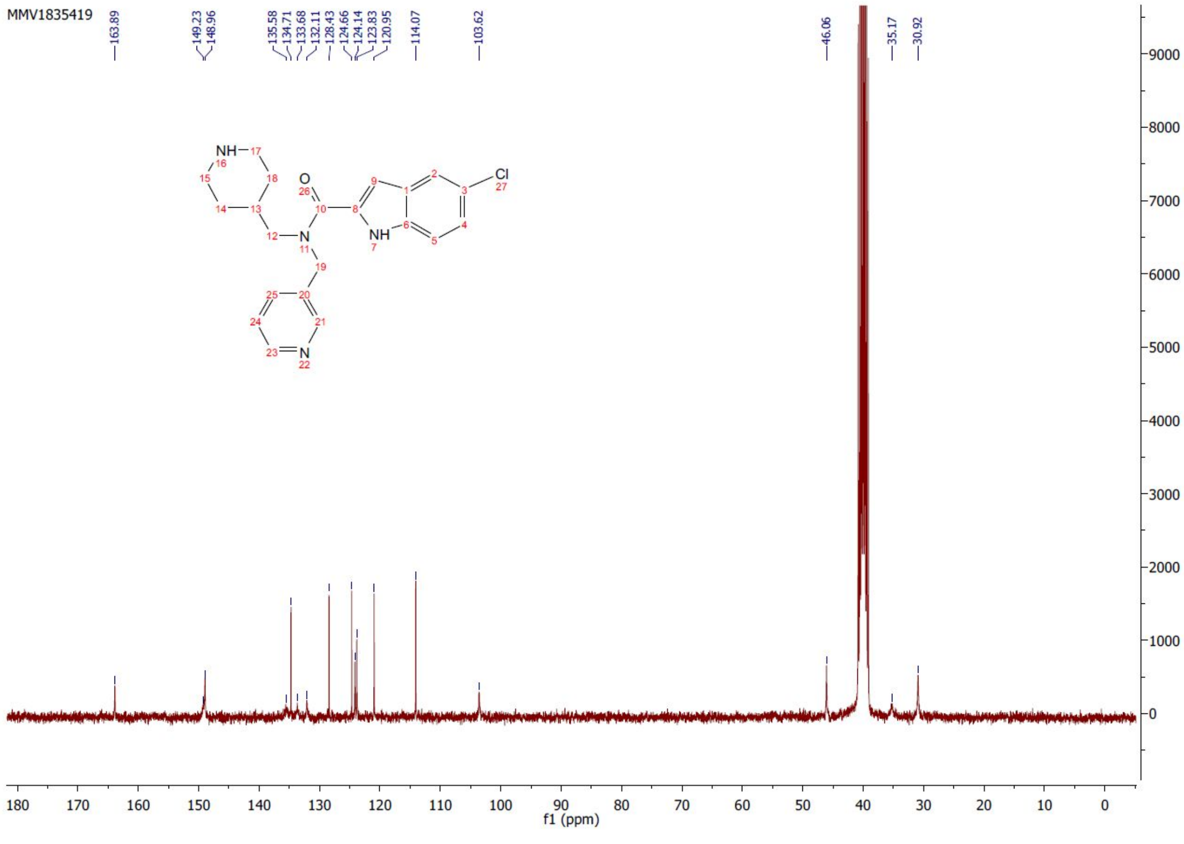

^{13}C NMR (75 MHz, DMSO-d_6)-6w

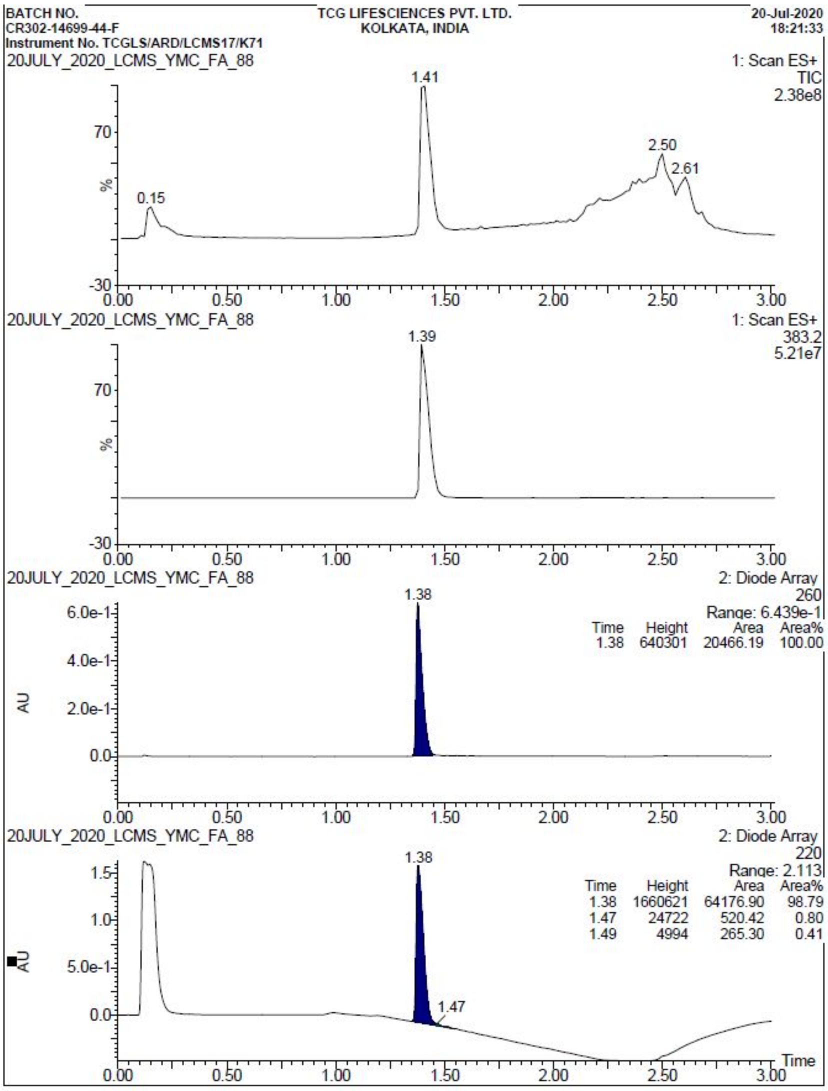
BATCH NO.
CR302-14699-44-F
Instrument No. TCGLS/ARD/LCMS17/K71
TCG LIFESCIENCES PVT. LTD.
KOLKATA, INDIA
20-Jul-2020
18:21:33
20JULY_2020_LCMS_YMC_FA_88
1: Scan ES+
TIC
2.38e8
1.41
2.50
2.61
0.15
1: Scan ES+
383.2
5.21e7
1.39
2: Diode Array
260
Range: 6.439e-1
Time Height Area Area%
1.38 640301 20466.19 100.00
1.38
2: Diode Array
220
Range: 2.113
Time Height Area Area%
1.38 1660621 64176.90 98.79
1.47 24722 520.42 0.80
1.49 4994 265.30 0.41
1.47
Time

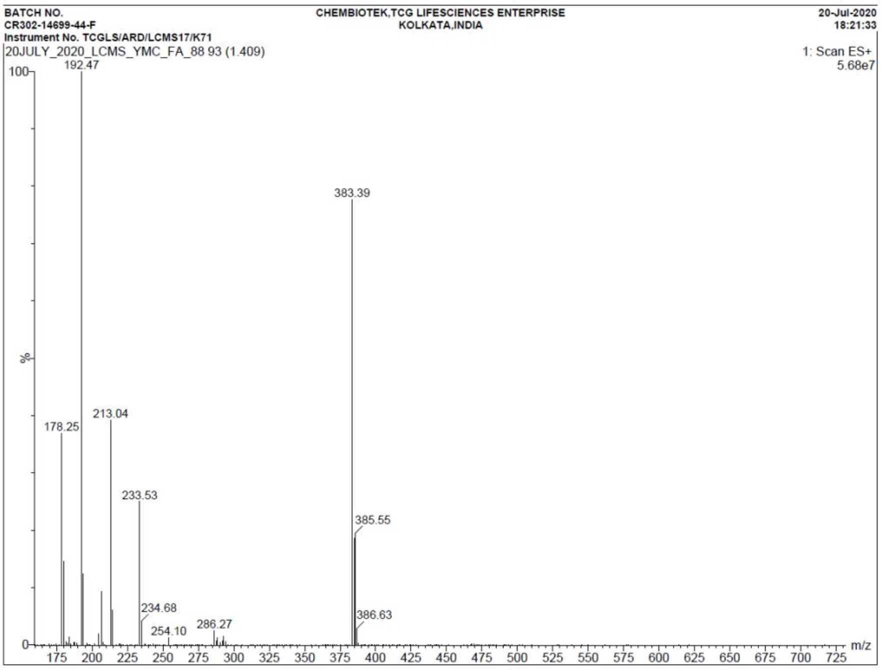
BATCH NO.
CR302-14699-44-F
Instrument No. TCGLS/ARD/LCMS17/K71
CHEMBIOTEK,TCG LIFESCIENCES ENTERPRISE
KOLKATA,INDIA
20-Jul-2020
18:21:33
20JULY_2020_LCMS_YMC_FA_88 93 (1.409)
1: Scan ES+
5.68e7
192.47
383.39
178.25
213.04
233.53
385.55
386.63
234.68
254.10
286.27
m/z

LCMS-6w

Resultados HRMS	
Responsável:	Diego C Andrade
Data:	06/10/22
**Laboratório Institucional de Espectrometria de Massas**	

Prof. Dr. Luiz Carlos Dias
Aluno Anwar Shamim
Amostra MMV419

Espectro completo de 50 a 750 m/z em modo positivo

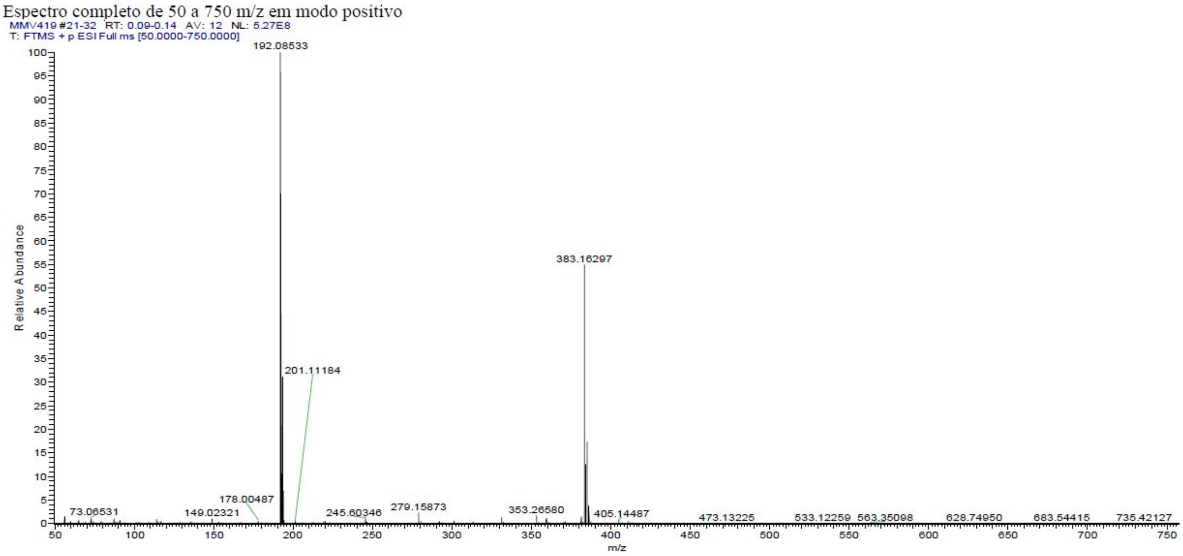

HRMS-6w

Resultados HRMS	
Responsável:	Diego C Andrade
Data:	06/10/22
**Laboratório Institucional de Espectrometria de Massas**	

Comparação entre espectro real (superior) e simulado (inferior) para C21H23ClN4OH+

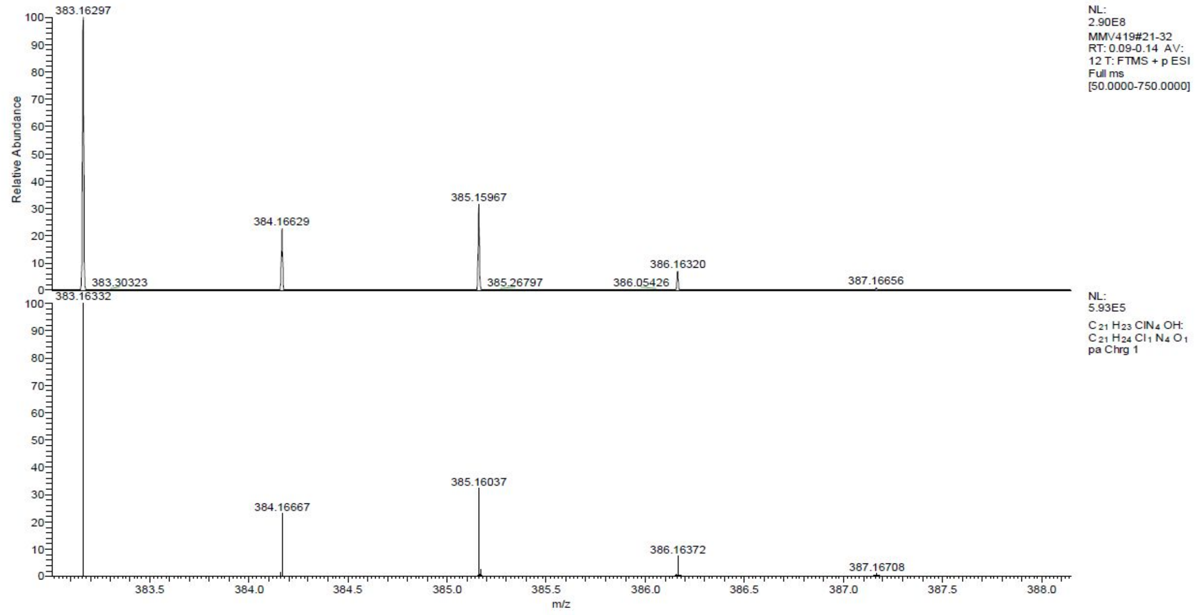

HRMS-6w

5-chloro-N-(piperidin-4-yl)-N-(pyridin-3-ylmethyl)-1H-indole-2-carboxamide hydrochloride (6x)

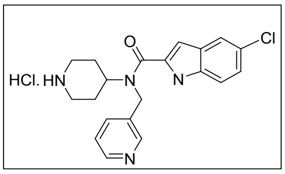

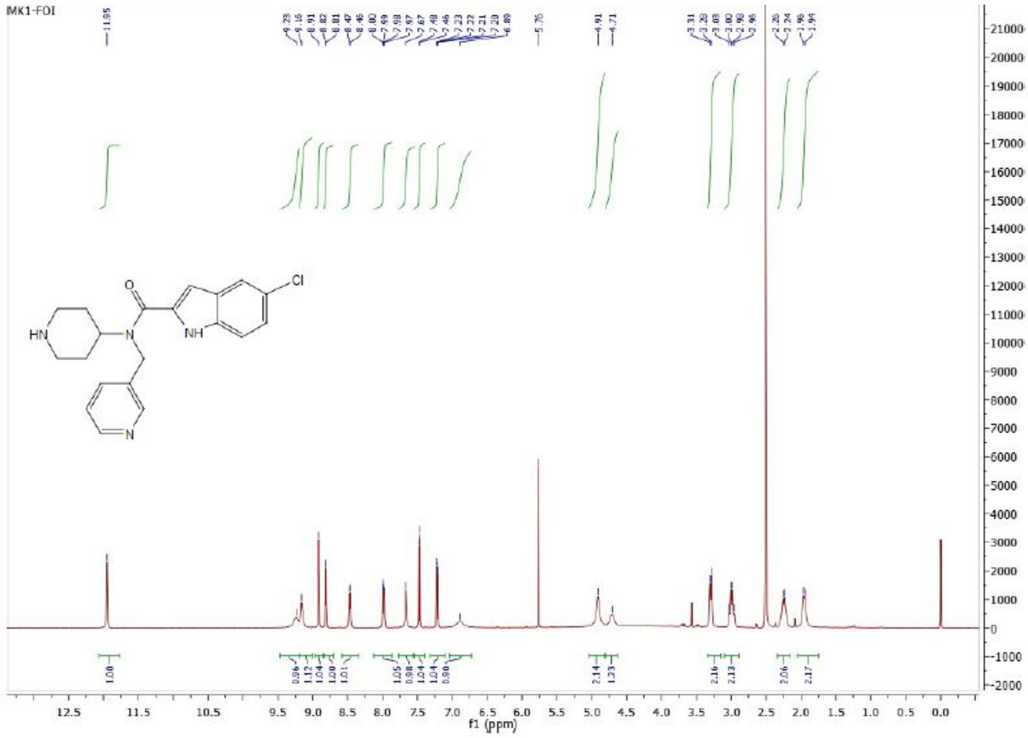

^{1}H NMR (500 MHz, DMSO-d_6)-6x

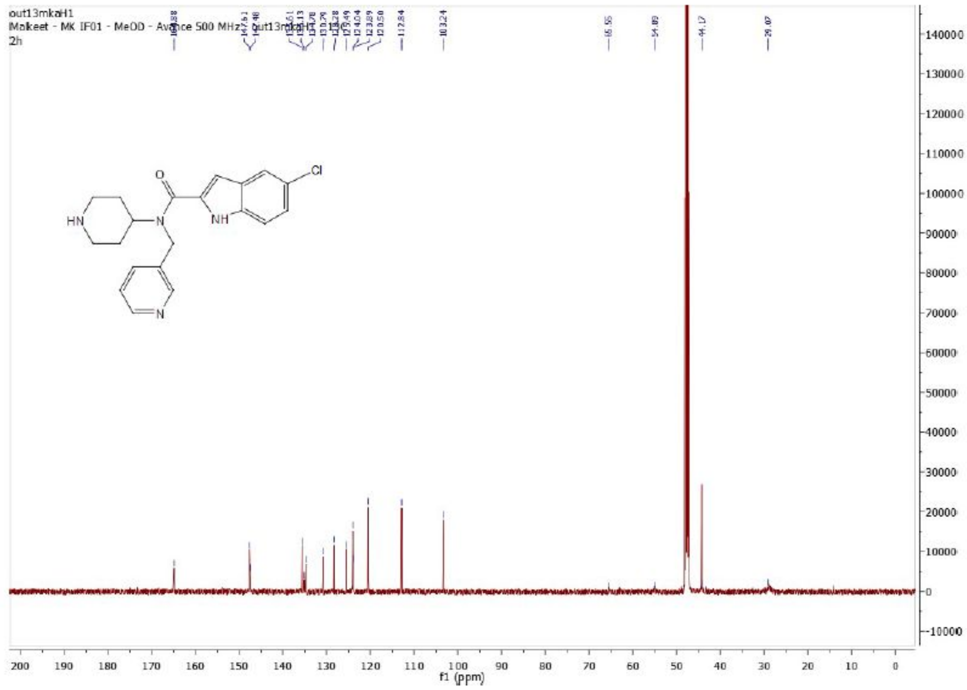

^{13}C NMR (126 MHz, CD_3OH)-6x

Resultados HRMS	
Responsável:	Diego C Andrade
Data:	12/04/2024
**Laboratório Institucional de Espectrometria de Massas**	

Prof. Dr. Luiz Carlos Dias
Aluno Anees Ahmad
Amostra MMV-649

Espectro completo de 50 a 750 m/z em modo positivo

MMV-649 #18-37 RT: 0.08-0.16 AV: 20 NL: 1.20E9
T: FTMS + p ESI Full ms [50.0000-750.0000]

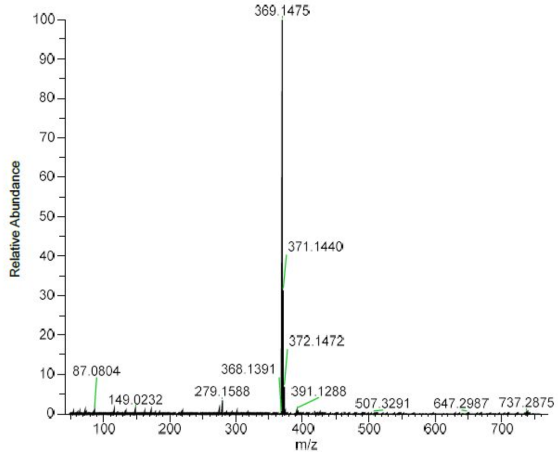

HRMS-6x

Resultados HRMS	
Responsável:	Diego C Andrade
Data:	12/04/2024
**Laboratório Institucional de Espectrometria de Massas**	

Comparação entre espectro real (superior) e simulado (inferior)

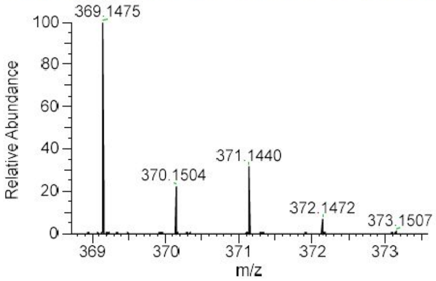

NL: 1.20E9
MMV-649 #18-37 RT: 0.08-0.16 AV: 20 NL: 1.20E9
T: FTMS + p ESI Full ms [50.0000-750.0000]

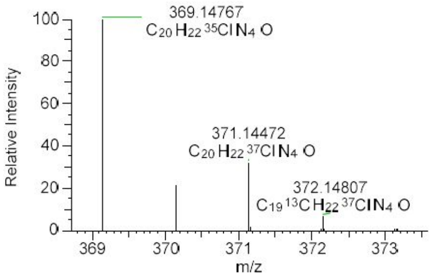

NL: 5.99E5
C20H21ClN4OH Chg: +1: $C_{20}H_{22}ClN_4O$
pa Chrg 1 Pattern

HRMS-6x

N-(piperidin-4-yl)-N-(pyridin-3-ylmethyl)-5-(trifluoromethyl)-1H-indole-2-carboxamide (6y)

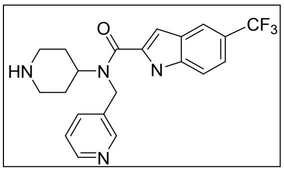

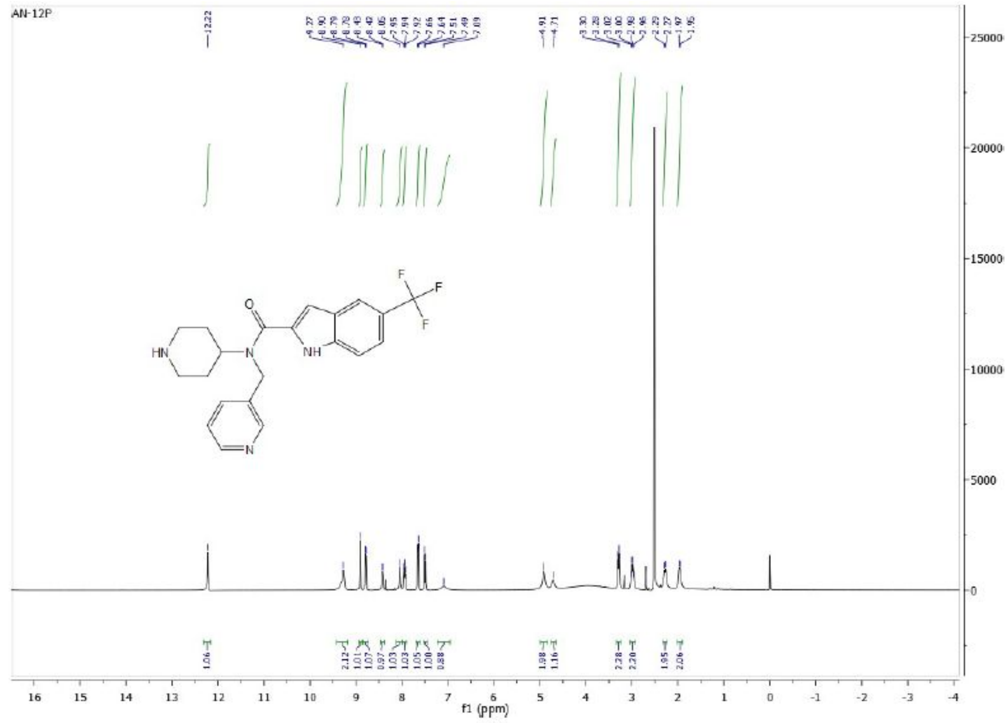

^{1}H NMR (500 MHz, DMSO-d_6)-6y

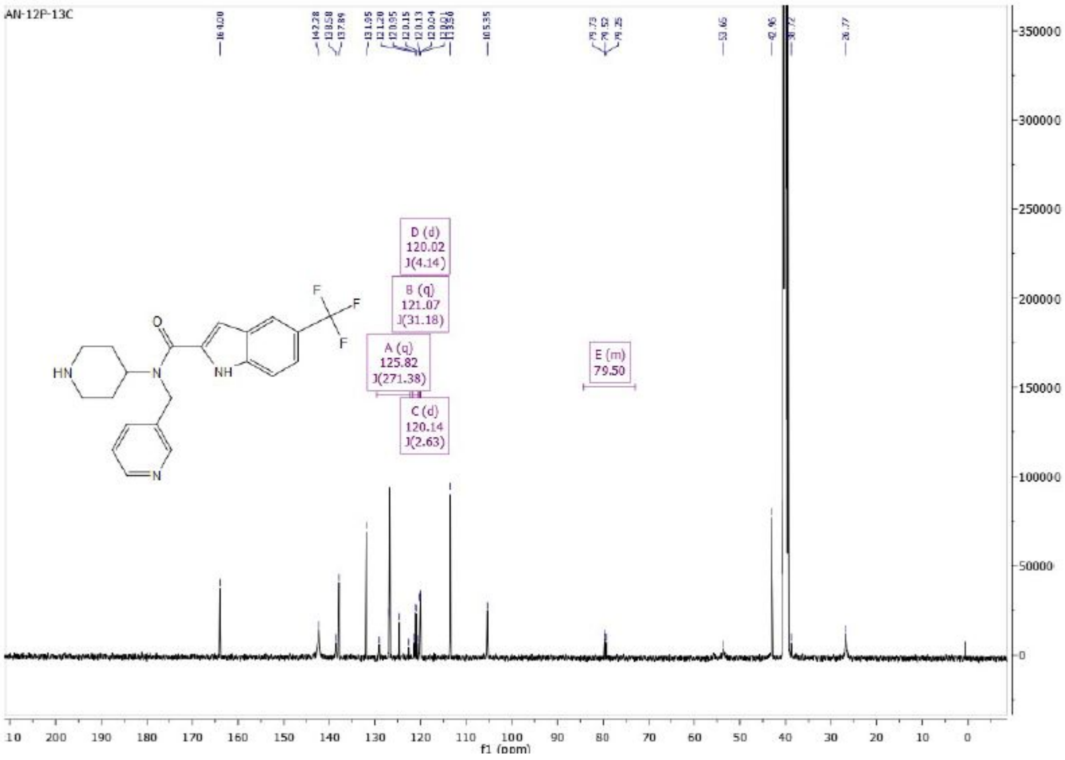

^{13}C NMR (126 MHz, DMSO-d_6)-6y

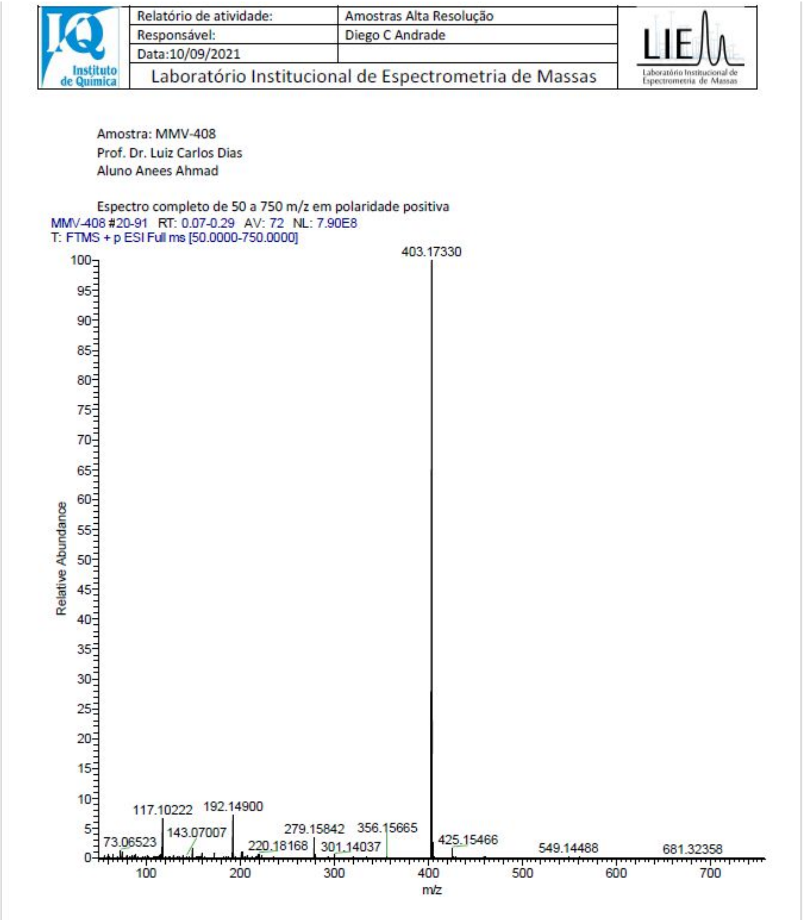

HRMS-6y

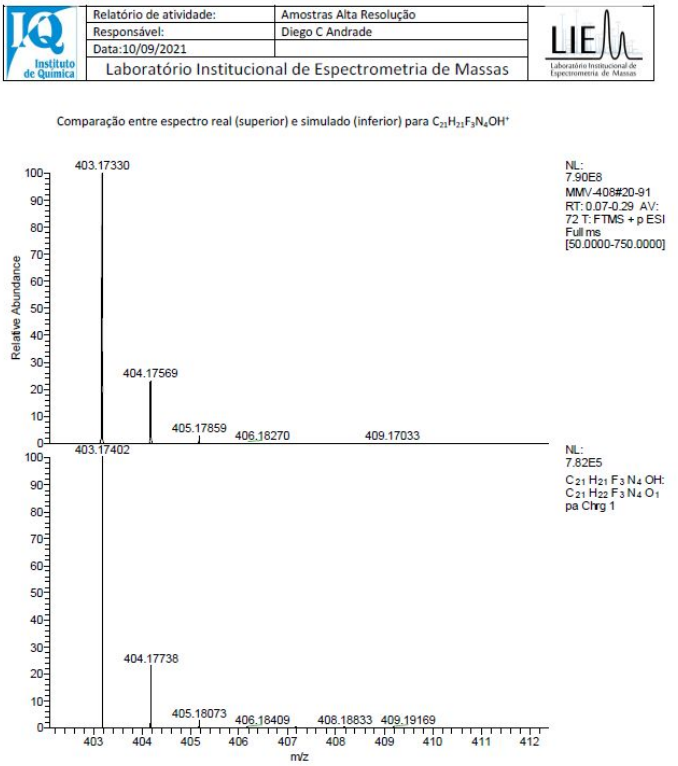

HRMS-6y

5-chloro-N-[(1-methylpyrrolidin-3-yl)methyl]-N-(pyridin-3-ylmethyl)-1H-indole-2-carboxamide (7a):

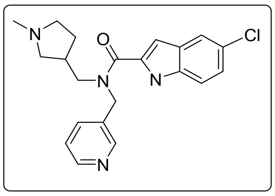

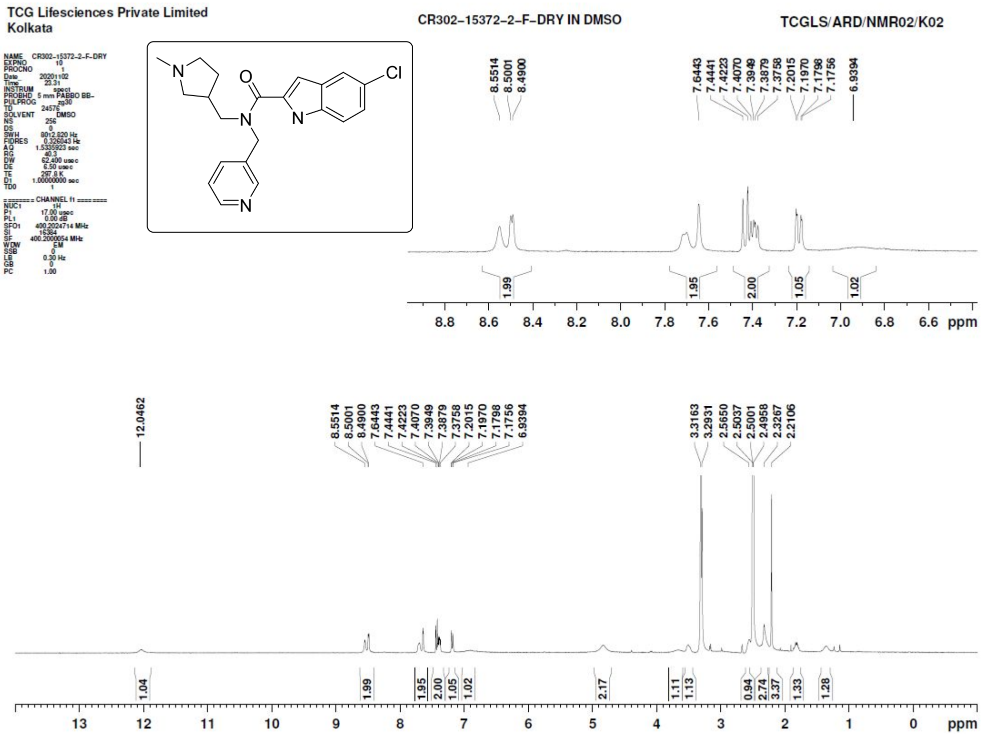

^{1}H NMR (400 MHz, DMSO-d$_6$)-7a

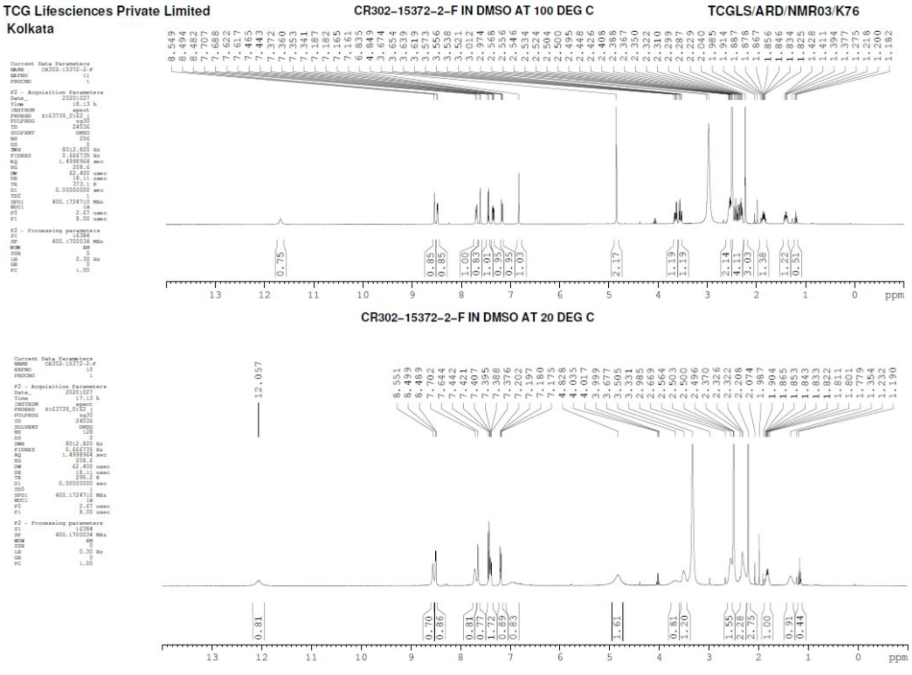

^{1}H NMR (20 °C and 100 °C) (400 MHz, DMSO-d$_6$)-7a

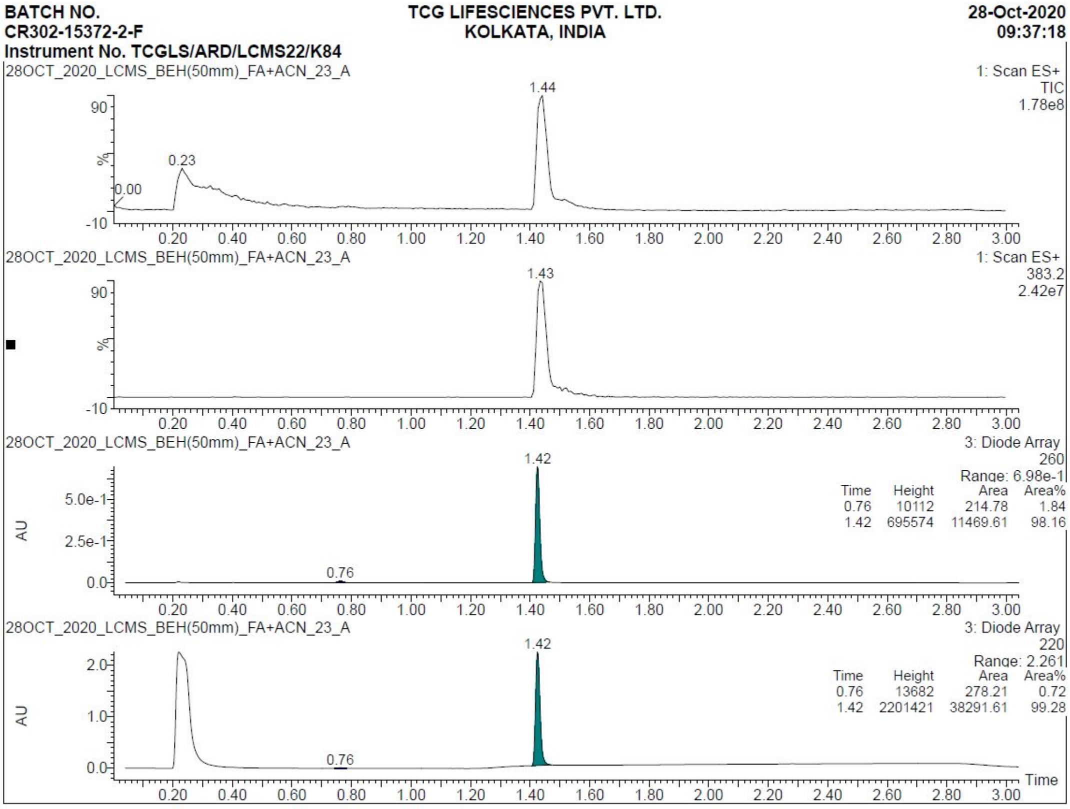
BATCH NO.
CR302-15372-2-F
Instrument No. TCGLS/ARD/LCMS22/K84
TCG LIFESCIENCES PVT. LTD.
KOLKATA, INDIA
28-Oct-2020
09:37:18
28OCT_2020_LCMS_BEH(50mm)_FA+ACN_23_A
1: Scan ES+
TIC
1.78e8
1.44
0.23
0.00
1.43
383.2
2.42e7
3: Diode Array
260
Range: 6.98e-1
Time Height Area Area%
0.76 10112 214.78 1.84
1.42 695574 11469.61 98.16
1.42
0.76
220
Range: 2.261
Time Height Area Area%
0.76 13682 278.21 0.72
1.42 2201421 38291.61 99.28
Time

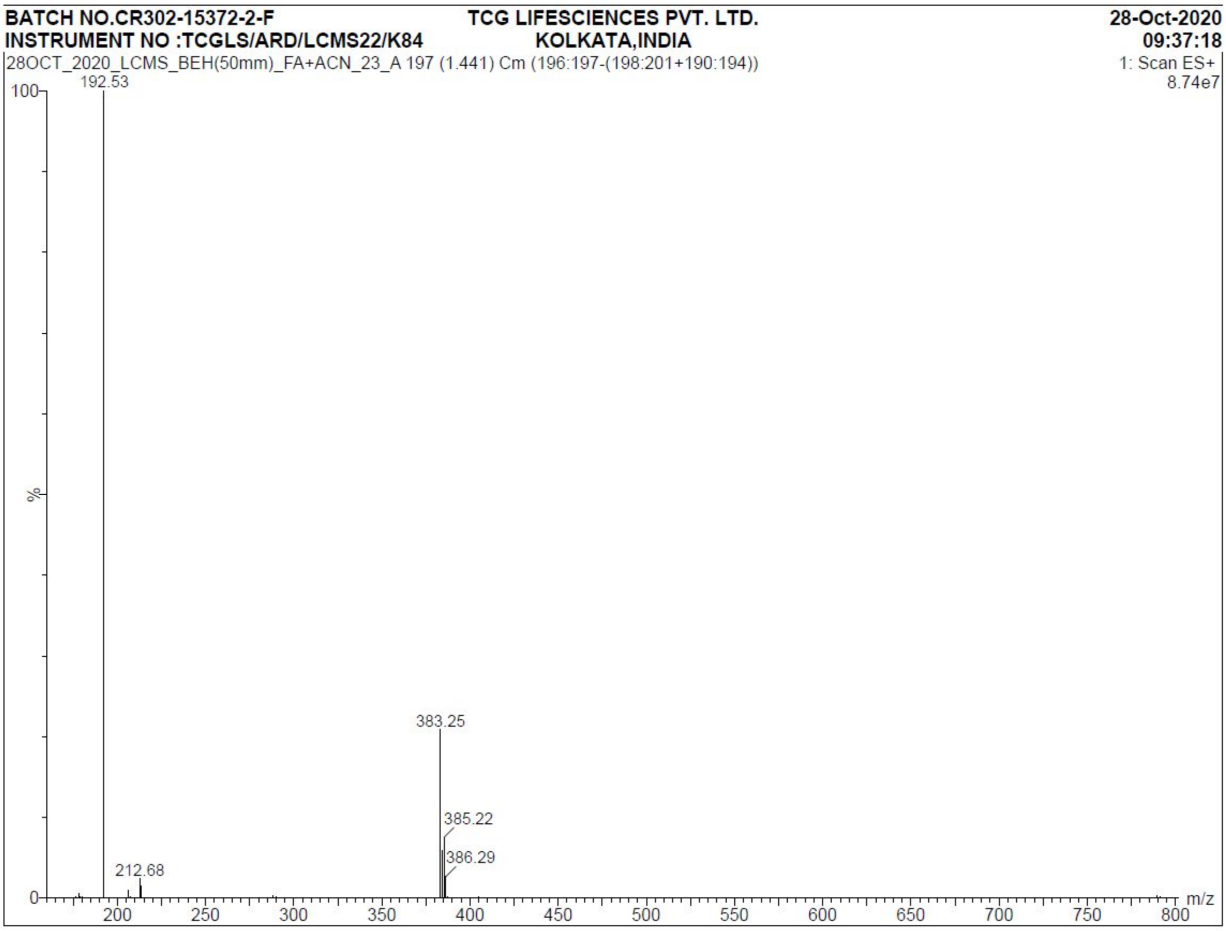
BATCH NO.CR302-15372-2-F
INSTRUMENT NO :TCGLS/ARD/LCMS22/K84
TCG LIFESCIENCES PVT. LTD.
KOLKATA,INDIA
28-Oct-2020
09:37:18
28OCT_2020_LCMS_BEH(50mm)_FA+ACN_23_A 197 (1.441) Cm (196:197-(198:201+190:194))
1: Scan ES+
8.74e7
192.53
212.68
383.25
385.22
386.29
m/z

LCMS-7a
